# Design, *in Vitro* Biological Activities and Molecular Dynamics Simulations of Novel Chalcone and Pyrazole Derivatives Targeting α‑Glucosidase and PPAR‑γ as Antidiabetic Agents

**DOI:** 10.1021/acsomega.6c02853

**Published:** 2026-08-14

**Authors:** Bedriye Seda Kurşun Aktar, Demet Taşdemir, Ebru Sağlam, Süleyman Kaya, Gizem Tatar Yılmaz, Ayse Şahin Yağlioğlu, Emine Elçin Oruç-Emre

**Affiliations:** † Department of Engineering Basic Sciences, Faculty of Engineering and Natural Sciences, 531771 Malatya Turgut Özal University, Malatya 44900, Turkiye; ‡ Respiratory Diseases and Respiratory Surgery Research and Practice Center, 37512 Gaziantep University, Gaziantep 27310, Turkiye; § Department of Medical Biochemistry, Faculty of Medicine, Gaziantep University, Gaziantep 27310, Turkiye; ∥ Department of Chemistry, Graduate School of Natural and Applied Sciences, Gaziantep University, Gaziantep 27310, Turkiye; ⊥ Institute of Health Sciences, Department of Bioinformatics, 52976 Karadeniz Technical University, Trabzon 61080, Turkiye; # Department of Biostatistics and Medical Informatics, Faculty of Medicine, Karadeniz Technical University, Trabzon 61080, Turkiye; ¶ Yılmaz Bilişim R&D Consulting Software Engineering and Services Trade Limited Company, Trabzon 61081, Turkiye; ∇ Department of Chemistry and Chemical Process Technology, Technical Sciences Vocational School, 111366 Amasya University, Amasya 05100, Turkiye; ○ Department of Chemistry, Faculty of Arts and Sciences, Gaziantep University, Gaziantep 27310, Turkiye

## Abstract

Diabetes mellitus (diabetes) is a chronic disease that is rapidly increasing worldwide. In 2019, there were 463 million patients globally, and this number is expected to reach 578 million in 2030 and 700 million in 2045. This necessitates the design of new drugs. When researching new drug synthesis methods worldwide, rather than synthesizing substances with unknown properties, research starts with substances whose efficacy is known or examines the pharmacotherapeutic effects of a drug used to treat one disease, considering that it may also be effective in other diseases. Following this strategy, acetohexamide, a compound found in the structure of glibenclamide, currently used as a second-generation antidiabetic drug and previously used as a first-generation antidiabetic drug, was selected as the starting material. Based on this compound, compounds with chalcones [(1,3-diaryl)-2-propen-1-one], known as members of the flavonoid family, and compounds with a pyrazole ring obtained by cyclization of chalcones have been designed to be pharmacologically active, safe, and, most importantly, suitable for oral use. Using molecular dynamics simulations, highly effective compounds were synthesized from these compounds, and their antidiabetic and cytotoxic activities targeting α-glucosidase and PPAR-γ were evaluated. Compounds **44** and **45** exhibited high cell viability in HEK 293 cells (IC_50_ > 100 μM) and showed no α-glucosidase inhibition. Both compounds induced concentration-dependent activation of PPAR-γ, with compound **45** exhibiting moderate activity. Notably, compound **44** significantly enhanced cell migration while remaining noncytotoxic, highlighting its potential for further investigation.

## Graphical abstract

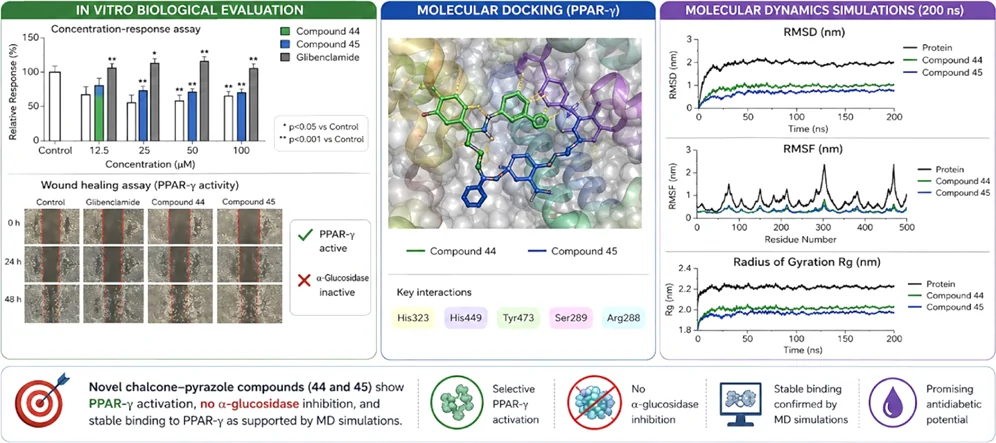

## 1 Introduction

Diabetes is a chronic disease characterized by insufficient insulin production, resulting in hyperglycemia. If left untreated, it causes problems such as vision loss, kidney failure, heart attack, stroke, and limb amputations. In 2021, 1.6 million people died from this disease. Today, it affects approximately 10.5% of the world’s population.^1^

α-Glucosidases are a group of hydrolase enzymes that convert complex sugars into simple glucose by hydrolyzing glycosidic bonds and play a critical role in carbohydrate metabolism. α-Glucosidases are found in the epithelium of the small intestinal mucosa. They catalyze the release of α-d-glucopyranose from the nonreducing ends of substrates. They play a role in the processing of glycoproteins and glycolipids by cleaving terminal nonreducing 1,4-linked α-glucose.^2–4^

Glucose and lipid metabolism are regulated by the peroxisome proliferator-activated receptor gamma (PPARγ). This receptor requires ligands to exhibit activity. After ligand binding, the receptor combines with retinoid X receptor-α (RXRα) to form a heterodimer. The effects of various genes on PPARγ are assessed by measuring heterodimer formation. Although thiazolidinediones are well-known PPARγ ligands used in diabetes therapy, their clinical application is limited by adverse effects.^5^ Consequently, there is a growing interest in identifying safer PPARγ modulators, such as partial agonists or selective PPARγ modulators, that retain therapeutic efficacy while minimizing side effects.

Chalcone contains the 1,3-diphenyl-2-propen-1-one structure, where a three-carbon structure joins two aromatic rings with an α,β-unsaturated carbonyl system. Chalcone derivatives possess biological activities such as antidiabetic, antineoplastic, antiinflammatory, antimalarial, antioxidant, antigout, and antibacterial.^6^ Furthermore, chalcones demonstrated antidiabetic activity through inhibition of aldose reductase, DPP-4, PTP1B, PPAR-γ, α-glucosidase.^2–4,7,8^

Pyrazoles are an important class of nitrogen-containing heterocyclic compounds with pharmacological significance.^9^ Pyrazoles are found in the structure of many drugs that have activities such as antibiotic, antifungal, antibacterial, antioxidant, anti-inflammatory, antiviral, anticancer, antidepressant and antidiabetic.^10–12^ At the same time, pyrazole derivatives exhibit α-glucosidase inhibitors^13,14^ and PPAR-γ agonist effects in the treatment of type II diabetes.^15^ Figure 1 shows the structures of drugs containing a pyrazole ring used in treatment.

**Figure 1:**
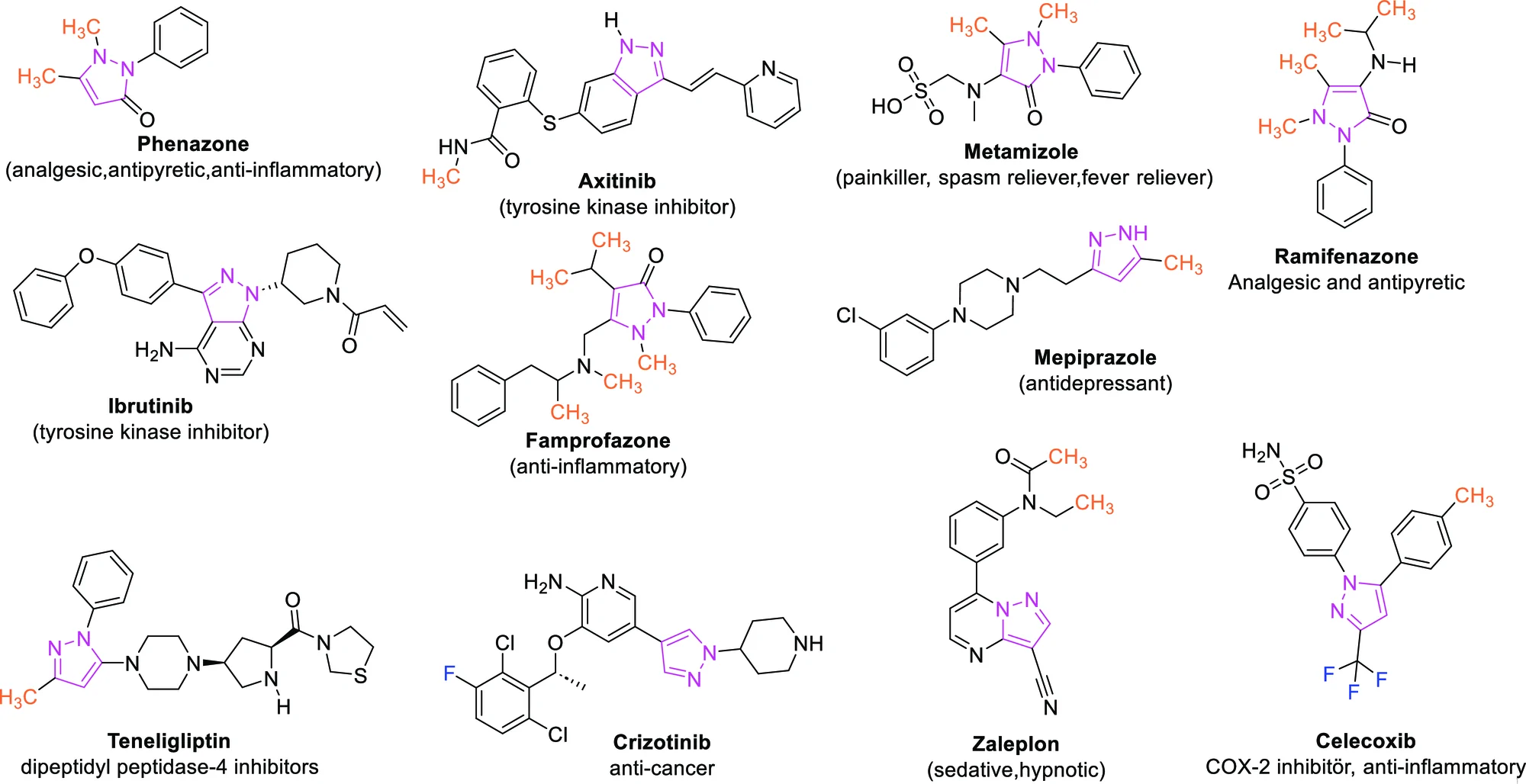
Some marketed drugs containing an pyrazoline-based group.

Numerous studies have reported that novel derivatives containing a pyrazole ring exhibit strong activation of PPAR-γ while simultaneously displaying inhibitory effects on α-glucosidase. Molecular modeling studies, in particular, have shown that the position of the pyrazole ring, side chain length, and the presence of functional groups are decisive factors in determining the binding affinity with enzymes.^16^ In this study, these novel compounds containing a pyrazole ring were rationally designed as multitarget molecules combining a PPAR-γ agonist and α-glucosidase inhibitor on the same molecule for the treatment of diabetes. The pyrazole core stands out as a scaffold capable of both hydrogen bonding and hydrophobic interactions due to its aromatic and heteroatom-containing structure. These properties allow for the optimization of the ligand’s interaction with critical amino acids in the receptor pocket. During the design process, a sulfonylurea group was placed at one end, a pyrazole ring in the middle, and hydrophobic/aromatic substituents at the other end to increase both the binding affinity and selectivity of the molecule. With this approach, the aim was for these designed compounds to be multitarget antidiabetic drug candidates that both increased insulin sensitivity and slowed carbohydrate digestion in the small intestine.^17–22^ The structures of molecules containing a pyrazole ring that exhibit PPAR-γ agonist and α-glucosidase inhibitory effects are given in Figure 2.^23–28^

**Figure 2:**
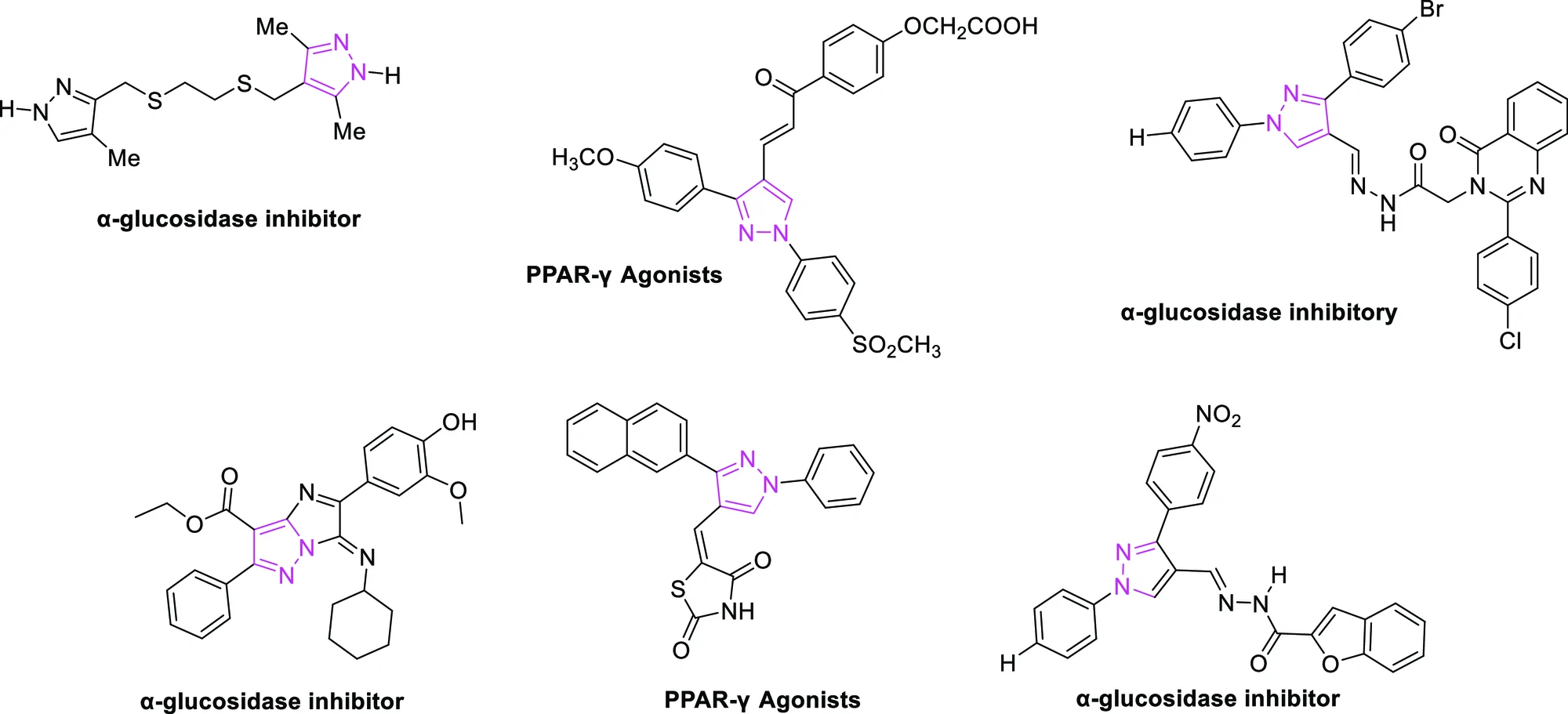
Chemical structures of molecules containing a pyrazole ring as PPAR-γ agonists and α-glucosidase inhibitors.

Given that both chalcone and pyrazole scaffolds independently exhibit significant antidiabetic properties through complementary mechanisms of action,^9^ the rational design of pyrazole and chalcone molecules represents a promising strategy for the development of novel antidiabetic agents. Following this strategy, in order to develop new generation antidiabetic agents, unique sulfonylurea-pyrazoline hybrids were designed by modifying acetohexamide, one of the precursor forms of the existing glibenclamide structure.

Furthermore, acetohexamide, chosen as the starting material, was selected due to its clinically proven antidiabetic activity and the presence of a sulfonylurea pharmacophore in its structure.^29^ Additionally, a pyrazole ring was incorporated as a key heterocyclic skeleton in the molecular design to enhance its binding affinity to the PPAR-γ active site. The pyrazole ring was chosen because of its ability to participate in hydrogen bonding, π–π stacking, and hydrophobic interactions. Various electron-donating and electron-withdrawing substituents were added to different positions of the aromatic ring at position 4 of the pyrazole ring. These substituents were selected to systematically investigate their effects on electronic properties, steric factors, hydrophobic interactions, and potential hydrogen bonding capabilities within the PPAR-γ binding pocket. In this context, 159 compounds were designed using ligand-based and structure-based drug design approaches, and a virtual library was created from these compounds.^30^ The compounds (**1–159**) were evaluated through molecular docking studies against PPAR-γ and α-glucosidase. The compounds were ranked according to their binding scores and protein–ligand interaction profiles. Based on these results, 17 candidate compounds **4**, **28**, **28A**, **44**, **45**, **46**, **47**, **49**, **54**, **57**, **64**, **65**, **66**, **67**, **68**, **70**, and **70A** with the most promise for synthesis and biological evaluation were selected. In the present study, these compounds were synthesized for the first time and evaluated for their potential activity against α-glucosidase and PPAR-γ. Additionally, their *in vitro* cytotoxic effects were investigated in HEK cells. Furthermore, molecular docking studies were performed on compounds **44** and **45**, which demonstrated notable activity toward PPAR-γ based on the *in vitro* findings.

## 2 Experimental Section

### 2.1 Materials and Methods

All chemicals and solvents were of analytical grade and obtained from Acros, Alfa Aesar, Sigma-Aldrich, and Merck. All reagents were used as received without further purification unless otherwise specified. The progress of the reactions was monitored by thin-layer chromatography (TLC) on silica gel 60 F254 plates. Melting points were determined using an EZ-Melt MPA120 automated melting point apparatus and are reported uncorrected. Fourier-transform infrared (FTIR) spectra were recorded on Cary 630 FTIR and PerkinElmer Frontier (model 1620) spectrometers equipped with an attenuated total reflectance (ATR) accessory. ^1^H and ^13^C NMR spectra were recorded on a Bruker spectrometer operating at 400 and 600 MHz. Tetramethylsilane (TMS) was used as the external reference, and chemical shifts (δ) are expressed in parts per million (ppm). Elemental analyses (C, H, N, and S) were performed using a Thermo Scientific Flash 2000 Organic Elemental Analyzer. Mass spectra were obtained using an Agilent Technologies 1260 Infinity II LC–MS/MS system coupled to a 6460 Triple Quadrupole Mass Spectrometer, with electrospray ionization (ESI) as the ionization technique.

Antidiabetic enzyme inhibition assays were carried out using a SpectraMax 340PC384 96-well microplate reader (Molecular Devices, USA).

### 2.2 General Synthesis Method of (*E*)-*N*-(Cyclohexylcarbamoyl)-4-(3-(Substituted)­acryloyl) Benzenesulfonamide Chalcone Compounds

Substituted benzaldehyde (0.01 mol) and *N*′-(1-[4-acetylphenyl)­sulfonyl-3-cyclohexylurea] (acetohexamide, 0.01 mol) were dissolved in methanol (25 mL) and stirred at room temperature. Solid NaOH (3 equiv) was added gradually. The reaction progress was monitored by TLC. Upon completion, the reaction mixture was extracted with dichloromethane, and the organic phase was dried over anhydrous MgSO_4_. Solvent removal under reduced pressure afforded the crude product, which was recrystallized from dichloromethane/*n*-hexane.^31^

#### 2.2.1 (*E*)-*N*-(Cyclohexylcarbamoyl)-4-(3-(*p*-Tolyl)­acryloyl)­benzenesulfonamide (2)

**Yield:** 80%. White solid. **Mp:** 219–220 °C. **FTIR (ν**_**max**_ **, cm** ^**–1**^ **):** 3348, 3081 (N–H); 2927, 2855 (C–H); 1691, 1666 (C=O); 1332/1167 (asymmetric/symmetric SO_2_). ^**1**^ **H NMR (600 MHz) (DMSO-*d***_**6**_ **/TMS):** δ 1.06–1.12 (m, 3H); 1.15–1.21 (m, 2H); 1.45 (d, 1H, *J* = 13.20 Hz); 1.56 (d, 2H, *J* = 13.20 Hz); 1.63 (d, 2H, *J* = 9.00 Hz); 2.33 (s, 3H, CH_3_ Hz); 3.25 (d, 1H, *J* = 7.20 Hz); 6.45 (d, 1H, *J* = 7.80 Hz, NH); 7.26 (s, 2H); 7.74 (d, 1H, *J* = 15.60 Hz); 7.77 (d, 2H, *J* = 7.80 Hz); 7.85 (d, 1H, *J* = 15.60 Hz); 8.02 (d, 2H, *J* = 9.00 Hz); 8.27 (d, 2H, *J* = 8.40 Hz); 10.53 (s, 1H, NH). ^**13**^ **C NMR (150 MHz) (DMSO-*d***_**6**_ **/TMS):** δ 189.1 (C=O); 21.6, 24.6, 25.4, 32.7, 48.6, 121.3, 125.7, 128.1, 129.4, 129.6, 130.0, 132.2, 141.5, 144.1, 145.7, 150.8. **Elemental Analysis:** C_23_H_26_N_2_O_4_S (426.53 g/mol) Anal. Calcd (%): C, 64.77; H, 6.14; N, 6.57; S, 7.52. Found (%): C, 64.85; H, 6.21; N, 6.65; S, 7.80.

#### 2.2.2 (*E*)-*N*-(Cyclohexylcarbamoyl)-4-(3-(4-Fluorophenyl)­acryloyl)­benzenesulfonamide (3)

**Yield:** 64%. Yellow solid. **Mp:** 216–217 °C. **FTIR (ν**_**max**_ **, cm** ^**–1**^ **):** 3305, 3236 (N–H); 2930, 2856 (C–H); 1681, 1611 (C=O); 1339/1161 (asymmetric/symmetric SO_2_). ^**1**^ **H NMR (600 MHz) (DMSO-*d***_**6**_ **/TMS):** δ 1.06–1.13 (m, 3H); 1.15–1.20 (m, 2H); 1.45 (d, 1H, *J* = 12.60 Hz); 1.56 (d, 2H, *J* = 13.20 Hz); 1.62 (d, 2H, *J* = 11.40 Hz); 3.25 (d, 1H, *J* = 7.20 Hz); 6.44 (d, 1H, *J* = 7.20, NH); 7.30 (t, 2H, *j*_*1*_ = 9.00, *j*_*2*_ = 9.00 Hz); 7.77 (d, 1H, *J* = 16.20 Hz); 7.87 (d, 1H, *J* = 15.60 Hz); 7.97 (dd, 2H, *j*_1_ = 5.40, *j*_2_ = 6.00 Hz); 8.03 (d, 2H, *J* = 8.40 Hz); 8.28 (d, 2H, *J* = 8.40 Hz); 10.52 (s, 1H). ^**13**^ **C NMR (150 MHz) (DMSO-*d***_**6**_ **/TMS):** δ 189.0 (C=O); 24.7, 25.4, 32.7, 48.6, 116.5, 122.3, 128.1, 129.5, 131.6, 131.9, 141.4, 144.3, 150.8, 163.2, 164.9. **Elemental Analysis:** C_22_H_23_FN_2_O_4_S (430.49 g/mol) Anal. Calcd (%): C, 61.38; H, 5.39; N, 6.51; S, 7.45. Found (%): C, 61.43; H, 5.47; N, 6.60; S, 7.51.

#### 2.2.3 (*E*)-4-(3-(4-Chlorophenyl)­acryloyl)-*N*-(cyclohexylcarbamoyl)­benzenesulfonamide (**4**)

**Yield:** 57%. Yellow solid. **Mp:** 214–215. **FTIR (ν**_**max**_ **, cm** ^**–1**^ **):** 3303, 3242 (N–H); 2938, 2856 (C–H); 1679, 1666 (C=O); 1338/1168 (asymmetric/symmetric SO_2_). ^**1**^ **H NMR (600 MHz) (DMSO-*d***_**6**_ **/TMS):** δ 1.06–1.11 (m, 3H, H_2,5_); 1.15–1.20 (m, 2H, H_3_); 1.45 (d, 1H, *J* = 9.00 Hz); 1.56 (d, 2H, *J* = 13.20 Hz); 1.62 (d, 2H, *J* = 12.60 Hz); 3.25 (d, 1H, *J* = 7.80 Hz); 6.45 (d, 1H, *J* = 7.80 Hz, NH); 7.52 (d, 2H, *J* = 8.40 Hz); 7.76 (d, 1H, *J* = 15.60 Hz); 7.92 (d, 2H, *J* = 9.00 Hz); 7.93 (d, 1H, *J* = 15.60 Hz); 8.03 (d, 2H, *J* = 9.00 Hz); 8.29 (d, 2H, *J* = 9.00 Hz); 10.53 (s, 1H, NH). ^**13**^ **C NMR (150 MHz) (DMSO-*d***_**6**_ **/TMS):** δ 189.0 (C=O); 24.6, 25.4, 32.7, 48.6, 123.1, 128.1, 129.5, 129.5, 131.2, 133.9, 135.9, 141.3, 144.1, 144.2, 150.8. **Elemental Analysis:** C_22_H_23_ClN_2_O_4_S (446.95 g/mol) Anal. Calcd (%): C, 59.12; H, 5.19; N, 6.27; S, 7.17. Found (%): C, 64.85; H, 6.21; N, 6.65; S, 7.80.

#### 2.2.4 (*E*)-*N*-(Cyclohexylcarbamoyl)-4-(3-(4-Ethoxyphenyl)­acryloyl)­benzenesulfonamide (**5**)

**Yield:** 81%. Yellow solid. **Mp:** 202–203 **°C. FTIR (ν**_**max**_ **, cm** ^**–1**^ **):** 3339, 3157 (N–H); 3067, 2933 (C–H); 1651 (C=O); 1353/1162 (asymmetric/symmetric SO_2_). ^**1**^ **H NMR (600 MHz) (DMSO-*d***_**6**_ **/TMS):** δ 1.06–1.11 (m, 3H); 1.15–1.20 (m, 2H); 1.45 (d, 1H, *J* = 12.00 Hz); 1.55 (d, 2H, *J* = 9.00 Hz); 1.63 (d, 2H, *J* = 8.40 Hz); 3.25 (d, 1H, *J* = 7.20 Hz); 3.80 (s, 3H, OCH_3_) 6.44 (d,1H, *J* = 7.80 Hz); 7.01 (d, 2H, *J* = 8.40 Hz); 7.74 (d, 1H, *J* = 15.60 Hz); 7.77 (d,1H, *J* = 15.60 Hz); 7.84 (d, 2H, *J* = 8.40 Hz); 8.02 (d, 2H, *J* = 8.40 Hz); 8.26 (d, 1H, *J* = 8.40 Hz); 10.52­(s,1H, NH). ^**13**^ **C NMR (150 MHz) (DMSO-*d***_**6**_ **/TMS):** δ 24.6, 25.4, 31.1, 48.6, 55.9, 114.9, 119.9, 127.6, 128.0, 129.4, 131.5, 141.7, 143.9, 145.6, 150.8, 162.1, 188.9. **Elemental Analysis:** C_23_H_26_N_2_O_5_S (442.53 g/mol) Anal. Calcd (%): C, 62.43; H, 5.92; N, 6.33; S, 7.24. Found (%): C, 62.52; H, 6.00; N, 6.37; S, 7.30.

#### 2.2.5 (*E*)-*N*-(Cyclohexylcarbamoyl)-4-(3-(4-Iodophenyl)­acryloyl)­benzenesulfonamide (**7**)

**Yield:** 73%. Yellow solid. **Mp:** 200–201. ^**1**^ **H NMR (600 MHz) (DMSO-*d***_**6**_ **/TMS):** δ 1.06–1.11 (m, 3H); 1.15–1.20 (m, 2H); 1.45 (d, 1H, *J* = 13.20 Hz); 1.56 (d, 2H, *J* = 13.20 Hz); 1.62 (d, 2H, *J* = 9.00 Hz); 3.25 (d, 1H, *J* = 3.60 Hz); 6.47 (d, 1H, *J* = 7.80 Hz, NH); 7.68 (d, 2H, *J* = 7.80 Hz); 7.71 (d, 1H, *J* = 16.20 Hz); 7.82 (d, 2H, *J* = 8.40 Hz); 7.94 (d, 1H, *J* = 15.60 Hz); 8.03 (d, 2H, *J* = 7.80 Hz); 8.28 (d, 2H, *J* = 8.40 Hz); 10.55 (s, 1H, NH).^**13**^ **C NMR (150 MHz) (DMSO-*d***_**6**_ **/TMS):** δ 189.0 (C=O); 24.6, 25.4, 32.7, 48.6, 98.7, 123.0, 128.1, 129.5, 131.1, 134.5, 137.6, 138.3, 141.3, 144.5, 150.8. **Elemental Analysis:** C_22_H_23_IN_2_O_4_S (538.40 g/mol) Anal. Calcd (%): C, 49.08; H, 4.31; N, 5.20; S, 5.95. Found (%): C, 49.21; H, 4.40; N, 5.25; S, 5.99.

#### 2.2.6 (*E*)-4-(3-(4-Bromophenyl)­acryloyl)-*N*-(cyclohexylcarbamoyl)­benzenesulfonamide (**12**)

**Yield:** 68%. Yellow solid. **Mp:** 218–219 °C. **FTIR (ν**_**max**_ **, cm** ^**–1**^ **):** 3302, 3243 (N–H); 2932, 2855 (C–H); 1666, 1677 (C=O); 1489, 1335/1162 (asymmetric/symmetric SO_2_). ^**1**^ **H NMR (600 MHz) (DMSO-*d***_**6**_ **/TMS):** δ 1.06–1.11 (m, 3H); 1.15–1.20 (m, 2H); 1.45 (d, 1H, *J* = 7.80 Hz); 1.55 (d, 2H, *J* = 9.00 Hz); 1.62 (d, 2H, *J* = 9.00 Hz); 3.25 (d, 1H, *J* = 3.60 Hz); 6.44 (d, 1H, *J* = 7.80 Hz, NH); 7.66 (d, 2H, *J* = 9.00 Hz); 7.74 (d, 1H, *J* = 15.60 Hz), 7.85 (d, 2H, *J* = 8.40 Hz); 7.94 (d, 1H, *J* = 16.20 Hz); 8.03 (d, 2H, *J* = 8.40 Hz); 8.29 (d, 2H, *J* = 9.00 Hz); 10.52 (s, 1H, NH). **Elemental Analysis:** C_22_H_23_BrN_2_O_4_S (491.40 g/mol) Anal. Calcd (%): C, 53.77; H, 4.72; N, 5.70; S, 6.52. Found (%): C, 53.81; H, 4.79; N, 5.80; S, 6.57.

#### 2.2.7 (*E*)-*N*-(Cyclohexylcarbamoyl)-4-(3-(4-(Methylthio)­phenyl)­acryloyl)­benzenesulfonamide (15)

**Yield:** 87%. Yellow solid. **Mp:** 211–212 °C. **FTIR (ν**_**max**_ **, cm** ^**–1**^ **):** 3321 (N–H); 3171, 3101, 2933 (C–H); 1647 (C=O); 1356/1162 (asymmetric/symmetric SO_2_). ^**1**^ **H NMR (600 MHz) (DMSO-*d***_**6**_ **/TMS):** δ 1.06–1.11 (m, 3H); 1.15–1.21 (m, 2H); 1.45 (d, 1H, *J* = 13.20 Hz); 1.56 (d, 2H, *J* = 13.20 Hz); 1.63 (d, 2H, *J* = 12.60 Hz); 3.25 (d, 1H, *J* = 7.80 Hz); 6.43 (d,1H, *J* = 7.8 Hz); 7.30 (d, 2H, *J* = 8.40 Hz); 7.74 (d, 1H, *J* = 15.6 Hz); 7.81 (d, 2H, *J* = 8.40 Hz); 7.86 (d, 1H, *J* = 15.60 Hz); 8.03 (d, 2H, *J* = 8.40 Hz); 8.27 (d, 2H, *J* = 8.40 Hz); 10.52­(s,1H, NH). **Elemental Analysis:** C_23_H_26_N_2_O_4_S_2_ (458.59 g/mol) Anal. Calcd (%): C, 60.24; H, 5.71; N, 6.11; S, 13.98. Found (%): C, 60.35; H, 5.80; N, 6.17; S, 14.02.

#### 2.2.8 (E)-*N*-(Cyclohexylcarbamoyl)-4-(3-(2,4-Dimethylphenyl)­acryloyl)­benzenesulfonamide (**22**)

**Yield:** 84%. Yellow solid. **Mp:** 186–188 °C. **FTIR (ν**_**max**_ **, cm** ^**–1**^ **):** 3339 (N–H); 3067, 2933 (C–H); 1651 (C=O); 1338/1162 (asymmetric/symmetric SO_2_). ^**1**^ **H NMR (600 MHz) (DMSO-*d***_**6**_ **/TMS):** δ 1.08–1.16 (m, 3H); 1.19–1.48 (m, 2H); 1.55 (d, 1H, *J* = 10.00 Hz); 1.61–1.71 (m, 4H); 2.32 (s, 3H); 2.95 (s, 1H); 6.33 (d, 1H, *J* = 7.60 Hz); 7.13 (d, 2H, *J* = 8.40 Hz), 7.80 (d, 1H, *J* = 21.60 Hz); 7.94 (d, 1H, *J* = 8.40 Hz); 7.99–8.04 (m, 3H); 8.26–8.32 (m, 3H). ^**13**^ **C NMR (150 MHz) (DMSO-*d***_**6**_ **/TMS):** δ 19.7, 21.5, 24.8, 25.54, 32.9, 48.6, 122.0, 126.5, 127.5, 127.6, 127.9, 129.6, 130.8, 132.0, 136.9, 138.8, 141.2, 142.4, 172.4, 189.1. **Elemental Analysis:** C_24_H_28_N_2_O_4_S (440.56 g/mol) Anal. Calcd (%): C, 65.43; H, 6.41; N, 6.36; S, 7.28. Found (%): C, 65.47; H, 6.47; N, 6.41; S, 7.35.

#### 2.2.9 (*E*)-*N*-(Cyclohexylcarbamoyl)-4-(3-(2,6-Dimethylphenyl)­acryloyl)­benzenesulfonamide (**23**)

**Yield:** 72%. White solid. **Mp:** 191–192 °C. **FTIR (ν**_**max**_ **, cm** ^**–1**^ **):** 3347 (N–H); 3078, 2929 (C–H); 1658 (C=O); 1353/1166 (asymmetric/symmetric SO_2_). ^**1**^ **H NMR (600 MHz) (DMSO-*d***_**6**_ **/TMS):** δ 1.06–1.11 (m, 3H); 1.14–1.20 (m, 2H); 1.45 (d, 1H, *J* = 12.60 Hz); 1.56 (d, 2H, *J* = 13.20 Hz); 1.62 (d, 2H, *J* = 8.40 Hz); 3.24 (d, 1H, *J* = 7.80 Hz); 6.45 (d,1H, *J* = 7.80 Hz); 7.11 (d, 2H, *J* = 7.20 Hz); 7.17 (t, 1H, *j*_*1*_ = 8.40, *j*_*2*_ *=* 6.60 Hz); 7.39 (d, 1H, *J* = 16.20 Hz); 7.86 (d, 1H, *J* = 16.20 Hz); 8.02 (d, 2H, *J* = 8.40 Hz); 8.22 (d, 1H, *J* = 8.40 Hz); 10.55 (s ,1H, NH). ^**13**^ **C NMR (150 MHz) (DMSO-*d***_**6**_ **/TMS):** δ 20.4, 21.2, 24.6, 25.4, 32.7, 48.6, 127.4, 127.6, 128.1, 128.2, 128.7, 129.1, 129.1, 129.5, 134.3, 134.8, 137.3, 141.2. **Elemental Analysis:** C_24_H_28_N_2_O_4_S (440.56 g/mol) Anal. Calcd (%): C, 65.43; H, 6.41; N, 6.36; S, 7.28. Found (%): C, 65.49; H, 6.51; N, 6.51; S, 7.41.

#### 2.2.10 (*E*)-*N*-(Cyclohexylcarbamoyl)-4-(3-(2,4,5-Trimethylphenyl)­acryloyl)­benzenesulfonamide (**24**)

**Yield:** 79%. Yellow solid. **Mp:** 213–214 °C. **FTIR (ν**_**max**_ **, cm** ^**–1**^ **):** 3343 (N–H); 3067, 2933 (C–H); 1651 (C=O); 1341/1162 (asymmetric/symmetric SO_2_). ^**1**^ **H NMR (600 MHz) (DMSO-*d***_**6**_ **/TMS):** δ 1.05–1.11 (m, 3H); 1.14–1.20 (m, 2H); 1.44 (d, 1H, *J* = 12.60 Hz); 1.56 (d, 2H, *J* = 9.00 Hz); 1.62 (d, 2H, *J* = 9.00 Hz); 2.20 (3H, CH_3_); 2.35 (3H, CH_3_); 3.26 (d, 1H, *J* = 9.60 Hz); 6.61 (d, 1H, *J* = 8.40 Hz); 7.75 (d, 1H, *J* = 15.00 Hz); 7.79 (s, 1H); 7.96­(d,1H, *J* = 15.00 Hz); 8.02 (s, 2H); 8.26 (d, 2H, *J* = 8.40 Hz); 10.67­(s, 1H, NH). ^**13**^ **C NMR (150 MHz) (DMSO-*d***_**6**_ **/TMS):** δ 19.1, 19.3.19.8, 24.6, 25.5, 32.7, 48.5, 121.6, 126.8, 127.9, 128.2, 129.3, 130.8, 131.5, 132.5, 134.7, 136.3, 140.2, 142.7, 188.9, 193.4. **Elemental Analysis:** C_25_H_30_N_2_O_4_S (454.59 g/mol) Anal. Calcd (%): C, 66.05; H, 6.65; N, 6.16; S, 7.05. Found (%): C, 66.20; H, 6.71; N, 6.21; S, 7.15.

#### 2.2.11 (*E*)-*N*-(Cyclohexylcarbamoyl)-4-(3-(2,4,6-Trimethylphenyl)­acryloyl)­benzenesulfonamide (25)

**Yield:** 61%. White solid. **Mp:** 190–191 °C. **FTIR (ν**_**max**_ **, cm** ^**–1**^ **):** 3343 (N–H); 3056, 2929 (C–H); 1658 (C=O); 1338/1162 (asymmetric/symmetric SO_2_). ^**1**^ **H NMR (600 MHz) (DMSO-*d***_**6**_ **/TMS):** δ 1.06–1.11 (m, 3H); 1.15–1.20 (m, 2H); 1.45 (d, 1H, *J* = 12.60 Hz); 1.55 (d, 2H, *J* = 13.80 Hz); 1.62 (d, 2H, *J* = 9.00 Hz); 2.20 (3H, CH_3_); 3.25­(d, 1H, *J* = 7.20 Hz), 6.43 (d, 1H, *J* = 7.80 Hz); 6.93 (s, 2H); 7.34 (d, 1H, *J* = 15.60 Hz); 7.86 (d, 1H, *J* = 16.20 Hz); 8.02­(d, 1H, *J* = 9.00 Hz); 8.20 (d, 2H, *J* = 8.40 Hz); 10.53 (s, 1H, NH). ^**13**^ **C NMR (150 MHz) (DMSO-*d***_**6**_ **/TMS):** δ 20.3, 21.1, 21.3, 24.6, 25.4, 32.7, 48.6, 127.1, 128.1, 129.5, 129.6, 131.2, 137.6, 138.9, 141.3, 143.6, 144.1, 150.8, 189.2. **Elemental Analysis:** C_25_H_30_N_2_O_4_S (454.59 g/mol) Anal. Calcd (%): C, 66.05; H, 6.65; N, 6.16; S, 7.05. Found (%): C, 66.15; H, 6.69; N, 6.19; S, 7.14.

#### 2.2.12 (*E*)-4-(3-([1.1′-Biphenyl]-4-yl)­acryloyl)-*N*(cyclohexylcarbamoyl)­benzenesulfonamide (**26**)

**Yield:** 60%. Yellow solid. **Mp:** 213–215 °C. **FTIR (ν**_**max**_ **, cm** ^**–1**^ **):** 3345, 3060 (N–H); 2935, 2857 (C–H); 1691, 1653 (C=O); 1346/1164 (asymmetric/symmetric SO_2_). ^**1**^ **H NMR (600 MHz) (DMSO-*d***_**6**_ **/TMS):** δ 1.06–1.11 (m, 3H); 1.12–1.20 (m, 2H); 1.46 (d, 1H, *J* = 12.60 Hz); 1.56 (d, 2H, *J* = 13.20 Hz); 1.63 (d, 2H, *J* = 13.80 Hz); 3.26 (d, 1H, *J* = 7.80 Hz); 6.44 (d, 1H, *J* = 7.80 Hz, NH); 7.39 (t, 1H, *j*_*1*_ = 7.80, *j*_*2*_ = 7.20 Hz); 7.47 (t, 2H, *j*_*1*_ = 7.80, *j*_*2*_ = 7.80 Hz), 7.74 (d, 1H, *J* = 7.20 Hz); 7.77 (d, 2H, *J* = 8.40 Hz); 7.82 (d, 1H, *J* = 15.60 Hz); 7.96 (d, 1H, *J* = 15.60 Hz); 7.98 (d, 2H, *J* = 8.40 Hz); 8.04 (d, 2H, *J* = 8.40 Hz); 8.31 (d, 2H, *J* = 8.40 Hz); 10.52 (s, 1H, NH). ^**13**^ **C NMR (150 MHz) (DMSO-*d***_**6**_ **/TMS):** δ 189.1 C=O); 24.7, 25.4, 32.7, 48.7, 122.3, 126.8, 127.2, 127.6, 128.1, 128.5, 129.5, 130.2, 134.1, 139.6, 141.5, 142.9, 144.1, 145.1, 150.8. **Elemental Analysis:** C_28_H_28_N_2_O_4_S (488.60 g/mol) Anal. Calcd (%): C, 68.83; H, 5.78; N, 5.73; S, 6.56. Found (%): C, 68.87; H, 5.83; N, 5.79; S, 6.61.

#### 2.2.13 (*E*)-*N*-(Cyclohexylcarbamoyl)-4-(3-(Naphthalen-2-yl)­acryloyl)­benzenesulfonamide (**28**)

**Yield:** 79%. White solid. **Mp:** 227–229 °C. **FTIR (ν**_**max**_ **, cm** ^**–1**^ **):** 3317 (N–H); 3179, 2933 (C–H); 1647 (C=O); 1360/1166 (asymmetric/symmetric SO_2_). ^**1**^ **H NMR (600 MHz) (DMSO-*d***_**6**_ **/TMS):** δ 1.06–1.12 (m, 3H); 1.15–1.20 (m, 2H); 1.44 (d, 1H, *J* = 12.60 Hz); 1.56 (d, 2H, *J* = 13.80 Hz); 1.62 (d, 2H, *J* = 9. 00 Hz); 3.26 (d, 1H, *J* = 11.40 Hz); 6.63 (d, 1H, *J* = 7.80 Hz); 7.54–7.58 (m, 2H); 7.92 (d, 1H, *J* = 16.20 Hz); 7.94 (t, 2H, *j*_*1*_ *=* 8.40, *j*_*2*_ *=* 9 Hz); 7.97 (d, 1H, *J* = 9 Hz); 8.03 (d, 1H, *J* = 15.60 Hz); 8.05 (d, 2H, *J* = 8.40 Hz); 8.10 (d, 1H, *J* = 10.80 Hz); 8.32 (d, 2H, *J* = 8.40 Hz); 8.34 (s, 1H, NH); 10.68 (s, 1H, NH). ^**13**^ **C NMR (150 MHz) (DMSO-*d***_**6**_ **/TMS):** δ 24.6, 25.4, 32.7, 48.6, 122.7, 124.8, 127.3, 128.1, 128.2, 128.9, 129.1, 129.5, 131.6, 132.6, 133.4, 134.5, 141.4, 144.2, 145.6, 150.9, 189.1. **Elemental Analysis:** C_26_H_26_N_2_O_4_S (462.56 g/mol) Anal. Calcd (%): C, 67.51; H, 5.67; N, 6.06; S, 6.93. Found (%): C, 67.60; H, 5.70; N, 6.14; S, 6.97. **MS (** *m* **/** *z* **):** 461.2 [M-H]^−^

#### 2.2.14 *N*-(Cyclohexylcarbamoyl)-4-(3-(Naphthalen-1-yl)­acryloyl)­benzenesulfonamide (28A)

**Yield:** 34%. Yellow solid. **Mp:** 197–198 °C. **FTIR (ν**_**max**_ **, cm** ^**–1**^ **):** 3343 (N–H); 3060, 2929 (C–H); 1651 (C=O); 1349/1162 (asymmetric/symmetric SO_2_). ^**1**^ **H NMR (600 MHz) (DMSO-*d***_**6**_ **/TMS):** δ 1.06–1.12 (m, 3H); 1.15–1.21 (m, 2H); 1.45 (d, 1H, *J* = 13.20 Hz); 1.56 (d, 2H, *J* = 13.20 Hz); 1.63 (d, 2H, *J* = 13.20 Hz); 3.26 (d, 1H, *J* = 7.80 Hz); 6.51 (d, 1H, *J* = 7.80 Hz, NH); 7.57–7.65 (m, 3H); 7.98 (d, 1H, *J* = 15.60 Hz); 7.99 (d, 1H, *J* = 8.40 Hz); 8.05 (d, 3H, *J* = 8.40 Hz); 8.2 (d, 1H, *J* = 8.40 Hz); 8.27 (d, 1H, *J* = 8.40 Hz); 8.34 (d, 2H, *J* = 8.40); 8.58 (d, 1H, *J* = 15.60 Hz); 10.59 (s, 1H, NH). ^**13**^ **C NMR (150 MHz) (DMSO-*d***_**6**_ **/TMS):** δ 24.6, 25.4, 32.7, 48.6, 123.4, 124.8, 126.1, 126.4, 126.8, 127.8, 127.9, 128.1, 129.3, 129.6, 131.5, 131.7, 133.8, 141.4, 141.4, 144.2, 150.8, 189.0. **Elemental Analysis:** C_26_H_26_N_2_O_4_S (462.56 g/mol) Anal. Calcd (%): C, 67.51; H, 5.67; N, 6.06; S, 6.93. Found (%): C, 67.58; H, 5.72; N, 6.16; S, 6.99. **MS (** *m* **/** *z* **):** 461.2 [M-H]^−^.

### 2.3 General Synthesis Method of *N*-(Cyclohexylcarbamoyl)-4-(5-(Substituted)-4,5-Dihydro-1*H*-Pyrazol-3-yl)­benzenesulfonamide Pyrazole Compounds

(*E*)-*N*-(Cyclohexylcarbamoyl)-4-(3-(substituted)­acryloyl)­benzenesulfonamide chalcone (0.01 mol) and hydrazine monohydrate (0.01 mol) were reacted in methanol in the presence of acetic acid (1 mL) under microwave irradiation (700 W, 70 °C) for 45 min. After completion, the reaction mixture was poured into ice-cold water to precipitate the product. The solid was filtered, washed with water, and purified by recrystallization.^32^

#### 2.3.1 *N*-(Cyclohexylcarbamoyl)-4-(5-(*p*-Tolyl)-4,5-Dihydro-1H-Pyrazol-3-yl)­benzenesulfonamide (**44**)

**Yield:** 18%. White solid. **Mp:** 153–155. ^**1**^ **H NMR (600 MHz) (DMSO-*d***_**6**_ **/TMS):** δ 1.04–1.10 (m, 3H, H); 1.14–1.21 (m, 2H); 1.45 (d, 1H, *J* = 13.20 Hz); 1.55 (d, 2H, *J* = 13.20 Hz); 1.62 (d, 2H, *J* = 8.40 Hz); 2.35 (s, 3H, CH_3_), 2.80–2.84 (dd, 1H, *j*_1_ = 10.20, *j*_*2*_ = 10.20 Hz); 3.24 (d, 1H, *J* = 7.80 Hz); 3.40–3.44 (dd, 1H, *j*_1_ = 10.80, *j*_*2*_ = 10.80 Hz); 4.82–4.86 (m, 1H); 6.25 (d, 1H, *J* = 5.40 Hz); 7.12 (d, 2H, *J* = 7.80 Hz); 7.22 (d, 2H, *J* = 8.40 Hz); 7.72 (t, 2H, *j*_*1*_ = 8.40, *j*_*2*_ = 6.00 Hz); 7.81 (d, 2H, *J* = 8.40 Hz); 7.89 (s, 1H). ^**13**^ **C NMR (150 MHz) (DMSO-*d***_**6**_ **/TMS):** δ 21.1, 24.7, 25.5, 40.5, 32.8, 48.5, 64.1, 125.5, 125.7, 126.9, 126.9, 127.9, 129.4, 129.9, 136.8, 140.2, 147.0. **Elemental Analysis:** C_26_H_28_N_4_O_3_S (476.60 g/mol) Anal. Calcd (%): C, 65.52; H, 5.92; N, 11.76; S, 6.73. Found (%): C, 65.60; H, 5.97; N, 11.81; S, 6.79.

#### 2.3.2 *N*-(Cyclohexylcarbamoyl)-4-(5-(4-Fluorophenyl)-4,5-Dihydro-1H-Pyrazol-3-yl)­benzenesulfonamide (**45**)

**Yield:** 12%. Yellow solid. **Mp:** 200–201 °C. **FTIR (ν**_**max**_ **, cm** ^**–1**^ **):** 3300 (N–H); 2932, 2956 (C–H); 1682 (C=O); 1509 (C=O); 1335/1159 (asymmetric/symmetric SO_2_). ^**1**^ **H NMR (600 MHz) (DMSO-*d***_**6**_ **/TMS):** δ 1.04–1.10 (m, 3H); 1.14–1.23 (m, 2H); 1.45 (d, 1H, *J* = 12.60 Hz); 1.55 (d, 2H, *J* = 9.00 Hz); 1.61 (d, 2H, *J* = 16.20 Hz); 2.83 (t, 1H, *j*_1_ = 10.80, *j*_*2*_ = 16.20 Hz); 3.42–3.47 (dd, 1H, *j*_1_ = 11.40, *j*_*2*_ = 10.80 Hz); 4.88 (t, 1H, *j*_1_ = 7.80, *j*_*2*_ = 13.80 Hz); 6.14 (s, 1H); 7.14 (t, 2H, *j*_*1*_ = 9.00, *j*_*2*_ = 9.00 Hz); 7.32 (s, 1H); 7.37–7.40 (dd, 2H, *j*_1_ = 5.40, *j*_*2*_ = 6.00 Hz); 7.69 (d, 2H, *J* = 8.40 Hz); 7.78 (d, 2H, *J* = 8.40 Hz); 7.89 (s, 1H). ^**13**^ **C NMR (150 MHz) (DMSO-*d***_**6**_ **/TMS):** δ 24.2, 25.5, 30.8, 40.5, 49.8, 63.6, 114.3, 115.5, 125.6, 126.4, 127.8, 128.1, 129.0, 139.4, 143.4, 161.0. **Elemental Analysis:** C_22_H_25_FN_4_O_3_S (444.53 g/mol) Anal. Calcd (%): C, 59.44; H, 5.67; N, 12.60; S, 7.21. Found (%): C, 59.60; H, 5.75; N, 12.64; S, 7.25.

#### 2.3.3 4-(5-(4-Chlorophenyl)-4,5-dihydro-1H-pyrazol-3-yl)-*N*-(cyclohexylcarbamoyl)­benzenesulfonamide (**46**)

**Yield:** 14%. Yellow solid. **Mp:** 181–183 °C. **FTIR (ν**_**max**_ **, cm** ^**–1**^ **):** 3336 (N–H); 2932, 2856 (C–H); 1682 (C=O); 1531 (C=N); 1335/1159 (asymmetric/symmetric SO_2_). ^**1**^ **H NMR (600 MHz) (DMSO-*d***_**6**_ **/TMS):** δ 1.04–1.08 (m, 3H); 1.14–1.22 (m, 2H); 1.45 (d, 1H, *J* = 12.60 Hz); 1.55 (d, 2H, *J* = 12.60 Hz); 1.64 (d, 2H, *J* = 4 Hz); 2.81–2.85 (dd, 1H, *j*_1_ = 10.80, *j*_*2*_ = 4.20 Hz); 2.91 (d, 1H, *J* = 4.20 Hz); 3.48–3.44 (dd, 1H, *j*_1_ = 11.40, *j*_*2*_ = 11.40 Hz); 4.88 (t, 1H, *j*_1_ = 10.20, *j*_*2*_ = 10.80 Hz); 6.19 (s, 1H); 7.37 (d, 4H, *J* = 3.00 Hz); 7.70 (d, 2H, *J* = 8.40 Hz); 7.79 (d, 2H, *J* = 10.20 Hz); 7.92 (s, 1H). ^**13**^ **C NMR (150 MHz) (DMSO-*d***_**6**_ **/TMS):** δ 24.2, 25.5, 31.0, 40.5, 49.8, 63.6, 125.9, 126.4, 127.3, 127.8, 128.9, 132.2, 136.7, 142.3, 143.4, 147.5. **Elemental Analysis:** C_22_H_25_ClN_4_O_3_S (460.98 g/mol) Anal. Calcd (%): C, 57.32; H, 5.47; N, 12.15; S, 6.95. Found (%): C, 57.40; H, 5.51; N, 12.20; S, 7.0.

#### 2.3.4 *N*-(Cyclohexylcarbamoyl)-4-(5-(4-Methoxyphenyl)-4,5-Dihydro-1H-Pyrazol-3-yl)­benzenesulfonamide (**47**)

**Yield:** 14%. White solid. **Mp:** 182–184 °C. **FTIR (ν**_**max**_ **, cm** ^**–1**^ **):** 3339 (N–H); 3067, 2929 (C–H); 1681 (C=O); 1338/1159 (asymmetric/symmetric SO_2_). ^**1**^ **H NMR (400 MHz) (DMSO-*d***_**6**_ **/TMS):** δ 1.06–1.14 (m, 3H); 1.17–1.47 (m, 2H); 1.51–1.64 (m,5H); 2.82–2.89 (dd,1H, *j*_*1*_ *=* 10.00 Hz, *j*_*2*_ *=* 10.80 Hz); 3.32 (d, 1H, *J* = 11 Hz); 3.40–3.47 (dd,1H, *j*_*1*_ *=* 10.40 Hz, *j*_*2*_ *=* 10.00 Hz); 3.74 (s, 3H, OCH_3_); 4.87 (t, 1H, 1H, *j*_*1*_ *=* 11.20, *j*_*2*_ *=* 10.00 Hz); 6.32 (d,1H, *J* = 7.60 Hz); 6.91 (d, 2H, *J* = 6.40 Hz); 7.29 (d, 2H, *J* = 8.80 Hz); 7.76 (d, 2H, *J* = 7.60 Hz); 7.84­(d, 2H, *J* = 8.40 Hz); 7.91 (s, 1H, NH). ^**13**^ **C NMR (150 MHz) (DMSO-*d***_**6**_ **/TMS):** 24.7, 25.4, 32.7, 40.9, 48.6, 56.1, 63.9, 94.2, 114.3, 125.7, 127.1, 128.2, 130.6, 138.9, 150.9, 159.0, 164.1. **Elemental Analysis:** C_23_H_28_N_4_O_4_S (456.56 g/mol) Anal. Calcd (%): C, 60.61; H, 6.18; N, 12.27; S, 7.02. Found (%): C, 60.65; H, 6.25; N, 12.30; S, 7.08. **MS (** *m* **/** *z* **):** 455.0 [M-H]^−^.

#### 2.3.5 *N-*(Cyclohexylcarbamoyl)-4-(5-(4-Iodophenyl)-4,5-Dihydro-1H-Pyrazol-3-yl)­benzenesulfonamide (**49**)

**Yield:** 37%. White solid. **Mp:** 185–187 °C. **FTIR (ν**_**max**_ **, cm** ^**–1**^ **):** 3261 (N–H); 2925 (C–H); 1684 (C=O); 1331/1155 (asymmetric/symmetric SO_2_). ^**1**^ **H NMR (400 MHz) (DMSO-*d***_**6**_ **/TMS):** δ 1.07–1.15 (m, 3H); 1.17–1.26 (m, 2H); 1.49 (d, 1H, *J* = 11.60 Hz); 1.59 (d, 2H, *J* = 12.80 Hz); 1.65 (d, 2H, *J* = 16.40 Hz); 2.84–2.91 (dd, 1H, *j*_*1*_ *=* 10.40 Hz, *j*_*2*_ *=* 10.40 Hz, CH_2_); 3.27 (d, 2H, *J* = 18.00 Hz); 3.46–3.53 (dd, 1H, *j*_*1*_ *=* 11.20 Hz, *j*_*2*_ *=* 11.20 Hz); 4.90 (t, 1H, *j*_*1*_ *=* 10.40 Hz, *j*_*2*_ *=* 13.20 Hz); 6.36 (d,1H, *J* = 7.60 Hz); 7.19 (d, 2H, *J* = 8.40 Hz); 7.72 (d,2H, *J* = 8.00 Hz); 7.78 (d, 2H, *J* = 8.40 Hz); 7.87 (d, 2H, *J* = 8.40 Hz); 8.04 (s,1H, NH); 10.38 (s, 1H, NH). ^**13**^ **C NMR (150 MHz) (DMSO-*d***_**6**_ **/TMS):** 24.7, 25.5, 32.7, 40.4, 48.6, 63.8, 125.9, 128.1, 128.9, 129.5, 137.7, 137.9, 143.2, 147.1, 150.9, 165.3. **Elemental Analysis:** C_22_H_25_lN_4_O_3_S (552.43 g/mol) Anal. Calcd (%): C, 47, 83; H, 4.56; N, 10.14; S, 5.80. Found (%): C, 47.90; H, 4.59; N, 10.20; S, 5.84. **MS (** *m* **/** *z* **):** 551.1 [M-H]^−^.

#### 2.3.6 4-(5-(4-Bromophenyl)-4,5-dihydro-1H-pyrazol-3-yl)-*N*-(cyclohexylcarbamoyl)­benzenesulfonamide (**54**)

**Yield**: 40%. White solid. **Mp:** 194–197 °C. ^**1**^ **H NMR (600 MHz) (DMSO-*d***_**6**_ **/TMS):** δ 1.04–1.10 (m, 3H); 1.15–1.21 (m, 2H); 1.45 (d, 1H, *J* = 9.00 Hz); 1.55 (d, 2H, *J* = 9.60 Hz); 1.61 (d, 2H, *J* = 13.20 Hz); 2.82–2.86 (dd, 1H, *j*_1_ = 10.20, *j*_*2*_ = 10.80 Hz); 3.25 (d, 1H, *J* = 7.80 Hz); 3.49–3.44 (dd, 1H, *j*_1_ = 10.80, *j*_*2*_ = 10.80 Hz); 4.88 (t, 1H, *j*_1_ = 13.80, *j*_*2*_ = 10.20 Hz); 6.25 (d, 1H, *J* = 7.80 Hz); 7.31 (d, 2H, *J* = 8.40 Hz); 7.52 (d, 2H, *J* = 8.40 Hz); 7.72 (d, 2H, *J* = 7.80 Hz); 7.81 (d, 2H, *J* = 7.80 Hz); 7.91 (s, 1H); 7.97 (s, 1H). ^**13**^ **C NMR (150 MHz) (DMSO-*d***_**6**_ **/TMS):** 24.2, 25.5, 32.8, 40.5, 48.5, 63.7, 120.6, 125.8, 128.0, 129.3, 131.4, 131.8, 135.1, 142.8, 154.2, 166.3. **Elemental Analysis:** C_22_H_25_BrN_4_O_3_S (505.43 g/mol) Anal. Calcd (%): C, 52.28; H, 4.99; N, 11.09; S, 6.34. Found (%): C, 52.31; H, 5.01; N, 11.15; S, 6.37.

#### 2.3.7 *N*-(Cyclohexylcarbamoyl)-4-(5-(4-(Methylthio)­phenyl)-4,5-Dihydro-1H-Pyrazol-yl) Benzenesulfonamide (**57**)

**Yield:** 45%. White solid. **Mp:** 177–179. **FTIR (ν**_**max**_ **, cm** ^**–1**^ **):** 3328, 3246 (N–H); 2929 (C–H); 1688 (C = O); 1330/1158 (asymmetric/symmetric SO_2_). ^**1**^ **H NMR (600 MHz) (DMSO-*d***_**6**_ **/TMS):** δ 1.09–1.17 (m, 3H); 1.19–1.28 (m, 2H); 1.50 (d, 1H, *J* = 12.00); 1.59 (d, 2H, *J* = 13.20); 1.67 (d, 2H, *J* = 13.20); 2.51­(s,1H); 2.85–2.92 (dd, 1H, *j*_*1*_ *=* 10, *j*_*2*_ *=* 10.80); 3.44–3.51 (dd, 1H, *j*_*1*_ *=* 11.20, *j*_*2*_ *=* 10.80); 4.91 (t, 1H, 1H, *j*_*1*_ *=* 10.80, *j*_*2*_ *=* 10.80); 6.32 (d, 1H, *J* = 8.40); 7.26 (d, 2H, *J* = 8.40); 7.32 (d, 2H, *J* = 8.4 0); 7.78 (d, 2H, *J* = 8.40); 7.87 (d, 2H, *J* = 8.80); 7.95 (s, 1H, NH); 10.15 (s, 1H, NH). **Elemental Analysis:** C_23_H_28_BrN_4_O_3_S_2_ (472.62 g/mol) Anal. Calcd (%): C, 58.45; H, 5.97; N, 11.85; S, 13.57. Found (%): C, 58.48; H, 6.00; N, 11.91; S, 13.60. **MS (** *m* **/** *z* **):** 473 [M + H]^+^.

#### 2.3.8 *N*-(Cyclohexylcarbamoyl)-4-(5-(2,4-Dimethylphenyl)-4,5-Dihydro-1H-Pyrazol-3-yl) Benzenesulfonamide (**64**)

**Yield:** 78%. White solid. **Mp:** 220–224 °C. **FTIR (ν**_**max**_ **, cm** ^**–1**^ **):** 3350, 3294, 3224 (N–H); 2922, 2851 (C–H); 1681 (C=O); 1334/1159 (asymmetric/symmetric SO_2_). ^**1**^ **H NMR (400 MHz) (DMSO-*d***_**6**_ **/TMS):** δ 1.06–1.15 (m, 3H); 1.17–1.23 (m, 2H); 1.49 (d, 1H, *J* = 11.60 Hz); 1.59 (d, 2H, *J* = 12.00 Hz); 1.65 (d, 2H, *J* = 14.80 Hz); 2.24 (s,3H,CH_3_); 2.30 (s,3H, CH_3_); 2.69–2.76 (dd, 1H, *j*_*1*_ *=* 10.00 Hz, *j*_*2*_ *=* 10.00 Hz); 3.28 (d, 1H, *J* = 7.20 Hz); 3.48–3.55 (dd, 1H, *j*_*1*_ *=* 11.20 Hz, *j*_*2*_ *=* 11.20 Hz); 5.05 (t, 1H, *j*_*1*_ *=* 10.40 Hz, *j*_*2*_ *=* 10.40 Hz); 6.37 (d, 1H, *J* = 8.00 Hz); 6.98 (d, 1H, *J* = 7.60 Hz); 7.01 (s, 1H); 7.26 (d, 1H, *J* = 7.60 Hz); 7.77 (d, 2H, *J* = 8.40 Hz); 7.86 (d, 2H, *J* = 8.40 Hz); 7.92 (s, 1H); 10.24 (s, 1H). ^**13**^ **C NMR (150 MHz) (DMSO-*d***_**6**_ **/TMS):** 19.4, 21.0, 24.7, 25.5, 32.8, 40.5, 48.6, 61.1, 125.7, 126.0, 126.9, 127.9, 131.5, 135.2, 136.4, 138.1, 138.2, 139.3, 146.5, 151.2. **Elemental Analysis:** C_24_H_30_N_4_O_3_S (454.59 g/mol). Anal. Calcd (%): C, 63.41; H, 6.65; N, 12.32; S, 7.05. Found (%): C, 63.45; H, 6.70; N, 12.37; S, 7.09. **MS (** *m* **/** *z* **):** 453.2 [M-H]^−^.

#### 2.3.9 *N*-(Cyclohexylcarbamoyl)-4-(5-(2,6-Dimethylphenyl)-4,5-Dihydro-1H-Pyrazol-3-yl) Benzenesulfonamide (65)

**Yield:** 44%. Yellow solid. **Mp:** 179–181 °C. **FTIR (ν**_**max**_ **, cm** ^**–1**^ **):** 3358 (N–H); 3067, 2929 (C–H); 1684 (C=); 1341/1159 (asymmetric/symmetric SO_2_). ^**1**^ **H NMR (400 MHz) (DMSO-*d***_**6**_ **/TMS):** 1.09–1.24 (m, 5H); 1.47–1.67 (m, 5H); 2.32 (s, 1H); 2.92–3.00 (dd, 1H, *j*_*1*_ *=* 13.20 Hz, *j*_*2*_ *=* 13.00 Hz); 3.31 (d, 1H, *J* = 8.80 Hz); 3.42–3.50 (dd, 1H, *j*_*1*_ *=* 12.80 Hz, *j*_*2*_ *=* 13.20 Hz); 5.33 (t, 1H, *j*_*1*_ *=* 12.00 Hz, *j*_*2*_ *=* 12.00 Hz); 6.37 (d, 1H, *J* = 7.20 Hz); 7.15 (t, 1H, *j*_*1*_ *=* 8.00 Hz, *j*_*2*_ *=* 7.60 Hz); 7.78 (d, 2H, *J* = 7.60 Hz); 7.85 (d, 1H, *J* = 8.00 Hz); 7.88 (d, 2H, *J* = 9.20 Hz); 8.01 (d, 1H, *J* = 8.00 Hz); 8.16 (s, 1H); 10.20 (s, 1H). ^**13**^ **C NMR (150 MHz) (DMSO-*d***_**6**_ **/TMS):** 20.7, 24.7, 25.5, 32.8, 40.5, 48.7, 59.6, 125.7, 127.3, 128.1, 129.1, 129.5, 137.3, 137.9, 138.3, 147.5, 151.6. **Elemental Analysis:** C_24_H_30_N_4_O_3_S (454.59 g/mol) Anal. Calcd (%): C, 63.41; H, 6.65; N, 12.32; S, 7.05. Found (%): C, 63.47; H, 6.69; N, 12.36; S, 7.08. **MS (** *m* **/** *z* **):** 453.1 [M-H]^−^.

#### 2.3.10 *N*-(Cyclohexylcarbamoyl)-4-(5-(2,4,5-Trimethylphenyl)-4,5-Dihydro-1H-Pyrazol-3-yl) Benzenesulfonamide (**66**)

**Yield:** 38%. Yellow solid. **Mp:** 188–190 °C. **FTIR (ν**_**max**_ **, cm** ^**–1**^ **):** 3343, 3298, 3224 (N–H); 2922 (C–H); 1681 (C=O); 1334/1159 (asymmetric/symmetric SO_2_). ^**1**^ **H NMR (400 MHz) (DMSO-*d***_**6**_ **/TMS):** δ 1.07–1.14 (m, 3H); 1.17–1.26 (m, 2H); 1.49 (d, 1H, *J* = 13.20 Hz); 1.59 (d, 2H, *J* = 12.80 Hz); 1.65 (d, 2H, *J* = 11.60 Hz); 2.68–2.75 (dd, 1H, *j*_*1*_ *=* 10.00 Hz, *j*_*2*_ *=* 10.40 Hz); 3.28 (d, 1H, *J* = 9.20 Hz); 3.48–3.25 (dd, 1H, *j*_*1*_ *=* 11.20 Hz, *j*_*2*_ *=* 11.20 Hz); 5.03 (t, 1H, *j*_*1*_ *=* 10.80 Hz, *j*_*2*_ *=* 8.40 Hz); 6.36 (d, 1H, *J* = 8.00 Hz); 6.95 (s, 1H); 7.15 (s, 1H); 7.77 (d, 2H, *J* = 8.80 Hz); 7.86 (d, 2H, *J* = 8.40 Hz); 7.8 (s, 1H, NH); 10.21 (s, 1H, NH). ^**13**^ **C NMR (150 MHz) (DMSO-*d***_**6**_ **/TMS):** 18.9, 19.3, 19.6, 24.7, 25.5, 32.7, 40.5, 48.6, 61.2, 125.7, 127.3, 128.0, 132.1, 132.4, 133.8, 134.9, 138.3, 138.4, 139.0, 146.4, 150.9. **Elemental Analysis:** C_25_H_32_N_4_O_3_S (468.62 g/mol) Anal. Calcd (%): C, 64.08; H, 6.88; N, 11.96; S, 6.84. Found (%): C, 64.13; H, 6.92; N, 12.00; S, 6.91. **MS (** *m* **/** *z* **):** 469.2 [M + H]^+^

#### 2.3.11 *N*-(Cyclohexylcarbamoyl)-4-(5-(2,4,6-Trimethylphenyl)-4,5-Dihydro-1H-Pyrazol-3-yl) Benzenesulfonamide (**67**)

**Yield:** 15%. White solid. **Mp:** 210–212 °C. **FTIR (ν**_**max**_ **, cm** ^**–1**^ **):** 3350 (N–H); 2929, 2855 (C–H); 1677 (C=O); 1531 (C=N); 1341/1159 (asymmetric/symmetric SO_2_). ^**1**^ **H NMR (400 MHz) (DMSO-*d***_**6**_ **/TMS):** δ 1.06–1.15 (m, 3H); 1.17–1.26 (m, 2H); 1.49 (d, 1H, *J* = 5.60 Hz); 1.59 (d, 2H, *J* = 14.00 Hz); 1.64 (d, 2H, *J* = 18.40 Hz); 2.21 (s, 3H); 2.28 (s, 6H); 2.89–2.96 (dd,1H, *j*_*1*_ *=* 13.20 Hz, *j*_*2*_ *=* 12.80 Hz); 3.47–3.26 (m, 2H); 5.31 (t, 1H, *j*_*1*_ *=* 15.60 Hz, *j*_*2*_ *=* 13.20 Hz); 6.35 (d, 1H, *J* = 8.00 Hz); 6.82 (s, 2H); 7.75 (d, 2H, *J* = 7.60 Hz); 7.86 (d, 2H, *J* = 8.00 Hz); 7.92 (s, 1H); 10.20 (s, 1H). ^**13**^ **C NMR (150 MHz) (DMSO-*d***_**6**_ **/TMS):** 20.6, 24.7, 25.5, 32.8, 40.5, 48.5, 56.3, 125.6, 128.1, 130.2, 134.7, 135.0, 135.5, 136.9, 140.8, 154.7, 165.3. **Elemental Analysis:** C_25_H_32_N_4_O_3_S (468.62 g/mol) Anal. Calcd (%): C, 64.08; H, 6.88; N, 11.96; S, 6.84. Found (%): C, 64.11; H, 6.90; N, 11.97; S, 6.89. **MS (** *m* **/** *z* **):** 466.9 [M-H]^−^

#### 2.3.12 4-(5-([1.1′-biphenyl]-4-yl)-4,5-dihydro-1H-pyrazol-3-yl)-*N*-(cyclohexylcarbamoyl) Benzenesulfonamide (**68**)

**Yield:** 12%. Yellow solid. **Mp:** 193–195 °C. **FTIR (ν**_**max**_ **, cm** ^**–1**^ **):** 3301, 3450 (N–H); 2928, (C–H); 1525 (C=N); 1683 (C=O); 1333/1160 (asymmetric/symmetric SO_2_). ^**1**^ **H NMR (600 MHz) (DMSO-*d***_**6**_ **/TMS):** δ 1.04–1.11 (m, 3H); 1.15–1.20 (m, 2H), 1.45 (d, 1H, *J* = 12.60 Hz); 1.56 (d, 2H, *J* = 9.00 Hz); 1.62 (d, 2H, *J* = 9.00 Hz); 2.89–2.94 (dd, 1H, *j*_1_ = 10.20, *j*_*2*_ = 10.20 Hz); 3.24 (d, 1H, *J* = 9.00 Hz); 3.51–3.46 (dd, 1H, *j*_1_ = 10.80, *j*_*2*_ = 10.80 Hz); 4.95 (t, 1H, *j*_1_ = 13.20, *j*_*2*_ = 10.80, Hz); 6.28 (d, 1H, *J* = 7.80 Hz); 7.33 (t, 1H, *j*_1_ = 7.80, *j*_*2*_ = 7. Twenty Hz); 7.44–7.42 (dd, 4H, *j*_1_ = 3.60, *j*_*2*_ = 3.16 Hz), 7.62 (d, 4H, *J* = 8.40 Hz); 7.76 (d, 2H, *J* = 8.40 Hz); 7.83 (d, 2H, *J* = 8.40 Hz); 8.00 (s, 1H). ^**13**^ **C NMR (150 MHz) (DMSO-*d***_**6**_ **/TMS):** δ 24.7, 25.5, 32.8, 40.4, 48.5, 64.0, 125.6, 125.7, 126.1, 127.0, 127.3, 127.6, 127.8, 127.9, 129.4, 129.4, 139.7, 140.4, 142.5, 142.5, 147.1. **Elemental Analysis:** C_28_H_30_N_4_O_3_S (502.63 g/mol) Anal. Calcd (%): C, 66.91; H, 6.02; N, 11.15; S, 6.38. Found (%): C, 66.95; H, 6.07; N, 11.20; S, 6.43.

#### 2.3.13 *N*-(Cyclohexylcarbamoyl)-4-(5-(Naphthalen-2-yl)-4,5-Dihydro-1H-Pyrazol-3-yl) Benzenesulfonamide (**70**)

**Yield:** 50%. White solid. **Mp:** 198–200 °C. **FTIR (ν**_**max**_ **, cm** ^**–1**^ **):** 3346, 3053 (N–H stretching band); 2934, 2858 (C–H); 1654, 1607 (C=O); 1342/1159 (asymmetric/symmetric SO_2_). ^**1**^ **H NMR (400 MHz) (DMSO-*d***_**6**_ **/TMS):** δ 1.07–1.15 (m, 3H); 1.17–1.26 (m, 2H); 1.19 (d, 1H, *J* = 12.00 Hz); 1.24 (d, 2H, *J* = 10.80 Hz); 1.49 (d, 2H, *J* = 11.60 Hz); 2.97–3.04 (dd, 1H, *j*_*1*_ *=* 10.40 Hz, *j*_*2*_ *=* 10.80 Hz); 3.33 (d, 1H, *J* = 30.40 Hz); 3.55–3.62 (dd, 1H, *j*_*1*_ *=* 11.80 Hz, *j*_*2*_ *=* 11.60 Hz); 5.11 (t, 1H, *j*_*1*_ *=* 8.40 Hz, *j*_*2*_ *=* 13.60 Hz); 6.37 (d, 1H, *J* = 7.60 Hz); 7.48–7.56 (m, 3H); 7.82 (d, 2H, *J* = 6.80 Hz); 7.88 (d, 3H, *J* = 8.80 Hz); 7.93 (d, 2H, *J* = 7.60 Hz); 7.99 (t, 1H, 1H, *j*_*1*_ *=* 8.80 Hz, *j*_*2*_ *=* 10.80 Hz); 8.14 (s, 1H); 10.10 (s, 1H, NH). ^**13**^ **C NMR (150 MHz) (DMSO-*d***_**6**_ **/TMS):** 24.7, 25.5, 32.8, 40.5, 48.6, 64.5, 125.4, 125.5, 125.9, 126.3, 126.7, 128.0, 128.1, 128.2, 128.7, 132.9, 133.3, 138.1, 139.2, 140.7, 147.1, 150.9. **Elemental Analysis:** C_26_H_28_N_4_O_3_S (476.60 g/mol) Anal. Calcd (%): C, 65.52; H, 5.92; N, 11.76; S, 6.73. Found (%): C, 65.57; H, 5.97; N, 11.80; S, 6.80. **MS (** *m* **/** *z* **):** 475.2 [M-H]^−^.

#### 2.3.14 *N*-(Cyclohexylcarbamoyl)-4-(5-(Naphthalen-1-yl)-4,5-Dihydro-1*H*-Pyrazol-3-yl)­benzenesulfonamide (**70A**)

**Yield:** 55%. White solid. **Mp:** 248–250 °C. **FTIR (ν**_**max**_ **, cm** ^**–1**^ **):** 3354, 3324, 3235 (N–H); 3056, 2922 (C–H); 1681 (C=O); 1326/1155 (asymmetric/symmetric SO_2_). ^1^ **H NMR (400 MHz) (DMSO-*d***_**6**_ **/TMS):** δ 1.06–1.15 (m, 3H); 1.17–1.25 (m, 2H); 1.49 (d, 1H, *J* = 11.20 Hz); 1.58 (d, 2H, *J* = 12.80 Hz); 1.65 (d, 2H, *J* = 13.20 Hz); 2.82–2.89 (dd, 1H, *j*_*1*_ *=* 10.00 Hz, *j*_*2*_ *=* 10.40 Hz); 3.77–3.84 (dd, 1H, *j*_*1*_ *=* 11.60 Hz, *j*_*2*_ *=* 11.60 Hz); 5.68 (t, 1H, *j*_*1*_ *=* 10.40 Hz, *j*_*2*_ *=* 9.20 Hz); 6.37 (d, 1H, *J* = 8.00 Hz); 7.51 (t, 1H, *j*_*1*_ *=* 8.40 Hz, *j*_*2*_ *=* 7.20 Hz); 7.55–7.62 (m, 2H); 7.66 (d, 1H, *J* = 7.20 Hz); 7.80 (d, 2H, *J* = 8.80 Hz); 7.86 (d, 1H, *J* = 8.40 Hz); 7.98 (d, 1H, *J* = 7.20 Hz); 8.15 (d, 2H, *J* = 3.20 Hz); 10.46 (s, 1H, NH). ^**13**^ **C NMR (150 MHz) (DMSO-*d***_**6**_ **/TMS):** 24.7, 25.4, 32.7, 40.4, 48.6, 61.2, 123.5, 124.1, 125.8, 126.0, 126.2, 126.7, 128.0, 129.2, 130.9, 134.0, 138.2, 138.9, 139.2, 146.8, 150.9, 165.9. **Elemental Analysis:** C_26_H_28_N_4_O_3_S (476.60 g/mol) Anal. Calcd (%): C, 65.52; H, 5.92; N, 11.76; S, 6.73. Found (%): C, 65.58; H, 5.96; N, 11.79; S, 6.79. **MS (** *m* **/** *z* **):** 477.1 [M + H]^+^.

### 2.4 Biological Evaluation of Compounds

#### 2.4.1 Cell Culture Conditions and Preparation of Compounds

HEK 293 cells (ATCC, Germany) were cultured in RPMI 1640 medium with 10% FBS, 1% penicillin/streptomycin, and 1% l-glutamine. The incubated at 37 °C in a humid incubator with 5% CO_2_ until they reached 70–80% confluence. The synthesized compounds and the reference drug Glibenclamide were dissolved in DMSO to make stock solutions. Glibenclamide was included as a clinically used antidiabetic reference drug; however, it does not directly inhibit α-glucosidase nor act as a PPAR-γ agonist, and therefore was not used as a mechanism-specific positive control. These solutions were stored at 4 °C until use. The test compounds were serially diluted in serum-free culture medium at concentrations of 100, 50, 25, and 12.5 μM. The highest chemical concentration was determined in the negative control DMSO percentage.^52^

#### 2.4.2 MTT Cell Viability Test

Cells were seeded in 96-well culture plates at 1 × 10^6^ cells/mL and incubated until 80% confluence. Following confluence, the cells were exposed to chemicals at various concentrations for 24 h. After compound exposure incubation, the MTT [3-(4,5-dimethylthiazol-2-yl)-2,5-diphenyl tetrazolium bromide] technique was used. After 24 h of incubation, the culture medium was removed and replaced with serum-free (SF) medium containing 1 mg/mL MTT. The cells were then incubated for 45 min at 37 °C. The MTT solution was removed carefully; to dissolve the formazan crystals, dimethyl sulfoxide (DMSO) was added to the wells. The color changed to purple and was measured at 550 nm with a microplate spectrophotometer. The absorbance readings obtained were normalized to the control group and expressed as a percentage of cell viability. Dose–response curves were generated, and IC_50_ values were determined.^53^

#### 2.4.3 α-Glucosidase Inhibitory Activity Assay

The α-glucosidase inhibition assay was performed as instructed by the manufacturer (Abcam, Cambridge, UK) (ab284520). To prepare the experiment, new compounds (100, 50, 25, and 12.5 μM/mL) were diluted with 100× buffer. To initiate the reaction, 10 μL of each sample was mixed with 30 μL of α-glucosidase enzyme and incubated at room temperature for 60 min. A microplate reader (Thermo Scientific Varioskan) colorimetrically measured 410 nm after 60 min. Acarbose (100 μg/mL) was used as the standard inhibitor control in the test kit. All experiments were performed in triplicate. Enzyme control (EC) values were obtained from wells containing enzyme and substrate without inhibitor, and were used as the reference for calculating percentage inhibition. Formula for relative inhibition percentage:^54^

SlopeofEnzymeControl−SlopeofSample×100

SlopeofEnzymeControl×100

#### 2.4.4 PPAR-γ Ligand Binding (Activation) Assay

A PPAR-γ ligand binding (activation) assay was performed using the manufacturer’s protocol (ab284546) (Abcam, Cambridge, UK). We prepare different concentrations of newly synthesized compounds (100, 50, 25, and 12.5 μM/mL). Each well received 1 μL of sample and 24 μL of reaction mixture comprising recombinant PPAR-γ protein and a fluorescent probe in a black 384-well microplate suitable for fluorescence detection. The kit’s control ligand, WY-14643, was prepared at various concentrations (10,000, 1000, 100, 50, 25, and 12.5 μM) with DMSO as the solvent control. For the control ligand and solvent control groups, 1 μL was added to 24 μL of the reaction mixture to obtain a final volume of 25 μL per well. Fluorescence readings were recorded every 10 min for 60 min at 375/460–470 nm using a Thermo Scientific Varioskan microplate reader, and 30 min values were used for relative fluorescence analysis.^55^

#### 2.4.5 Scratch Wound Healing Assay

The experiment on scratch wound healing was used to determine cell migratory capacity. HEK 293 cells were seeded into 6-well plates at 1.0 × 10^6^ cells/mL per well. The culture was maintained at 37 °C in a humidified environment to achieve 80–90% confluence. For mechanical wounds, a sterile 200 μL pipet tip was used to scrape a line across each well’s center. Once, the wells were gently rinsed with HBSS to remove unattached cells and debris. Compounds **44**, **45** and Glibenclamide (reference drug) were prepared at their IC_50_ concentrations in serum-free medium and exposed to cells. The plate was observed at 0, 24, and 48 h using an inverted microscope. The same scratch locations were photographed at each time point, and wound widths were measured with ImageJ. The original scratch width and the 24- and 48 h widths were compared to estimate the wound closure percentage.^56^

### 2.5 Molecular Dynamics (MD) Simulations and MM/PBSA Free Energy Analyses

Based on previous molecular docking studies and biological activity assessments, compounds **44** and **45** were identified as candidates with significant binding affinity for the peroxisome proliferator-activated receptor gamma (PPAR-γ) enzyme.^30^ Molecular dynamics (MD) simulations were performed to assess the conformational stability and temporal dynamics of complexes formed between these compounds and PPAR-γ. The simulations were performed for 200 ns (ns) using GROMACS 5.0.7.^33^ The protein structure was generated parametrically using the CHARMM36 all-atom force field to simulate atomic–level interactions, while the TIP3P water model was used as the solvent.^34^ The topological parameters for the ligands were produced via the CGenFF server.^35^ Each protein–ligand complex was characterized within a dodecahedral simulation box under periodic boundary conditions, and the system was solvated. To maintain electrical neutrality, an appropriate amount of sodium ions has been incorporated. Following the system preparation, the molecular dynamics simulation was executed in three phases. Initially, energy reduction was performed using a 1000-step gradient-descent method to resolve steric incompatibilities in the initial configuration and optimize the system’s geometry. Thereafter, the system was equilibrated under isochoric–isothermal (*NVT*) and isothermal–isobaric (*NPT*) conditions for 100 ps (ps). A simulation lasting 200 ns was run throughout the production phase, with a time step of 2 fs. The simulation assessed the structural stability of the protein–ligand complexes using root-mean-square deviation (RMSD), root-mean-square fluctuation (RMSF), and radius of gyration (Rg).

The binding energies of the complexes were assessed using the MM/PBSA methodology based on molecular dynamics trajectories. The computations were performed using the g_mmpbsa module in GROMACS.^36^ Calculations of binding free energy (Δ*G* binding) were conducted according to the equations presented below.

$${\Delta G}_{binding}=G_{PL-complex}-G_{P}-G_{L}$$

$$G=E_{MM}+G_{solv}-TS$$

$$E_{MM}=E_{bond}+E_{angle}+E_{dihedral}+E_{improperdihedral}+E_{vdw}+E_{ele}$$

$$G_{solv}=G_{solv-pol}+G_{solv-nonpol,}$$

Δ*G*_binding denotes the binding free energy, determined by subtracting the free energies of the unbound protein and unbound ligand from the free energy of the protein–ligand complex. Total free energy (G) is the sum of molecular mechanical potential energy (E_MM), solvation free energy (G_solv), and the entropic contribution (TS). The E_MM term includes bond, angle, dihedral, and incorrect dihedral components, as well as van der Waals and electrostatic interactions. G_solv, conversely, denotes the aggregate of polar and nonpolar solvation contributions.

### 2.6 Statistical Analysis

All experiments were performed in triplicate (*n* = 3) and repeated independently, using four different concentrations of each synthesized compound for the antidiabetic inhibition assays. IC_50_ values were calculated from dose–response curves using nonlinear regression analysis. Data are expressed as mean ± SEM (standard error of the mean). Statistical significance was determined using one-way ANOVA followed by post hoc analysis, and differences were considered statistically significant at *p* < 0.05.

## 3 Result and Discussion

### 3.1 Chemistry

The structures of 14 chalcone and 14 pyrazole compounds, all original and synthesized for the first time in this study, were confirmed by elemental analysis, FT-IR, 1H NMR, 13C NMR, and LC-MS-MS. In the ^1^H NMR and ^13^C NMR spectra of the synthesized chalcone and pyrazole derivatives, all protons and carbons showed appropriate splitting and chemical shift values, consistent with their chemical structures, when compared with the literature. The synthesized chalcone and pyrazole molecules were obtained in yields of 87–12% (Scheme 1). A decrease in yield was observed during the cyclization of chalcone derivatives into the corresponding pyrazole compounds, which can be attributed to the multistep reaction mechanism, steric effects, and the formation of side products during cyclization. Notably, compounds **44** and **45** bearing F and CH_3_ substituents exhibited the lowest isolated yields. From a structure–activity relationship perspective, the electron-withdrawing property of fluochalcorine may alter the electrophilicity of the chalcone moiety and affect the efficiency of nucleophilic addition and subsequent cyclization, whereas the methyl group, due to its electron-donating and lipophilic character, may influence both reaction kinetics and molecular packing behavior. In addition, both substituents can impact intermolecular interactions, leading to poor crystallization properties and increased loss during purification. Since high purity is essential for subsequent biological evaluation against PPAR-γ and α-glucosidase targets, recrystallization was necessary, further contributing to the reduced yields of these derivatives.

**Scheme 1:**
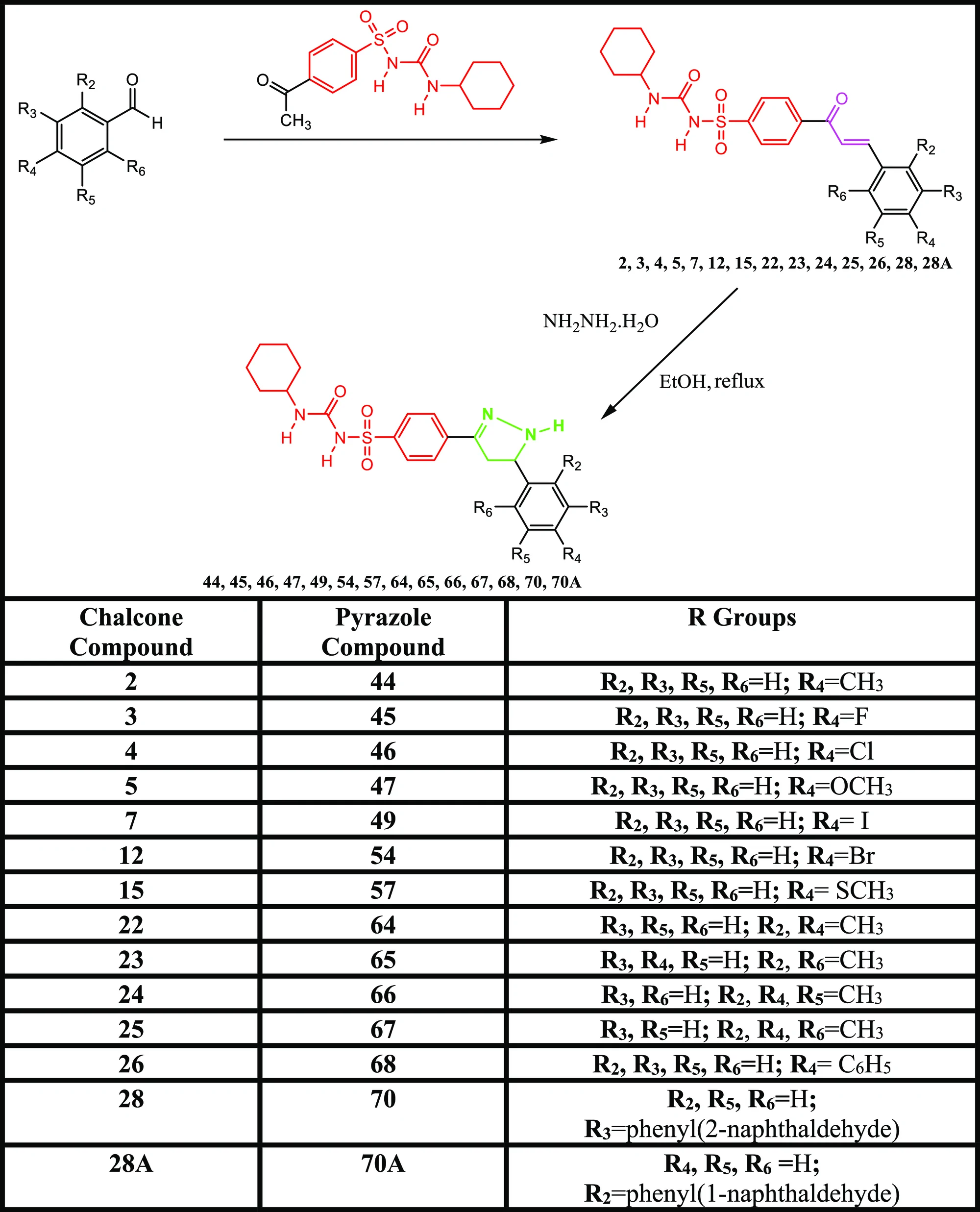
Synthesis Pathway of Chalcone and Pyrazole Compounds

Molecular modeling revealed that compounds **4**, **28**, **28A**, **44**, **45**, **46**, **47**, **49**, **54**, **57**, **64**, **65**, **66**, **67**, **68**, **70**, and **70A** exhibited very high binding affinity to α-glucosidase and PPAR-γ target enzymes. Therefore, compounds **4**, **28**, **28A**, **44**, **45**, **46**, **47**, **49**, **54**, **57**, **64**, **65**, **66**, **67**, **68**, **70**, and **70A**, which have very high binding affinity to α-glucosidase and PPAR-γ target enzymes, were synthesized. In order to synthesize pyrazole compounds, chalcone compounds **2**, **3**, **5**, **7**, **12**, **15**, **22**, **23**, **24**, **25**, and **26** were synthesized as the first step. A total of twenty-eight compounds, including chalcone and pyrazole compounds, were synthesized and their structures characterized.

When the FT-IR spectra of the synthesized chalcone compounds were examined, it was determined that they absorbed N–H bands in the range of 3081–3348 cm^–1^; aromatic C–H stretching bands in the range of 2927–3179 cm^–1^; C=O stretching bands in the range of 1611–1691 cm^–1^; and SO_2_ asymmetric and symmetric stretching bands in the ranges of 1360–1332 and 1161–1168 cm^–1^, respectively. When the ^1^H NMR spectra were examined, it was determined that the H_α_ protons adjacent to the carbonyl, which are the strongest evidence of chalcone formation, were in the range of 7.34–7.98 (*J* = 15.00–16.20) ppm; and the H_β_ protons adjacent to the carbonyl were in the range of 7.77–8.58 (*J* = 15.00–16.20) ppm. Based on these values, it was determined that their structures are in the trans form. When the ^13^C NMR spectra were examined, the C=O carbon atom resonated in the range of 188.89–189.18 ppm.^37–40^

When the FT-IR spectra of the pyrazole compounds were examined, the N–H bands were in the range of 3450–3224 cm^–1^; the aromatic C–H stretching bands were in the range of 3067–2851 cm^–1^. It was determined that C=O stretching bands were in the range of 1684–1654 cm^–1^; SO_2_ asymmetric and symmetric stretching bands were in the ranges of 1330–1342 and 1155–1160 cm^–1^, respectively, indicating absorption. Examination of the ^1^H NMR spectra revealed the absence of peaks for H_α_ and H_β_ protons; two dd-split peaks were observed for the CH_2_ group of the pyrazole ring, and a triplet peak was observed for the CH proton of the pyrazole ring. Based on these data, the formation of a pyrazole ring was confirmed. The CH_2_ protons of the pyrazole ring resonated in the ranges of 2.68–2.75/2.89–2.96 and 3.40–3.44/3.55–3.62 ppm, while the CH proton of the pyrazole ring resonated in the range of 4.82–5.68 ppm. When ^13^C NMR spectra are examined, the carbon atom belonging to C=O resonates in the range of 147.10–165.88 ppm. Peaks for aromatic C were detected in the expected range.^41–44^

### 3.2 *In vitro* Cytotoxic Analysis

#### 3.2.1 Cytotoxicity Evaluation in HEK 293 Cells

The compounds were tested for viability in HEK 293 cells using the MTT assay, and IC_50_ values were determined. Some compounds exhibited no toxic effects on cell viability, while others exhibited significant cytotoxicity. Compounds **44** and **45** had IC_50_ values above 100 μM, indicating no toxic effects in HEK 293 cells at the investigated concentration. Similarly, the reference drug, Glibenclamide, showed an IC_50_ value greater than 100 μM.

The compounds **66**, **70A**, **28A**, and **28** (IC_50_ = 17.00, 17.31, 23.23, and 24.67 μM) showed significant cytotoxicity in HEK 293 cells due to their low IC_50_ values. As a result, the nontoxic compounds **44** and **45** were chosen for the next step of biological testing. Table 1 shows the IC_50_ values for all tested compounds.

**Table 1:**
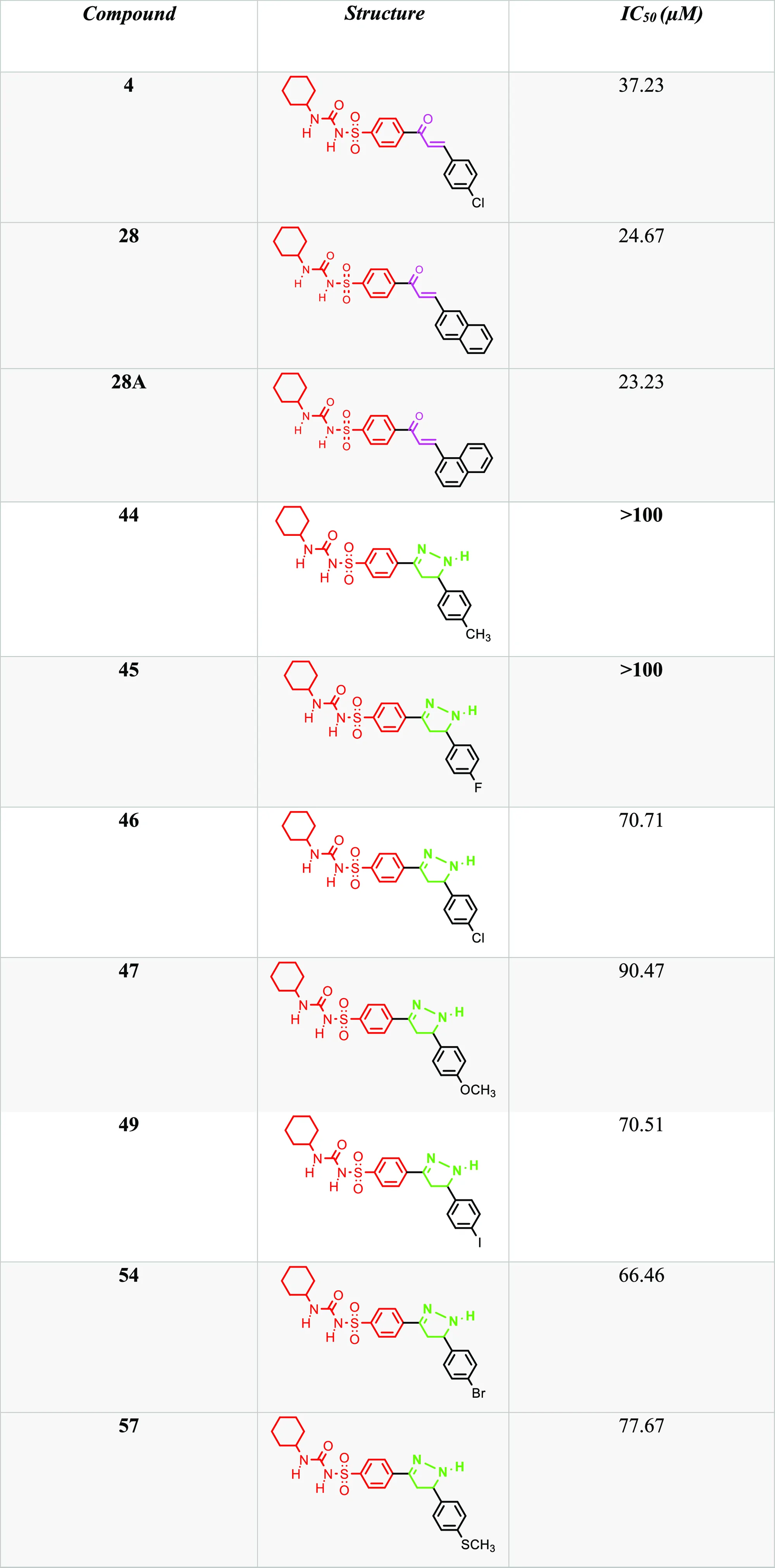

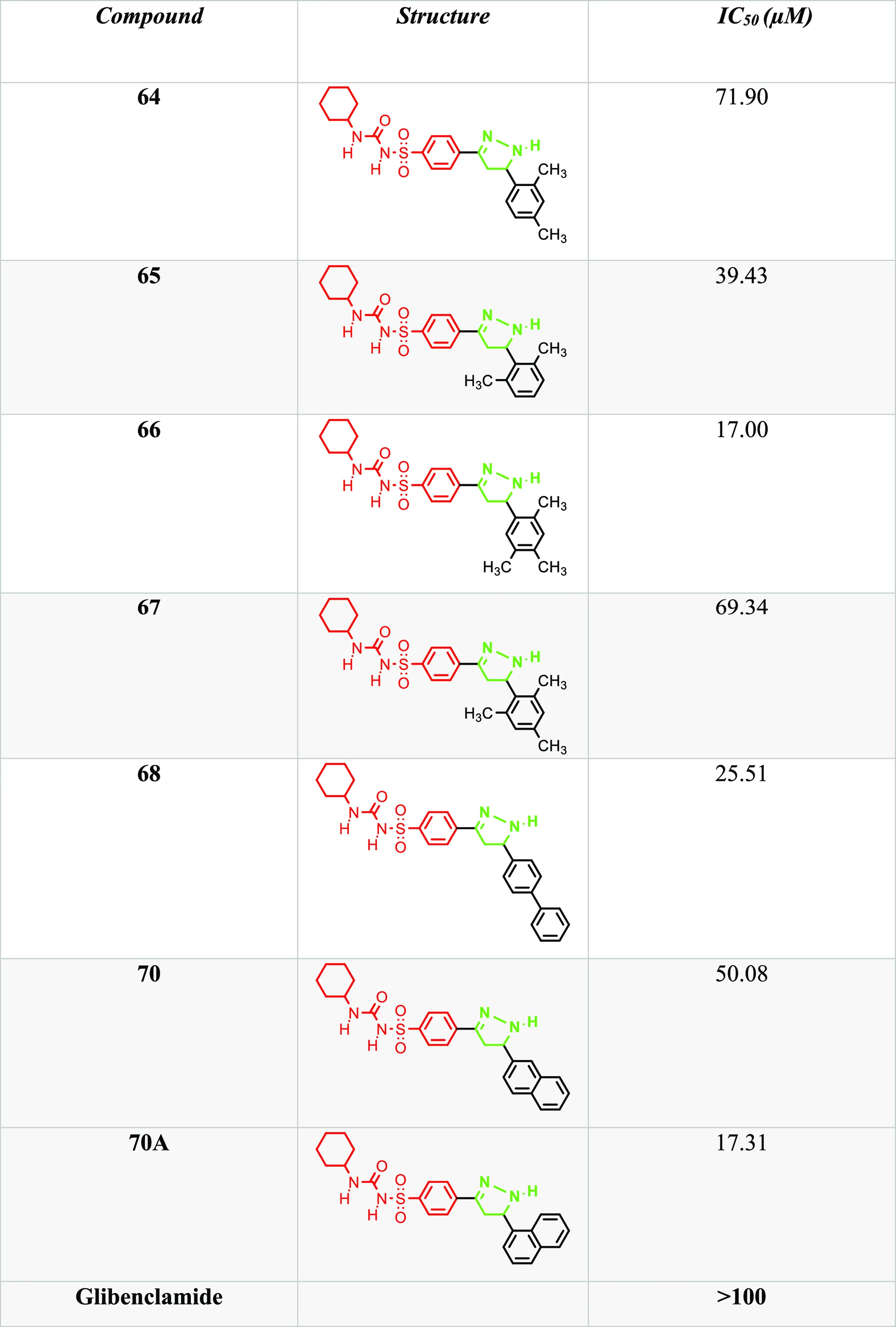
IC_50_ Values of the Compounds in HEK 293 Cells

#### 3.2.2 α-Glucosidase Inhibition Assay Results

Compounds **44** and **45** showed minimal α-glucosidase inhibitory activity across the tested concentrations. In contrast, acarbose exhibited strong and concentration-dependent inhibition, with inhibition values ranging from approximately 80% to over 90%, confirming assay validity (Figure 3.).

**Figure 3:**
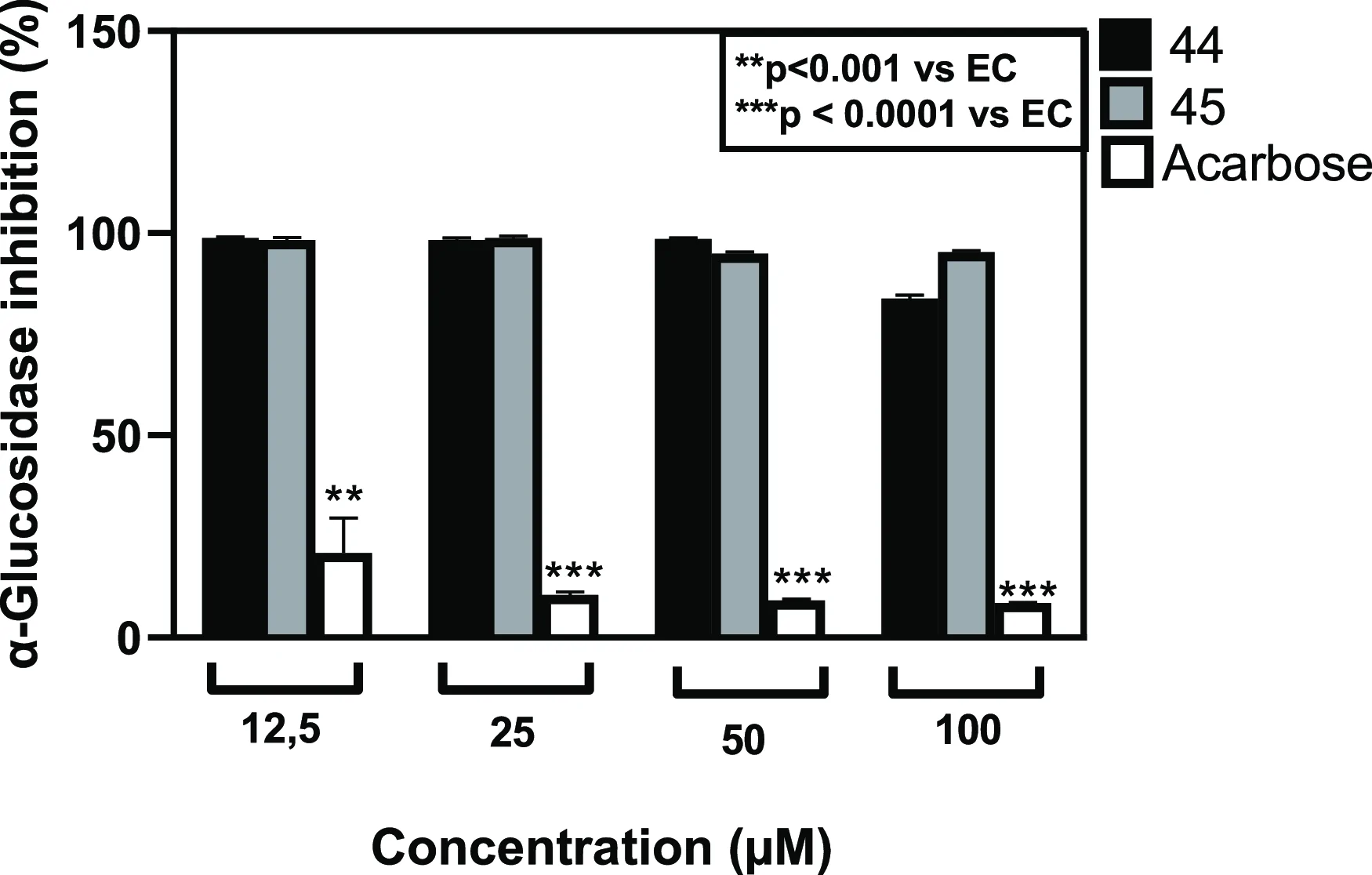
α-Glucosidase inhibitory activity of compounds **44** and **45** compared with acarbose at different concentrations. Data are expressed as mean ± SEM (*n* = 3). Statistical significance was determined using one-way ANOVA. ***p < 0.001 vs enzyme control (EC).

#### 3.2.3 Evaluation of PPAR-γ Ligand Binding Activity

The PPAR-γ ligand binding activation potentials of compounds **44** and **45** were evaluated. Compound **45** exhibited moderate activation potency (EC_50_ = 50.38 μM), whereas compound **44** showed weaker activation (EC_50_ = 98.43 μM). As shown in Figure 4, both compounds induced significant PPAR-γ activation relative to the control group across the tested concentrations. The ligand control WY-14643 exhibited strong activation with an EC_50_ value of 33.55 μM, confirming the validity and proper performance of the assay system. In contrast, Glibenclamide did not exhibit measurable activation within the tested concentration range (EC_50_ > 100 μM).

**Figure 4:**
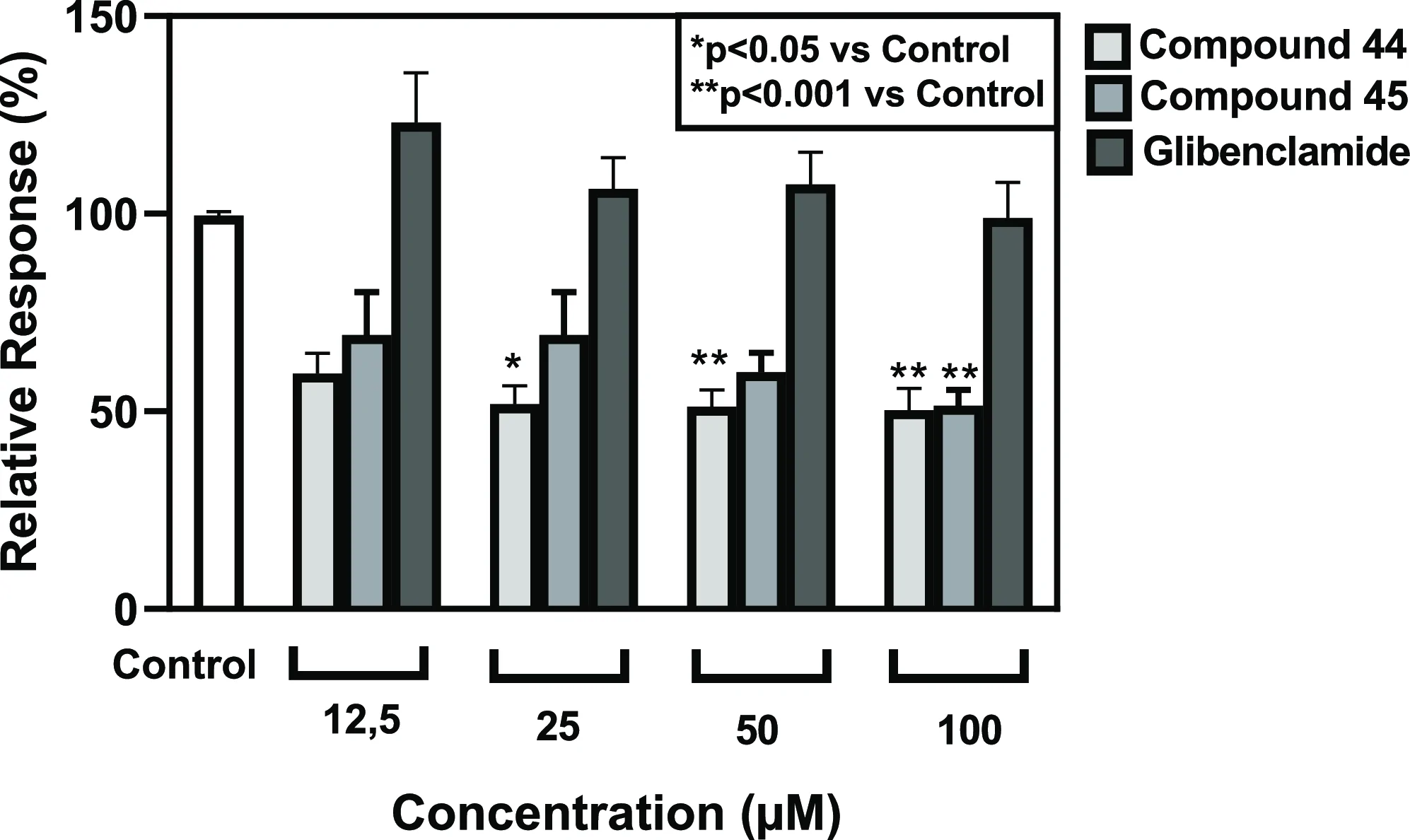
Relative PPAR-γ response of compounds **44** and **45** compared with Glibenclamide. Values are expressed as a percentage of the control group. Data represent mean ± SEM *p < 0.05, **p < 0.001 vs control.

The observation that both fluorine- and methyl-substituted derivatives exhibit high biological activity with low cytotoxicity can be rationalized by their distinct yet favorable physicochemical contributions. Fluorine, as a strong electron-withdrawing substituent, is known to modulate electronic distribution, enhance binding affinity, and improve metabolic stability, thereby contributing to increased biological activity without elevating toxicity. In contrast, the methyl group, a weak electron-donating and lipophilic substituent, can enhance membrane permeability and optimize hydrophobic interactions within the binding pocket while maintaining low intrinsic reactivity. The convergence of these different substituent effects toward a similar biological outcome suggests that both electronic tuning (F) and lipophilicity/steric optimization (CH_3_) can independently lead to compounds with improved activity and reduced off-target toxicity. These findings are consistent with previous reports highlighting the beneficial role of fluorine in drug design as well as the contribution of methyl groups to activity enhancement and selectivity.^45–47^

#### 3.2.4 Scratch Wound Healing Assay Results

Cell migration was measured by calculating the percentage of wound closure at 48 h relative to the initial scratch width. Figure 4 shows that the control group had a wound closure rate of 56.2%. Glibenclamide treatment significantly increased cell migration, resulting in a 72.8% closure rate. Compound **44** considerably improved wound closure (72.3%), indicating a strong stimulatory effect on cell migration compared to the control group. Compound **45**, on the other hand, significantly inhibited cell migration, resulting in a wound closure rate of only 31.3% after 48 h (Figure 5).

**Figure 5:**
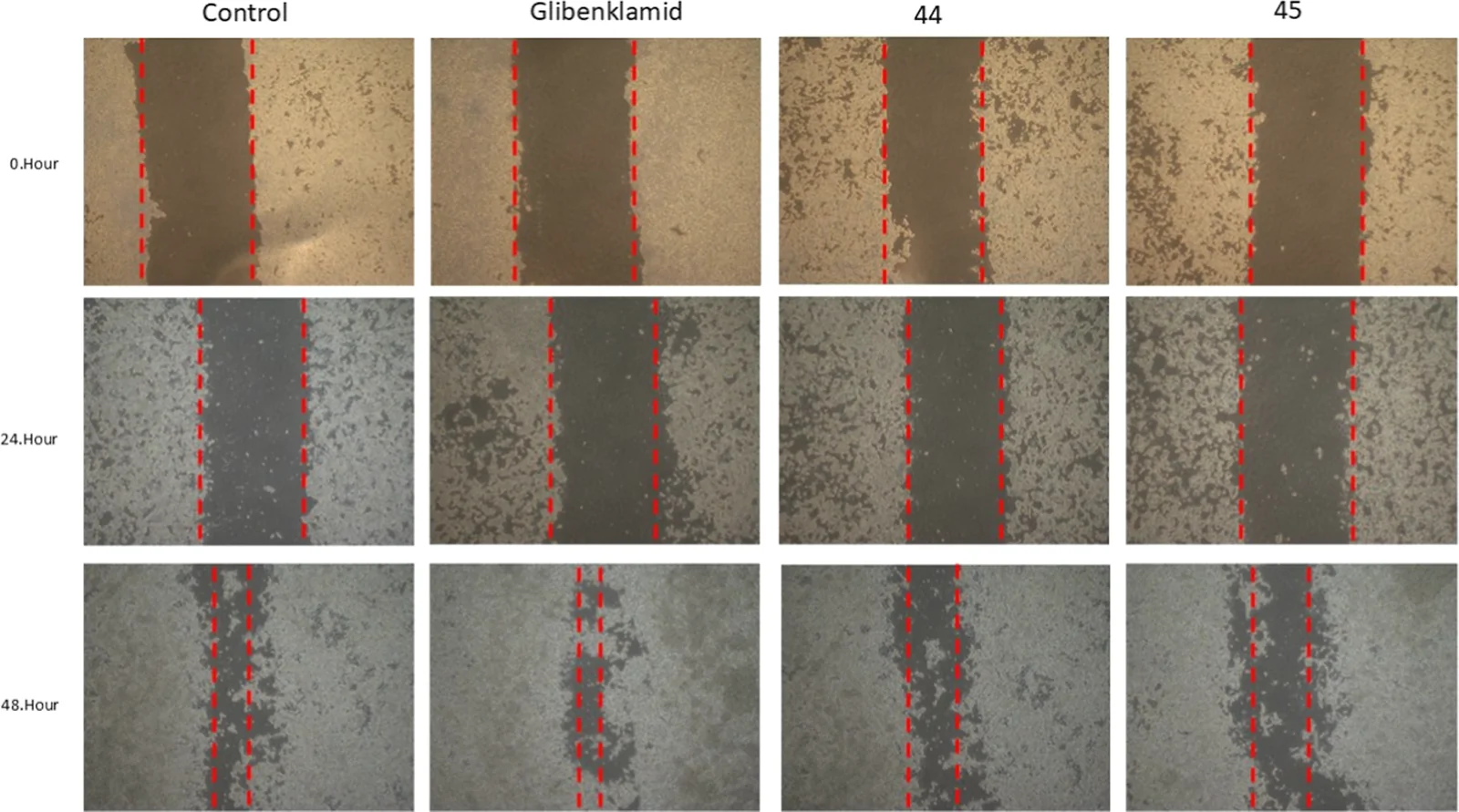
Scratch wound healing assay showing the effects of compounds **44**, **45** and Glibenclamide on cell migration.

Compounds **44** and **45** showed high cell viability in HEK 293 cells, with IC_50_ values of >100 μM and a safe profile at the tested concentration range. The compounds did not significantly inhibit α-glucosidase activity, indicating no inhibitory effect on this digestive enzyme. Both compounds activated PPAR-γ in a concentration-dependent manner, with compound **45** showing moderate activity and compound **44** having lesser activation. According to the results, compound **44** exhibited no cytotoxic effect while significantly promoting cell migration and inducing measurable PPAR-γ activation, highlighting its potential for further investigation.

#### 3.2.5 Structural Activity Relationship of Sulfonylurea-Pyrazoline Hybrids

The design of PPAR-γ agonists is generally a pharmacophoric model consisting of three essential functional regions that control ligand–receptor interactions and biological activity. First, the hydrophobic region plays a crucial role in stabilizing ligand binding through van der Waals and hydrophobic interactions within the receptor’s ligand-binding domain. In this context, the cyclohexyl group in the compounds **44** and **45** increases binding affinity by interacting with important amino acid residues such as His449, Phe363, Met364, Ile281, and Cys285. Second, the linker/aromatic region provides the necessary conformational flexibility and confirms the proper orientation of the ligand within the binding pocket. The pyrazolin ring forms this structural skeleton (Figure 6 **).** The presence of aryl groups at the C-3 and C-5 positions in the pyrazoline ring significantly affects activity. These aryl groups effectively fill the large hydrophobic pocket of PPAR-γ and contribute to ligand stabilization through π–π stacking interactions with aromatic residues in the binding site. In compounds **44** and **45**, the presence of a phenyl group at the C-3 position and either a 4-methylphenyl or 4-fluorophenyl group at the C-5 position increases receptor affinity. Specifically, the methyl substituent enhances hydrophobic interactions, while the fluorine atom contributes to improved bonding orientation and electronic optimization. Finally, the polar/acidic region is critical for receptor activation because it forms significant hydrogen bonds with residues located in the receptor’s activation function-2 (AF-2) region. The sulfonylurea moiety fulfills this role through – SO_2_ and – NH groups, which function as hydrogen bond acceptors and donors. This group typically interacts with key residues such as Tyr473, His323, His449, and Ser289, facilitating receptor activation. In summary, the coordinated contribution of the hydrophobic cyclohexyl ring, the aryl-substituted pyrazoline linker, and the hydrogen bond-forming sulfonylurea group is necessary to achieve potent and selective PPAR-γ agonistic activity.^48–51^

**Figure 6:**
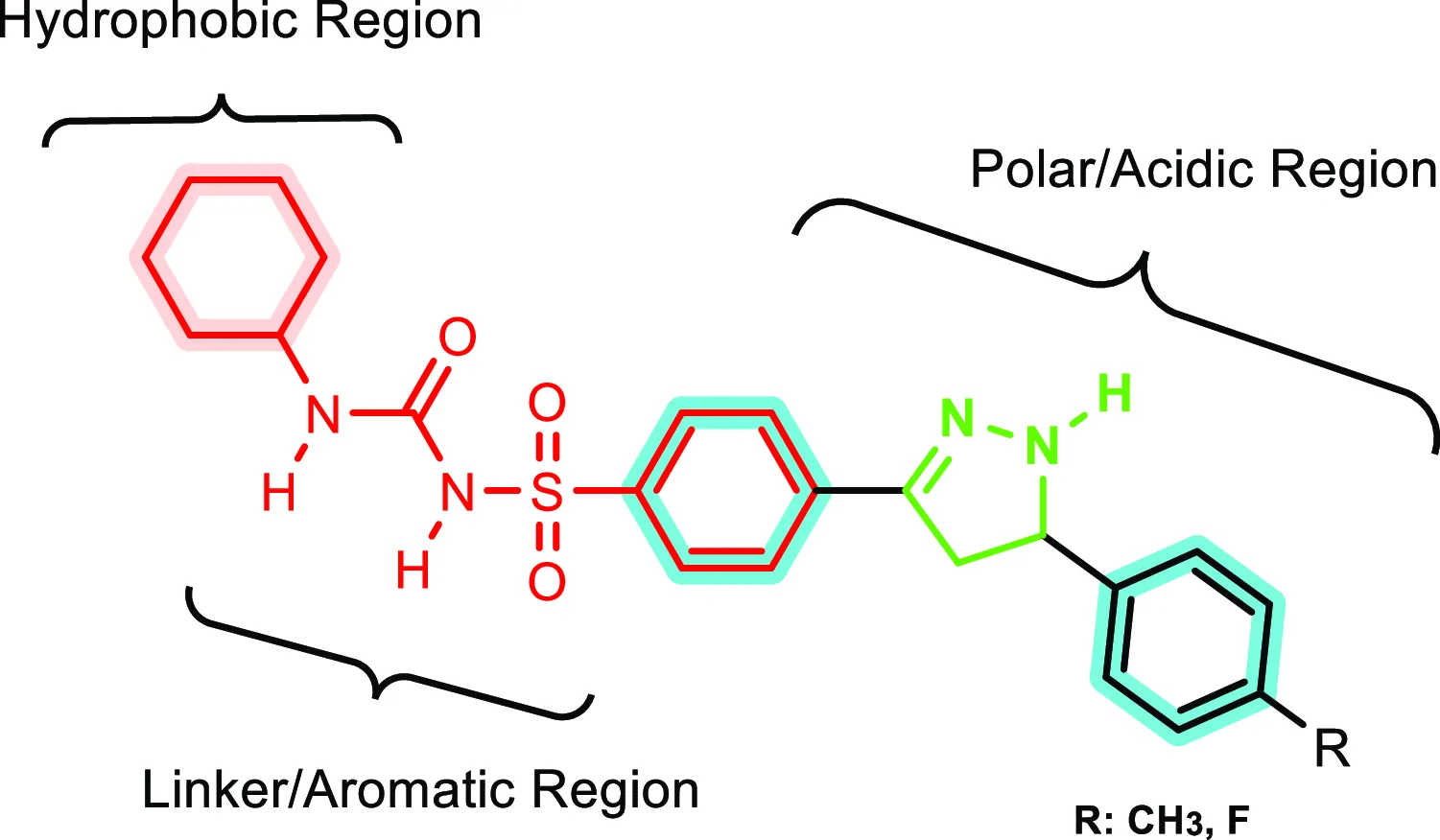
Newly synthesized compounds exhibiting structural features of PPARγ agonists.

### 3.3 Molecular Dynamics (MD) Simulations and MM/PBSA Free Energy Analyses Results

MD simulations indicated that the key ligand–residue interactions were consistently maintained over 200 ns for both complexes. For the PPAR-γ–44 complex, stable contacts were observed with Ala278, Ile281, Phe282, Ser289, Ala292, Glu295, Ile296, Ile326, Met329, Leu330, Leu353, Leu356, Phe360, His449, Phe226, and Cys285, supporting a persistent binding mode. Likewise, the PPAR-γ–45 complex retained stable interactions with Phe226, Ile281, Phe282, Cys285, Arg288, Ser289, Ala292, Ile326, Tyr327, Met329, Leu330, Leu353, Leu356, Phe360, Phe363, and Met364 throughout the 200 ns MD simulation time (Figure 7).

**Figure 7:**
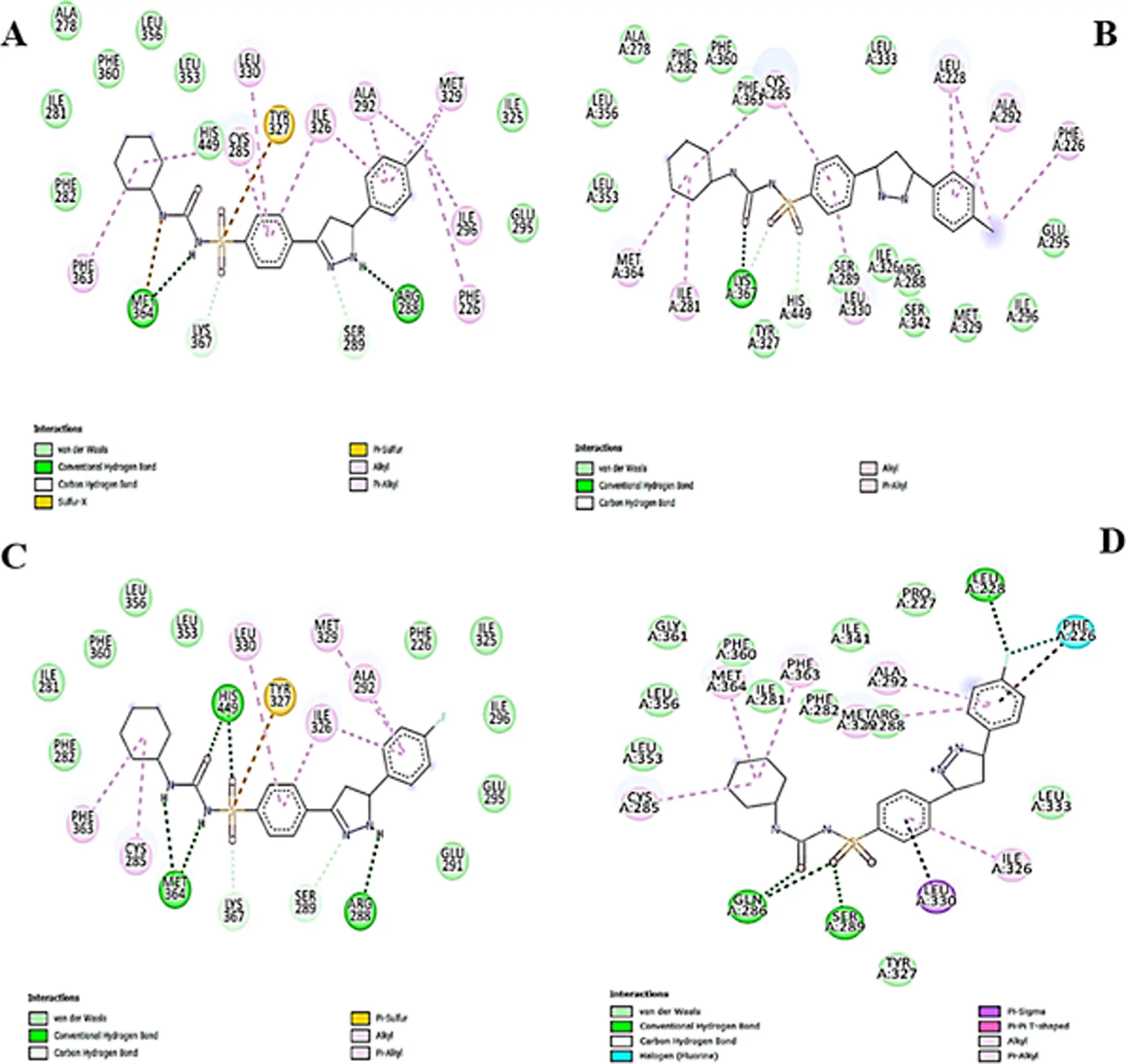
Initial state of compound **44** with PPAR-γ (A) and its final state post a 200 ns MD simulation (B), and the initial state of compound **45** with PPAR-γ (C) and its final state post a 200 ns MD simulation (D).

RMSD analysis showed that, for the PPAR-γ–44 system, the protein RMSD varied between 0.0005 and 0.27 nm, the ligand RMSD between 0.0004 and 0.21 nm, and the overall complex RMSD between 1.13 and 1.29 nm (Figure 8A). Similarly, for the PPAR-γ–45 system, RMSD values ranged from 0.0005 to 0.27 nm for the protein, 0.0004 to 0.21 nm for the ligand, and 1.15 to 1.29 nm for the complex (Figure 8A). Overall, both complexes exhibited stable RMSD profiles without pronounced drift, indicating convergence to a stable conformational state during the simulation.

**Figure 8:**
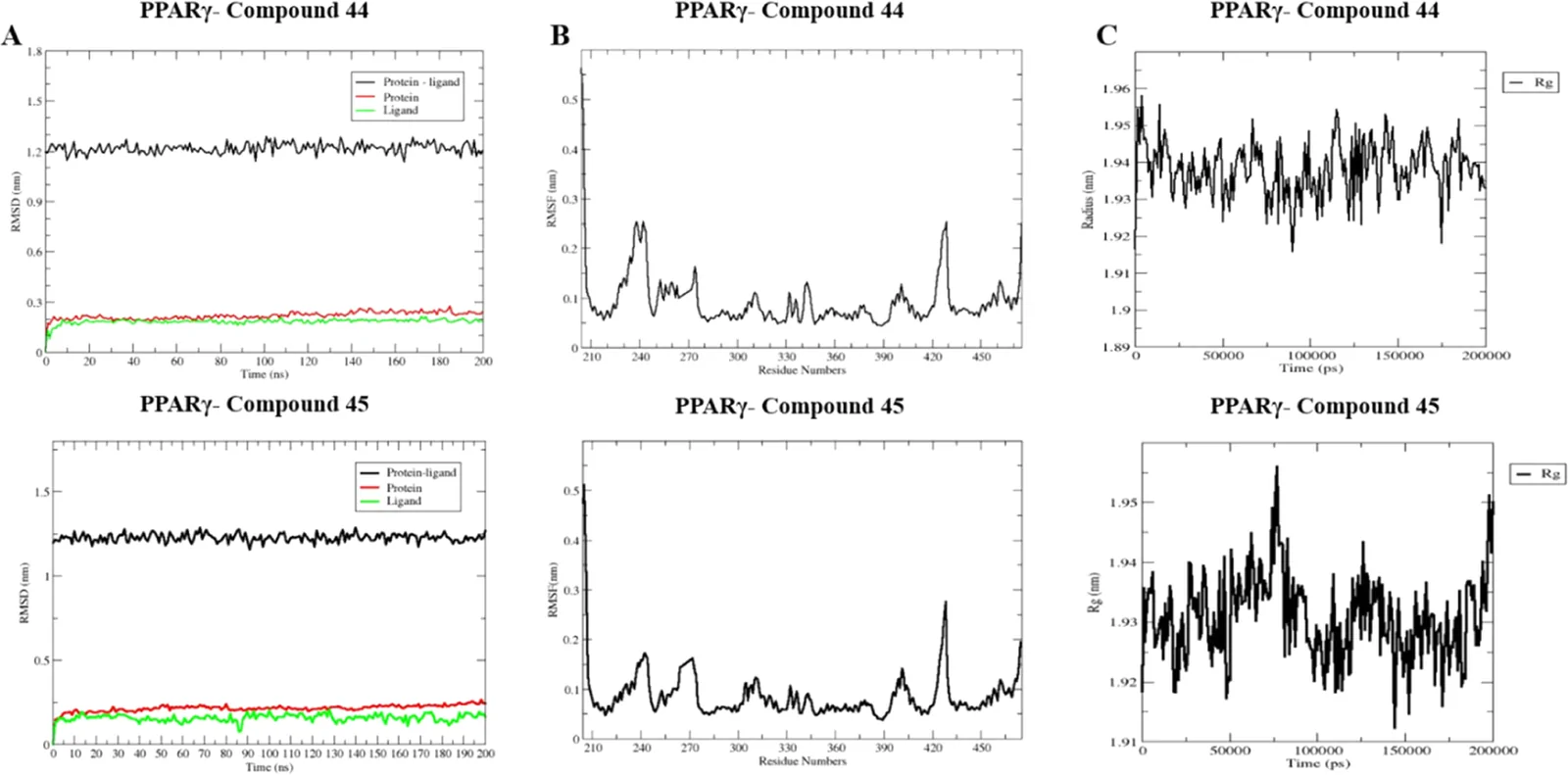
RMSD analysis of the PPAR-γ enzyme with the data from the 200 ns MD simulation of Compounds **44** and **45**. (A) The purple hue denotes the protein, the green hue signifies the ligand, and the black hue illustrates the protein–ligand complex structure. (B) The RMSF curve of the PPAR-γ enzyme during the 200 ns molecular dynamics simulation. (C) The Rg study examines the PPAR-γ enzyme within the protein complex structure throughout the 200 ns duration of the MD simulation.

RMSF analysis indicated limited residue-level fluctuations within the PPAR-γ activation site for both complexes. In the PPAR-γ–44 complex, residues Phe226, Ala278, Ile281, Phe282, Cys285, Ser289, Ala292, Glu295, Ile296, Ile326, Met329, Leu330, Leu353, Leu356, Phe360, Met364, and His449 exhibited consistently low RMSF values (0.04–0.56 nm), suggesting a stable local environment around the binding region. Likewise, in the PPAR-γ–45 complex, key activation-site residues Phe226, Ile281, Phe282, Cys285, Arg288, Ser289, Ala292, Ile326, Tyr327, Met329, Leu330, Leu353, Leu356, Phe360, Phe363, and Met364 showed low fluctuations throughout the simulation (0.03–0.52 nm) (Figure 8B).

The radius of gyration (Rg) was evaluated to assess the overall compactness of PPAR-γ during the MD simulations. In both ligand-bound systems, Rg values remained within a narrow range of 1.91–1.96 nm over 200 ns, indicating that PPAR-γ maintained stable structural compactness throughout the simulations (Figure 8C).

MM/PBSA binding free energy analyses revealed that the binding free energy of the PPAR-γ-44 complex is −178.113 ± 16.976 kJ/mol, with van der Waals energy at −254.118 ± 14.276 kJ/mol, electrostatic energy at −48.500 ± 7.569 kJ/mol, polar solvation energy at 149.832 ± 12.189 kJ/mol, and solvent accessible surface area (SASA) energy at −25.327 ± 0.896 kJ/mol. The binding free energy of the PPAR-γ-45 complex was determined to be −161.991 ± 14.096 kJ/mol, with van der Waals energy at −241.071 ± 10.775 kJ/mol, electrostatic energy at −23.324 ± 7.330 kJ/mol, polar solvation energy at 126.763 ± 11.920 kJ/mol, and solvent-accessible surface area (SASA) energy at −24.359 ± 0.765 kJ/mol.

## 4 Conclusions

The structures of the chalcones and pyrazoles were successfully characterized, with yields ranging from 14% to 87%. In this study, we noticed the formation of byproducts during reaction monitoring using TLC; because more than two speckles were observed in the reaction except for the initiations, purification was carried out by column chromatography. Compounds **44** and **45** exhibited high cell viability in HEK-293 cells (IC_50_ > 100 μM) and showed negligible α-glucosidase inhibitory activity. In contrast, these compounds demonstrated notable activity toward PPAR-γ, with compound **45** showing particularly strong effects compared to the reference compound. Furthermore, *in silico* molecular dynamics analyses revealed that compounds **44** and **45** formed stable interactions with the PPAR-γ enzyme throughout the simulation period. Overall, these findings indicate that compounds **44** and **45** exhibit a selective pharmacological profile favoring PPAR-γ modulation rather than a dual-target mechanism. PPAR-γ is a vital transcription factor that directly influences insulin resistance by regulating carbohydrate and lipid metabolism. Based on the findings of this study, it is planned to conduct *in vivo* experiments with these molecules and to synthesize new candidate compounds targeting PPAR-γ based on these lead structures.

## Supplementary Material



## Notes

^1^H NMR, ^13^C NMR, FT-IR, and Mass spectrum of compounds chalcone and pyrazole (PDF)
