# Supplementary material for: Design, in Vitro Biological Activities and Molecular Dynamics Simulations of Novel Chalcone and Pyrazole Derivatives Targeting α‑Glucosidase and PPAR‑γ as Antidiabetic Agents
Source: ACS Omega. 2026 Aug 14;11(33):49464–79. doi: 10.1021/acsomega.6c02853 (PMC13520005; doi:10.1021/acsomega.6c02853)
Supplement: Supplementary file 1 [file ao6c02853_si_001.pdf]

SUPPLEMENTARY DATA

Title: Design, in vitro biological activities and molecular dynamics simulations of novel chalcone and pyrazole derivatives targeting α-glucosidase and PPAR-γ as antidiabetic agents

Bedriye Seda Kurşun Aktar[a],*, Demet Taşdemir[b,c], Ebru Sağlam[b,d], Süleyman Kaya[e], Gizem Tatar Yılmaz[e,f,g], Ayse Sahin Yaglioglu[h], Emine Elçin Oruç-Emre[i]

aDepartment of Engineering Basic Sciences, Faculty of Engineering and Natural Sciences, Malatya Turgut Özal University, Battalgazi, Malatya, Türkiye.

bRespiratory Diseases and Respiratory Surgery Research and Practice Center, Gaziantep University, 27310, Gaziantep, Türkiye

cDepartment of Medical Biochemistry, Faculty of Medicine, Gaziantep University, 27310, Gaziantep, Türkiye

dDepartment of Chemistry, Graduate School of Natural and Applied Sciences, Gaziantep University, 27310, Gaziantep, Türkiye

eInstitute of Health Sciences, Department of Bioinformatics, Karadeniz Technical University, 61080, Trabzon, Türkiye

fDepartment of Biostatistics and Medical Informatics, Faculty of Medicine, Karadeniz Technical University, Trabzon, Türkiye

gYılmaz Bilişim R&D Consulting Software Engineering and Services Trade Limited Company 61081, Trabzon, Türkiye

hDepartment of Chemistry and Chemical Process Technology, Technical Sciences Vocational School, Amasya University, Amasya, Türkiye

iDepartment of Chemistry, Faculty of Arts and Sciences, Gaziantep University, 27310, Gaziantep, Türkiye

***Correspondence Author:**

Bedriye Seda Kurşun Aktar

Department of Engineering Basic Sciences, Faculty of Engineering and Natural Sciences, Malatya Turgut Özal University, Battalgazi, Malatya, Turkey

E-mail: bseda.kursunaktar@ozal.edu.tr

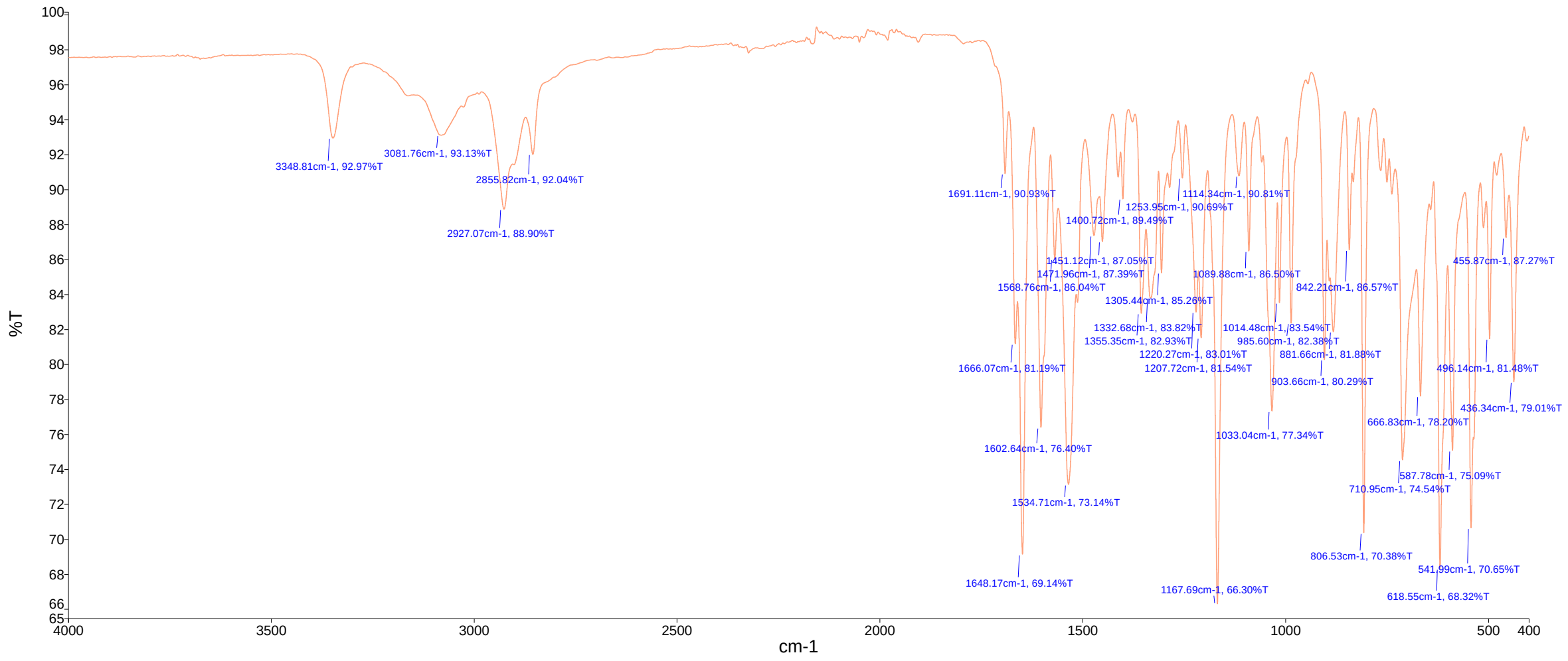

Figure S1. FTIR spectrum of compound **2**

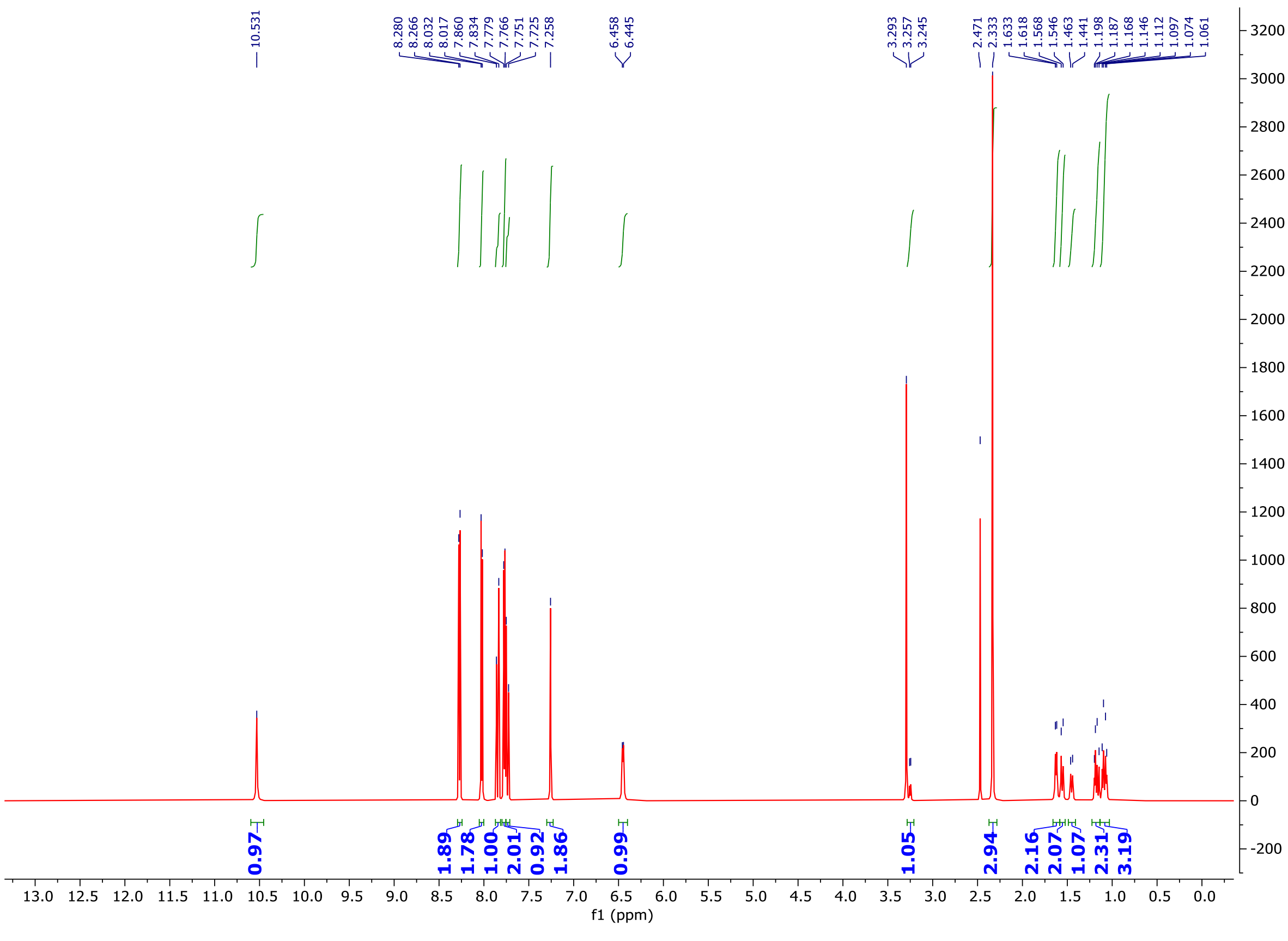

Figure S2. ^{1}H NMR spectrum of compound **2**

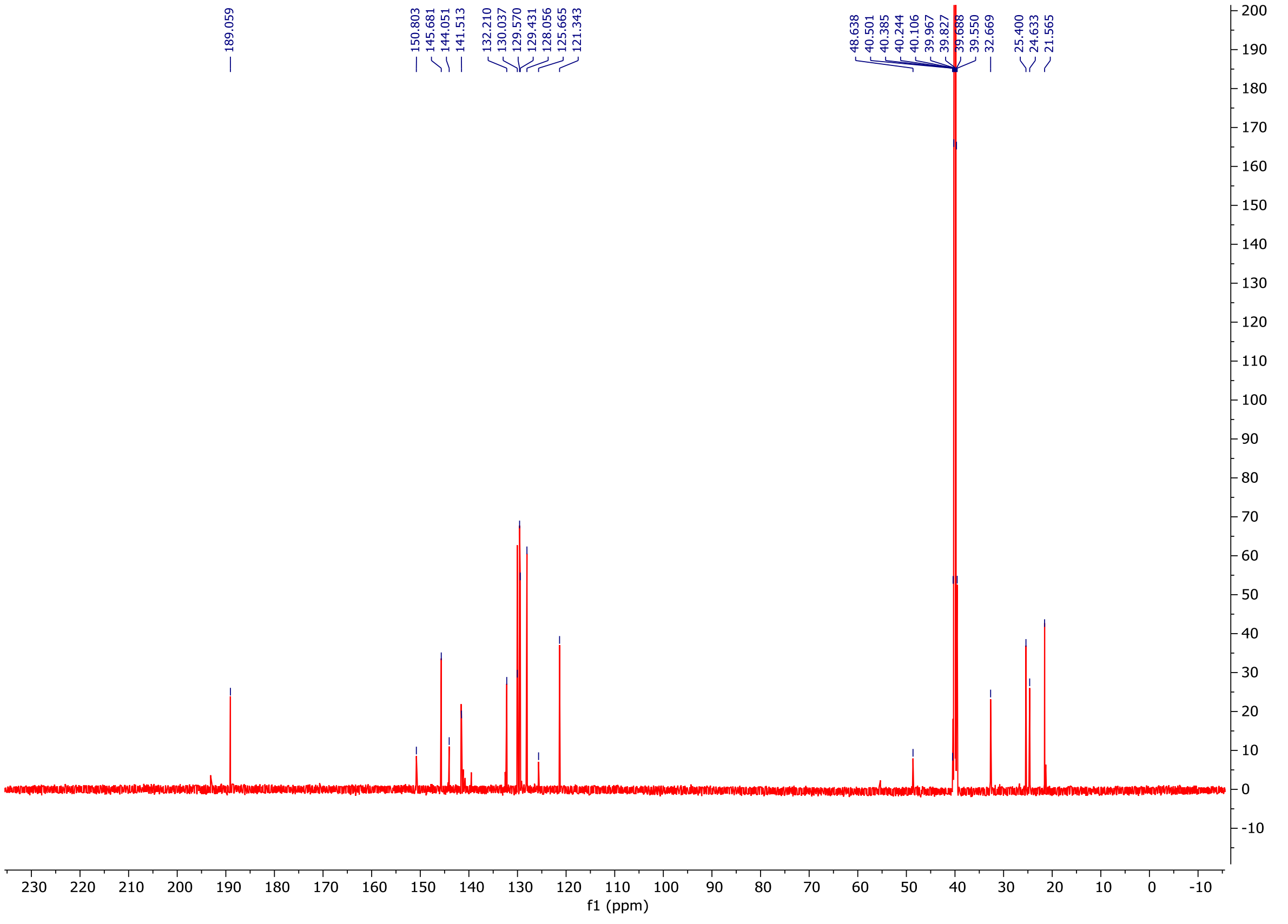

Figure S3. ^{13}C NMR spectrum of compound **2**

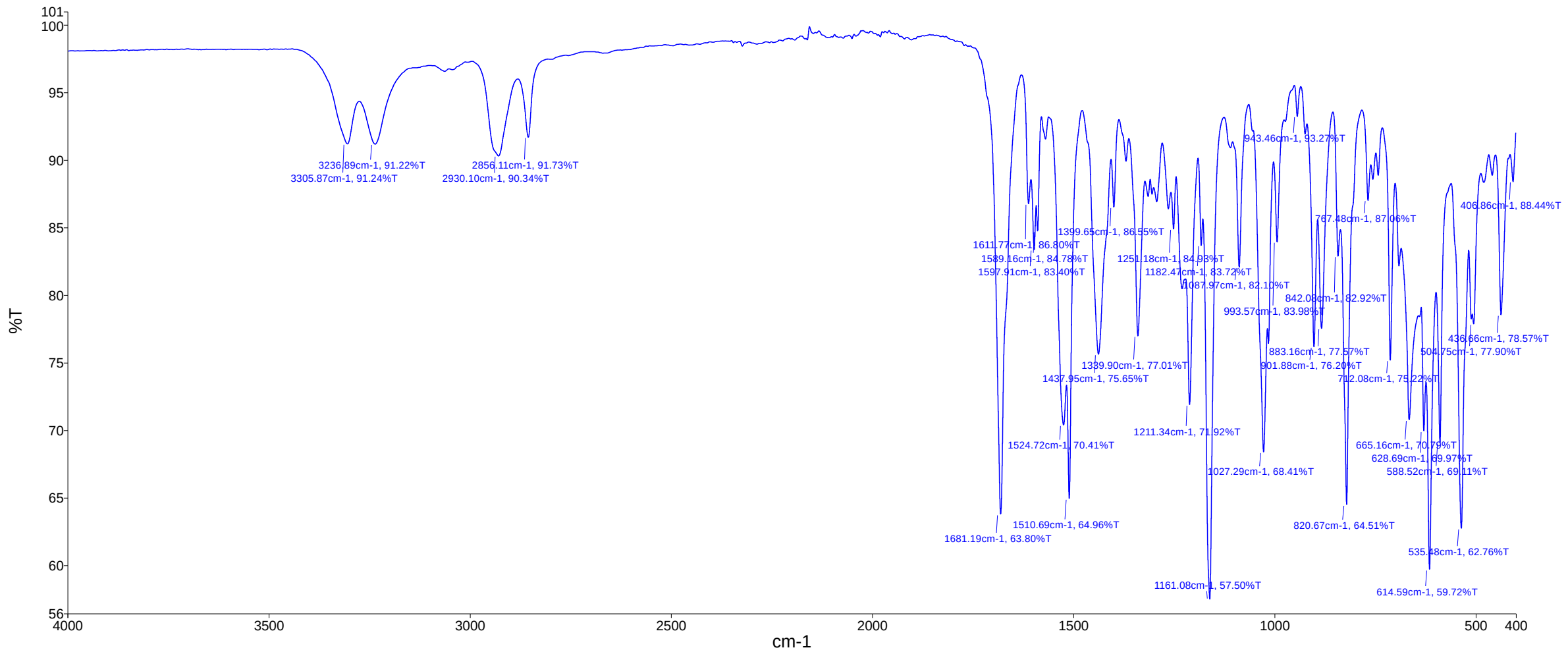

Figure S4. FTIR spectrum of compound **3**

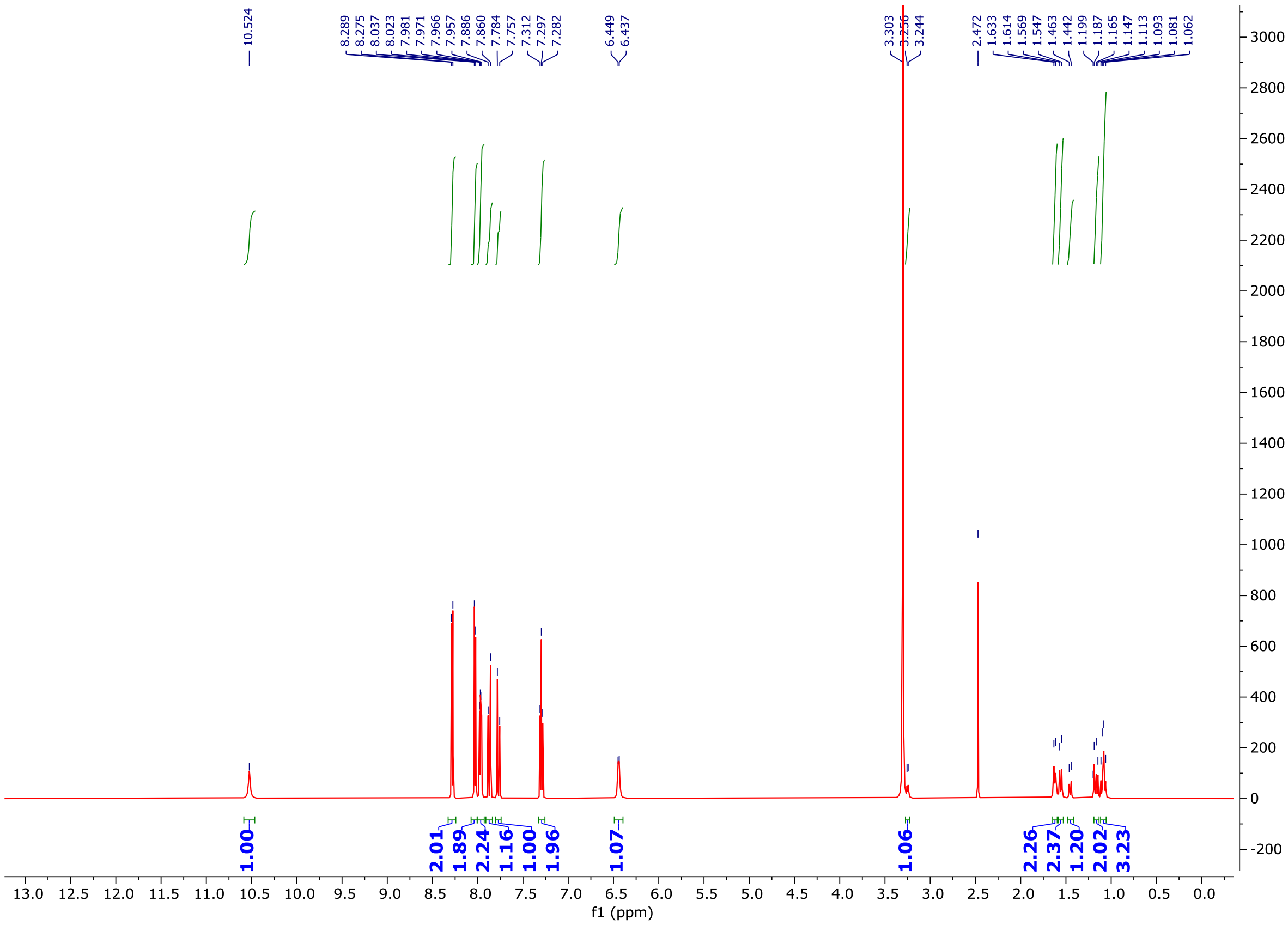

Figure S5. ^{1}H NMR spectrum of compound **3**

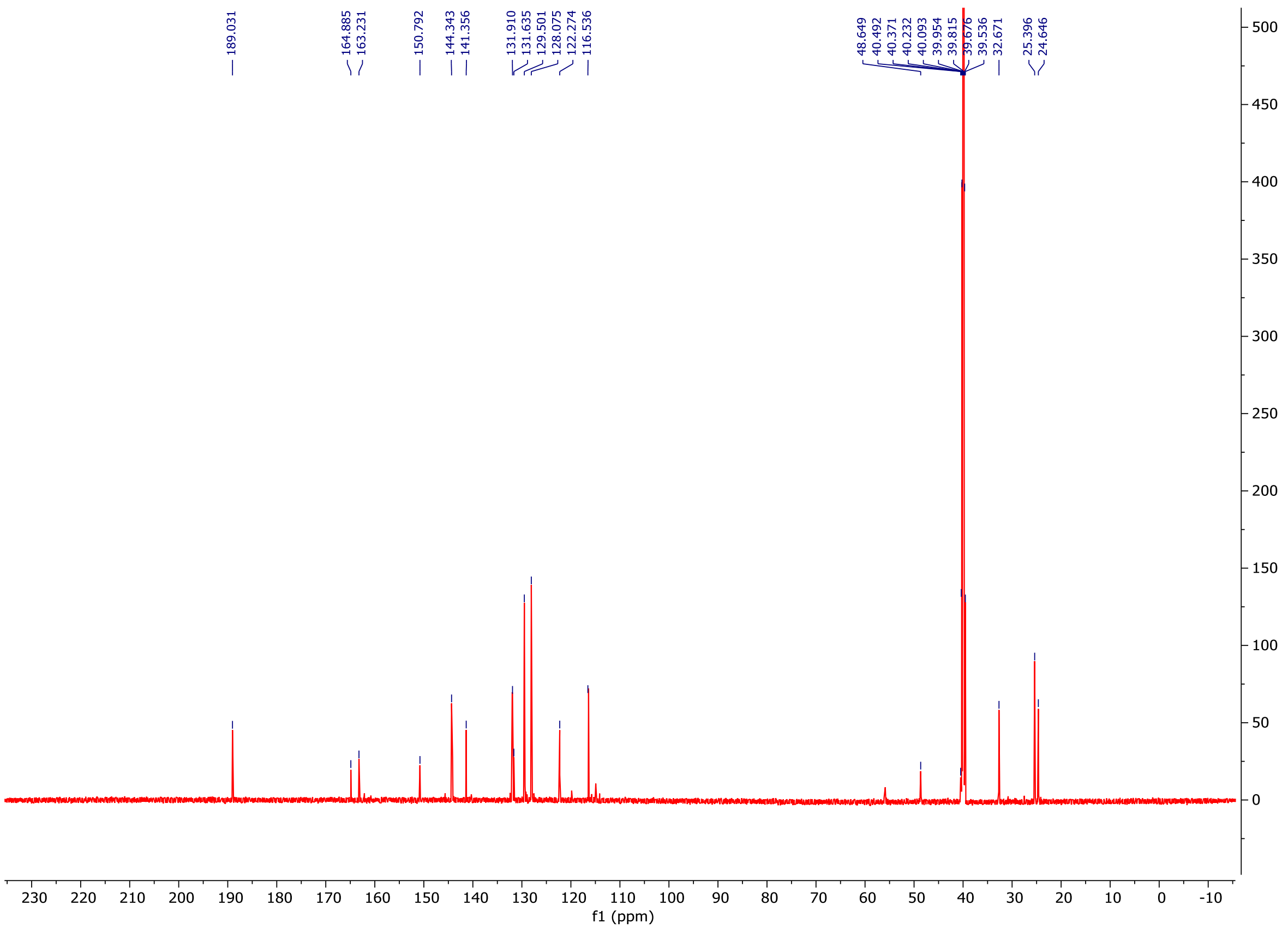

Figure S6. ^{13}C NMR spectrum of compound **3**

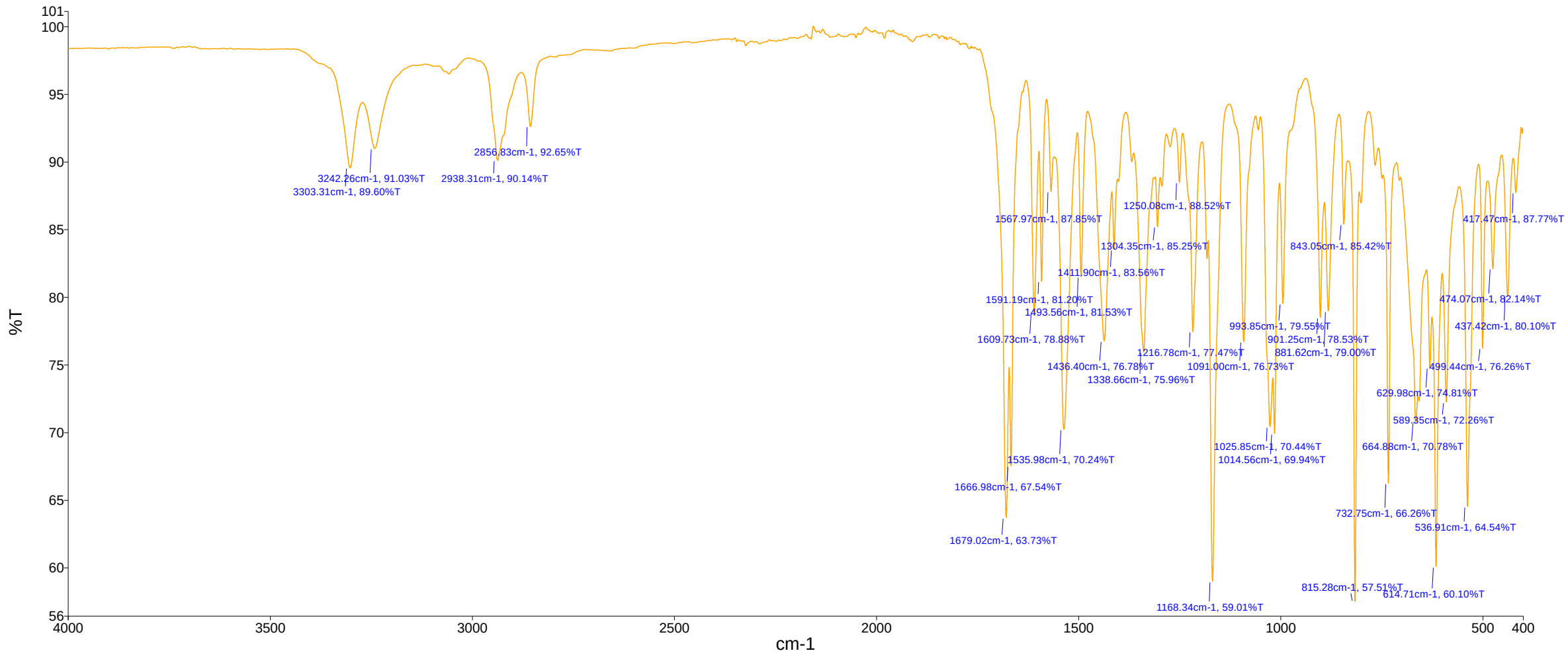

Figure S7. FTIR spectrum of compound **4**

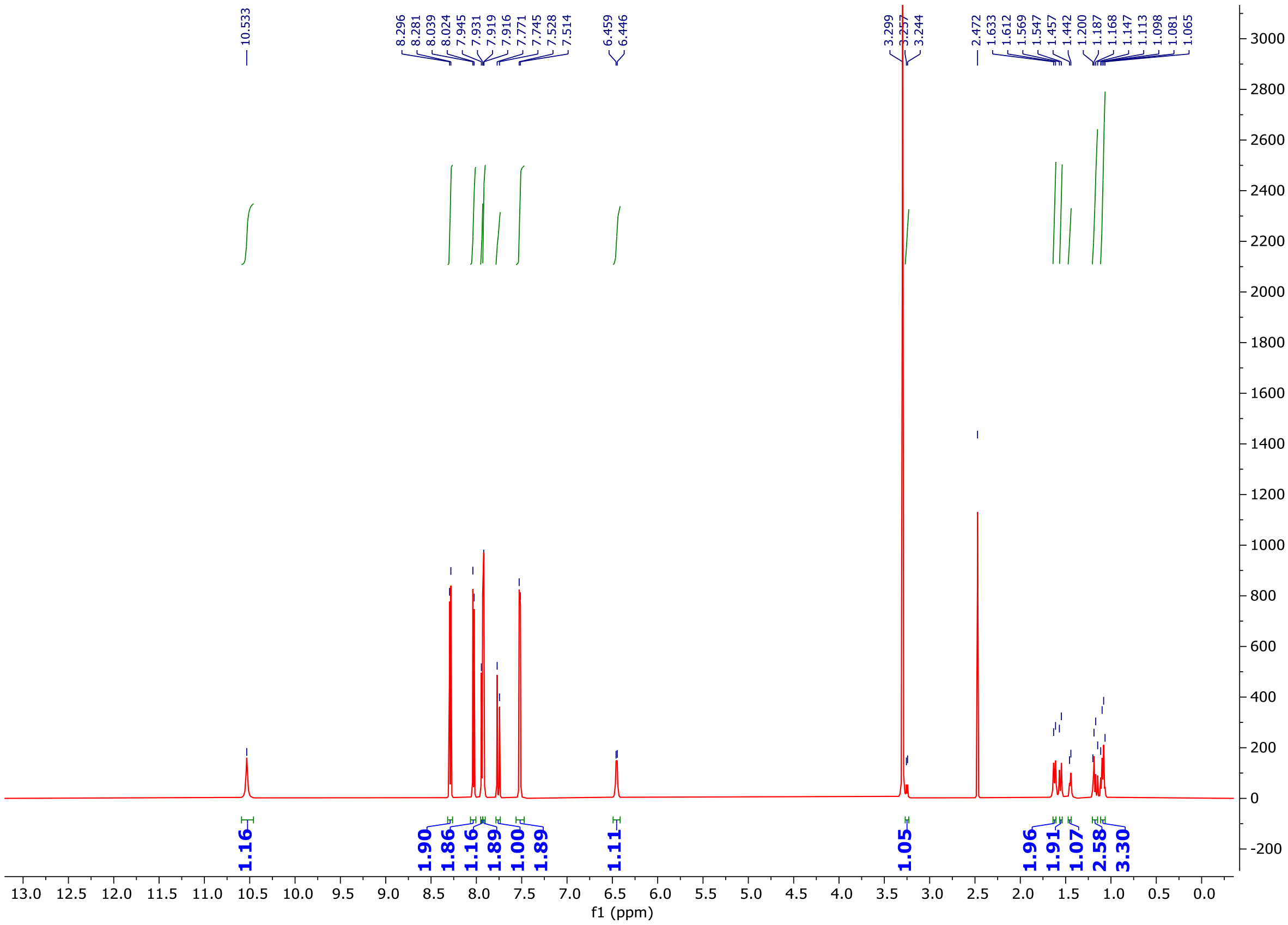

Figure S8. ^{1}H NMR spectrum of compound **4**

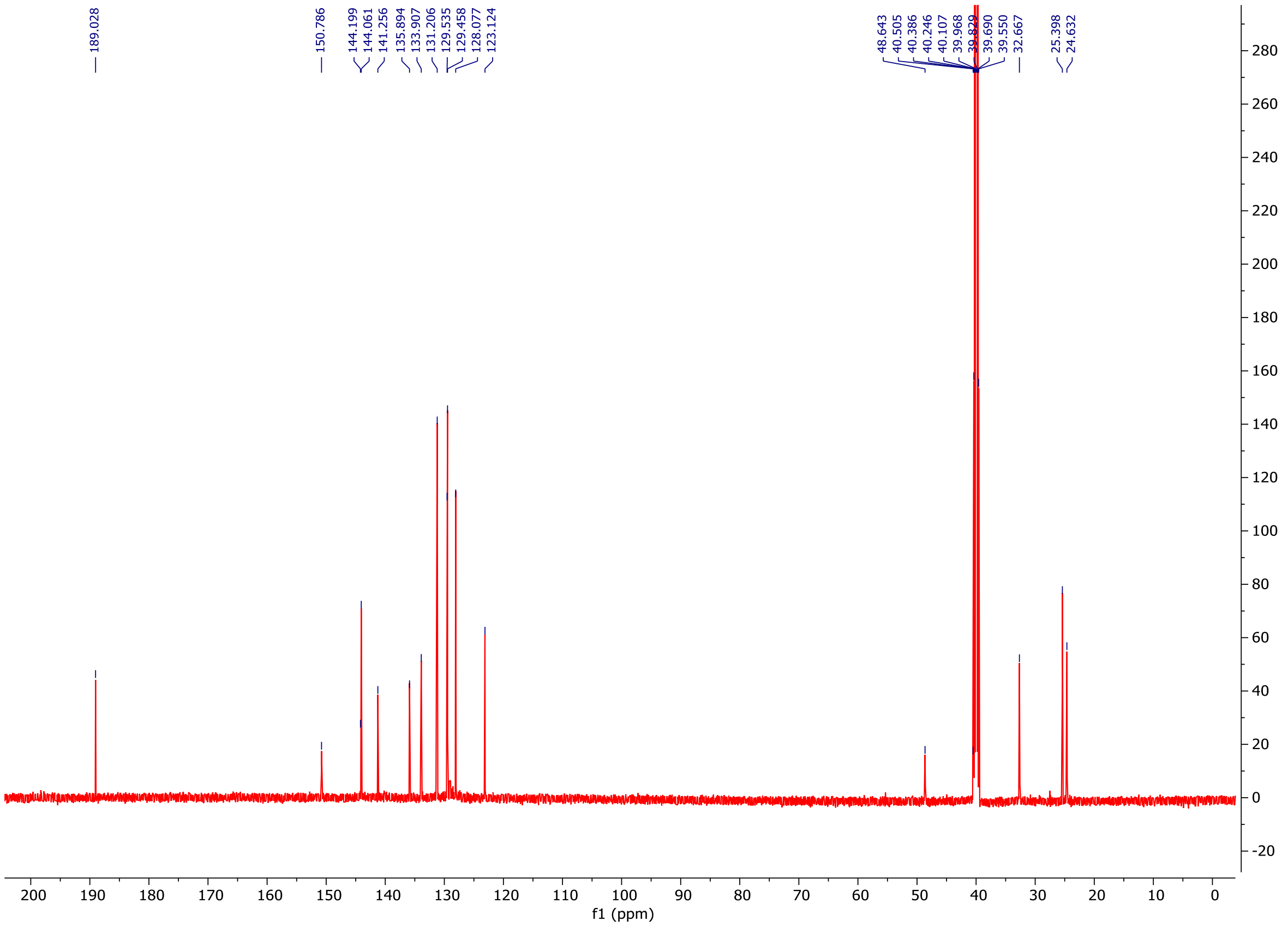

Figure S9. ^{13}C NMR spectrum of compound **4**

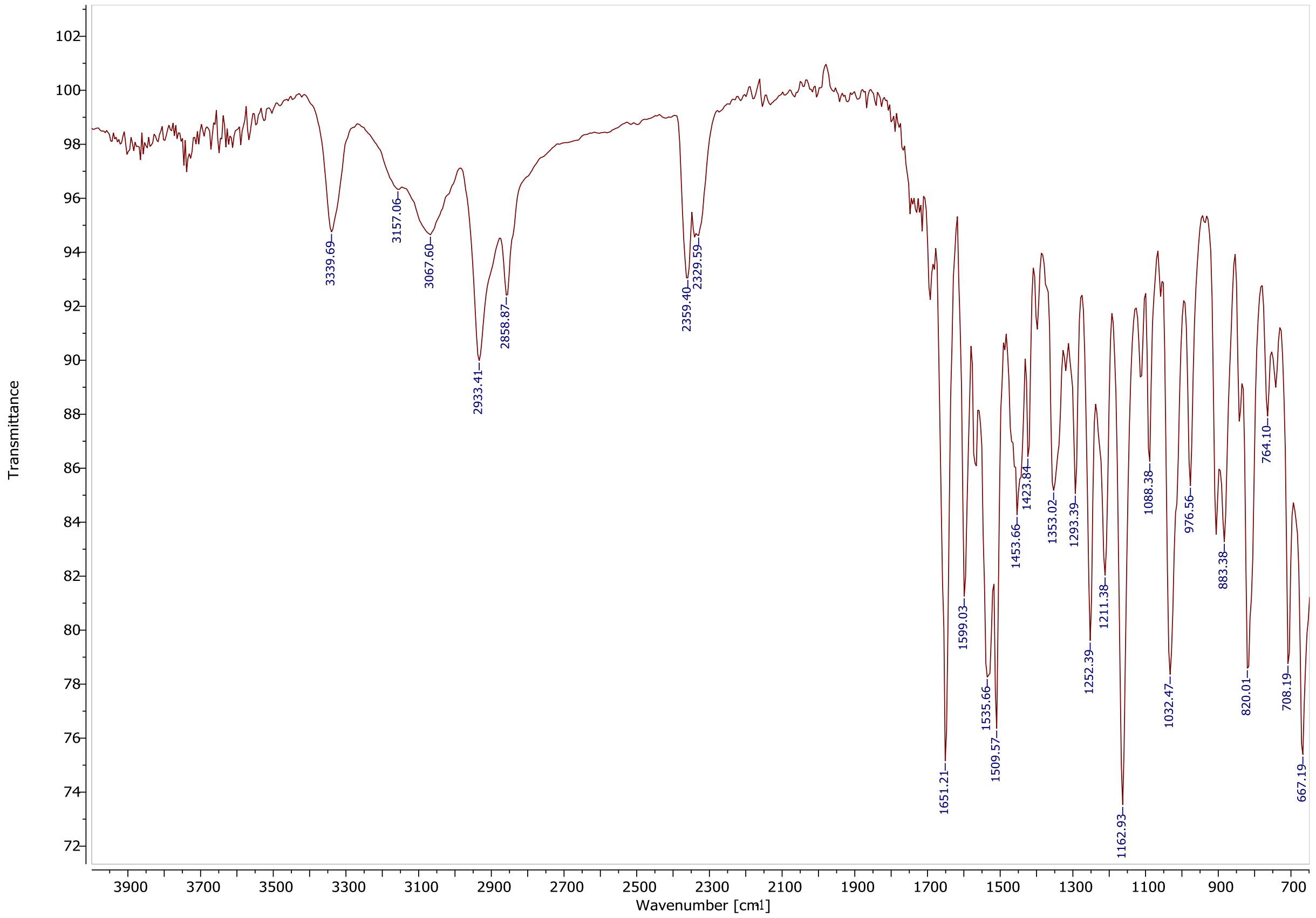

Figure S10. FTIR spectrum of compound **5**

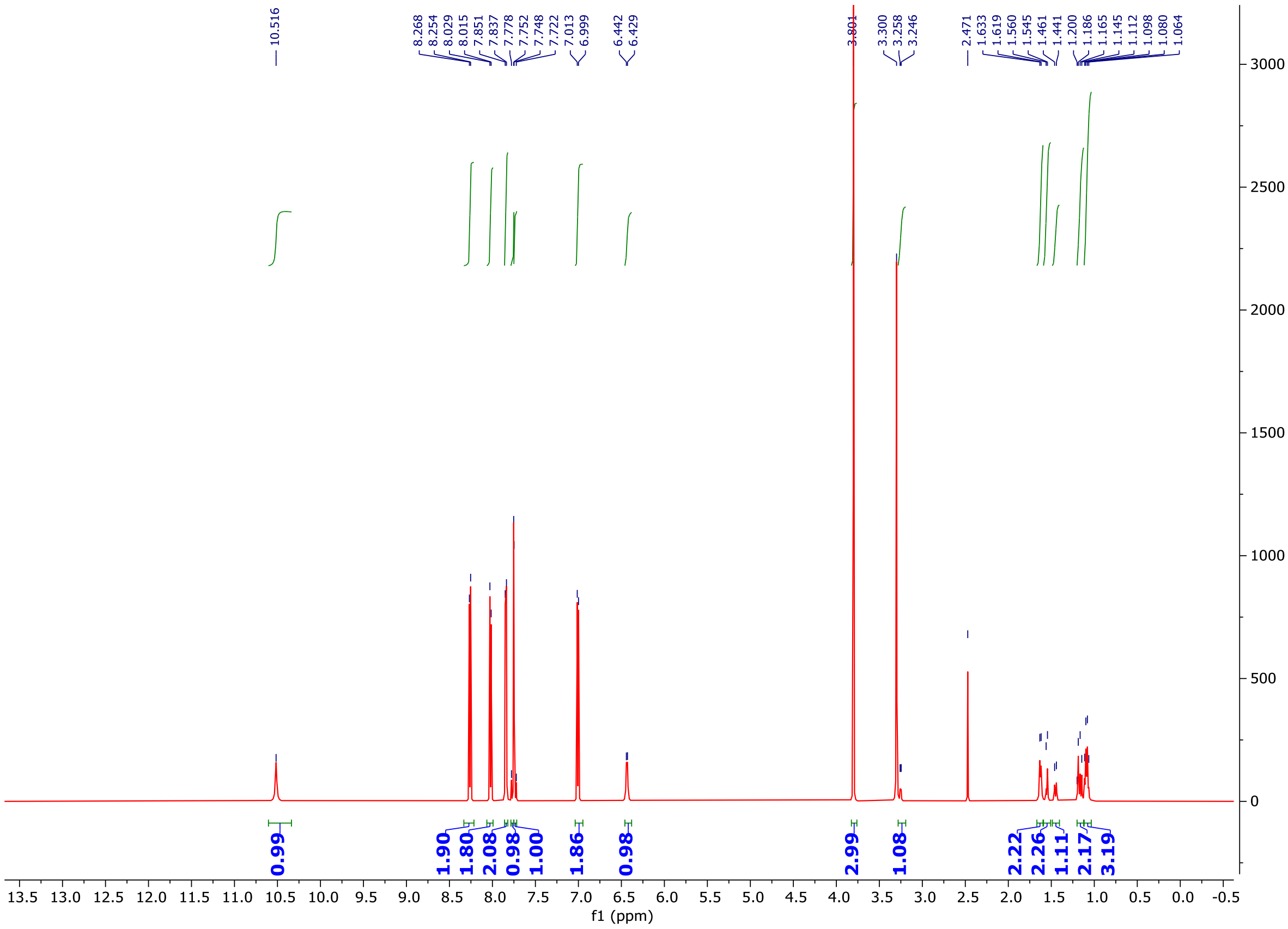

Figure S11. ^{1}H NMR spectrum of compound **5**

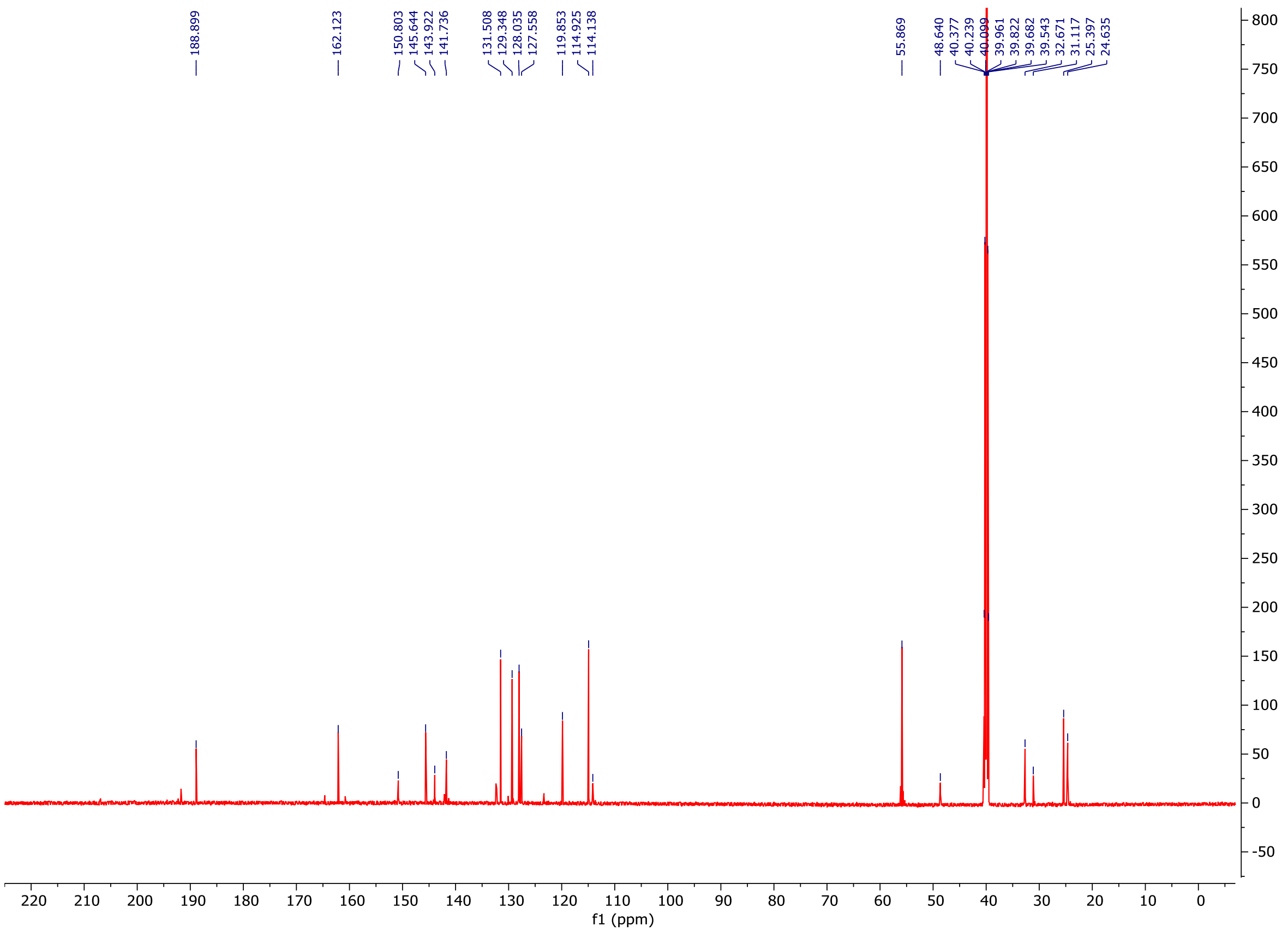

Figure S12 ^{13}C NMR spectrum of compound **5**

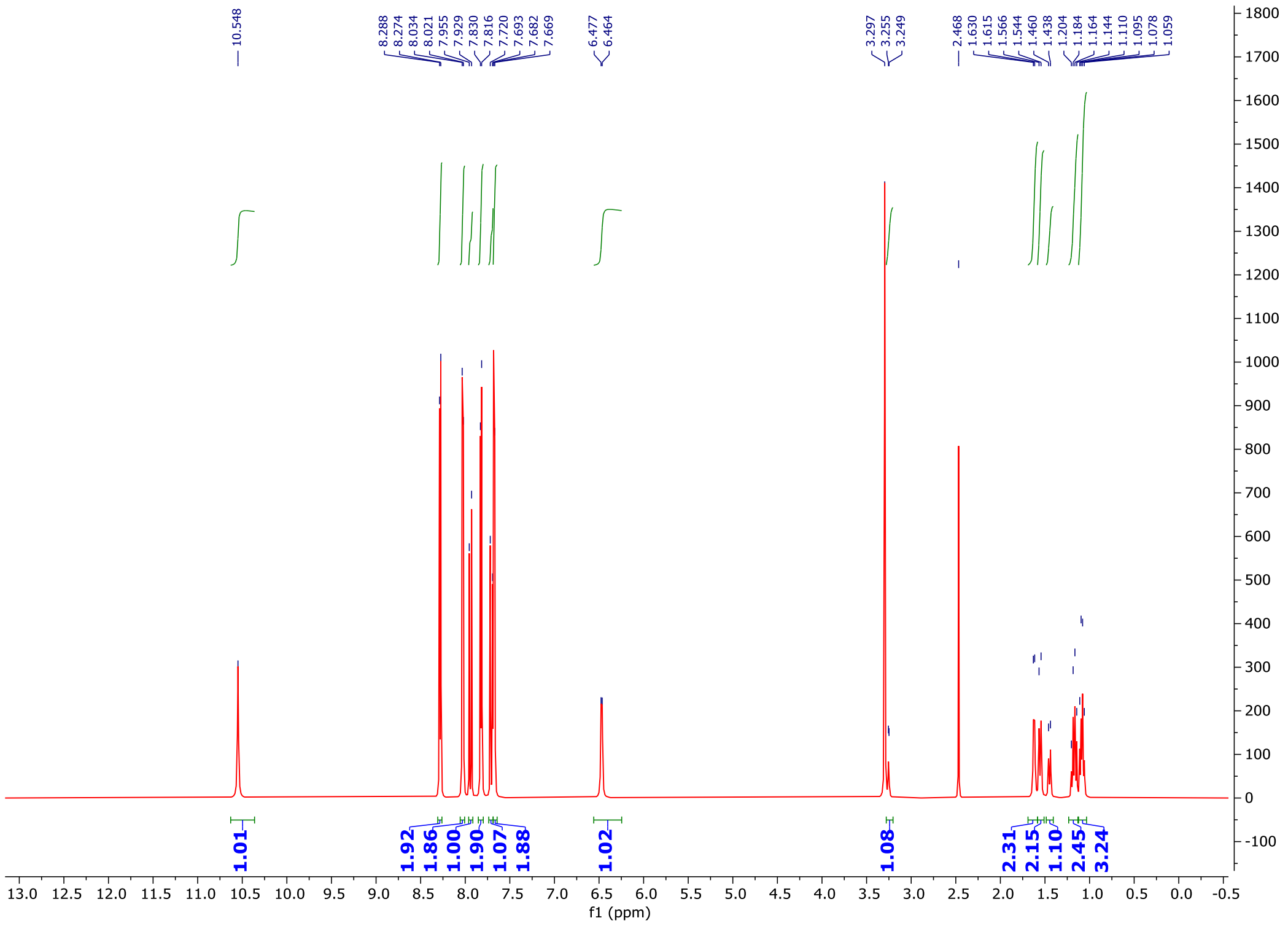

Figure S13. ^{1}H NMR spectrum of compound **7**

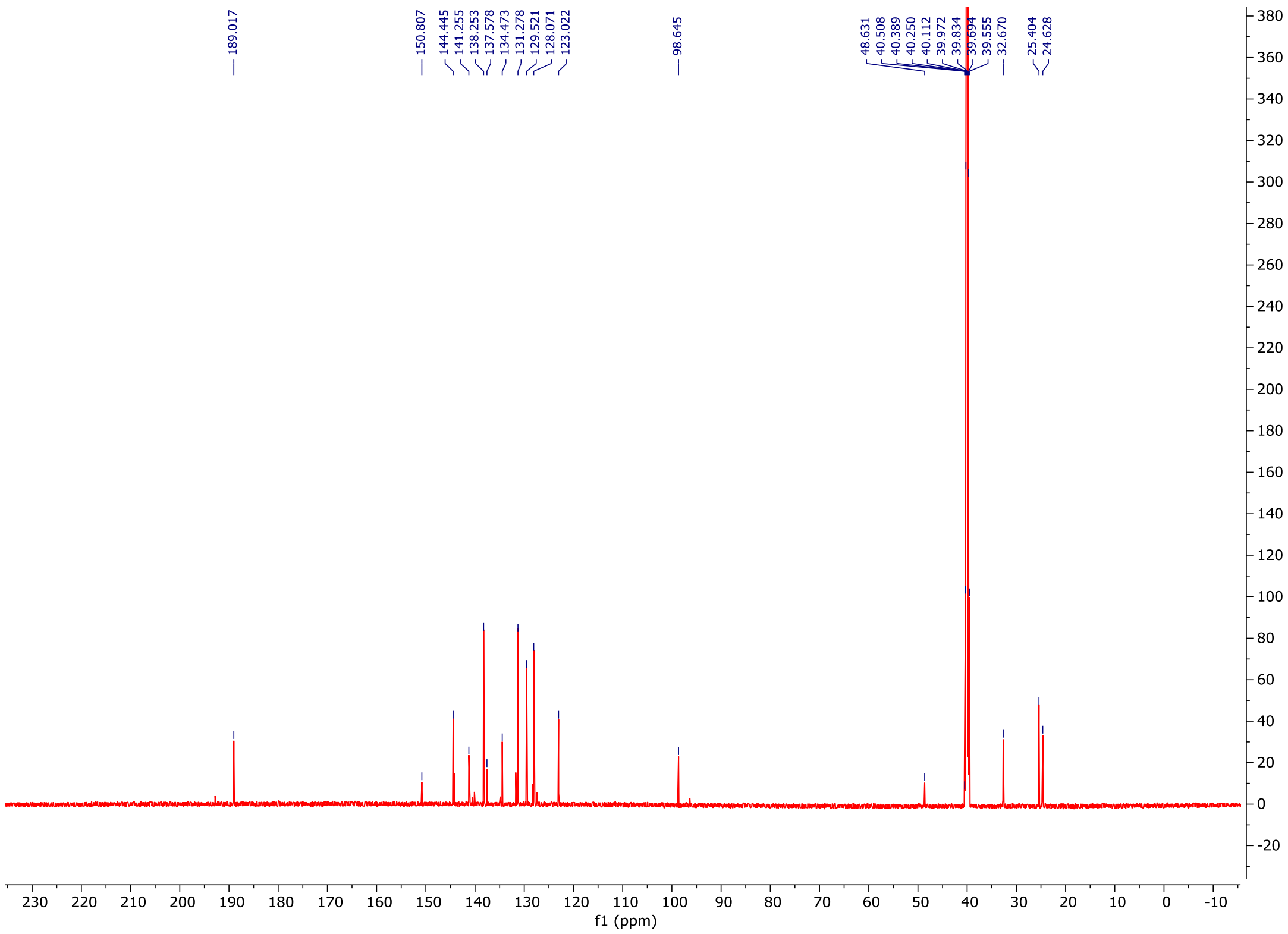

Figure S14 ^{13}C NMR spectrum of compound **7**

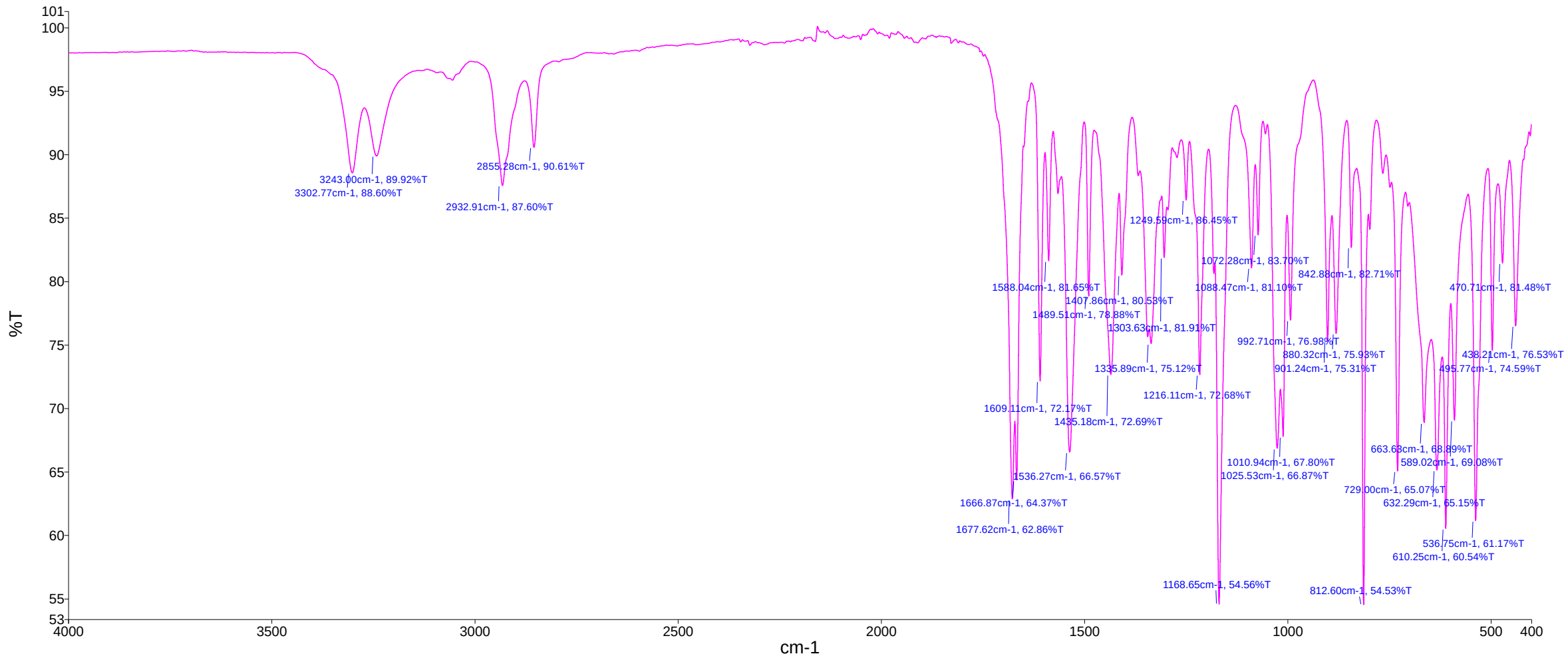

Figure S15. FTIR spectrum of compound **12**

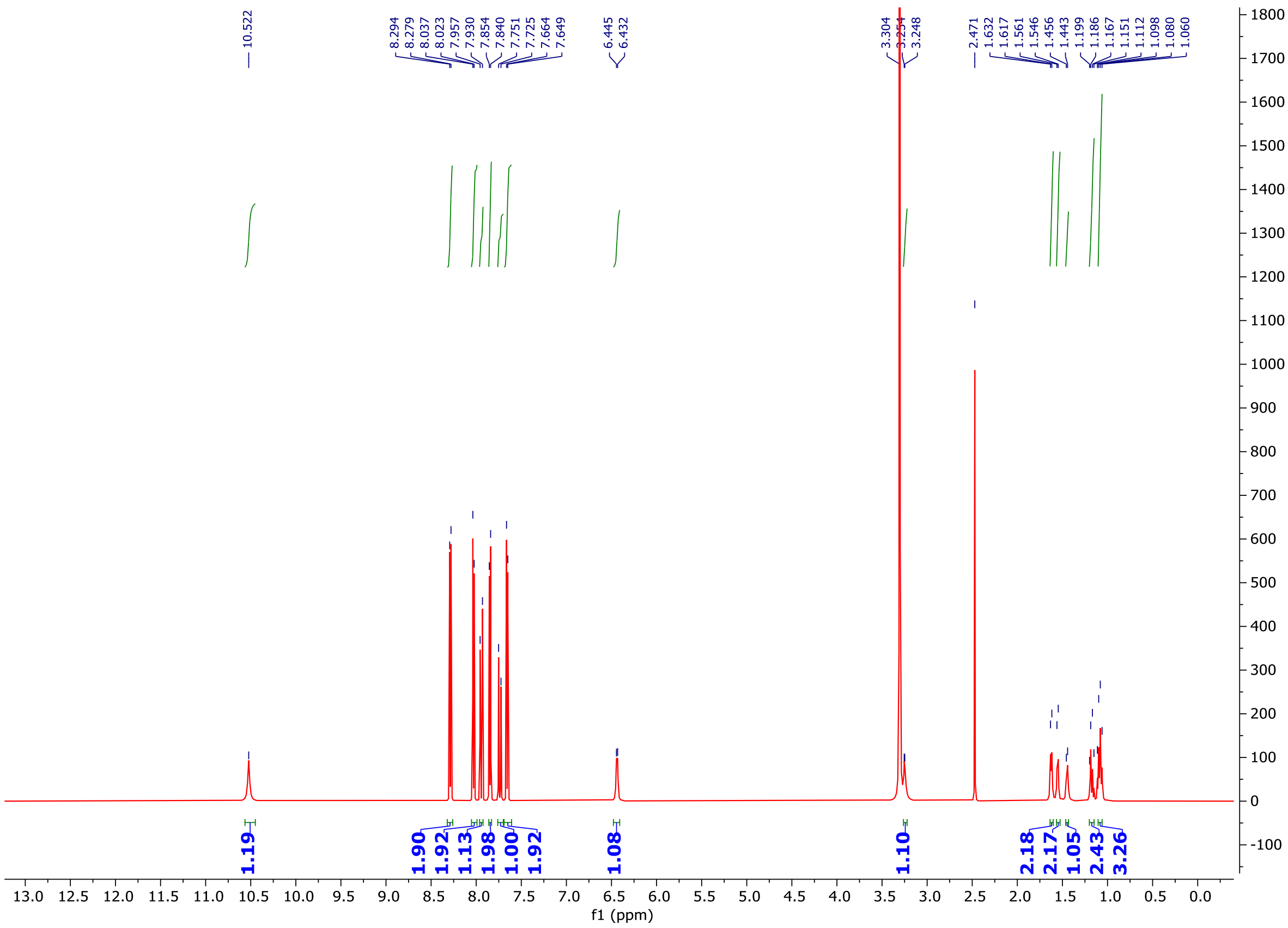

Figure S16. ^{1}H NMR spectrum of compound **12**

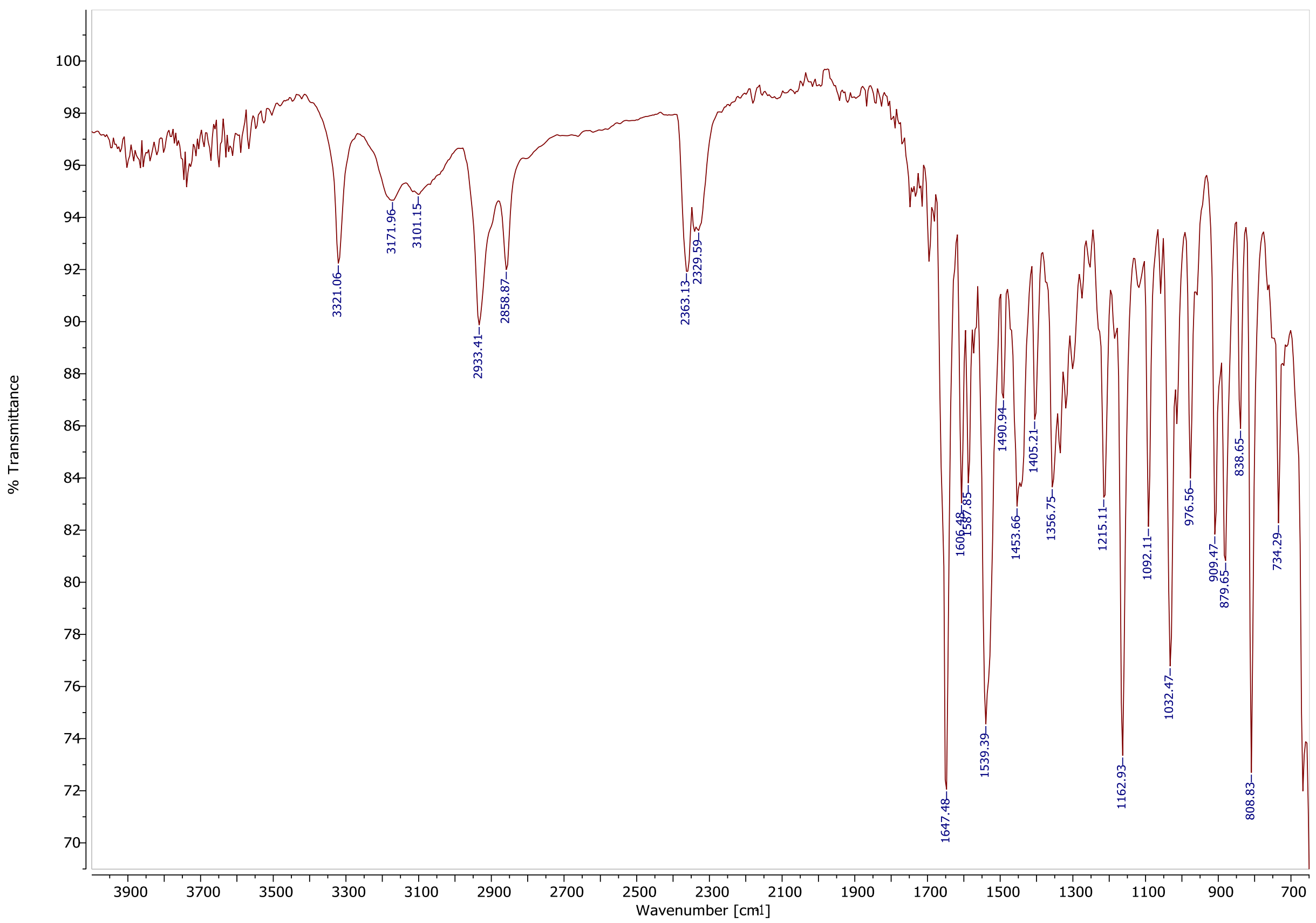

Figure S17. FTIR spectrum of compound **15**

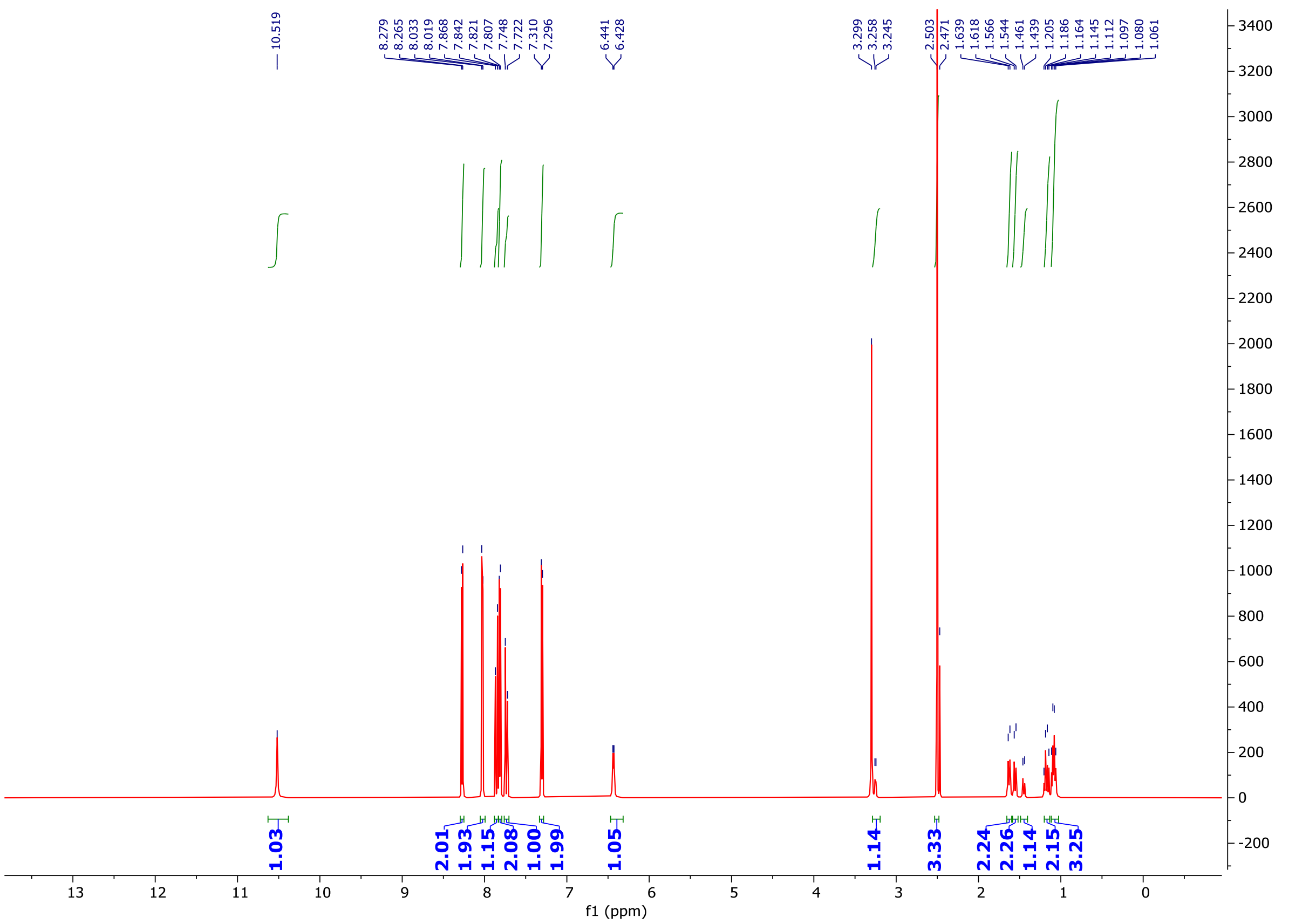

Figure S18. ^{1}H NMR spectrum of compound **15**

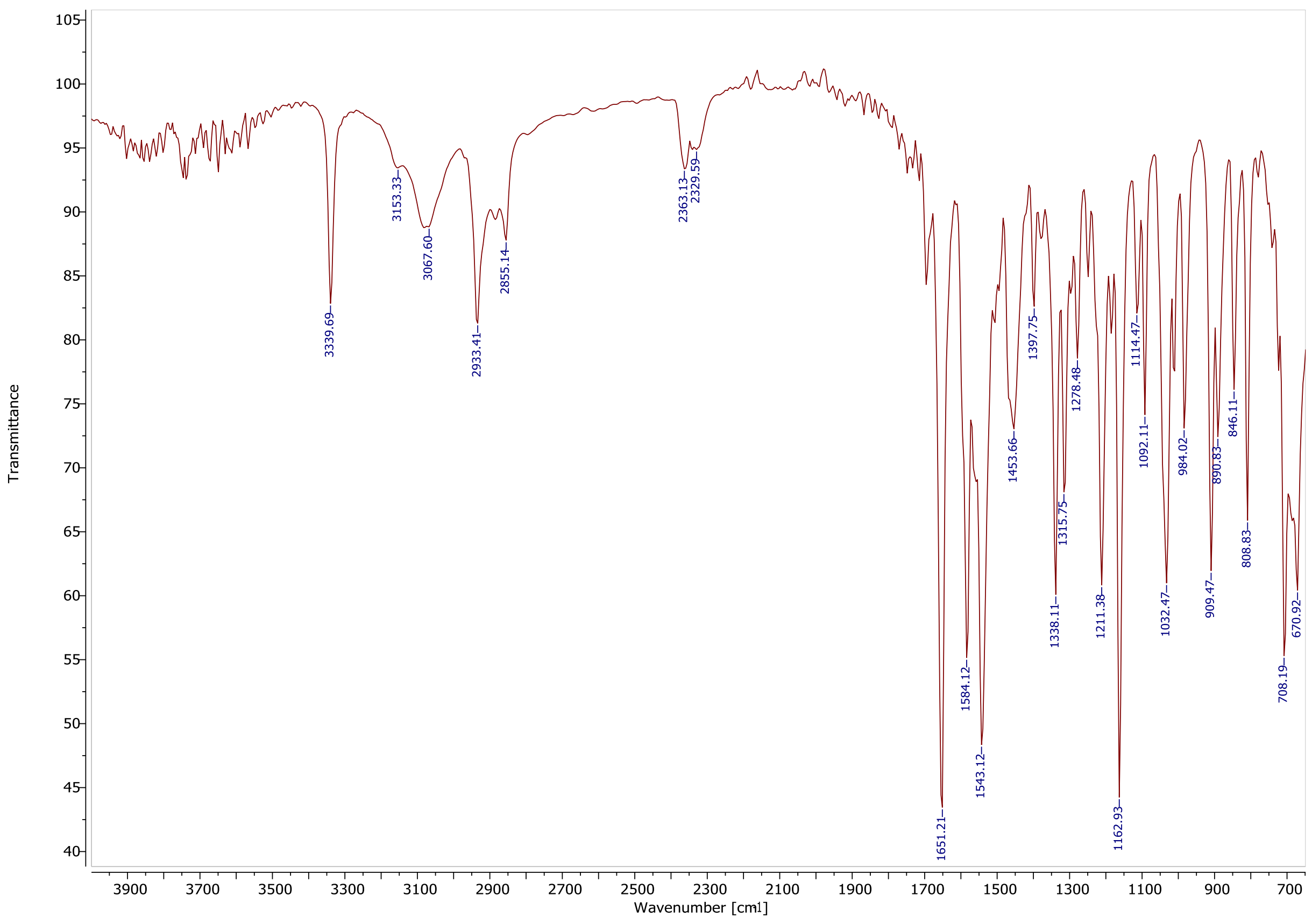

Figure S19. FTIR spectrum of compound **22**

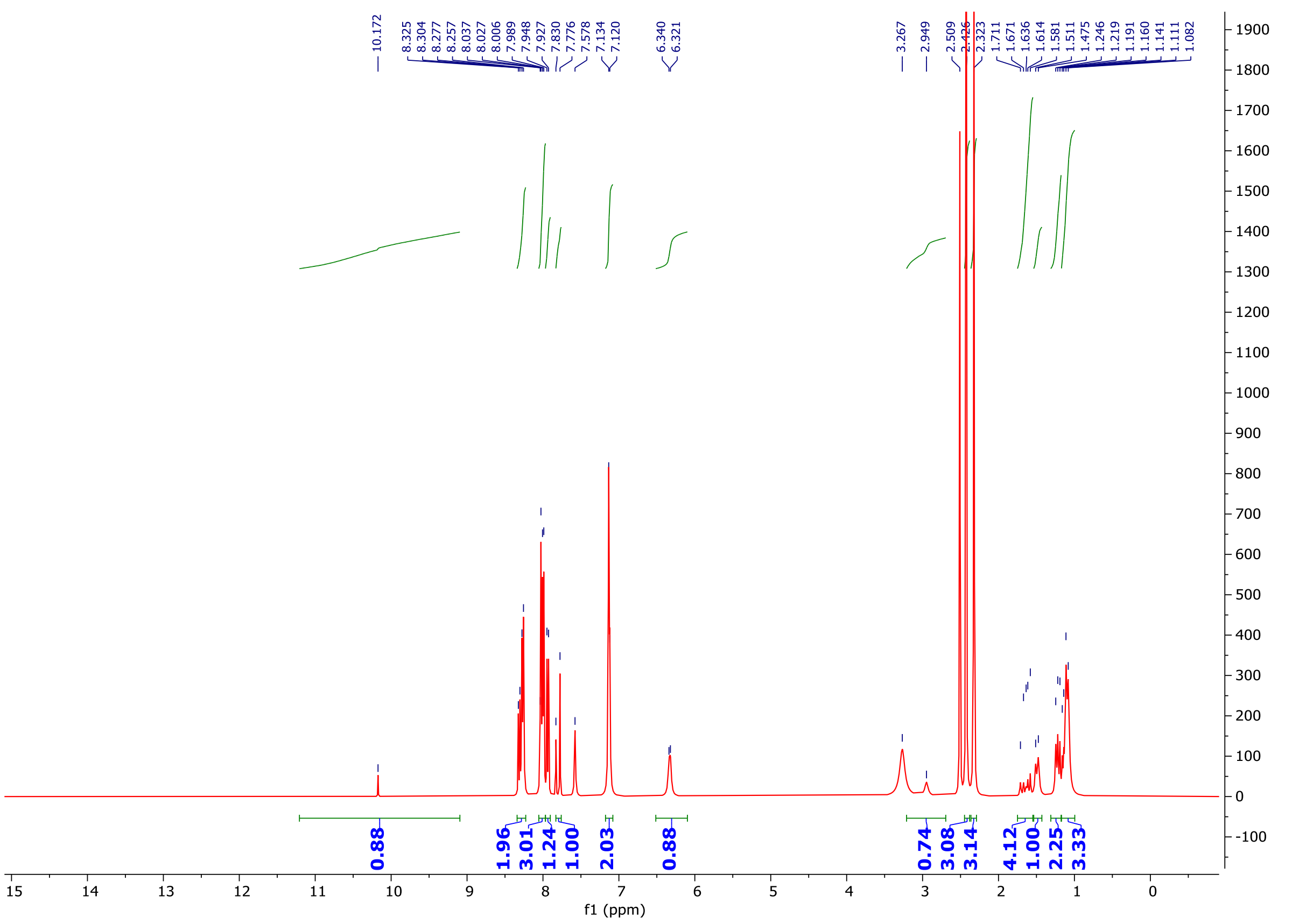

Figure S20. ^{1}H NMR spectrum of compound **22**

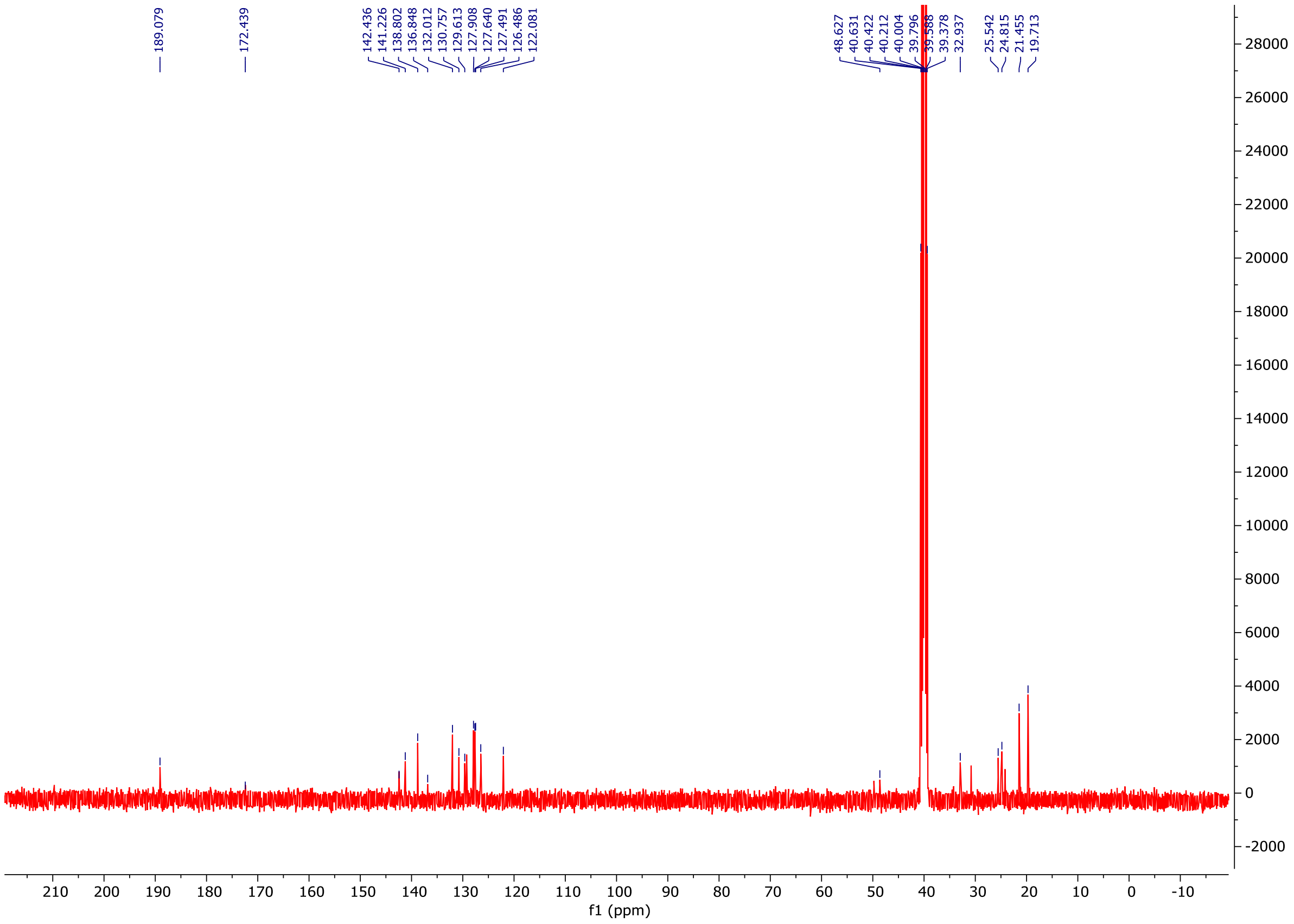

Figure S21. ^{13}C NMR spectrum of compound **22**

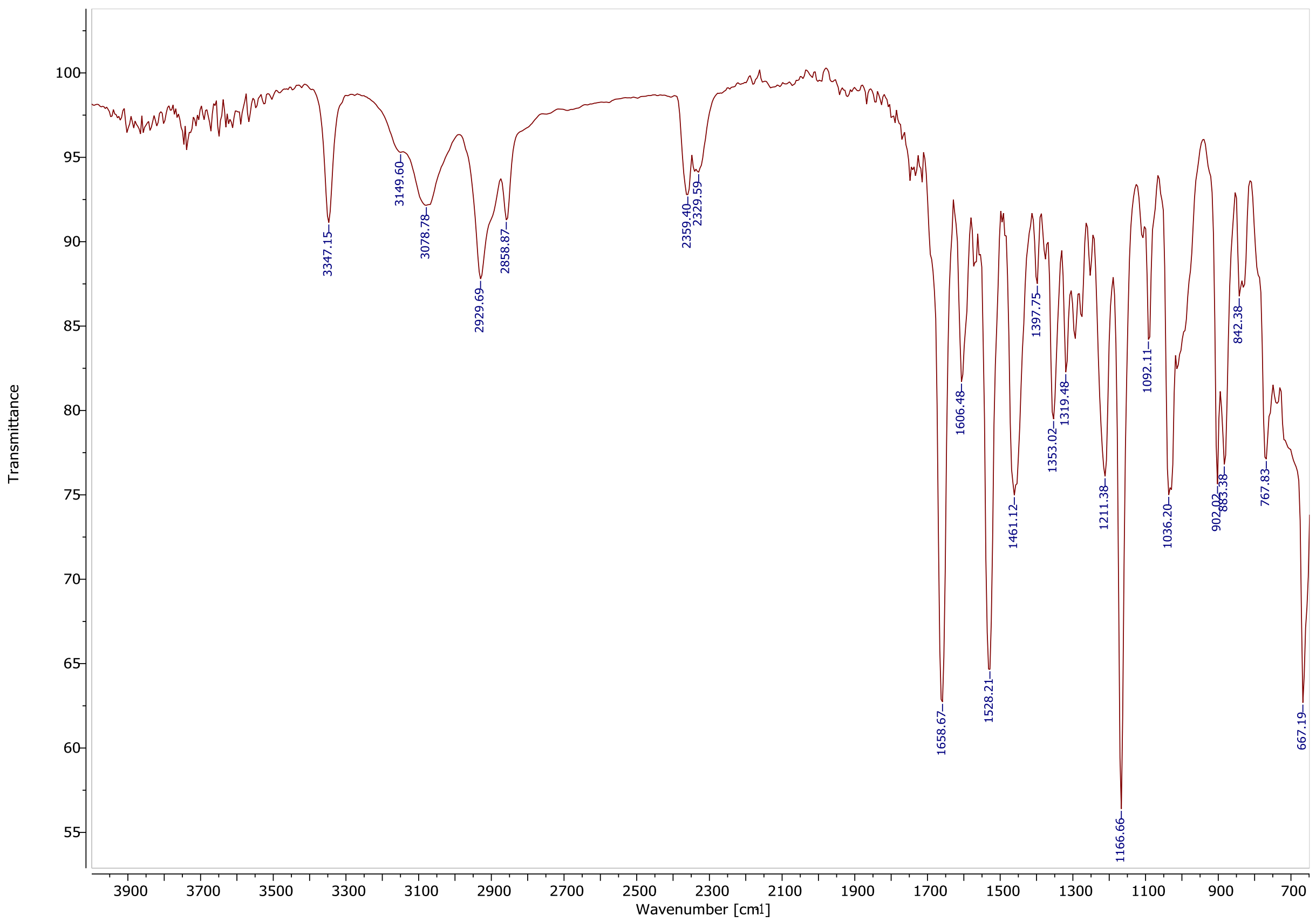

Figure S22. FTIR spectrum of compound 2**3**

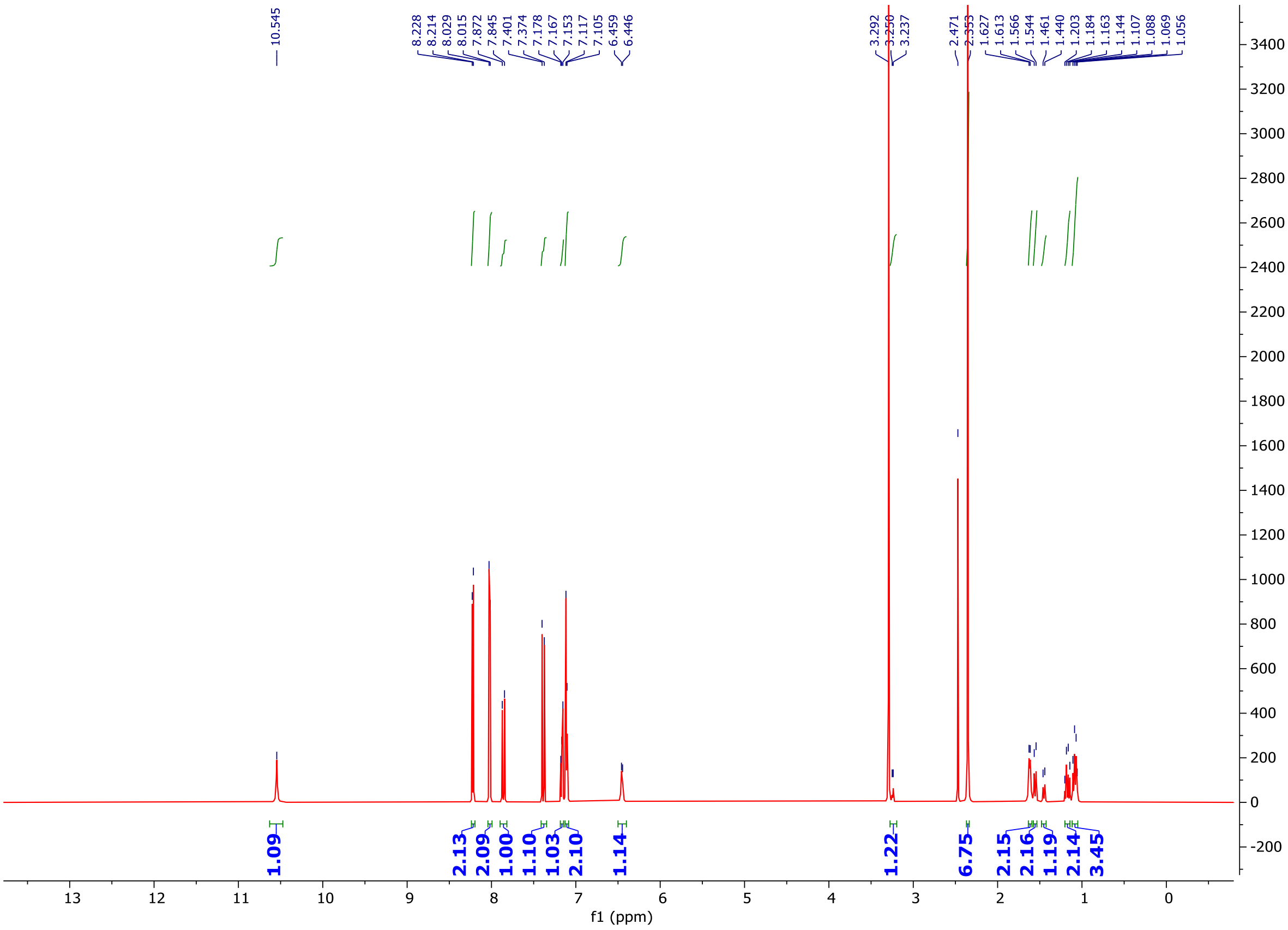

Figure S23. ^{1}H NMR spectrum of compound 2**3**

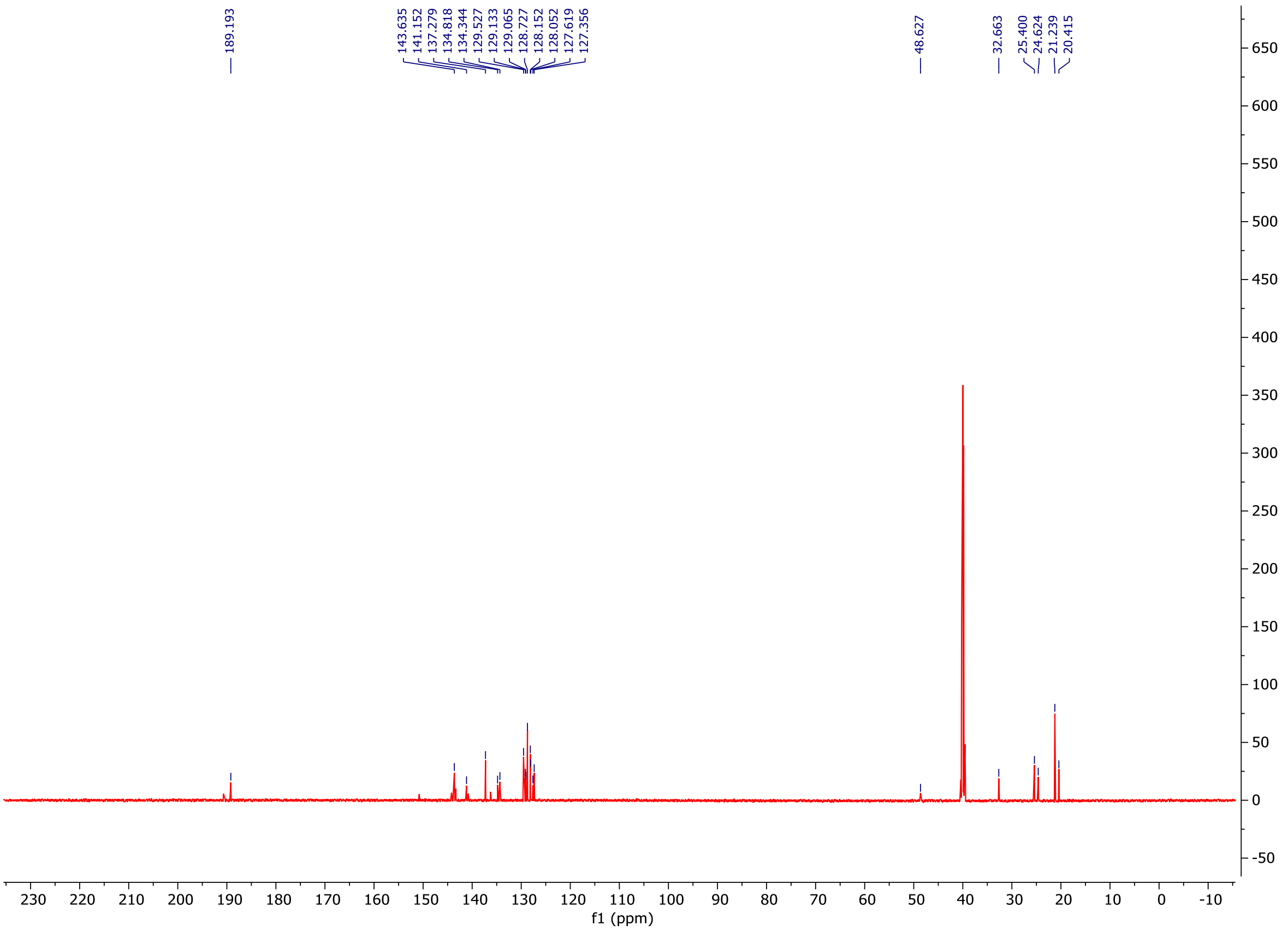

Figure S24 ^{13}C NMR spectrum of compound 2**3**

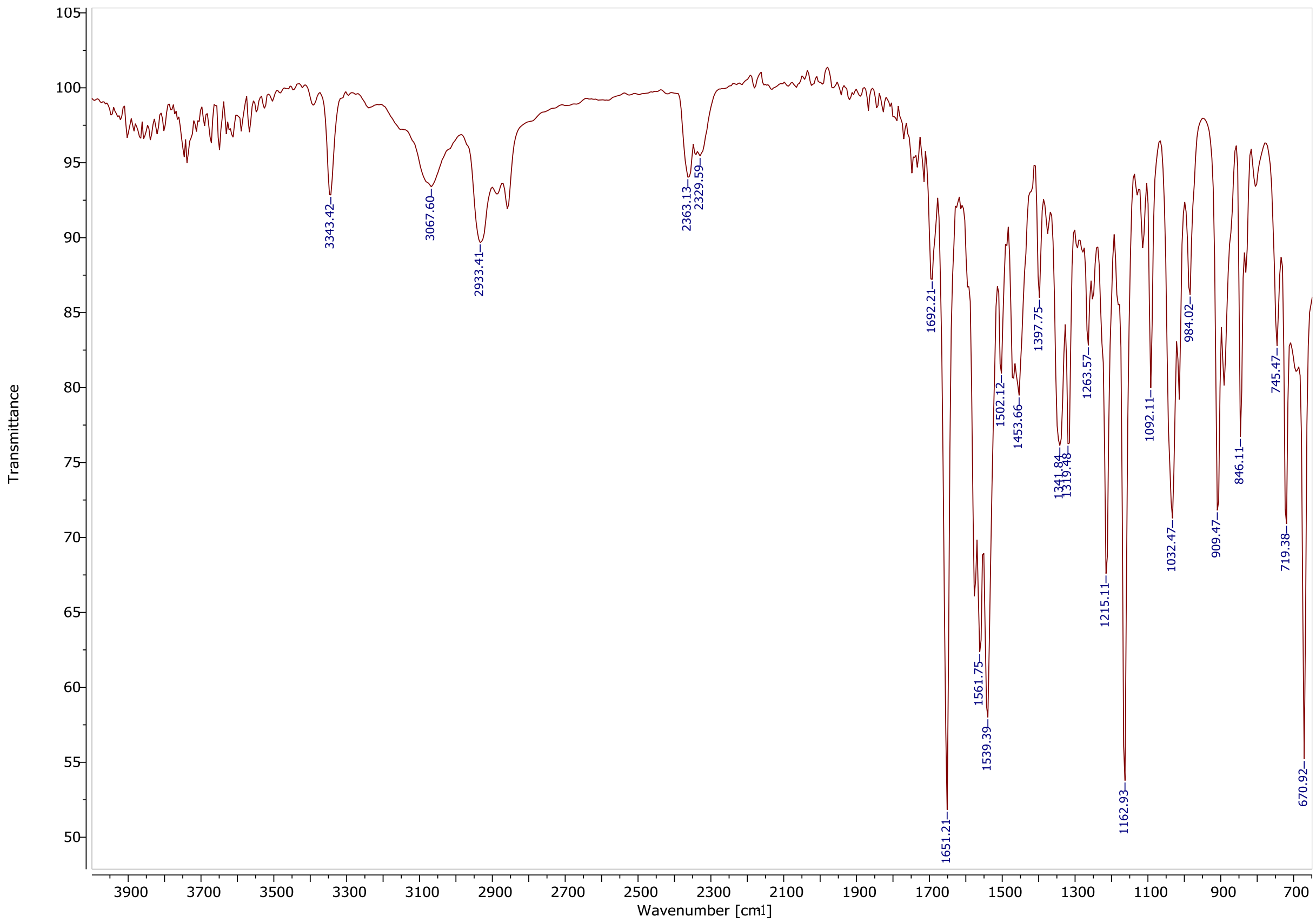

Figure S25. FTIR spectrum of compound **24**

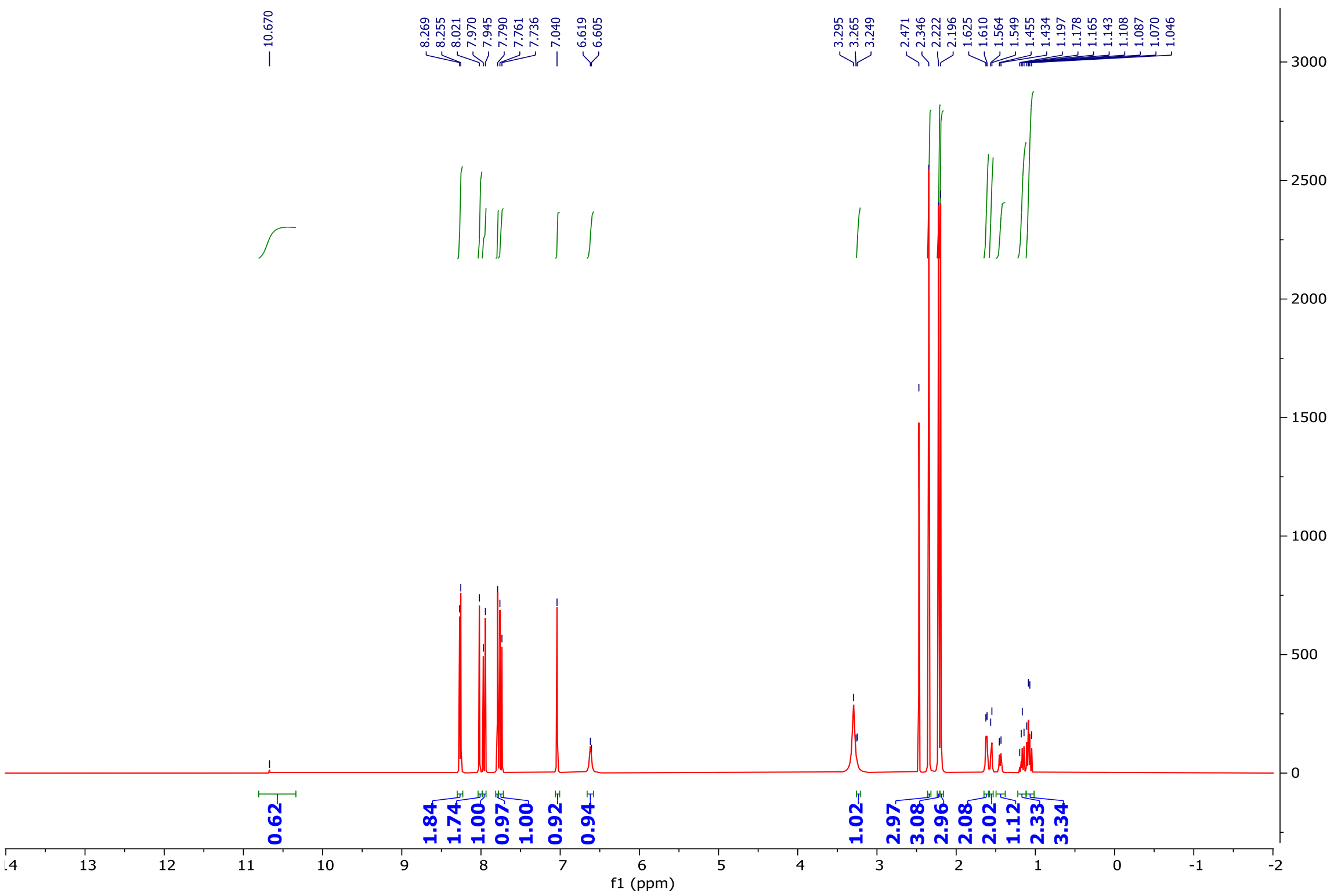

Figure S26. ^{1}H NMR spectrum of compound **24**

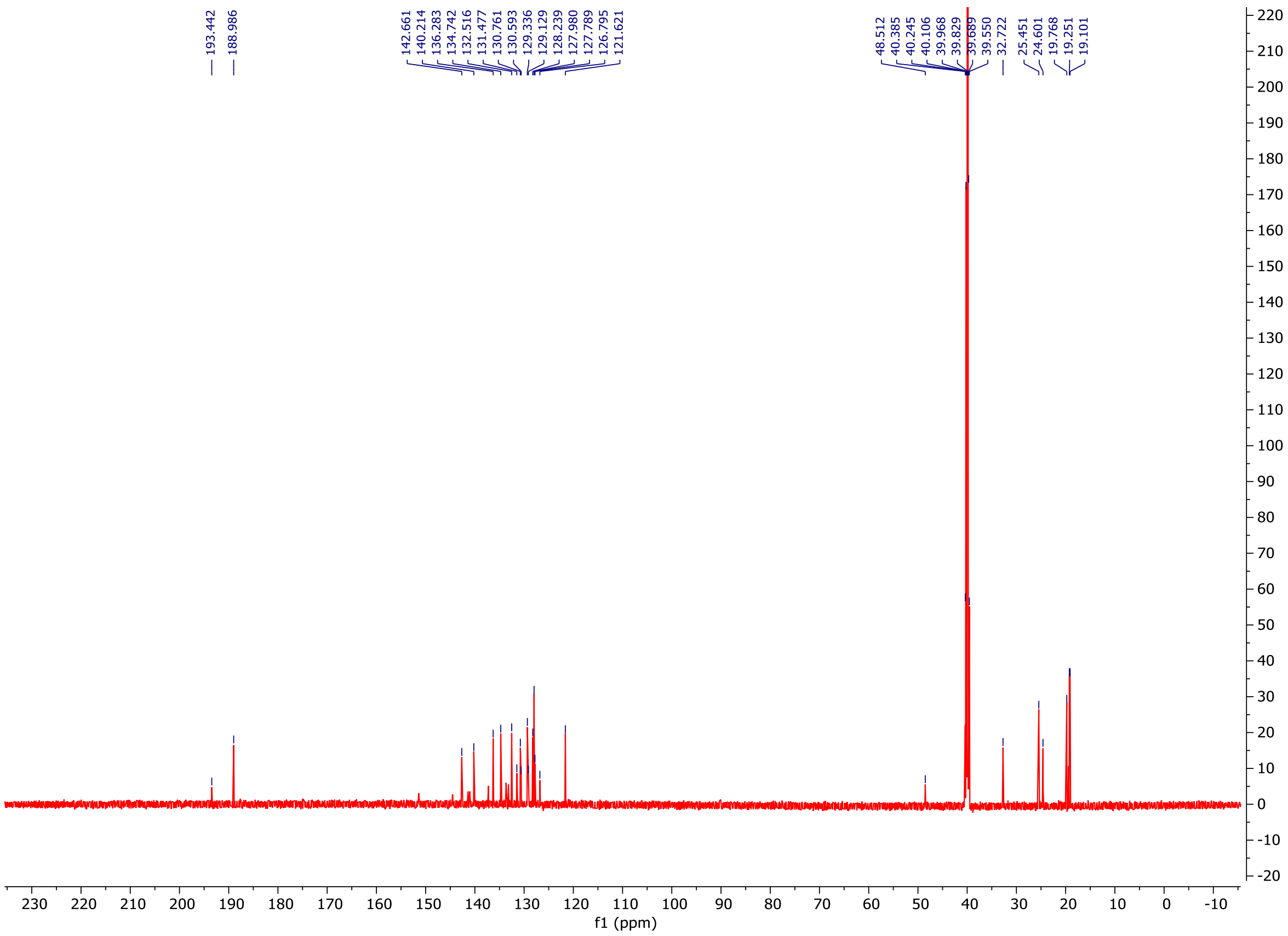

Figure S27. ^{13}C NMR spectrum of compound **24**

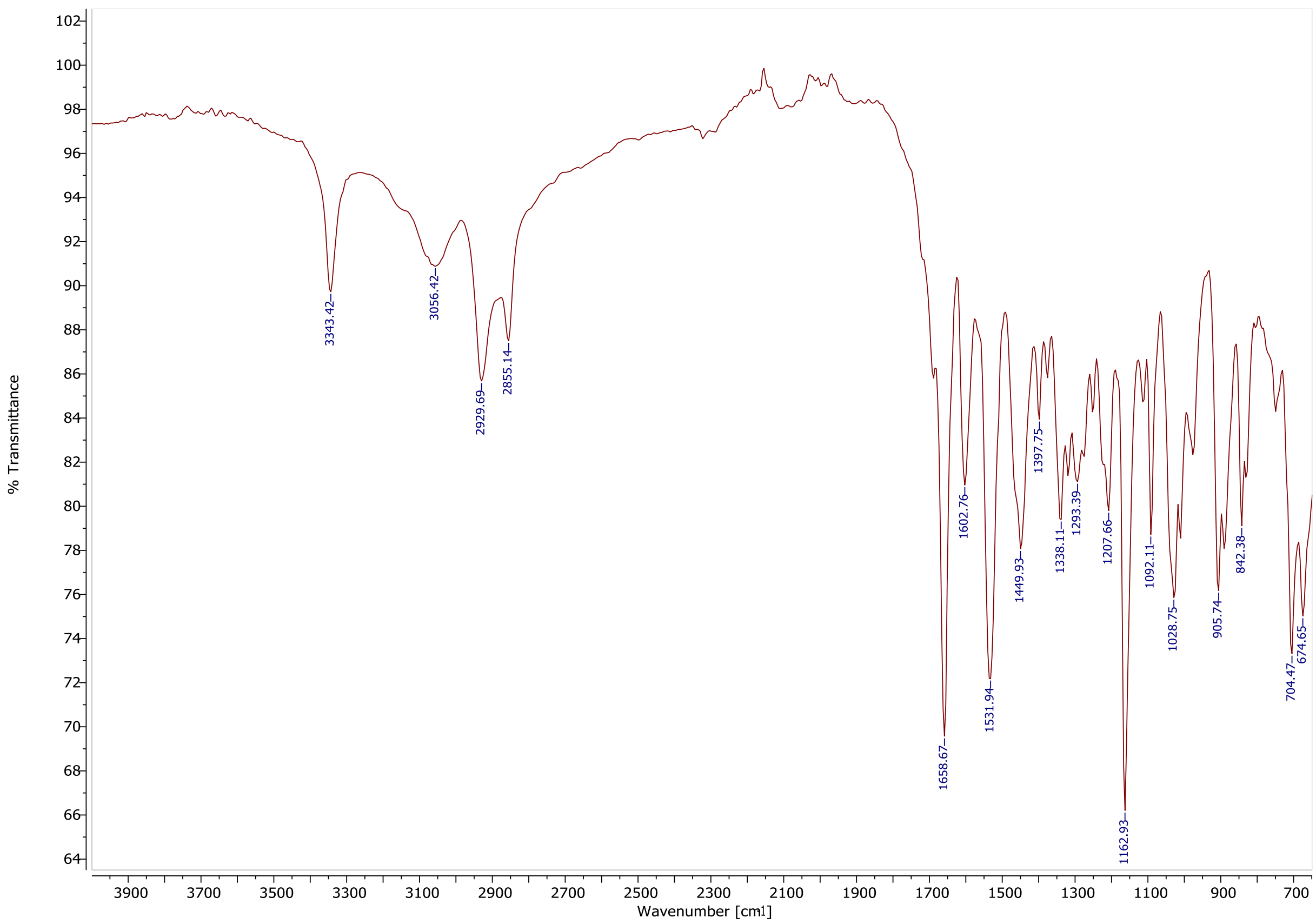

Figure S28. FTIR spectrum of compound **25**

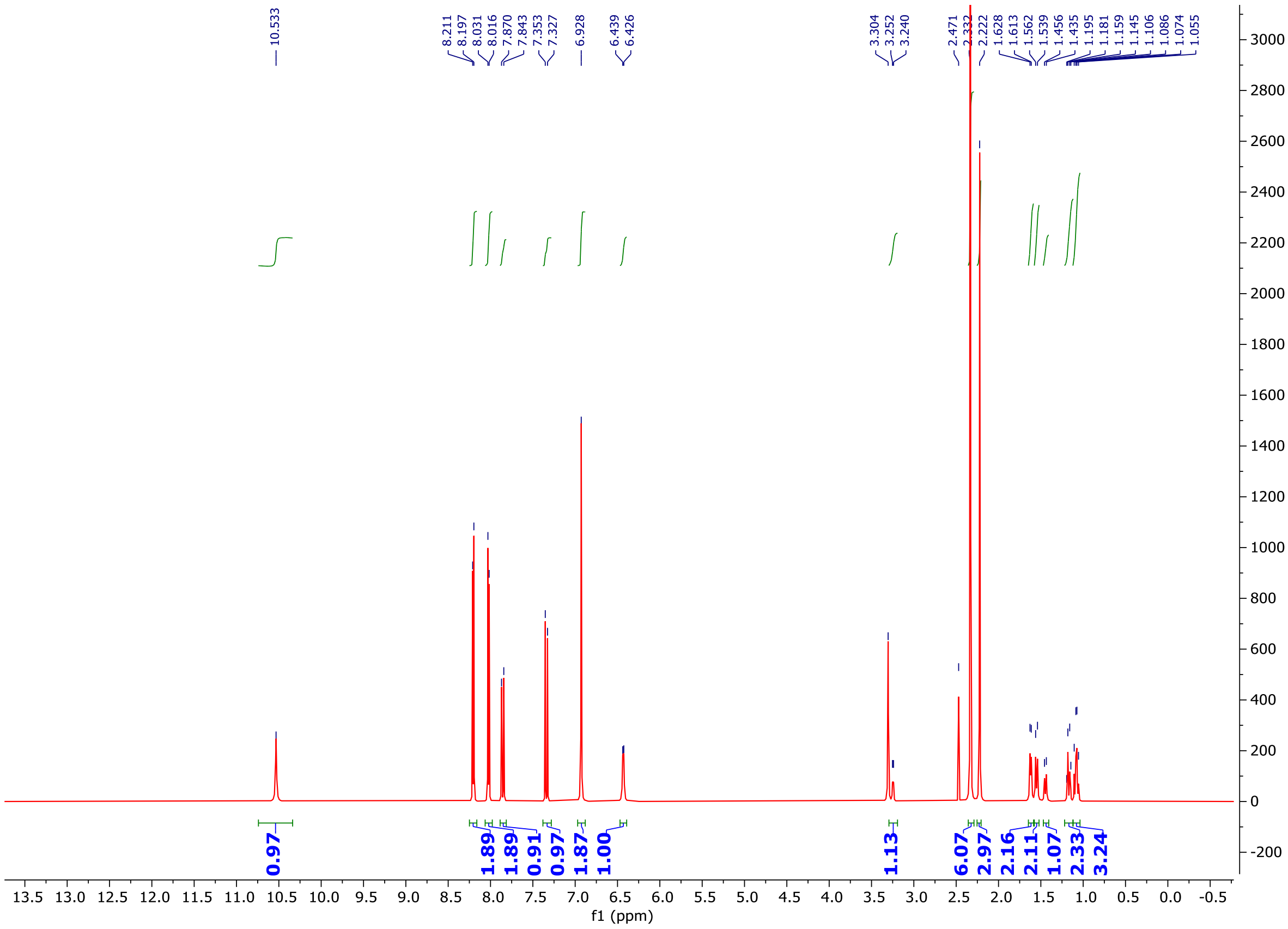

Figure S29. ^{1}H NMR spectrum of compound **25**

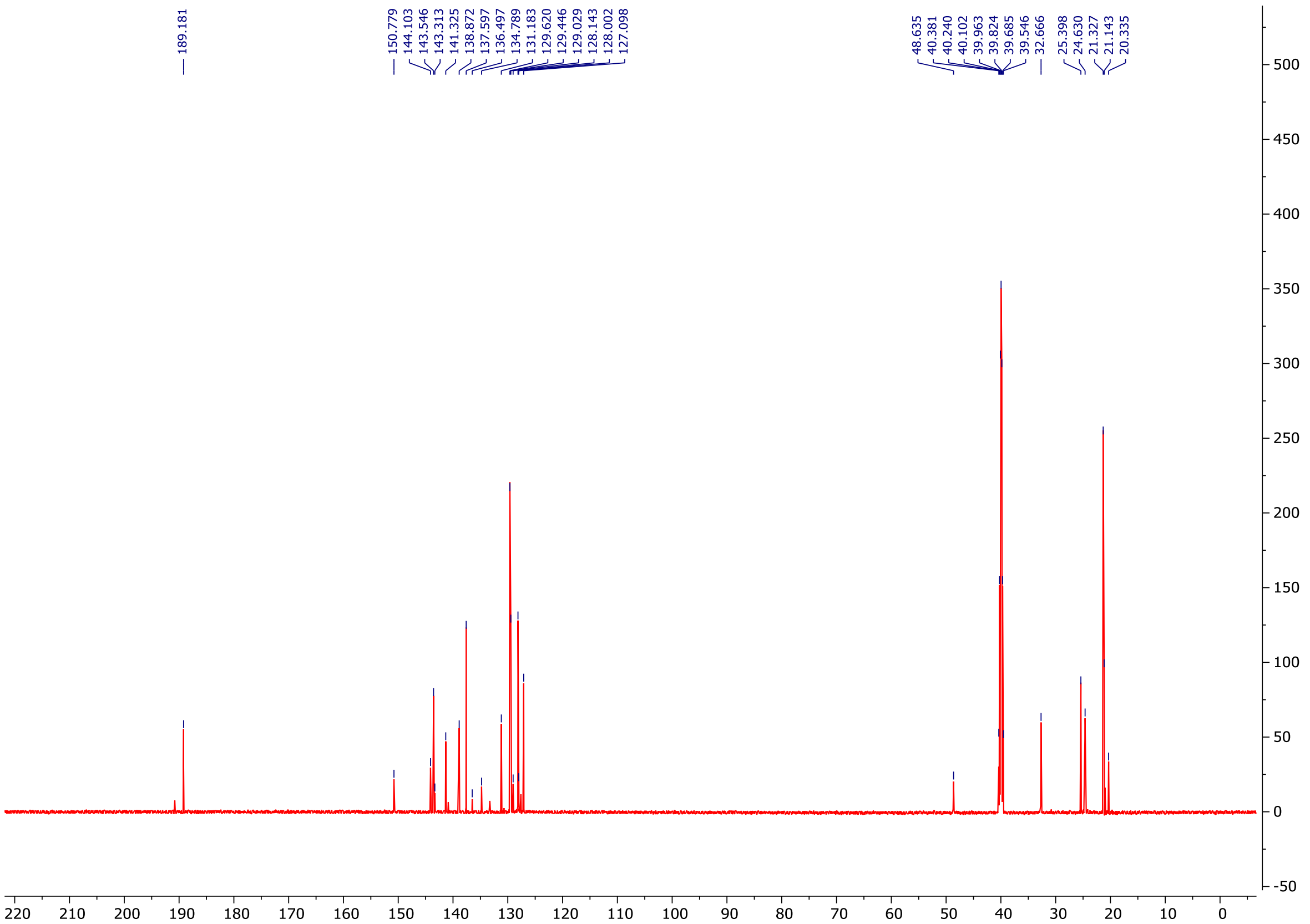

Figure S30. ^{13}C NMR spectrum of compound **25**

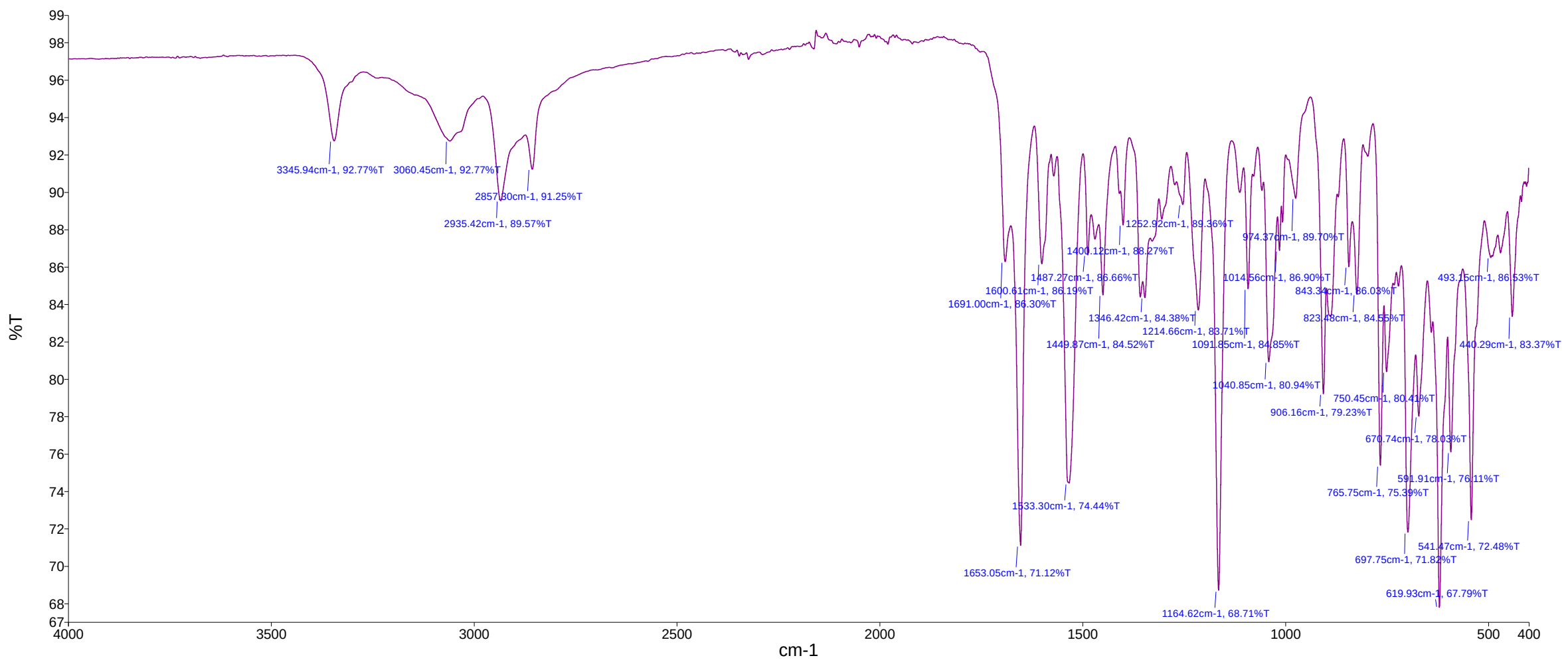

Figure S31. FTIR spectrum of compound **26**

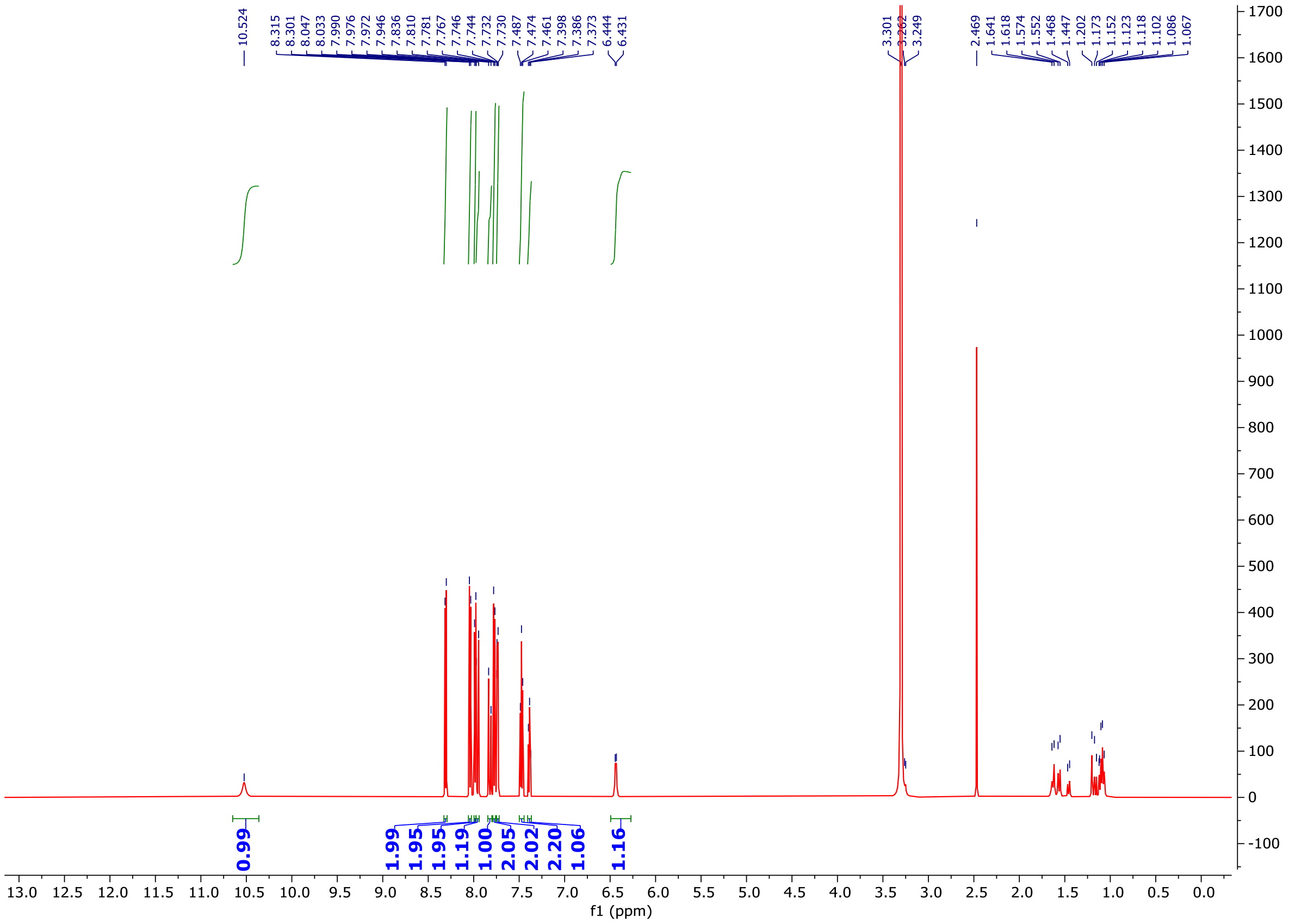

Figure S32. ^{1}H NMR spectrum of compound **26**

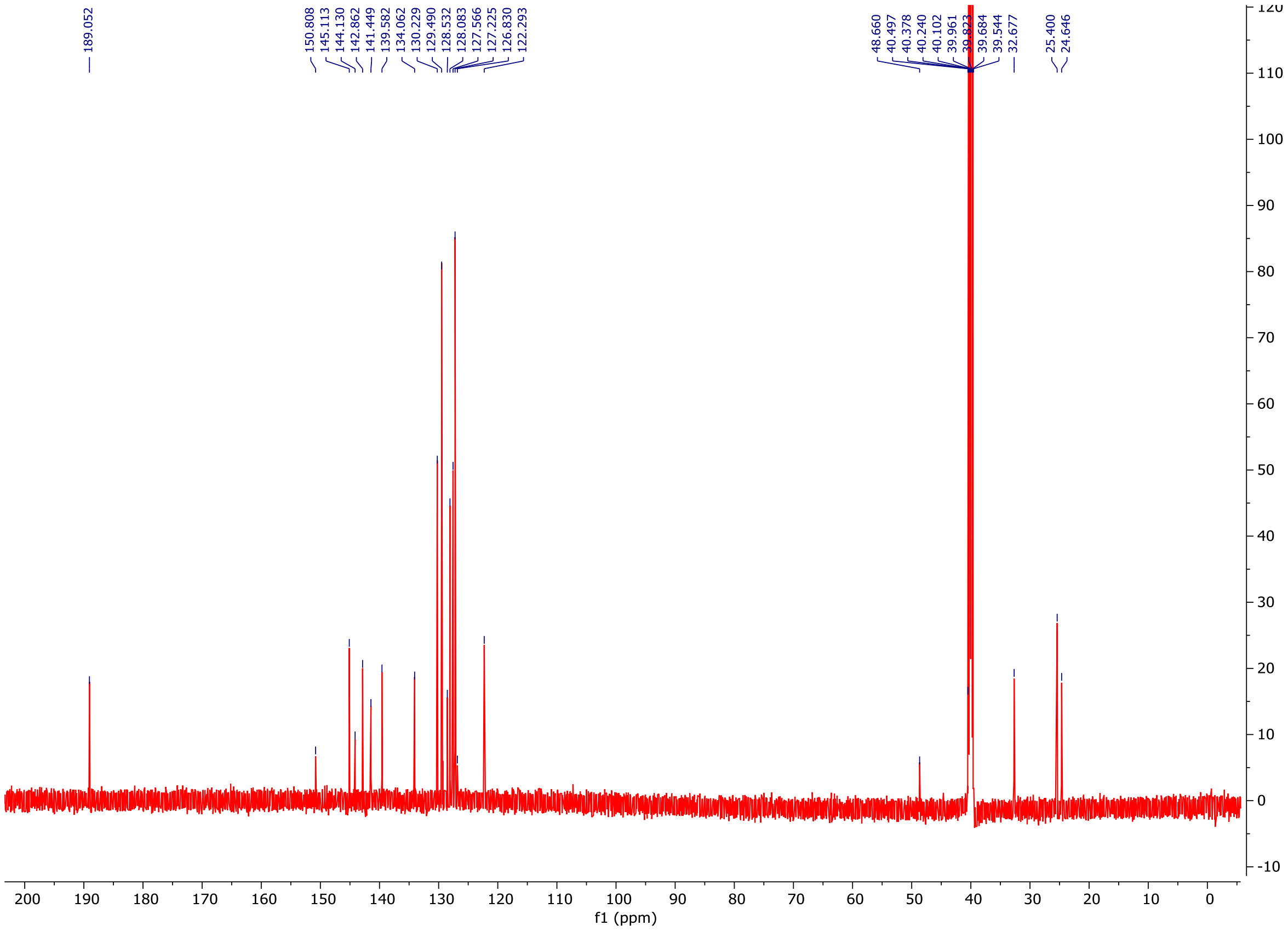

Figure S33. ^{13}C NMR spectrum of compound **26**

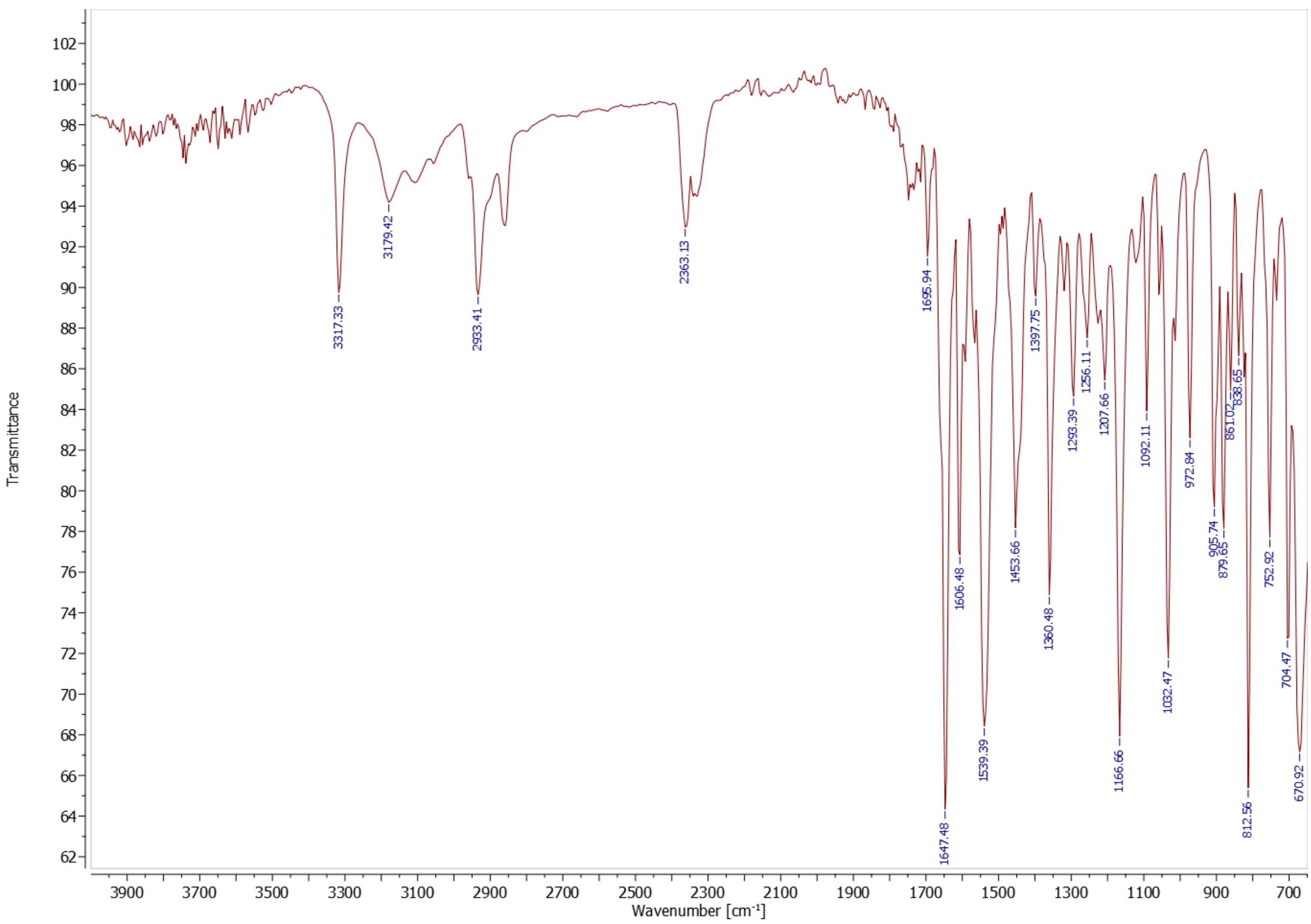

Figure S34. FTIR spectrum of compound **28**

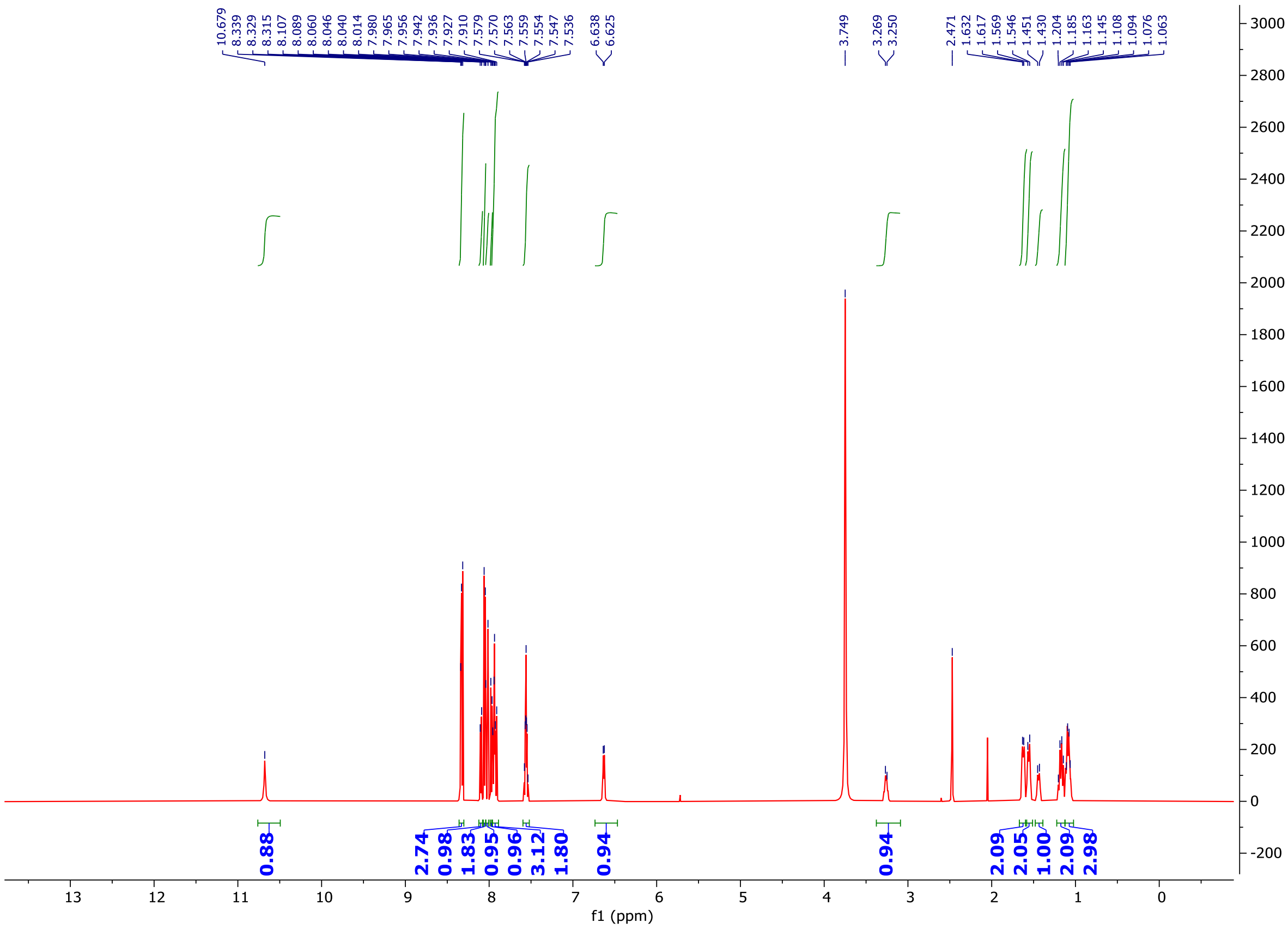

Figure S35. ^{1}H NMR spectrum of compound **28**

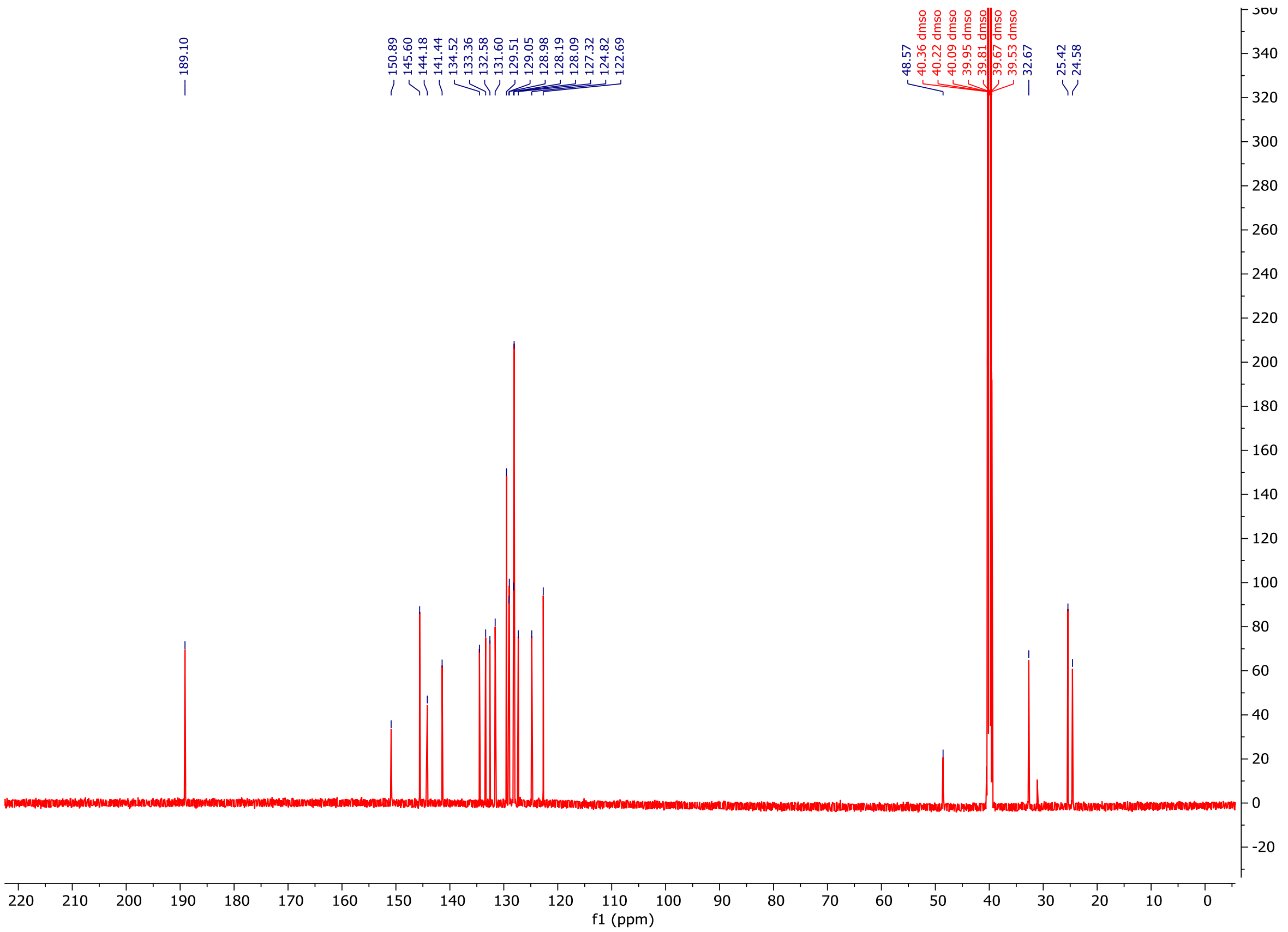

Figure S36. ^{13}C NMR spectrum of compound **28**

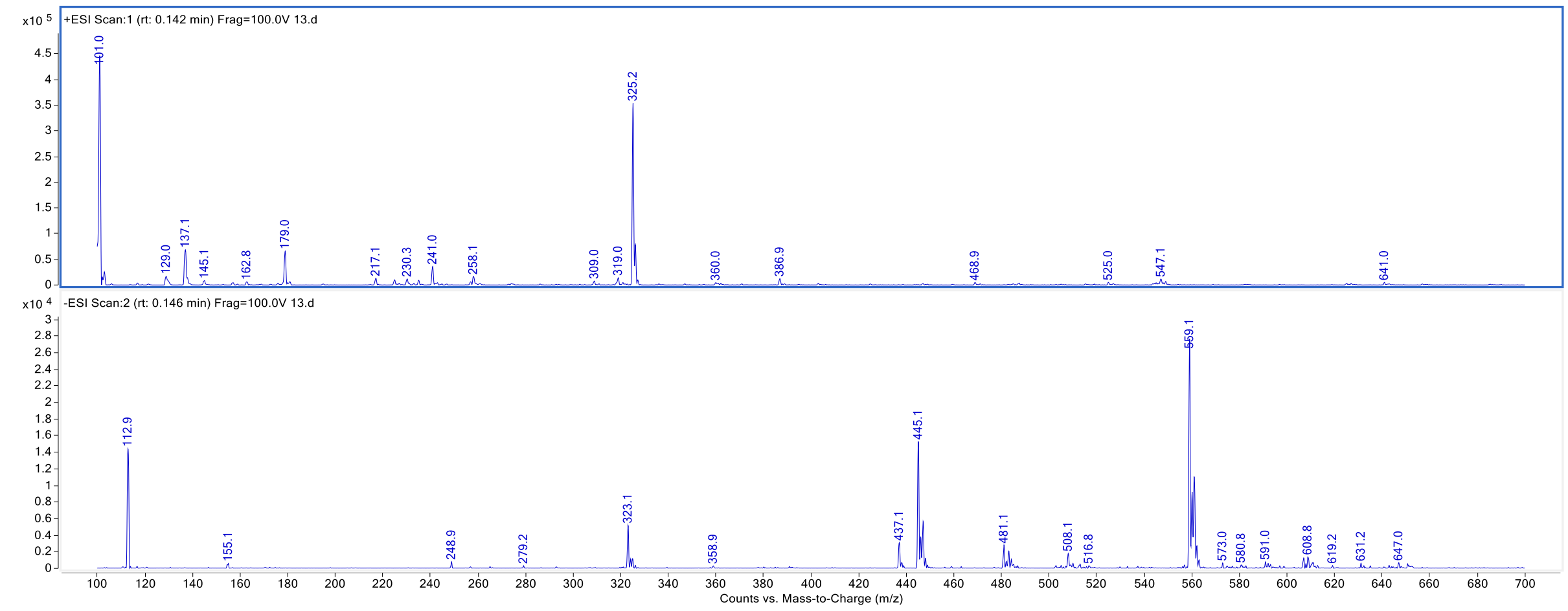

Figure S37. Mass spectrum of compound **28**

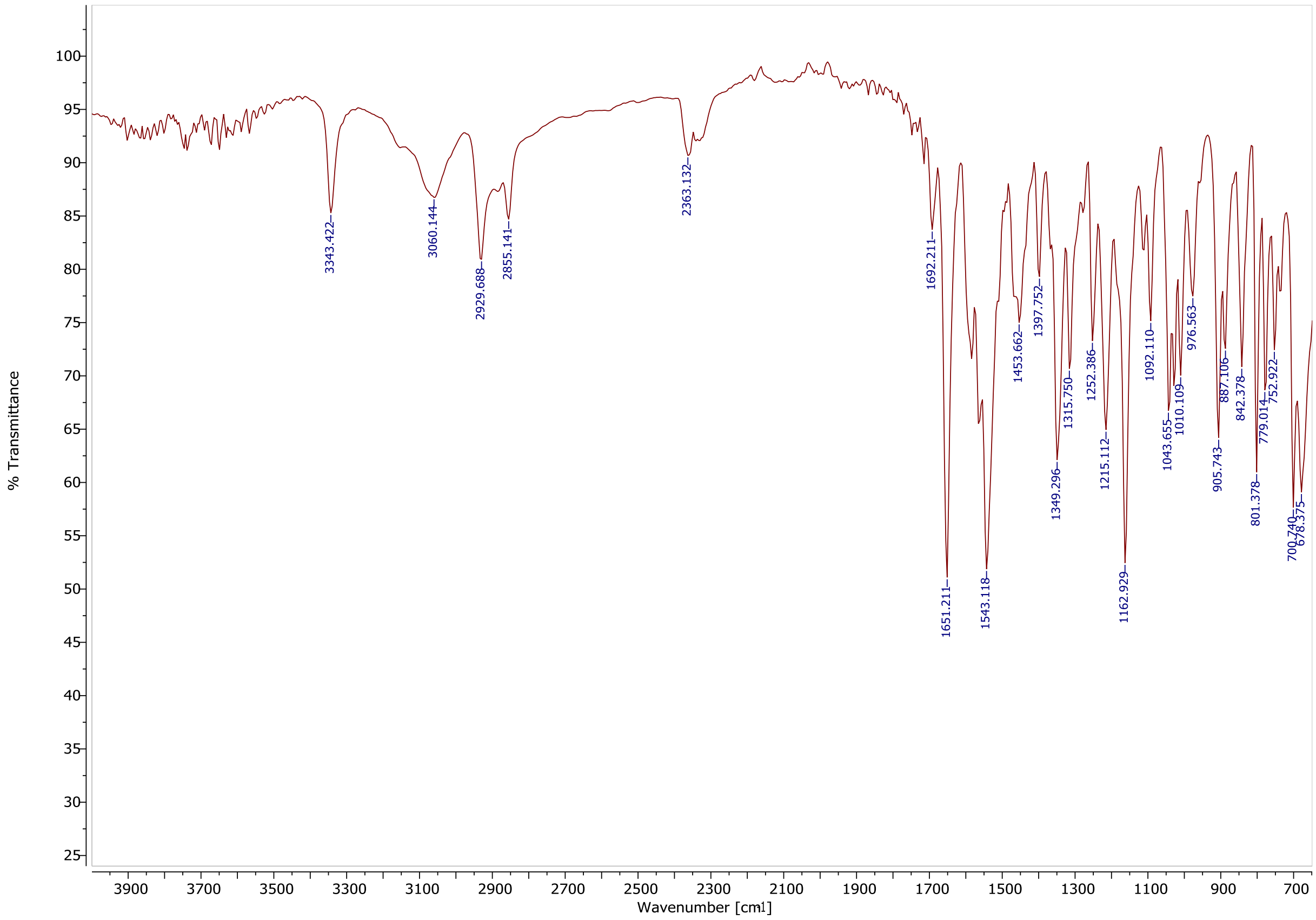

Figure S38. FTIR spectrum of compound **28A**

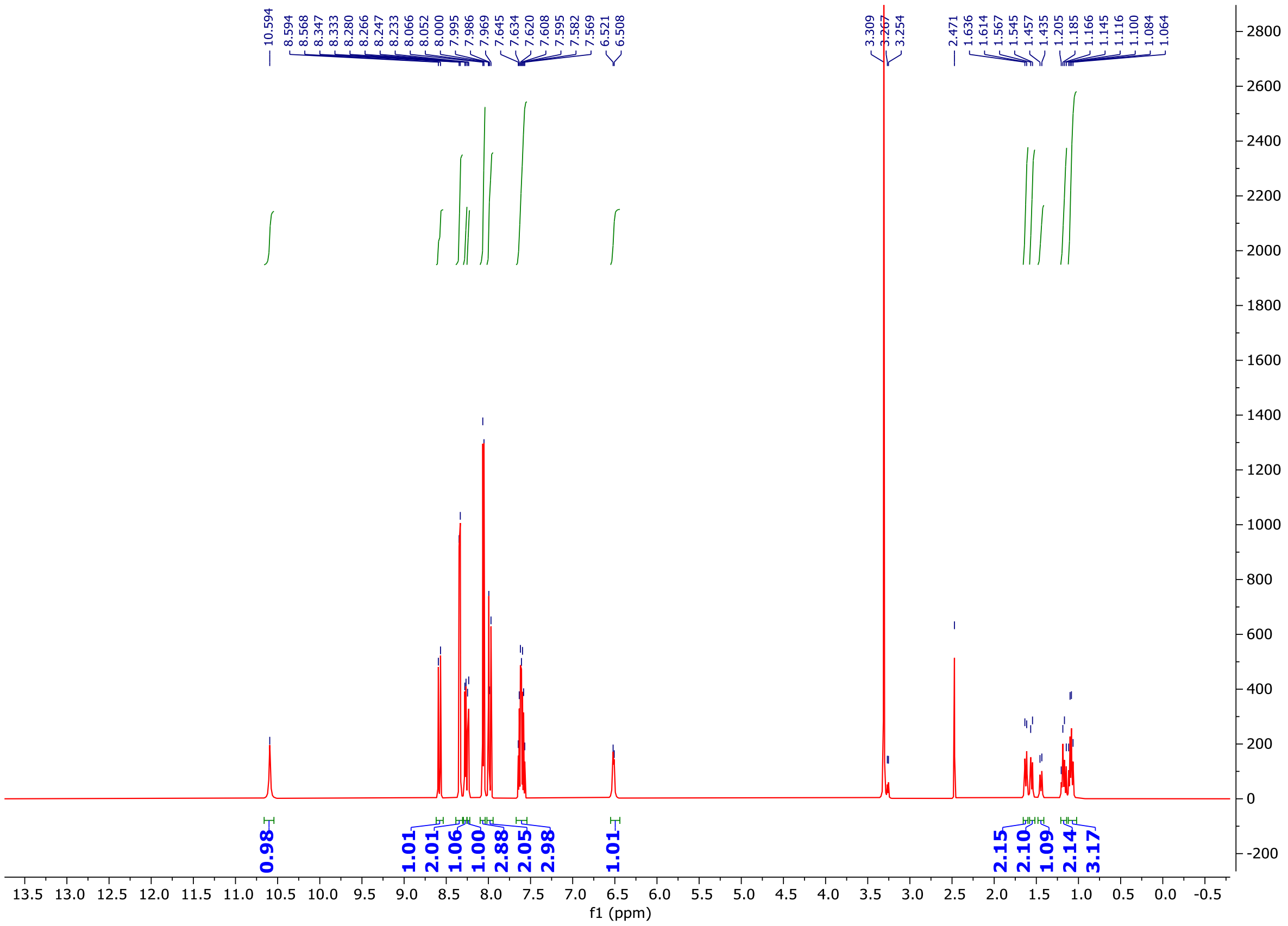

Figure S39. ^{1}H NMR spectrum of compound **28A**

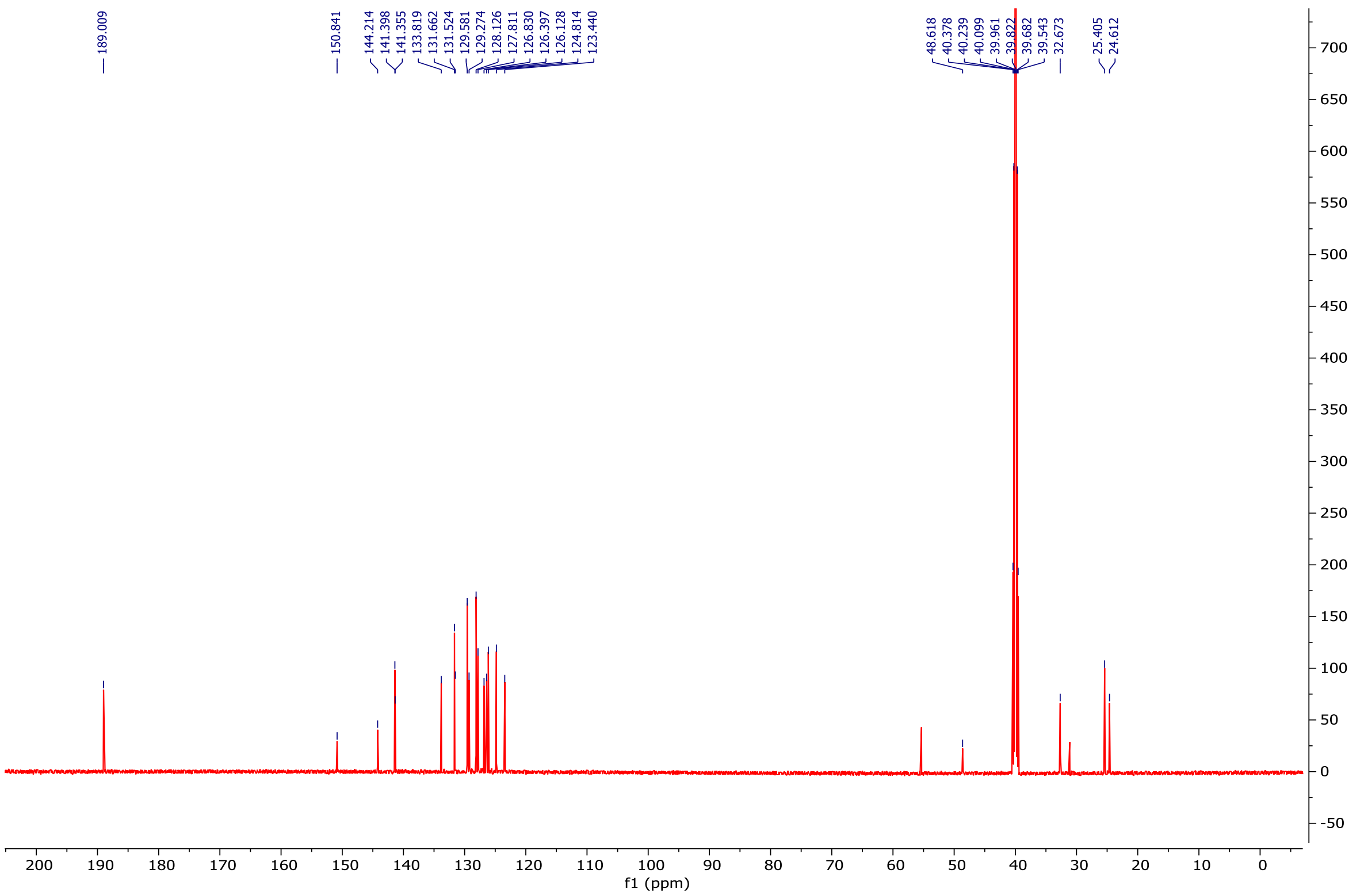

Figure S40. ^{13}C NMR spectrum of compound **28A**

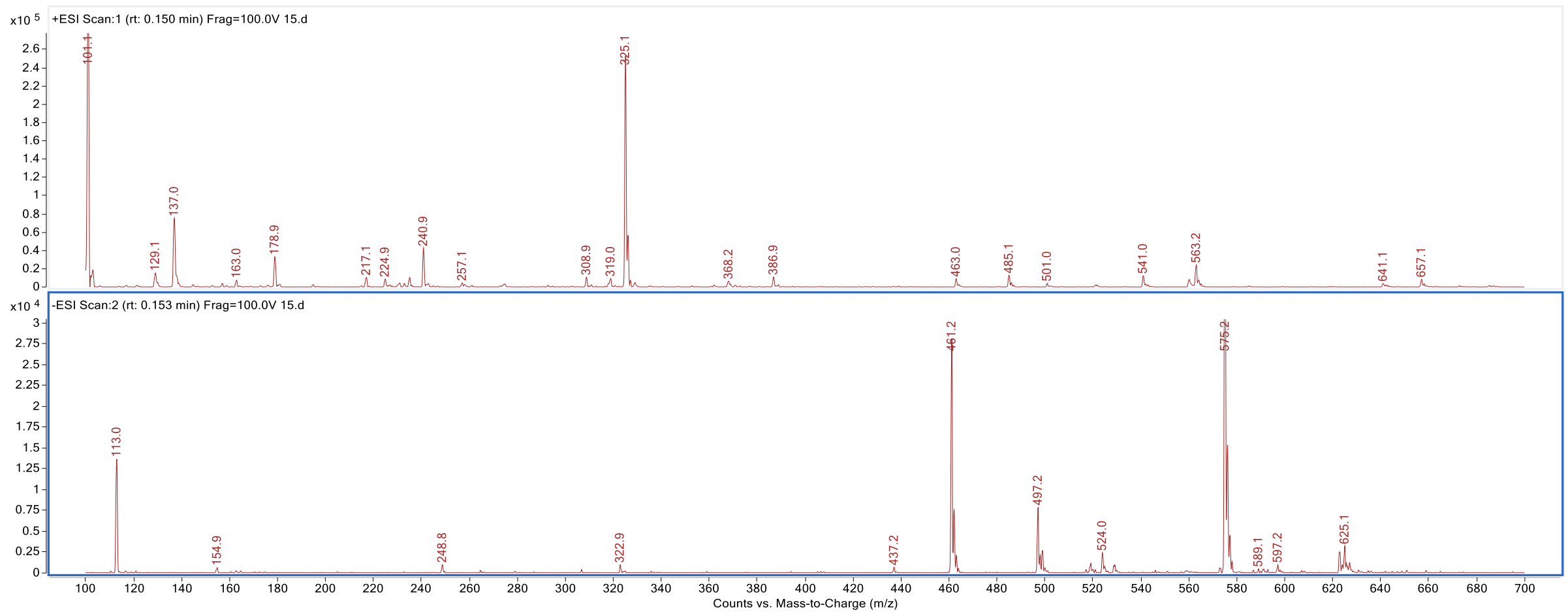

Figure S41. Mass spectrum of compound **28A**

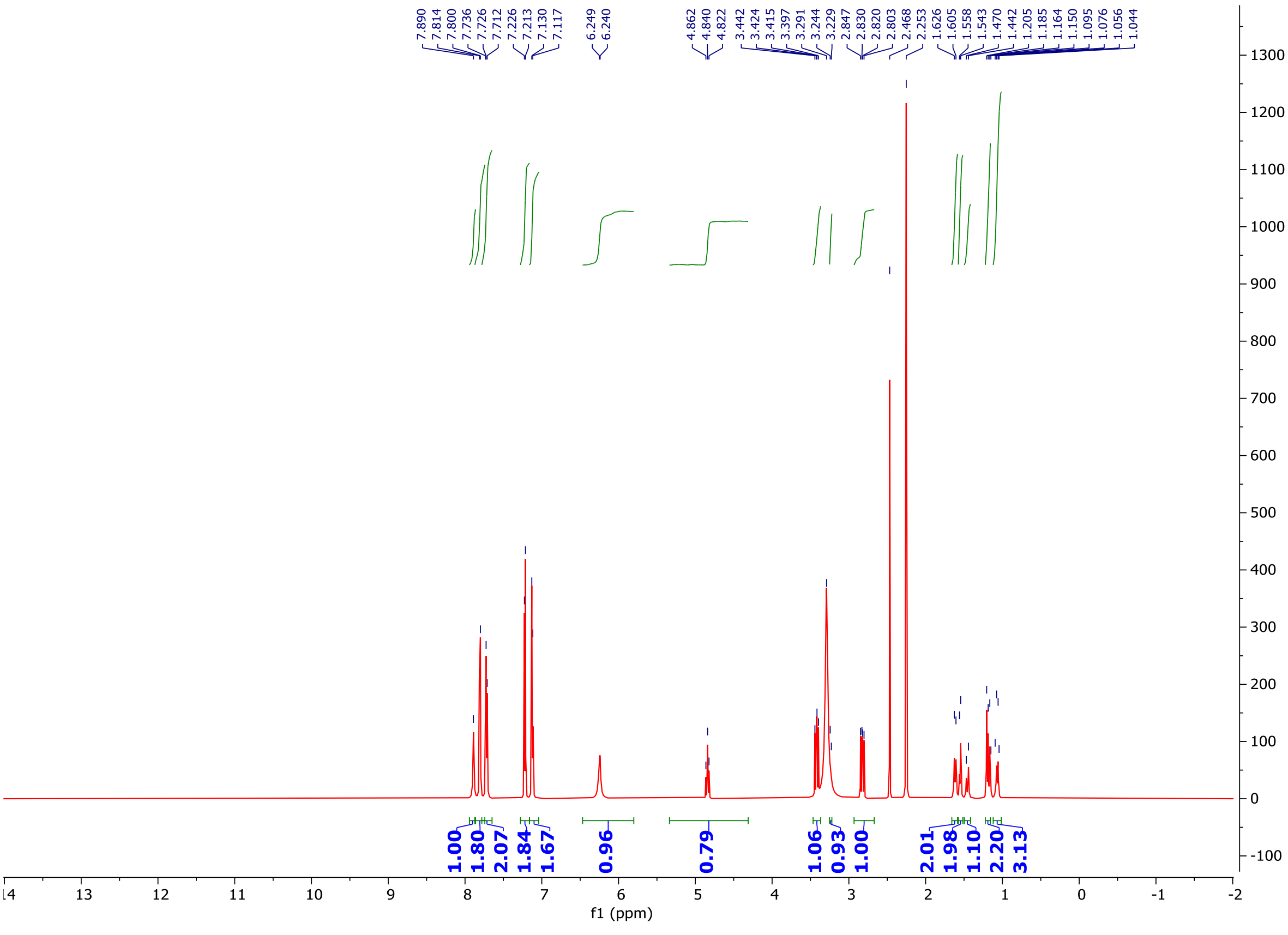

Figure S42. ^{1}H NMR spectrum of compound **44**

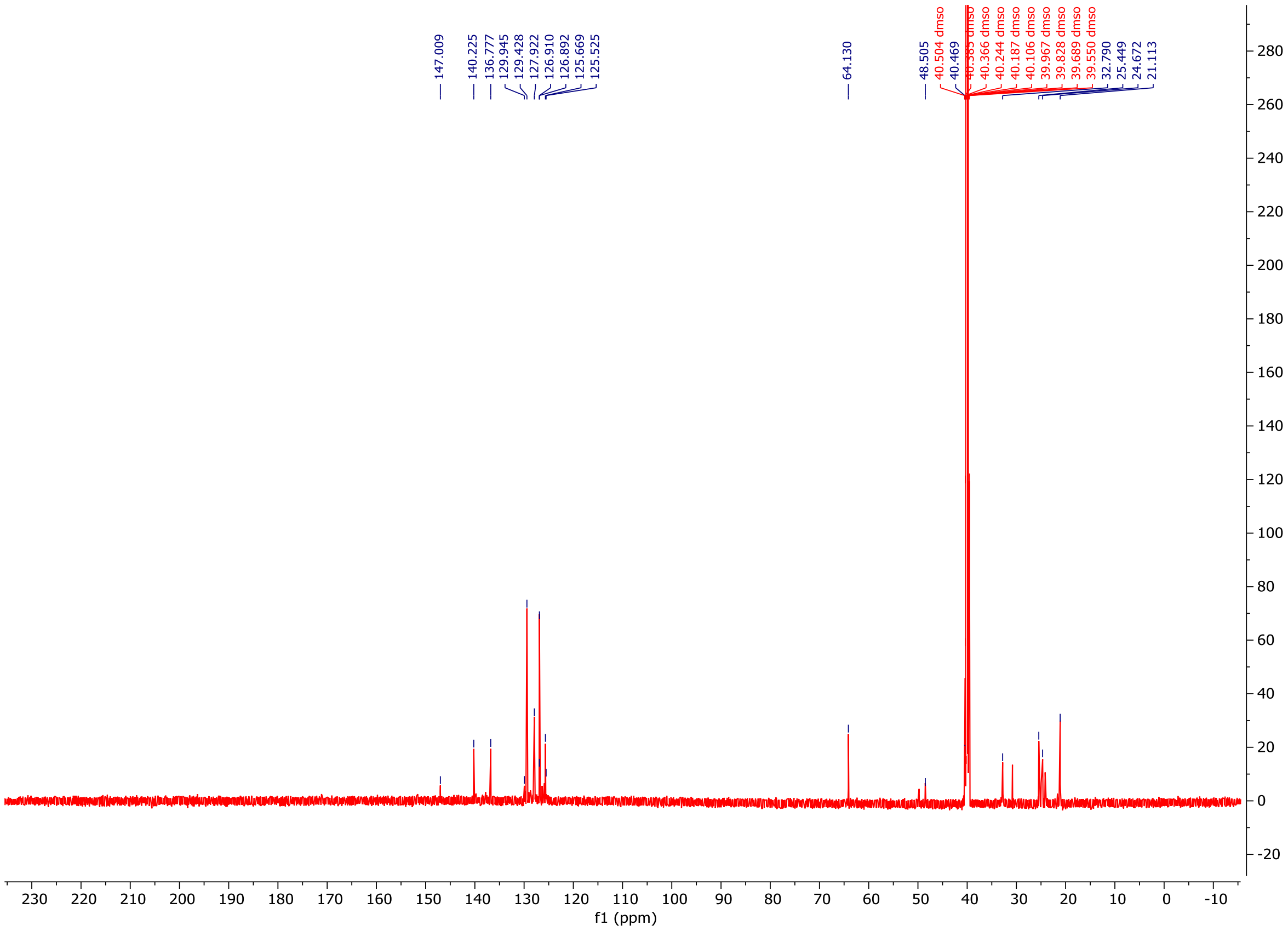

Figure S43. ^{13}C NMR spectrum of compound **44**

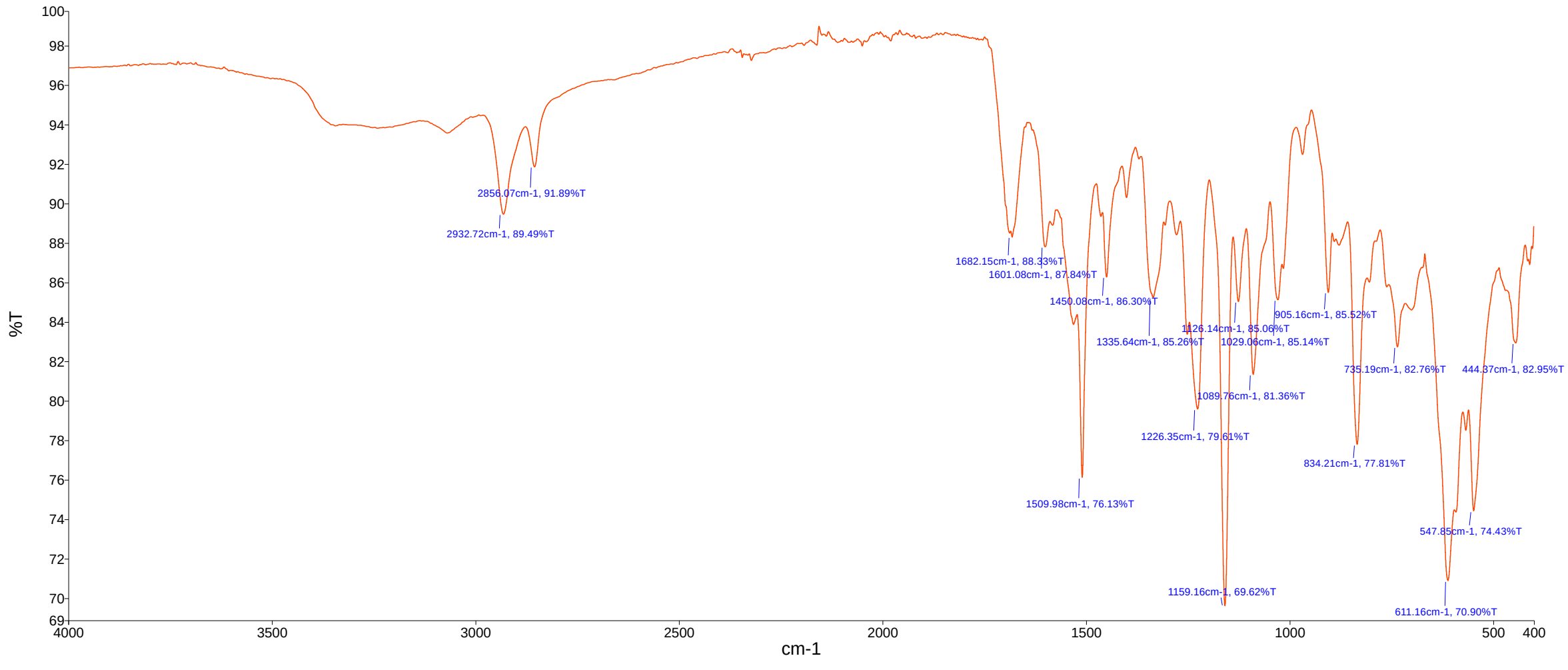

Figure S44. FTIR spectrum of compound **45**

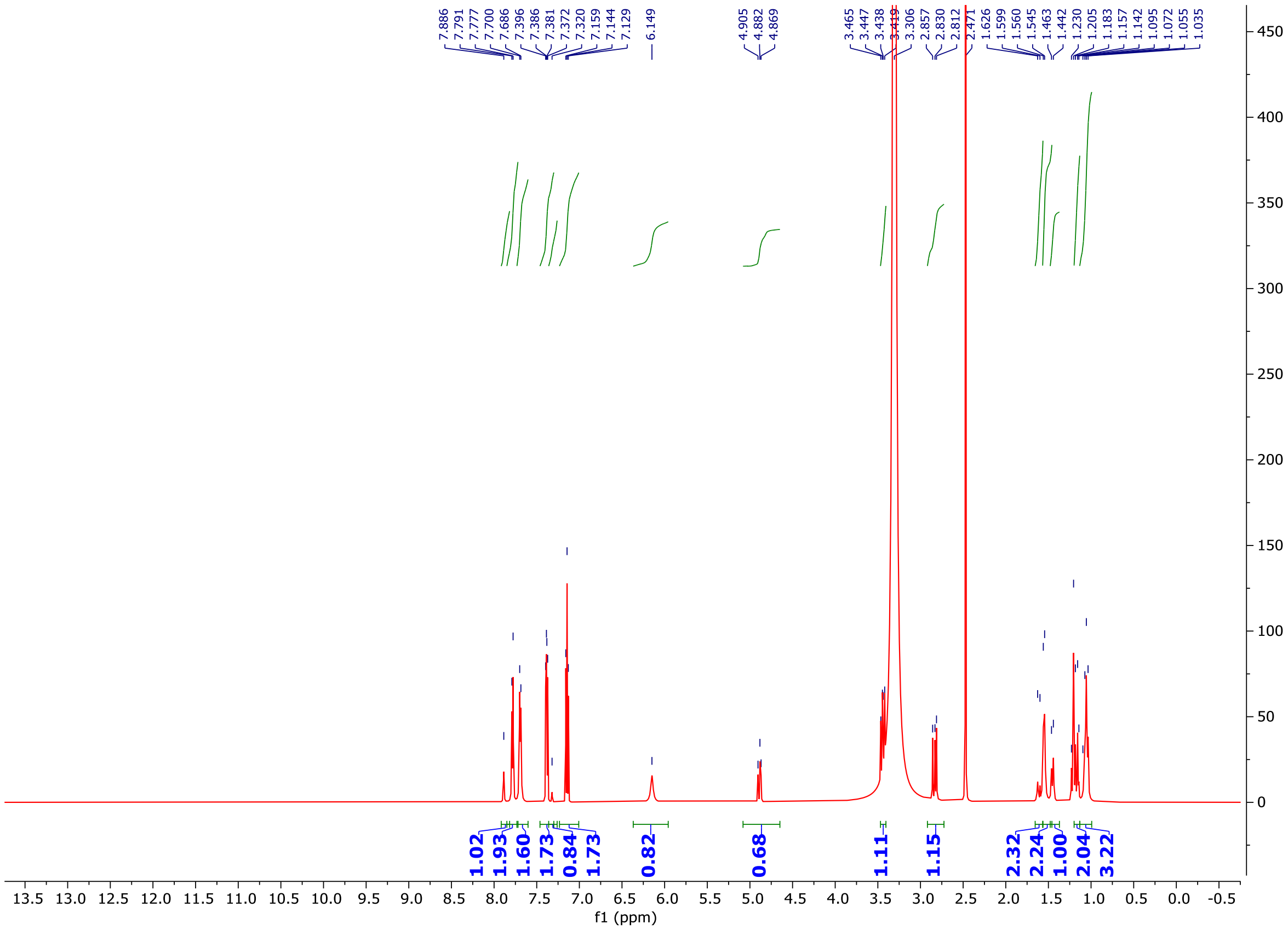

Figure S45. ^{1}H NMR spectrum of compound **45**

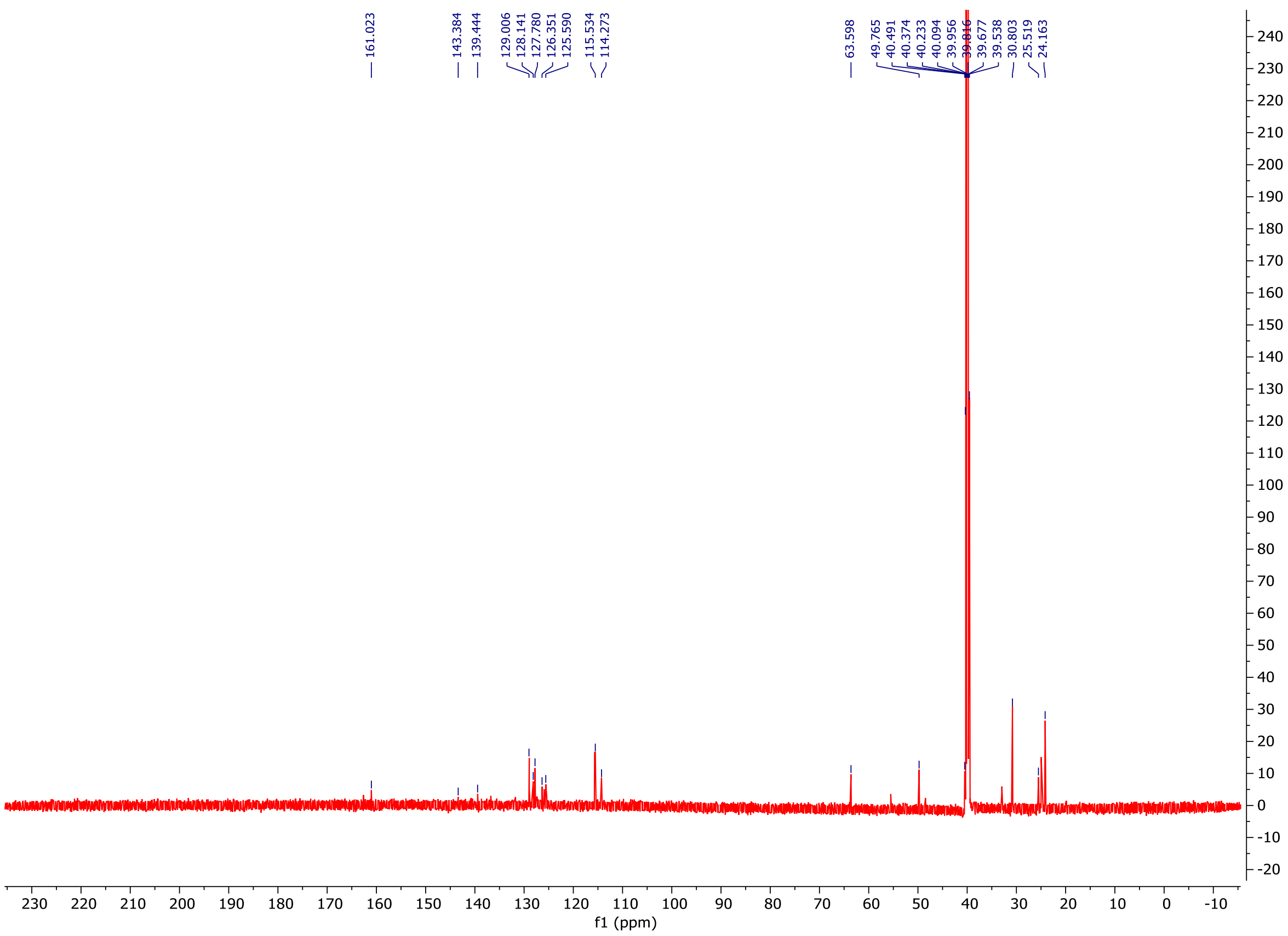

Figure S46. ^{13}C NMR spectrum of compound **45**

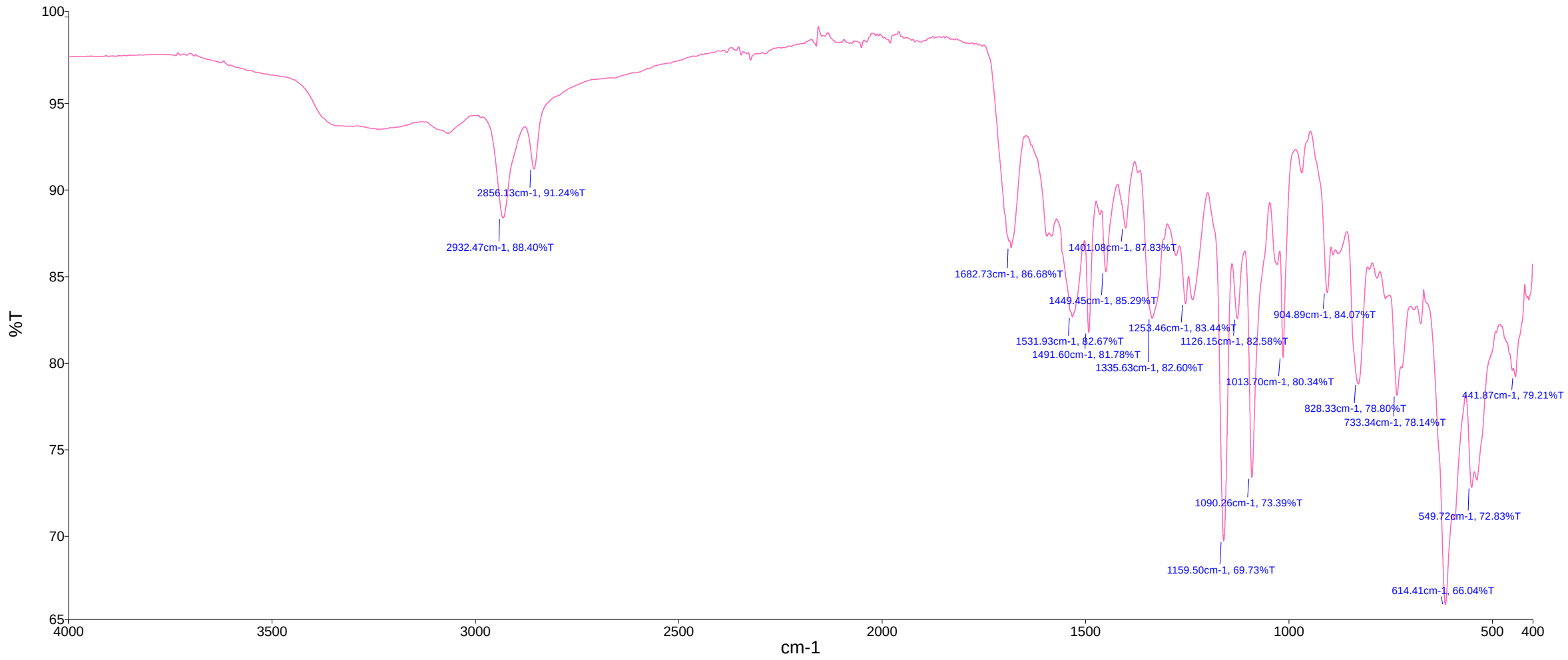

Figure S47. FTIR spectrum of compound **46**

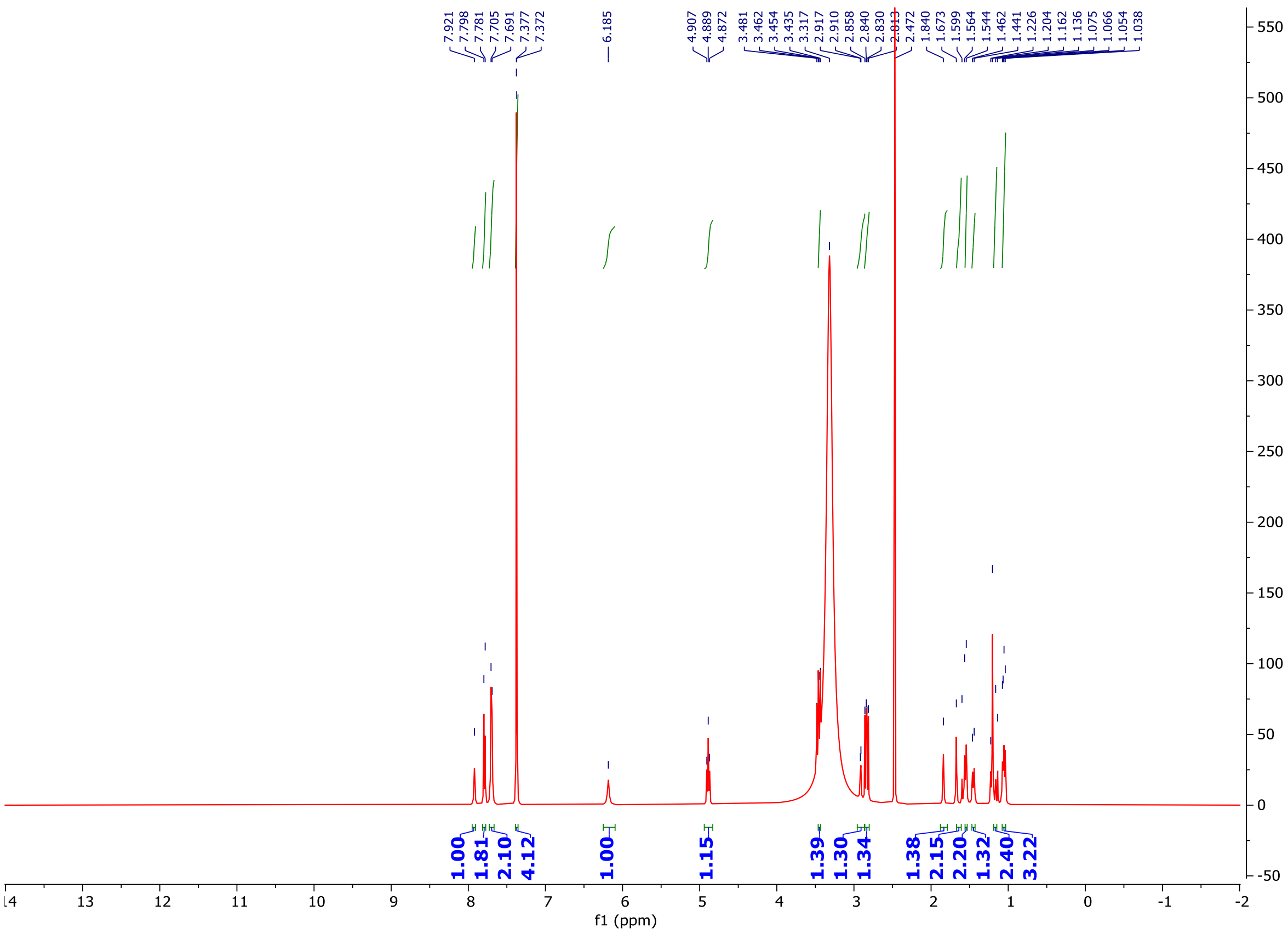

Figure S48. ^{1}H NMR spectrum of compound **46**

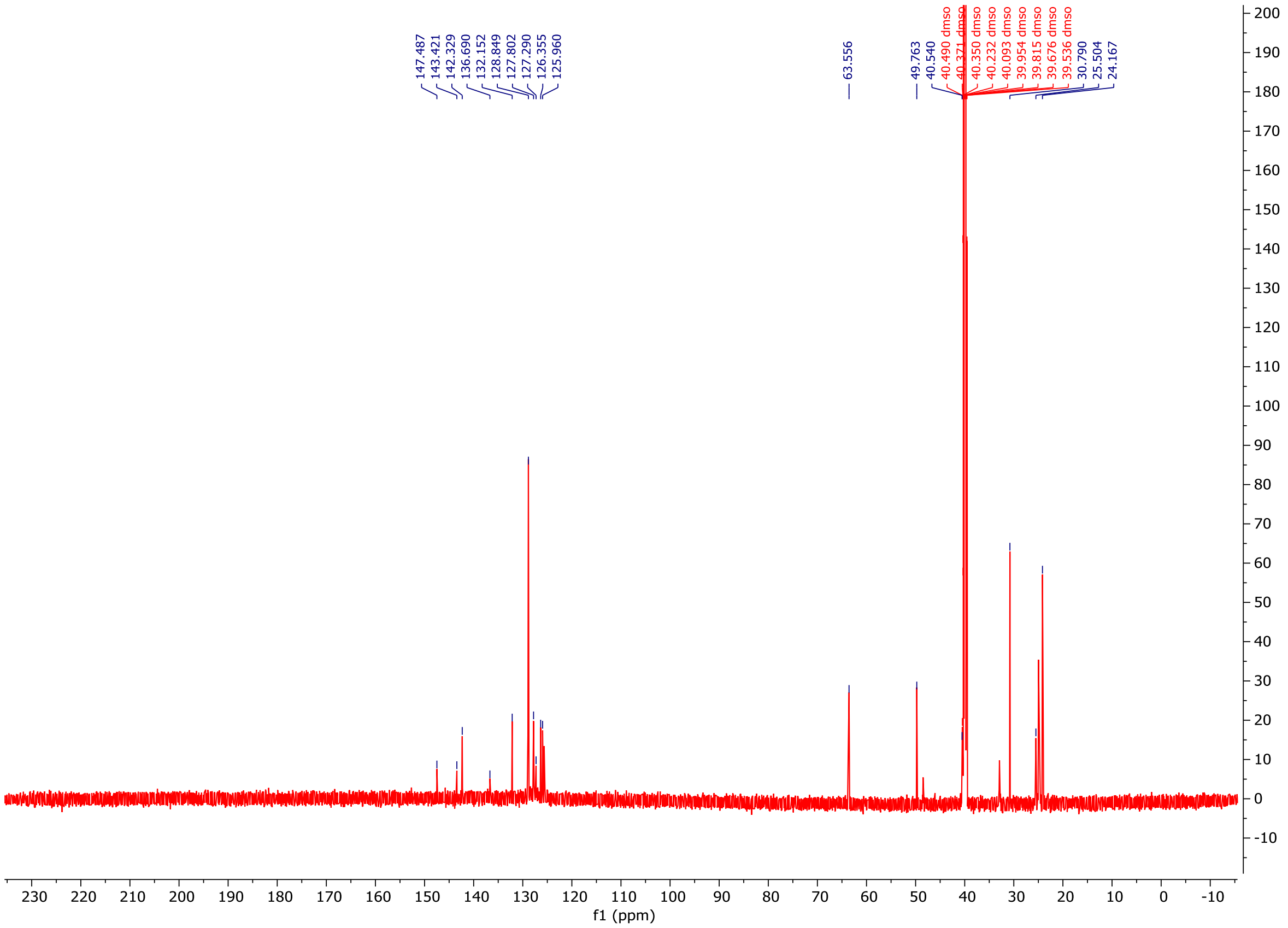

Figure S49. ^{13}C NMR spectrum of compound **46**

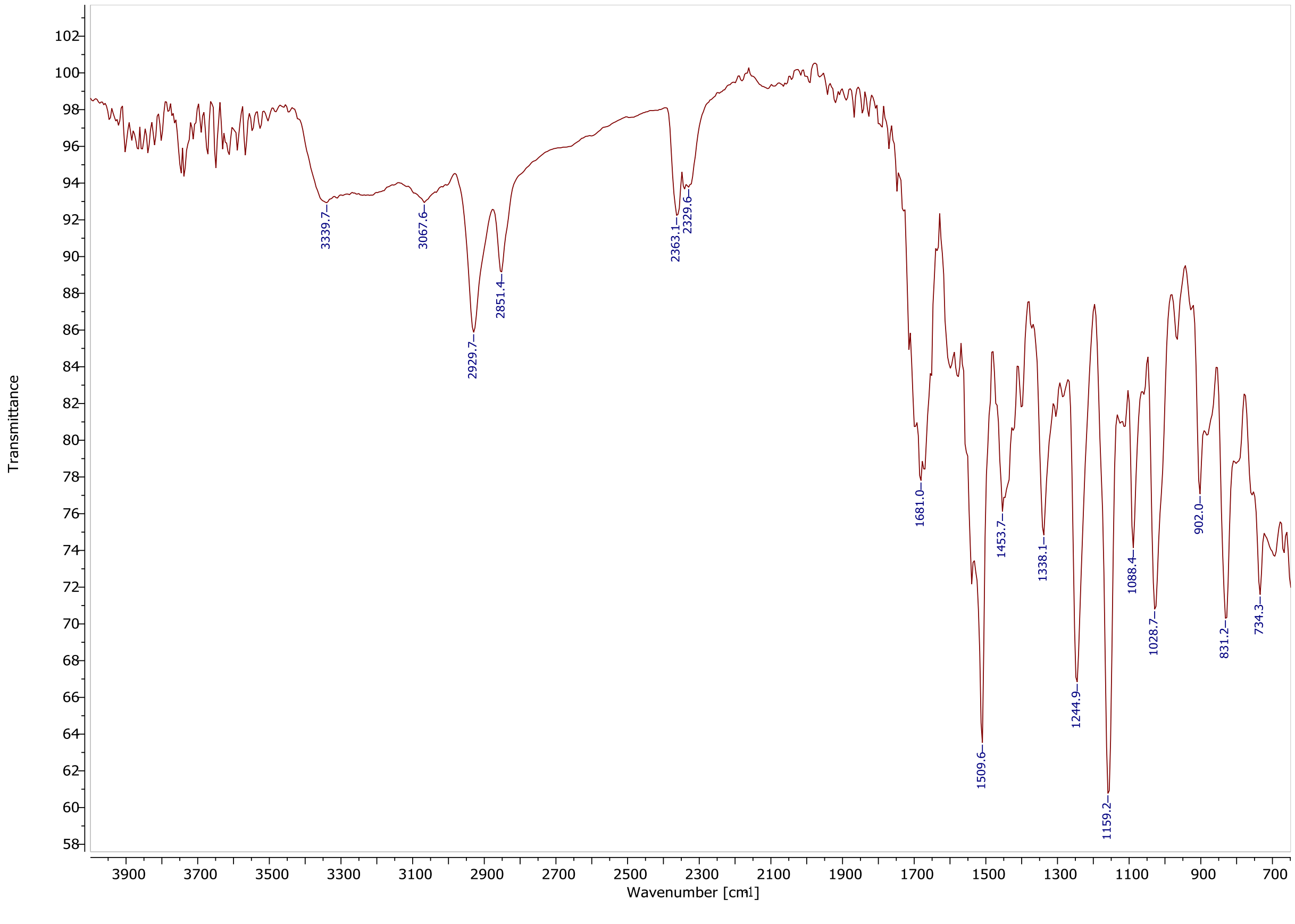

Figure S50. FTIR spectrum of compound **47**

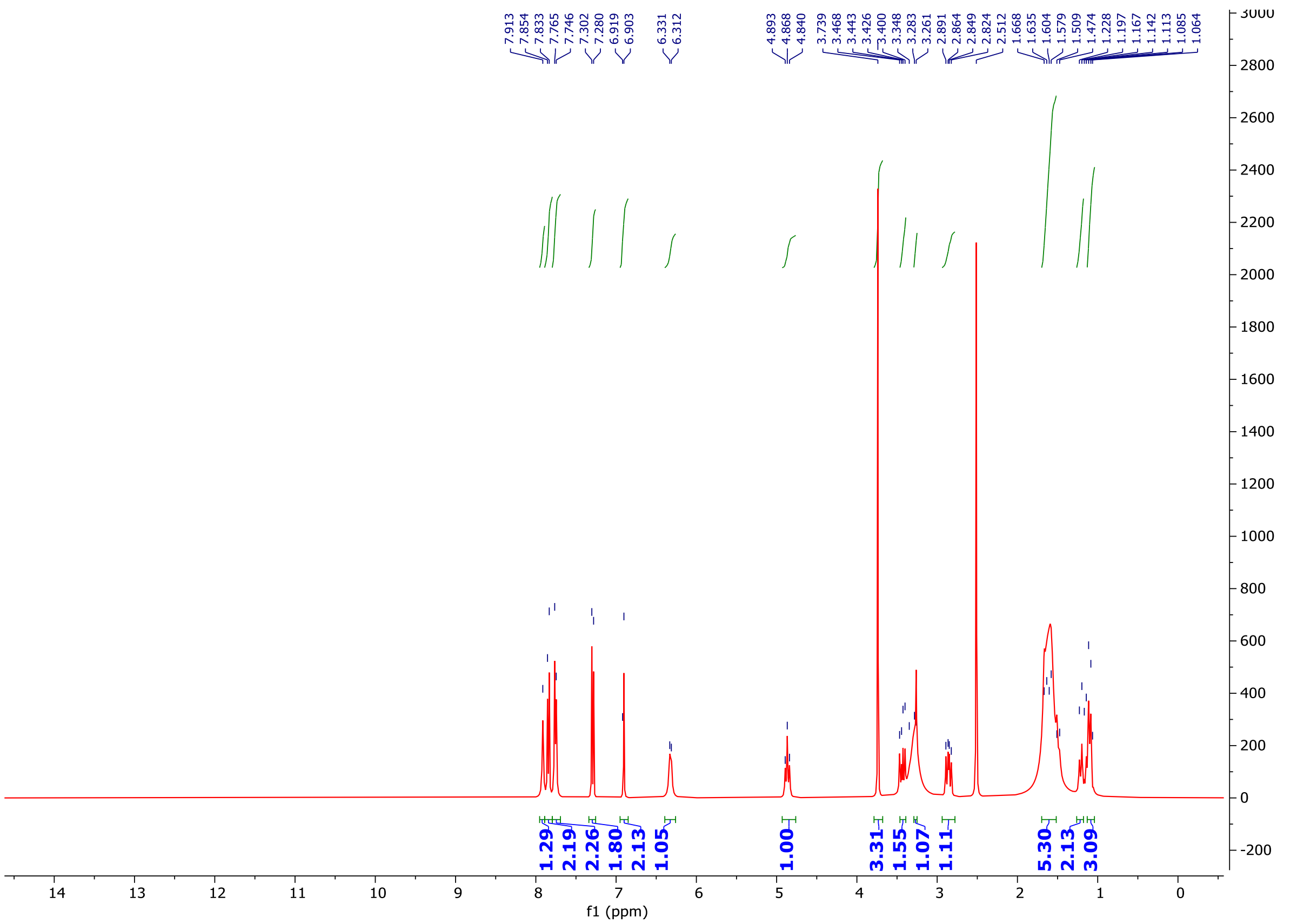

Figure S51. ^{1}H NMR spectrum of compound **47**

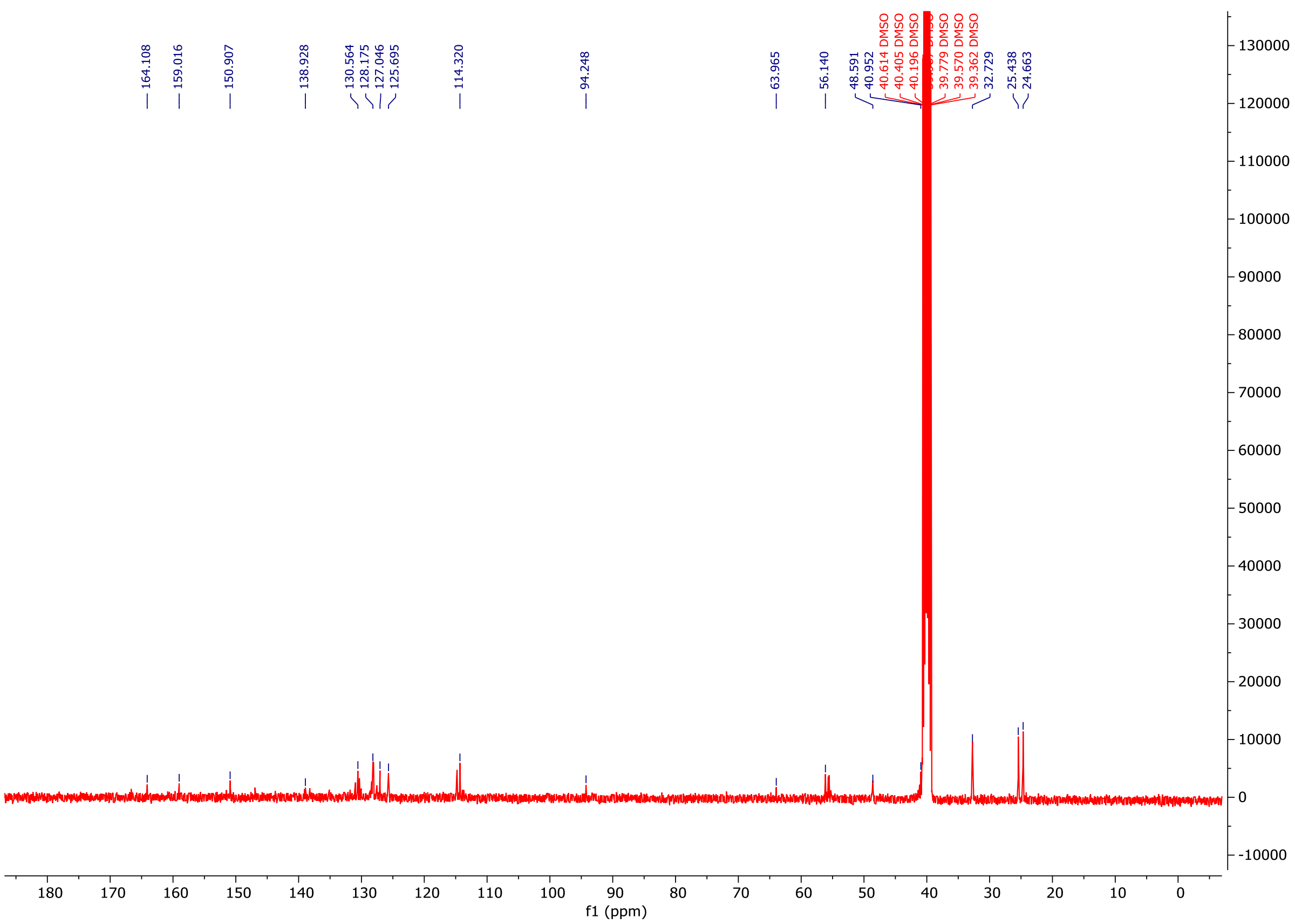

Figure S52. ^{13}C NMR spectrum of compound **47**

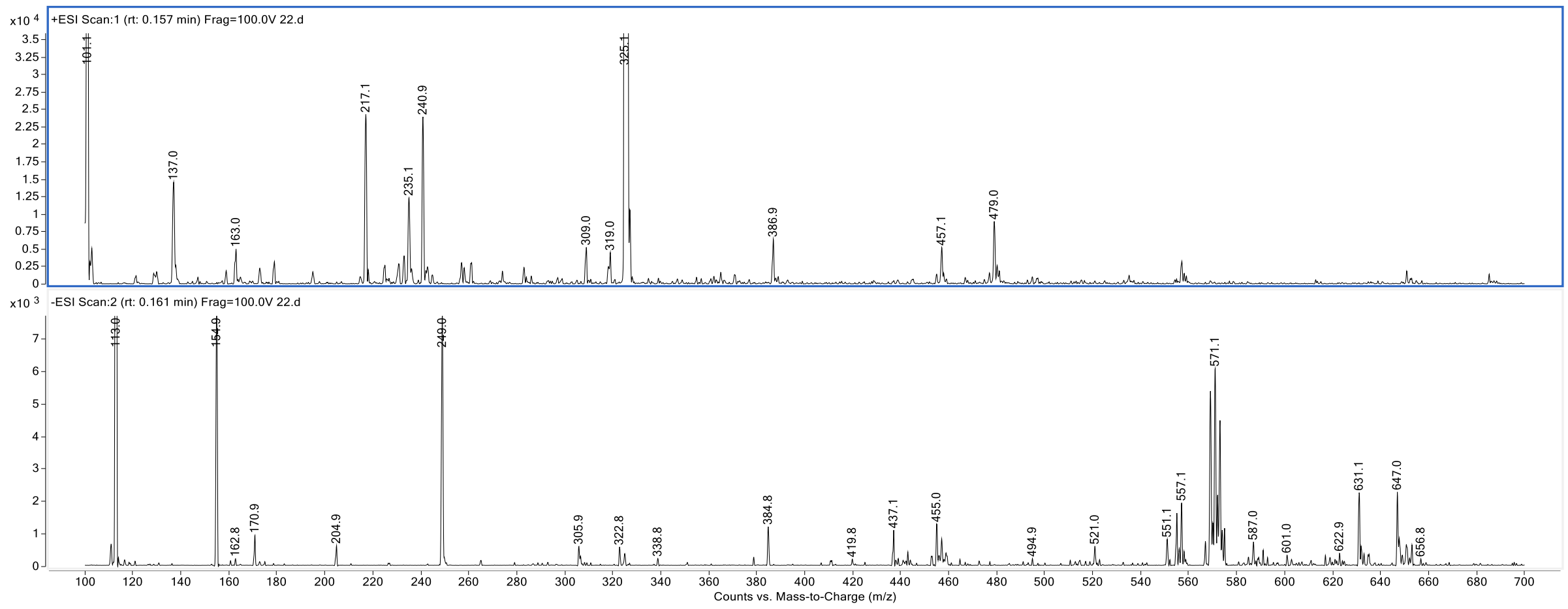

Figure S53. Mass spectrum of compound **47**

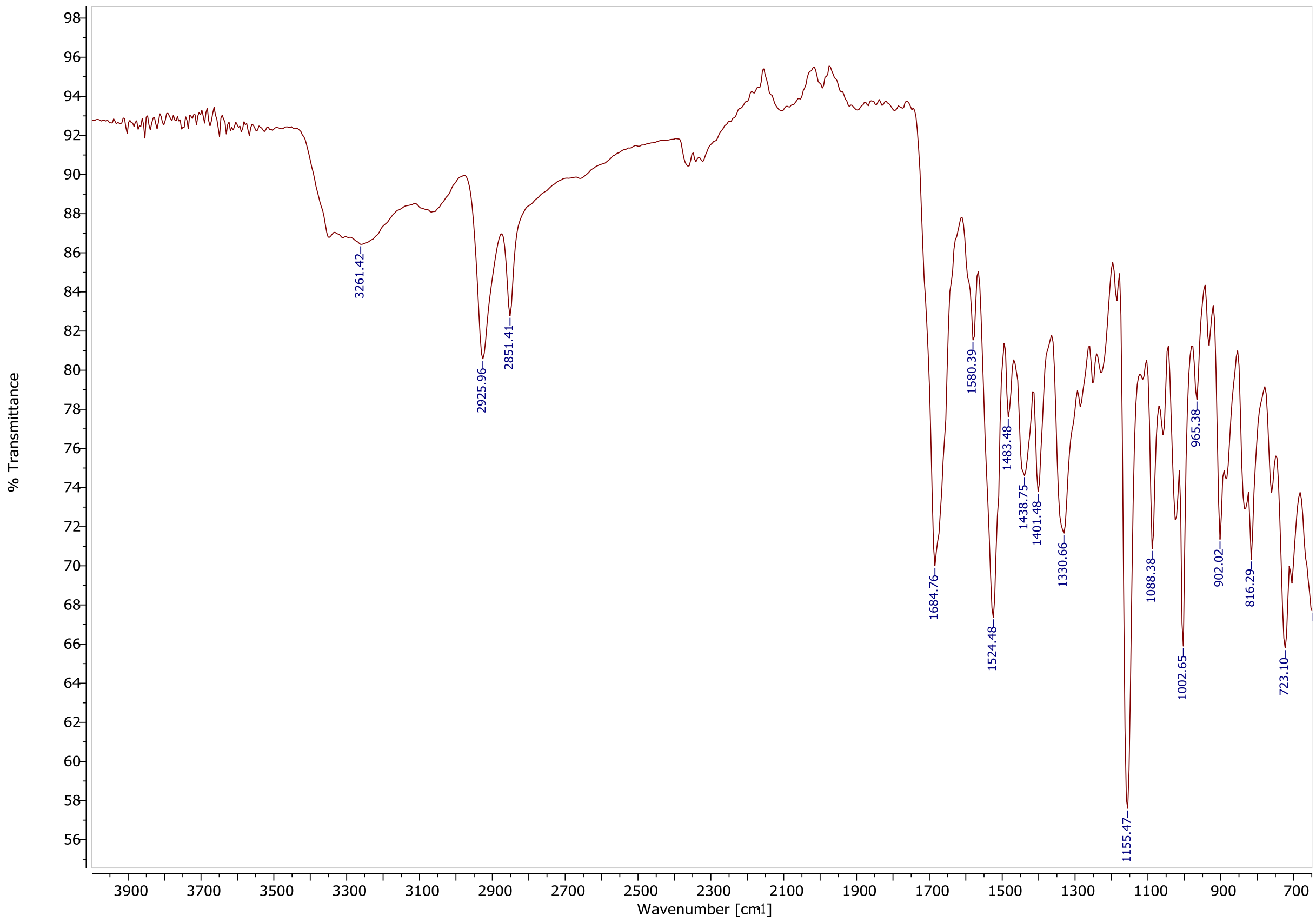

Figure S54. FTIR spectrum of compound **49**

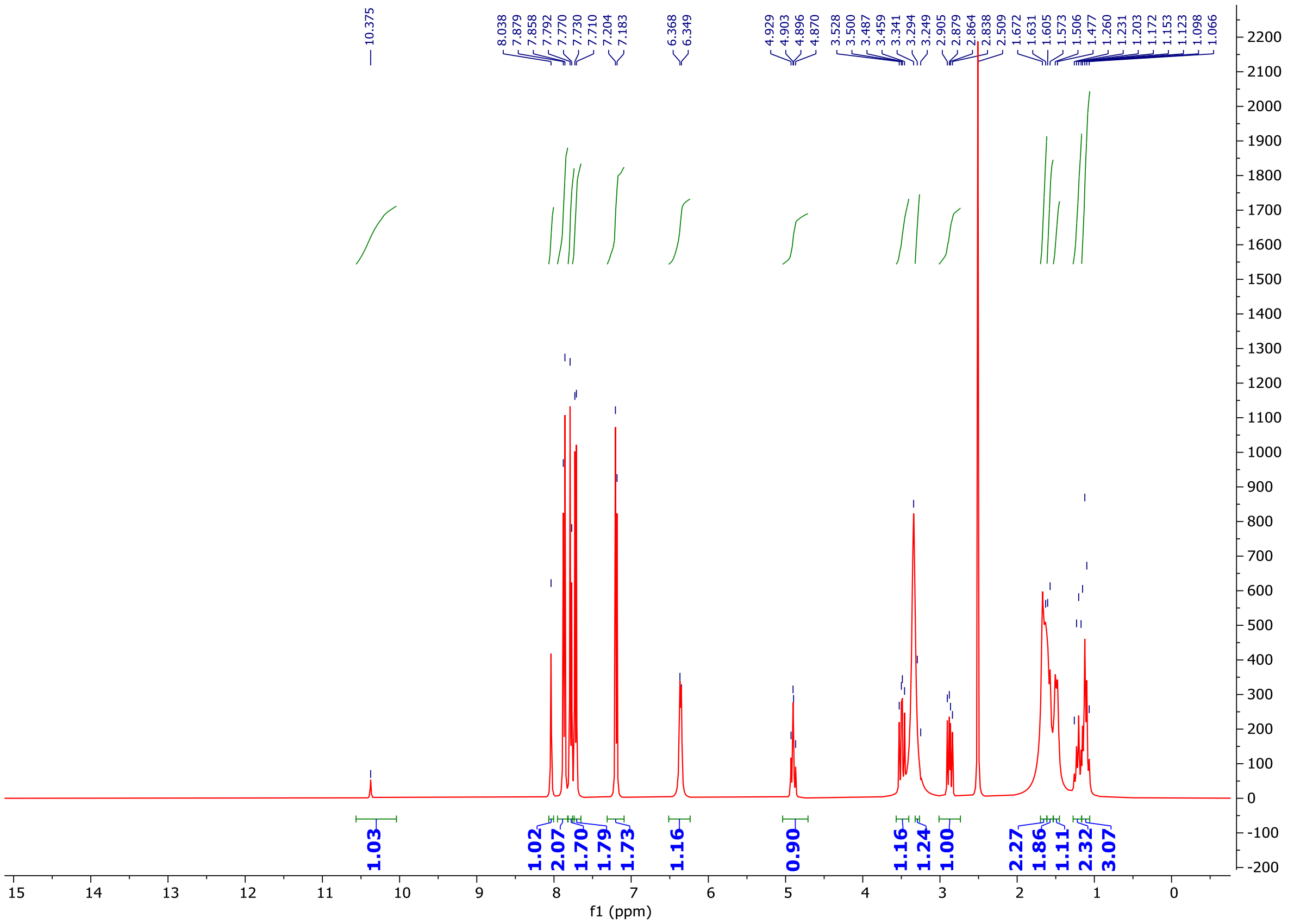

Figure S55. ^{1}H NMR spectrum of compound **49**

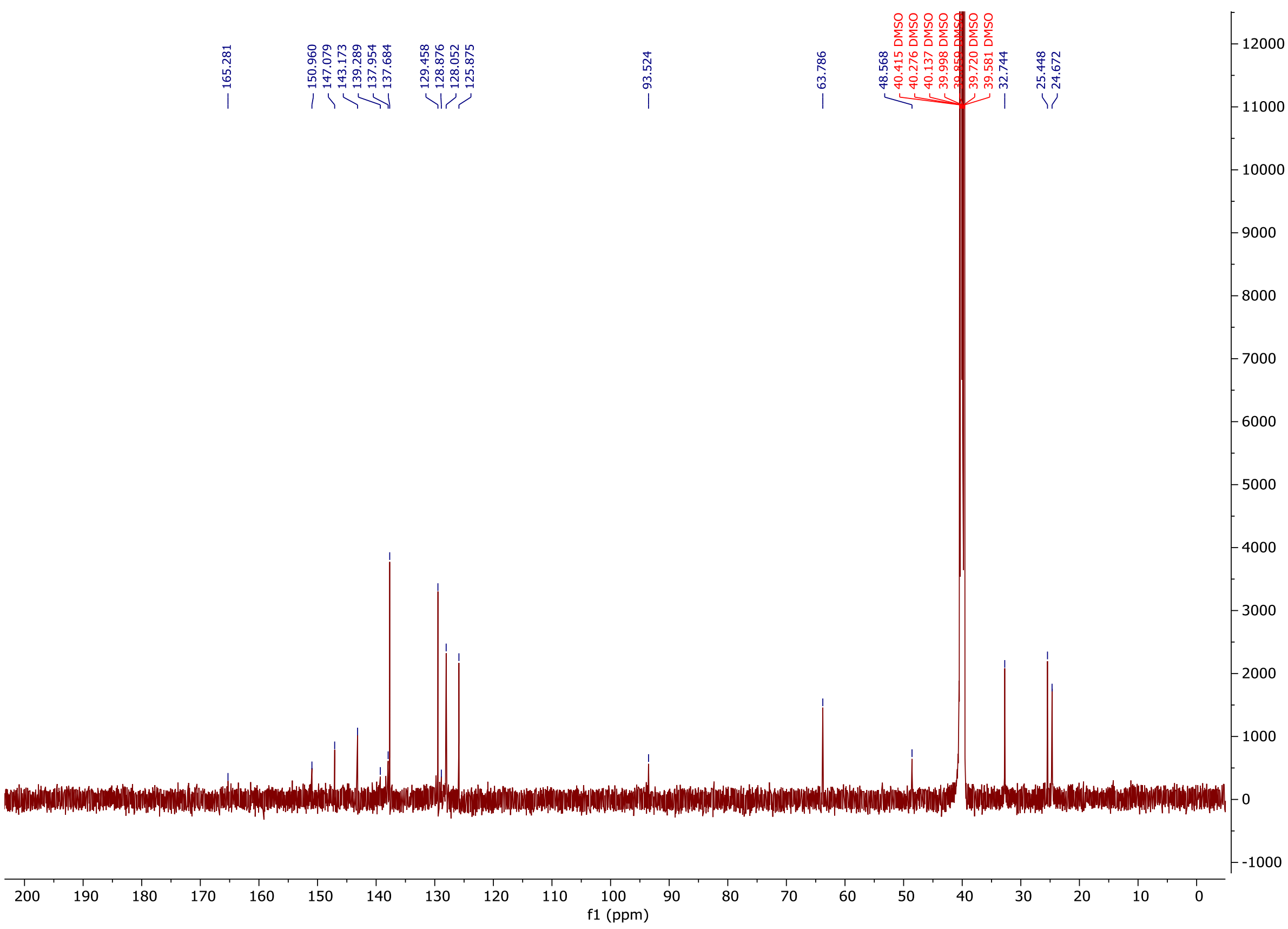

Figure S56. ^{13}C NMR spectrum of compound **49**

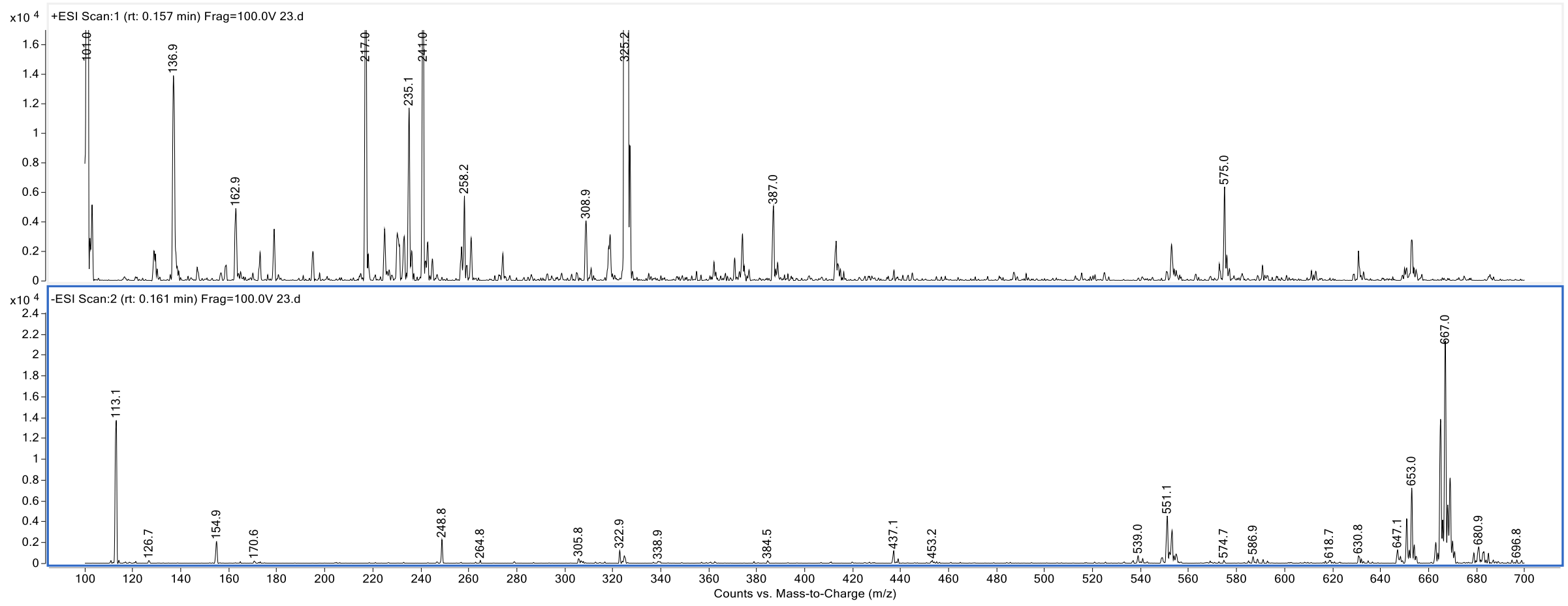

Figure S57. Mass spectrum of compound **49**

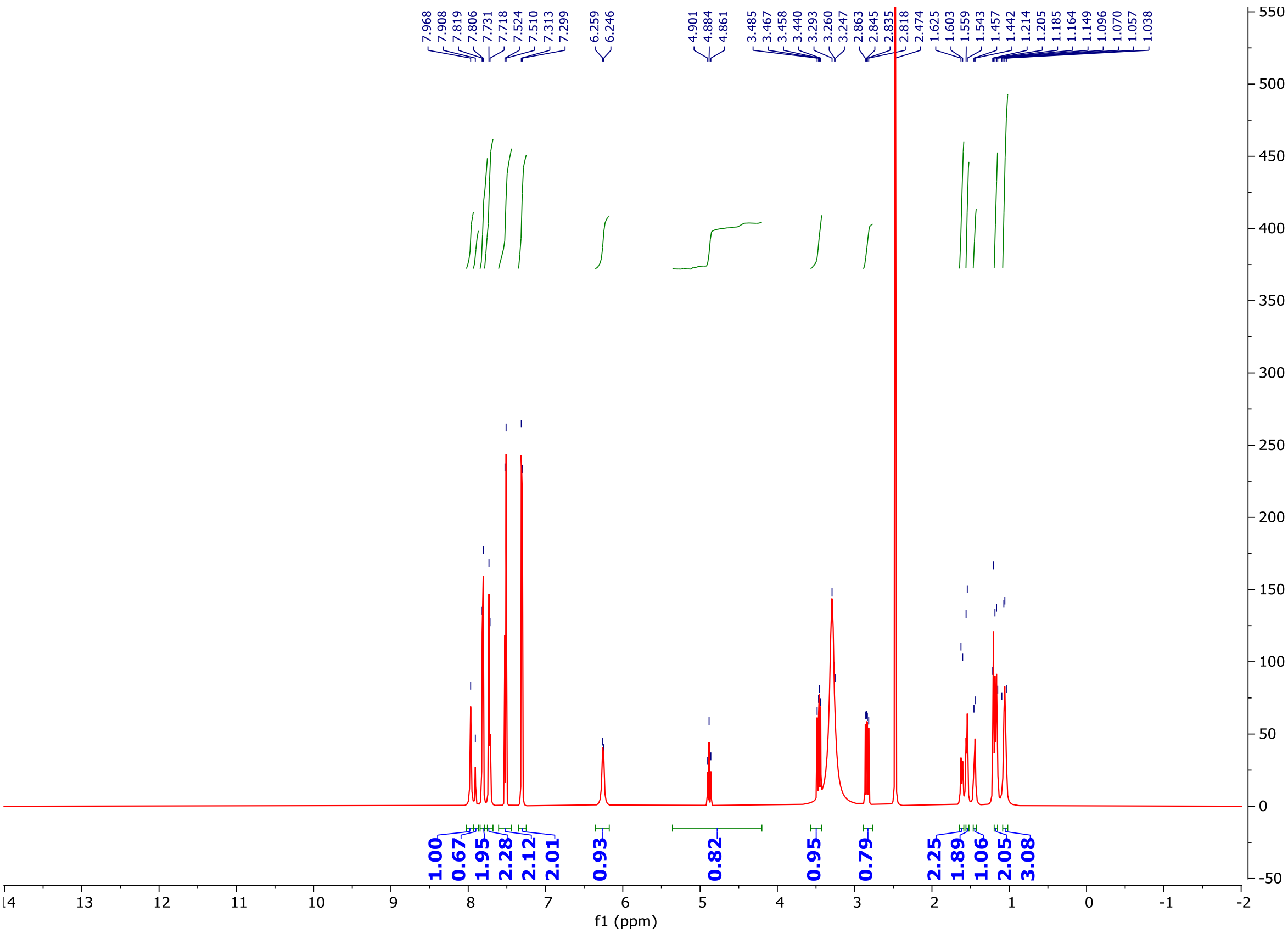

Figure S58. ^{1}H NMR spectrum of compound **54**

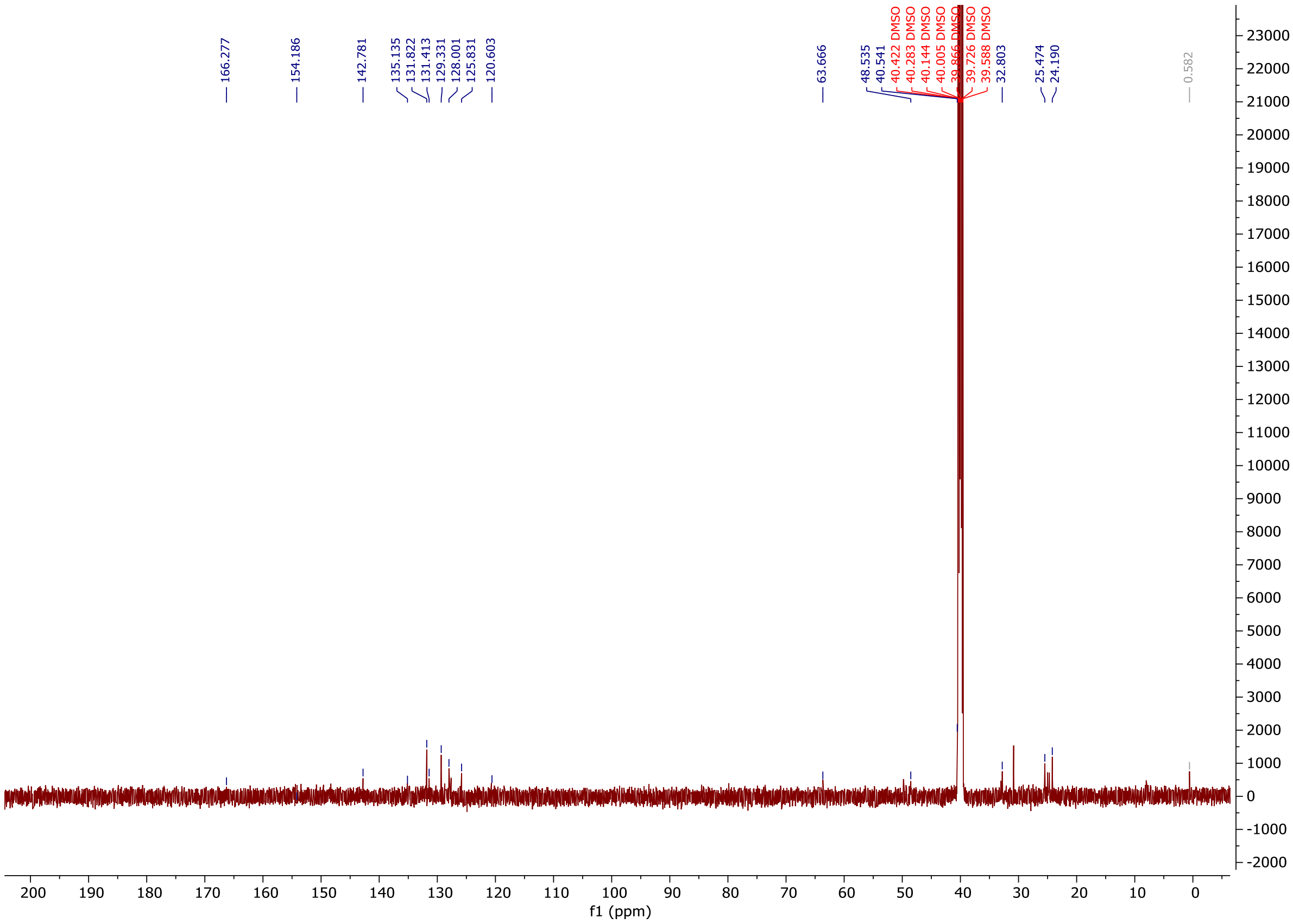

Figure S59. ^{13}C NMR spectrum of compound **54**

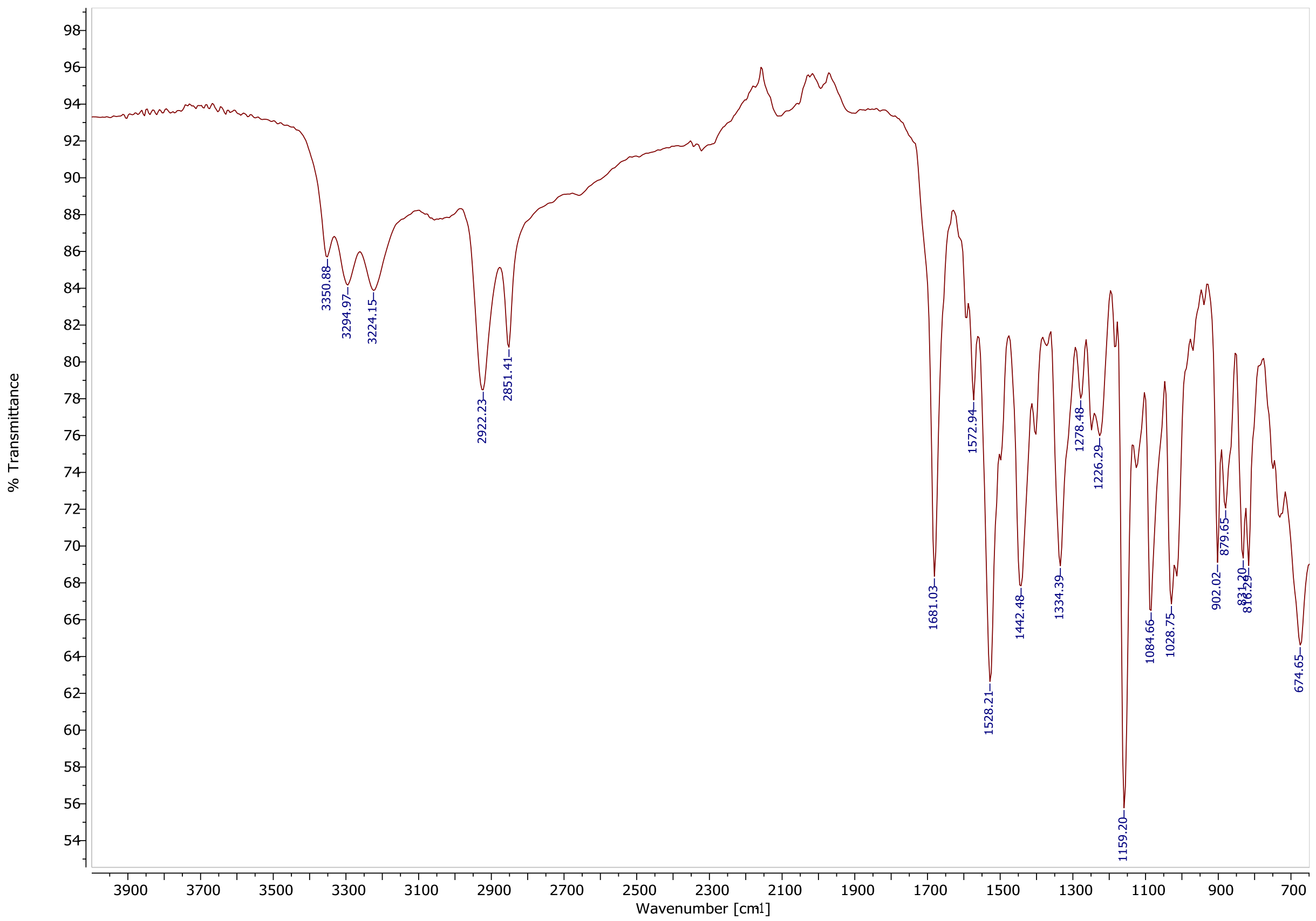

Figure S60.. FTIR spectrum of compound **64**

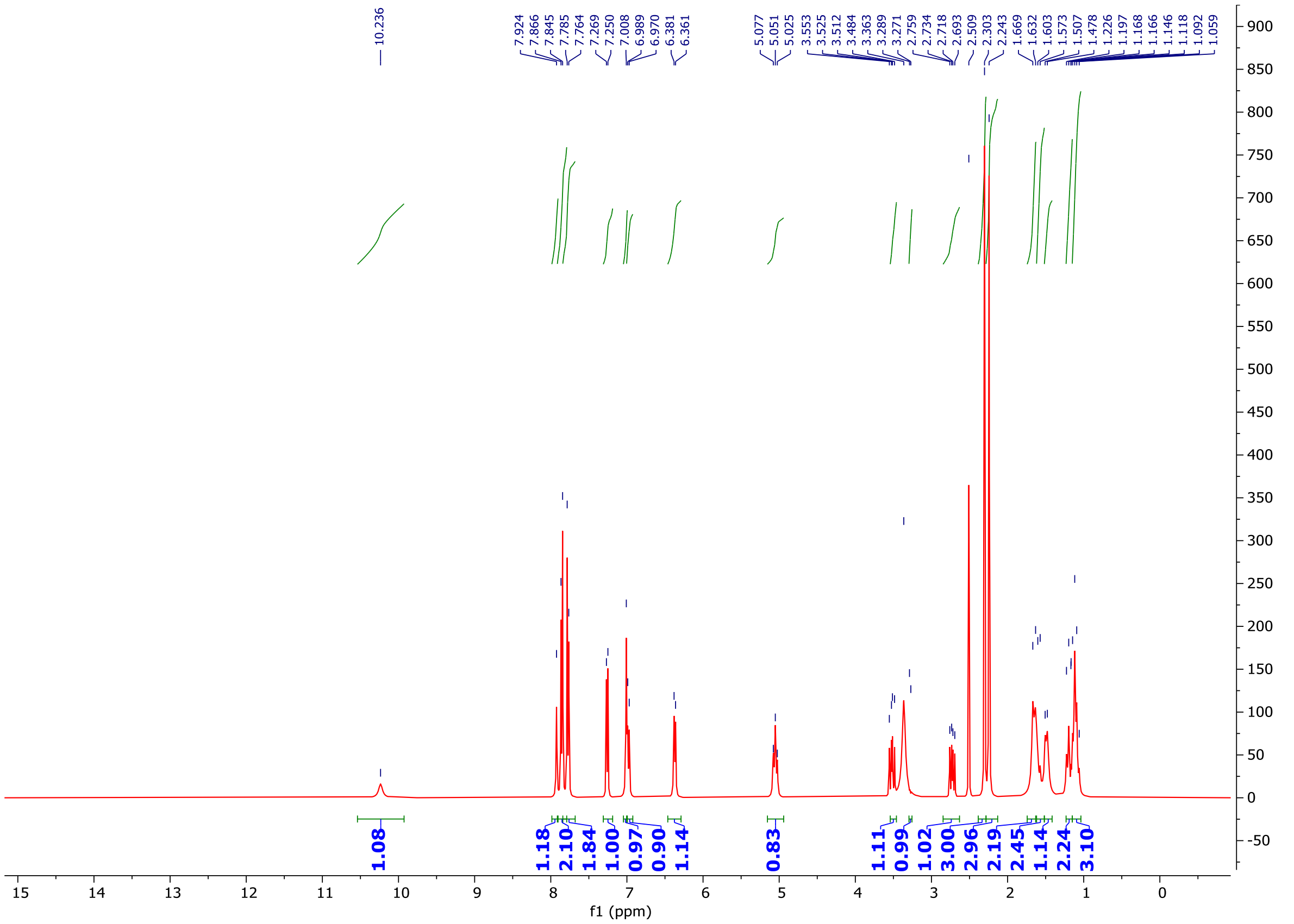

Figure S61. ^{1}H NMR spectrum of compound **64**

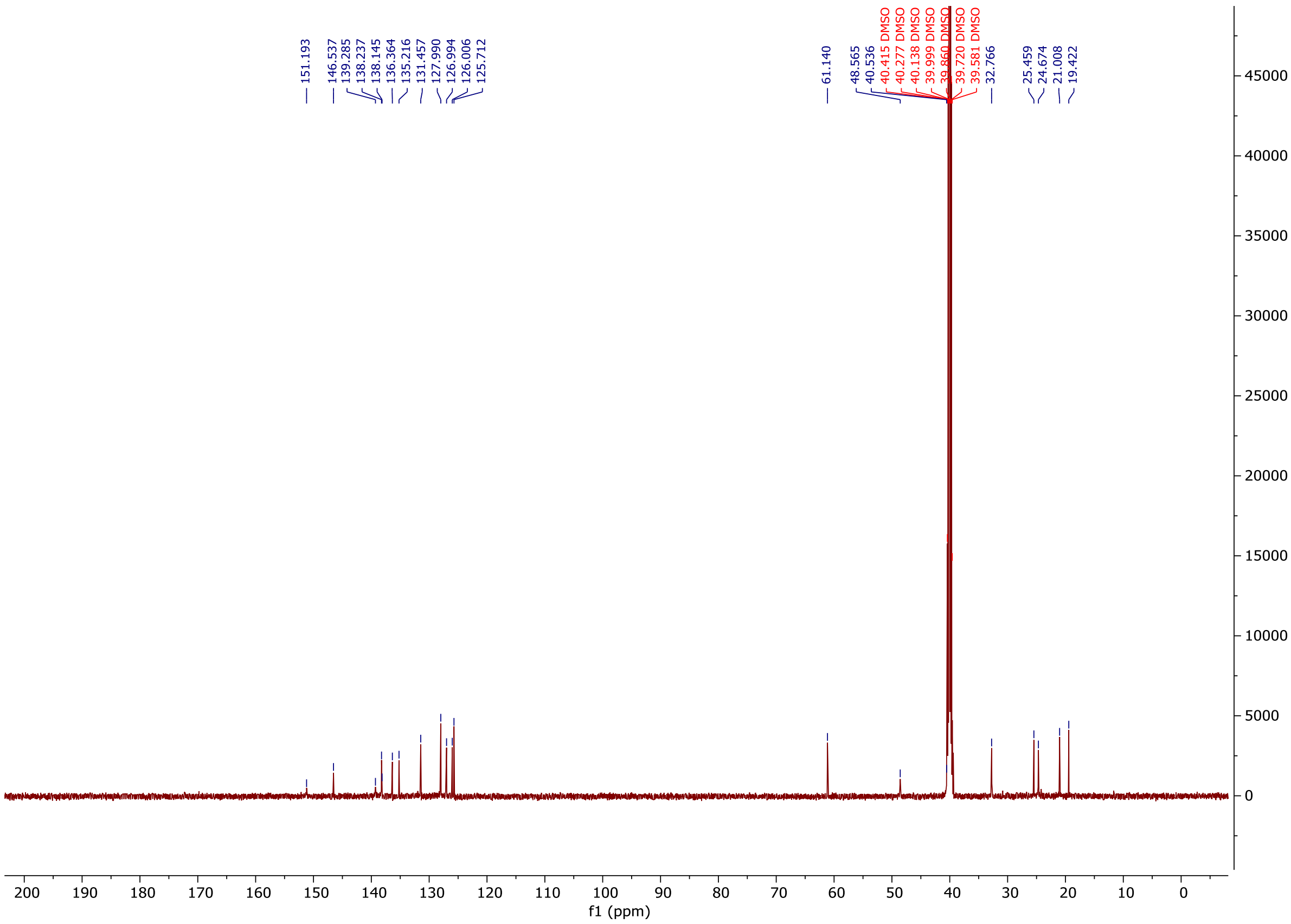

Figure S62. ^{13}C NMR spectrum of compound **64**

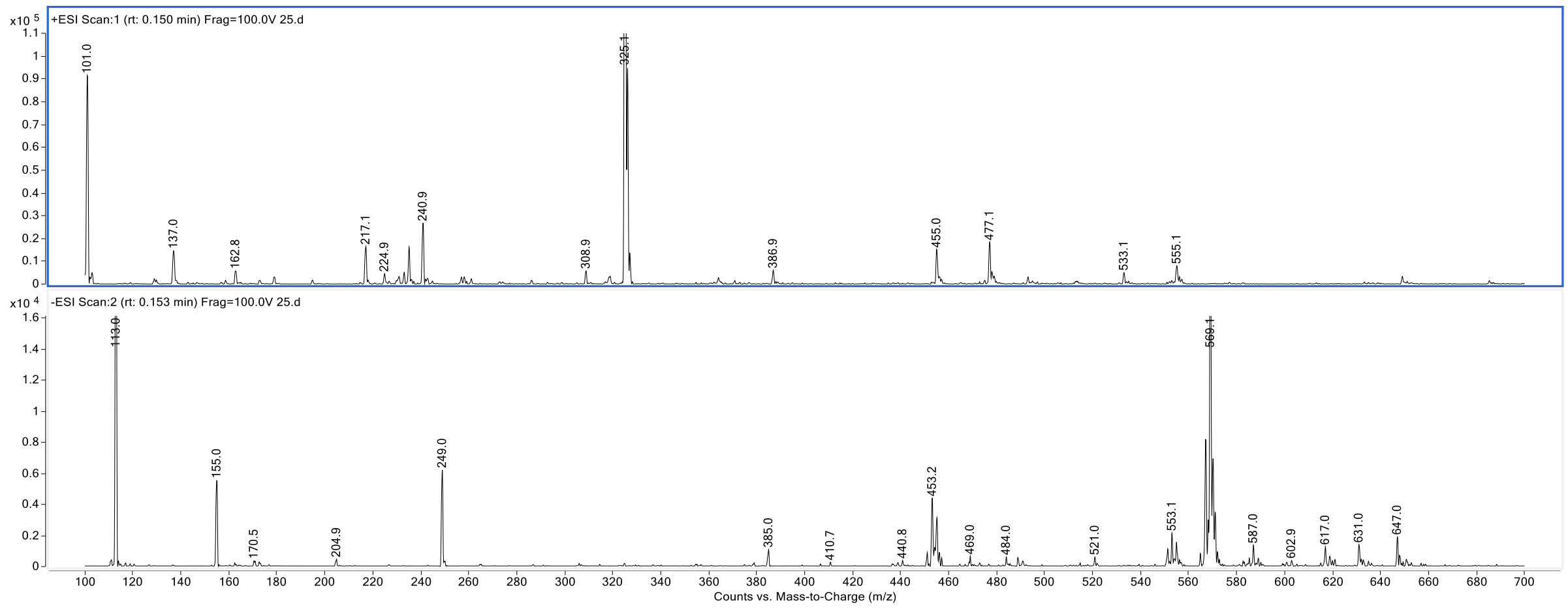

Figure S63. Mass spectrum of compound **64**

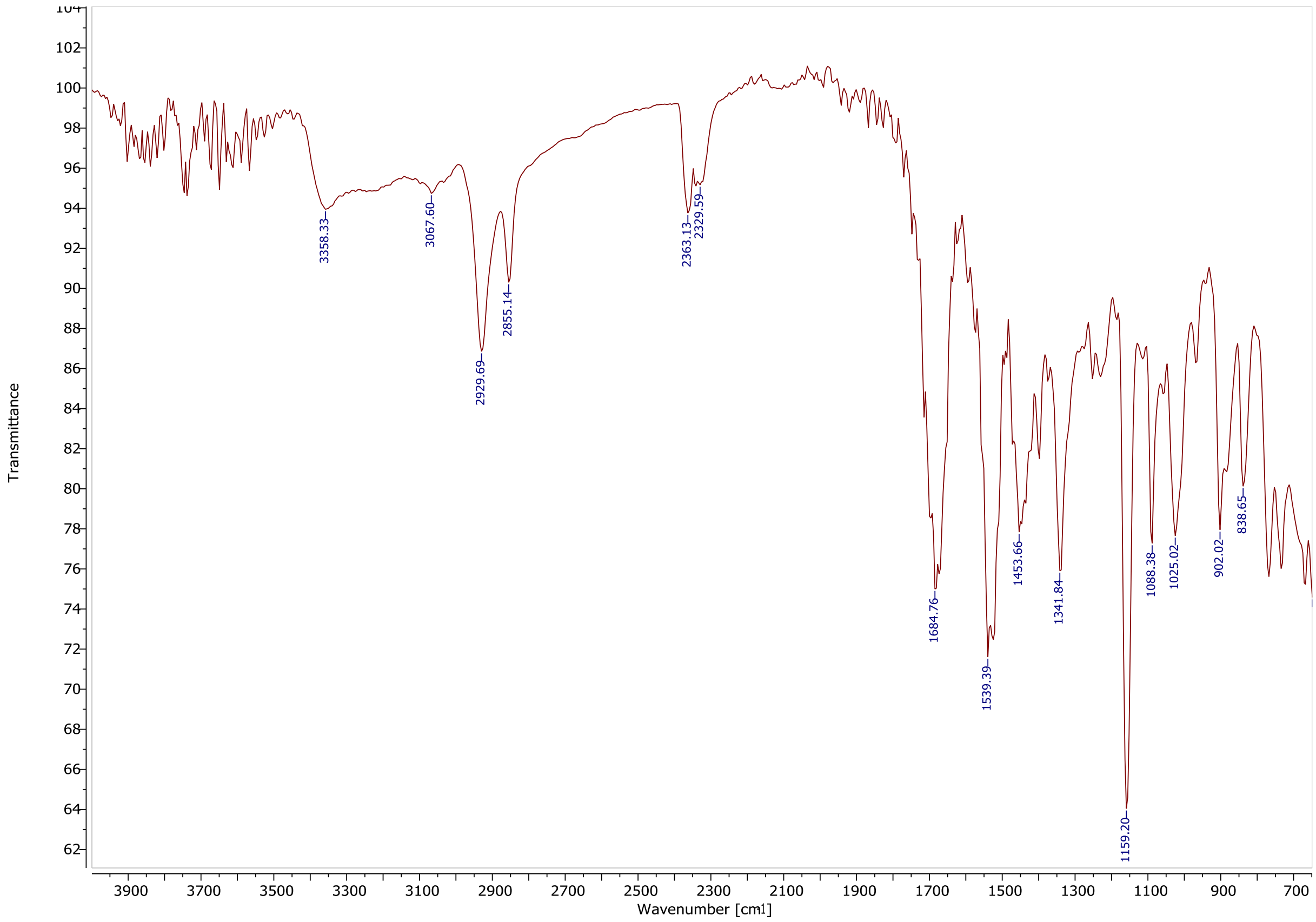

Figure S64. FTIR spectrum of compound **65**

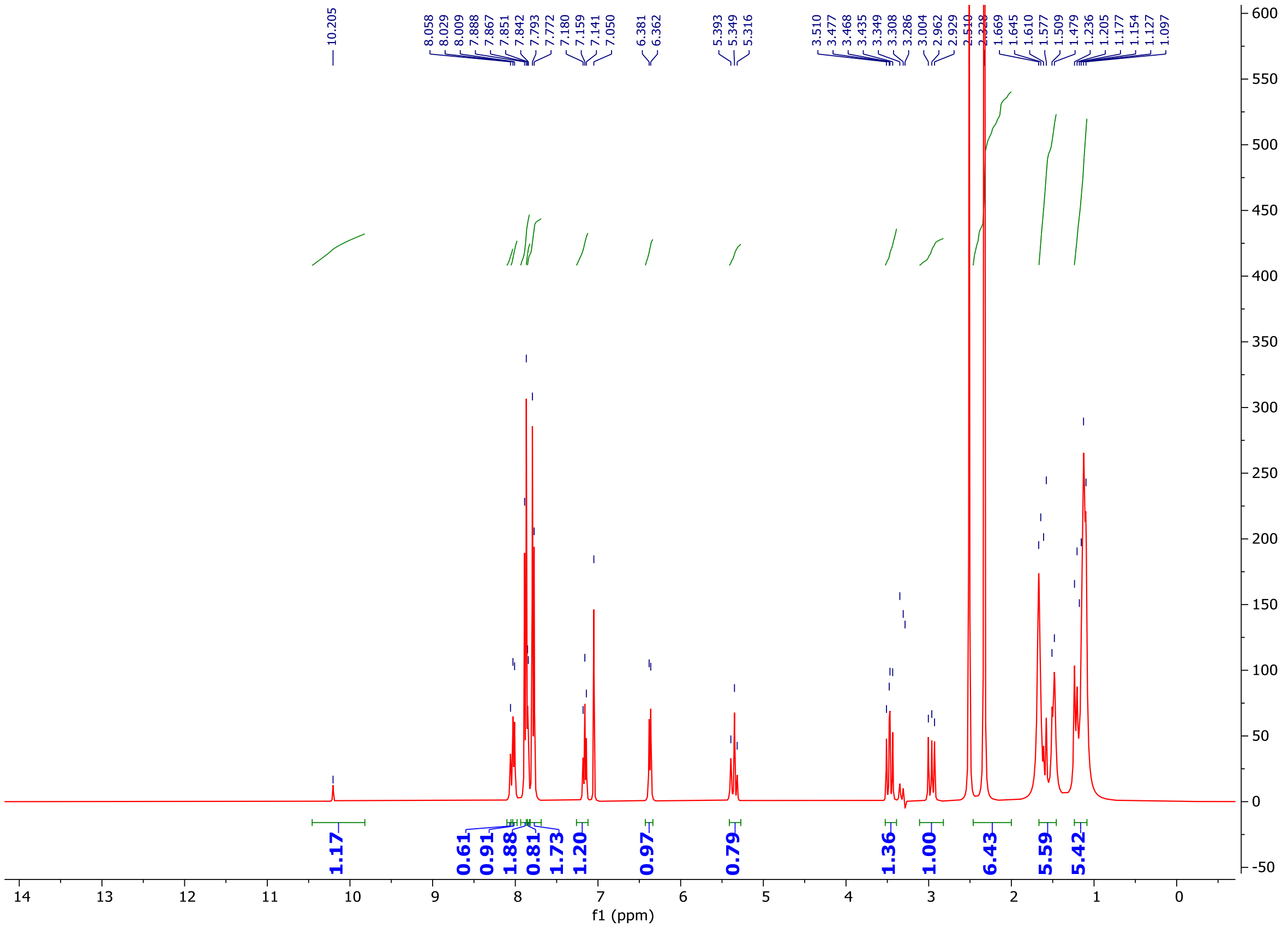

Figure S65. ^{1}H NMR spectrum of compound **65**

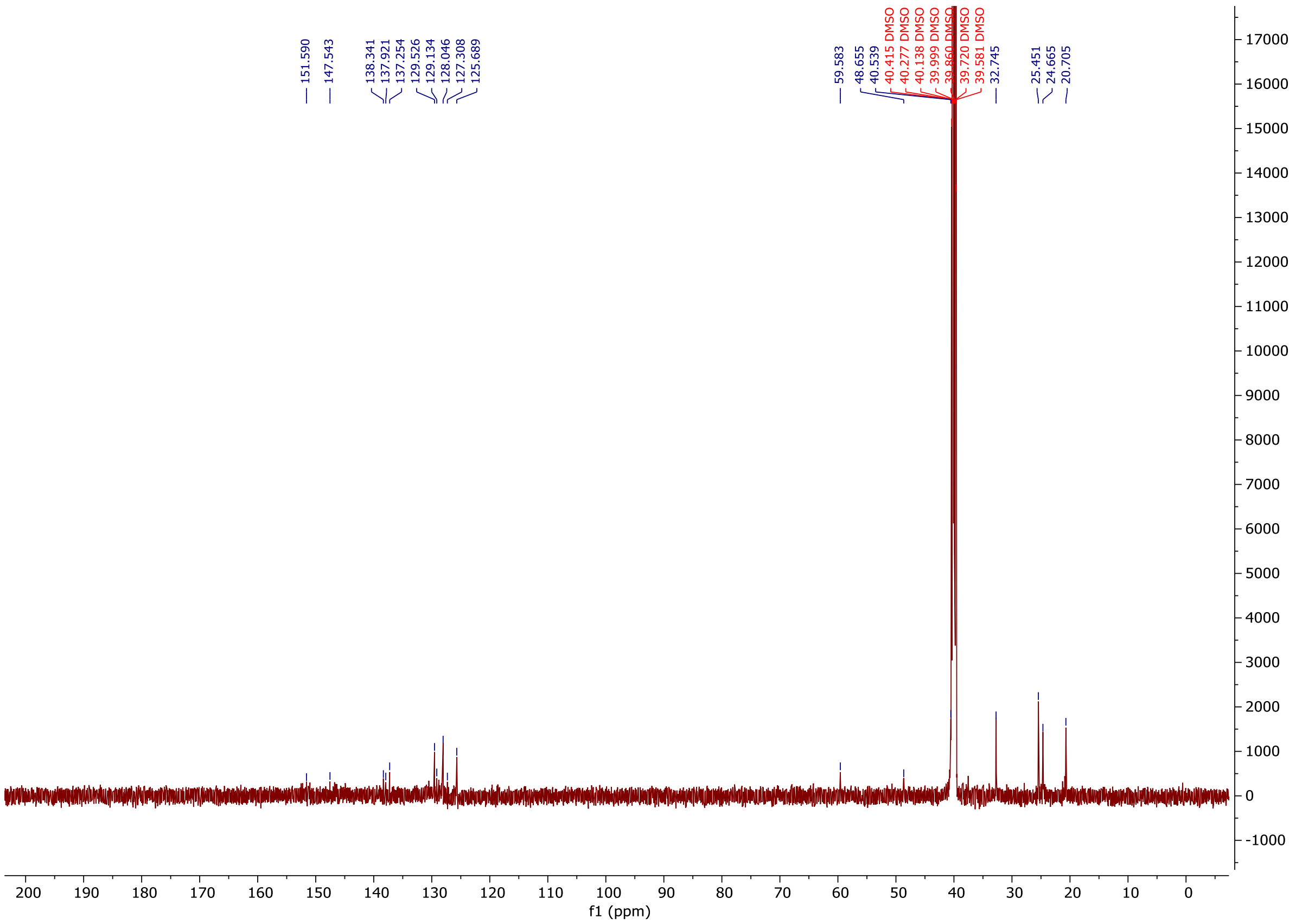

Figure S66 ^{13}C NMR spectrum of compound **65**

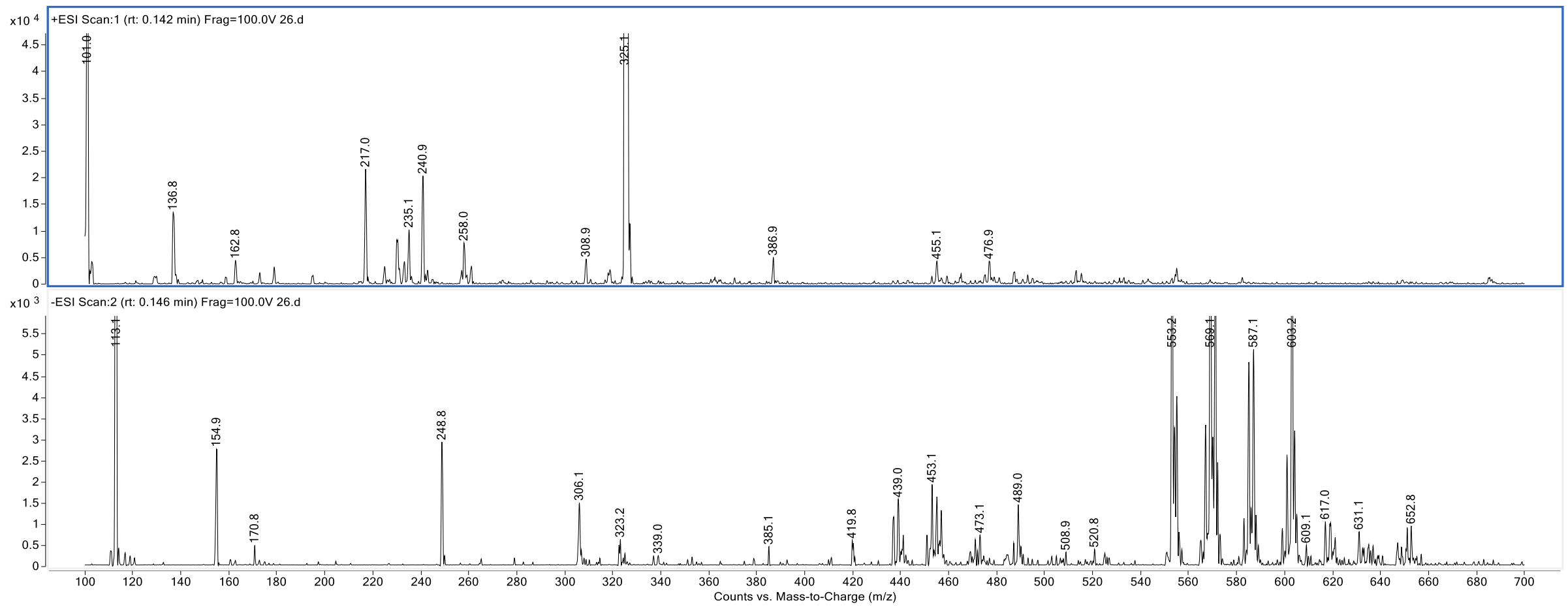

Figure S67. Mass spectrum of compound **65**

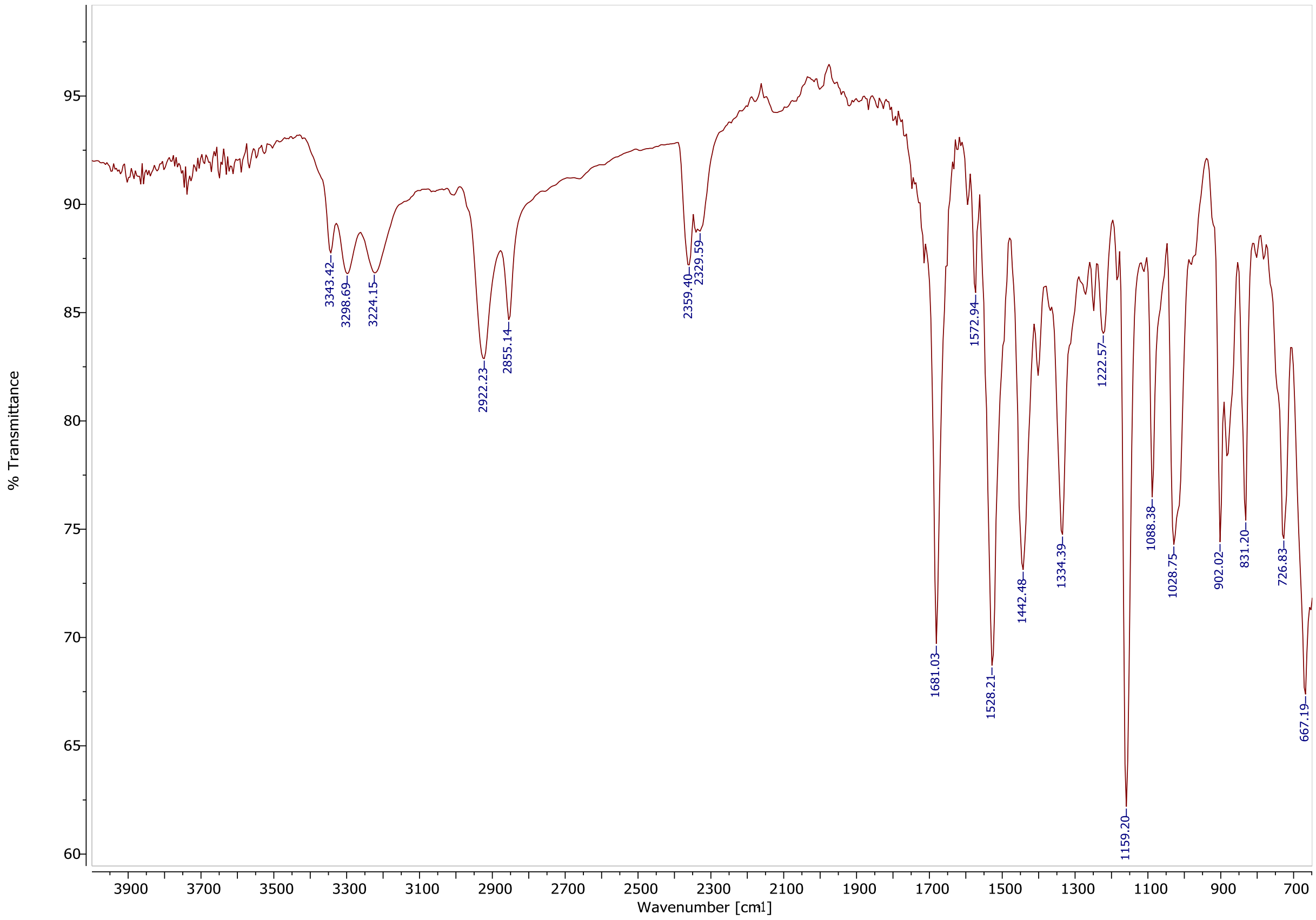

Figure S68. FTIR spectrum of compound **66**

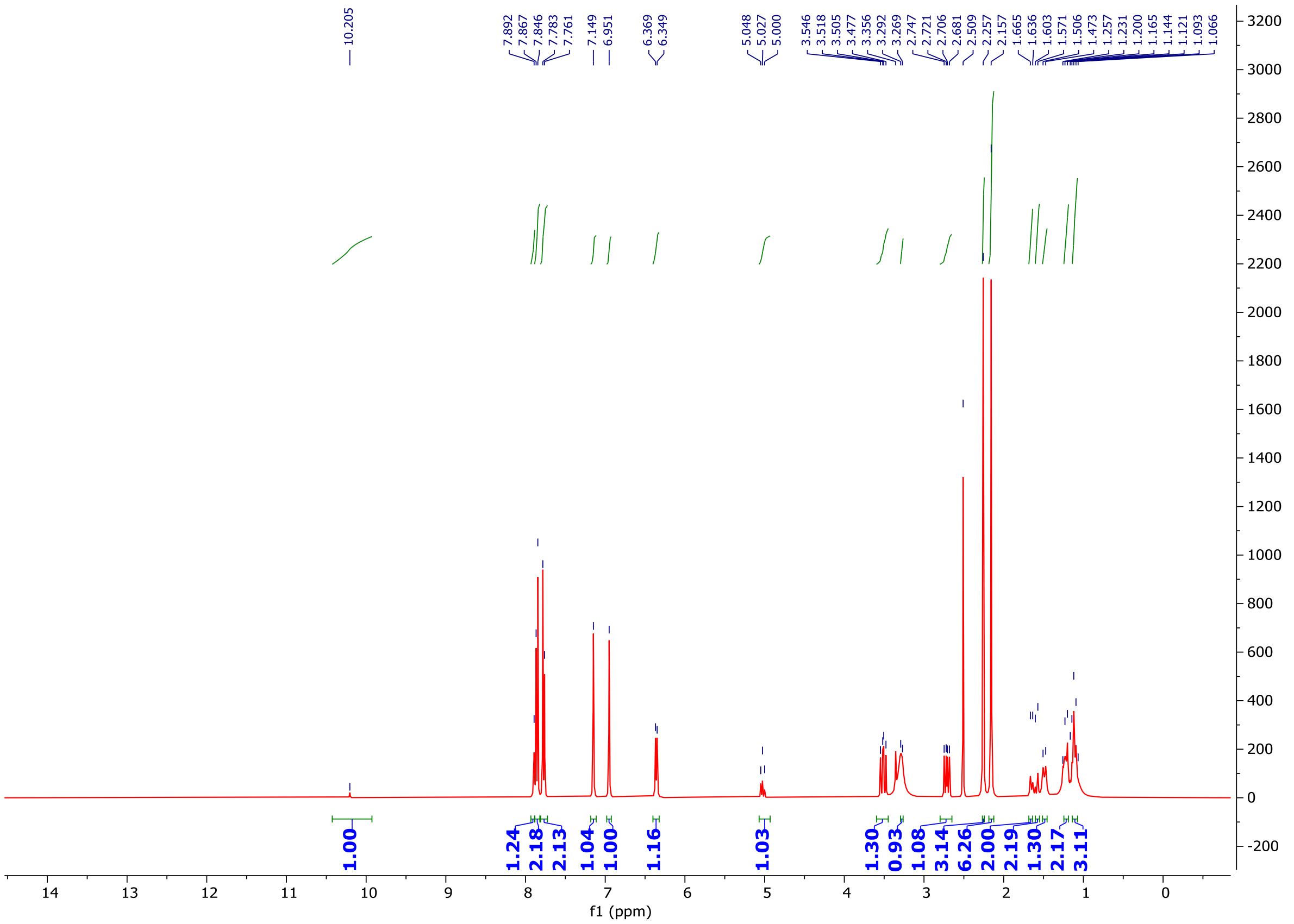

Figure S69. ^{1}H NMR spectrum of compound **66**

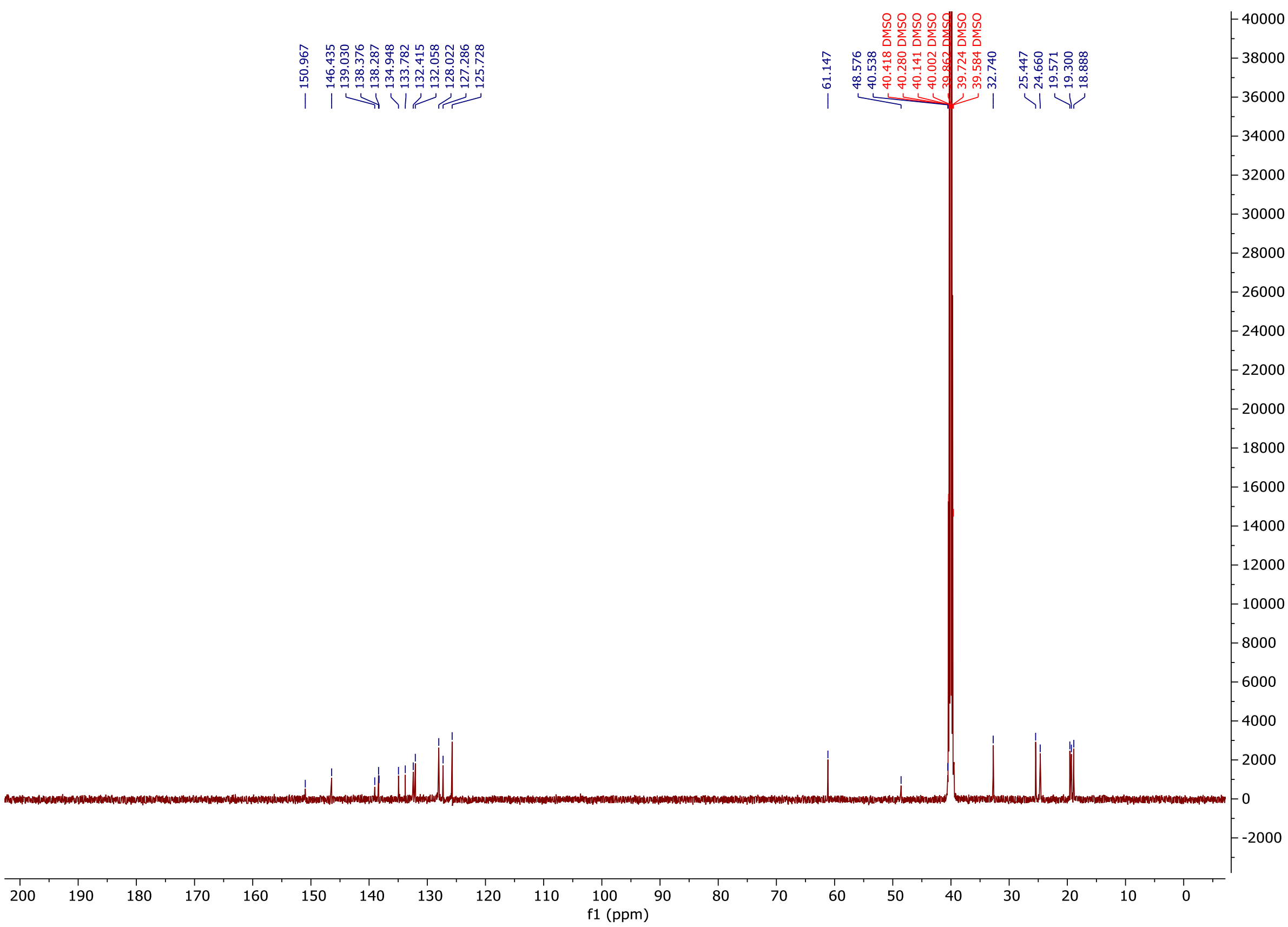

Figure S70. ^{13}C NMR spectrum of compound **66**

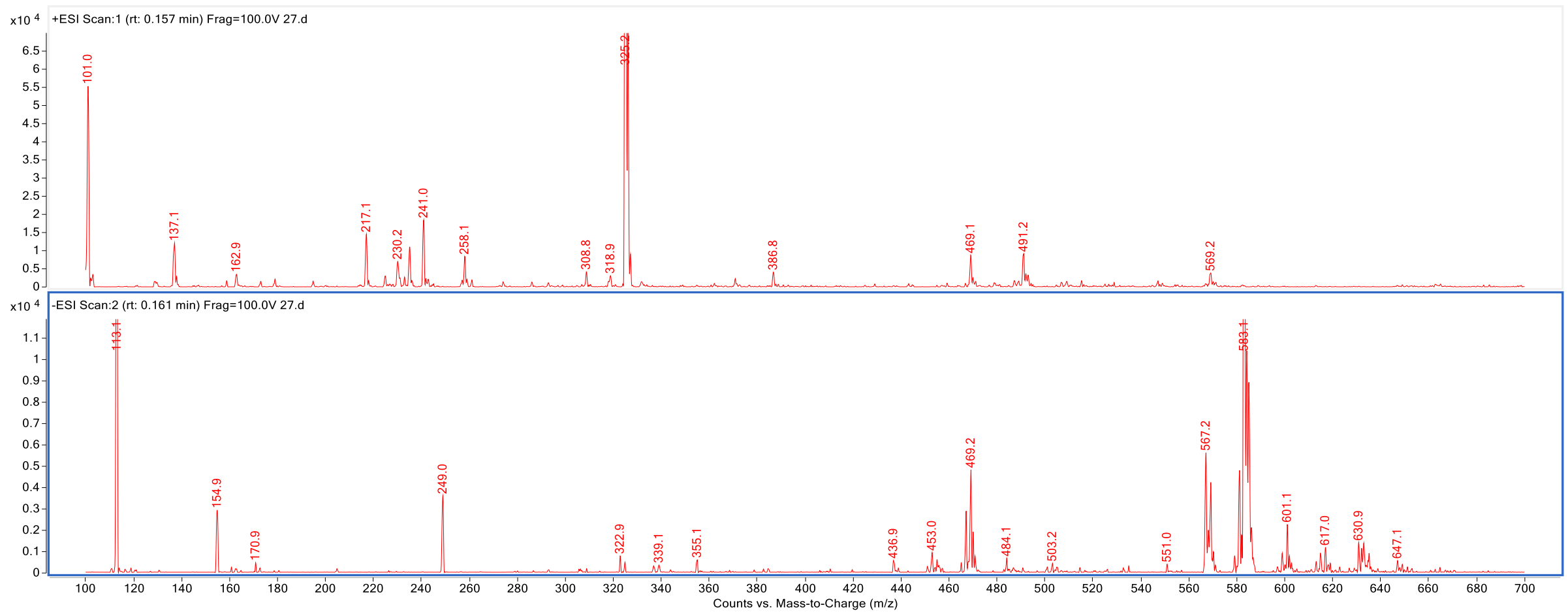

Figure S71. Mass spectrum of compound **66**

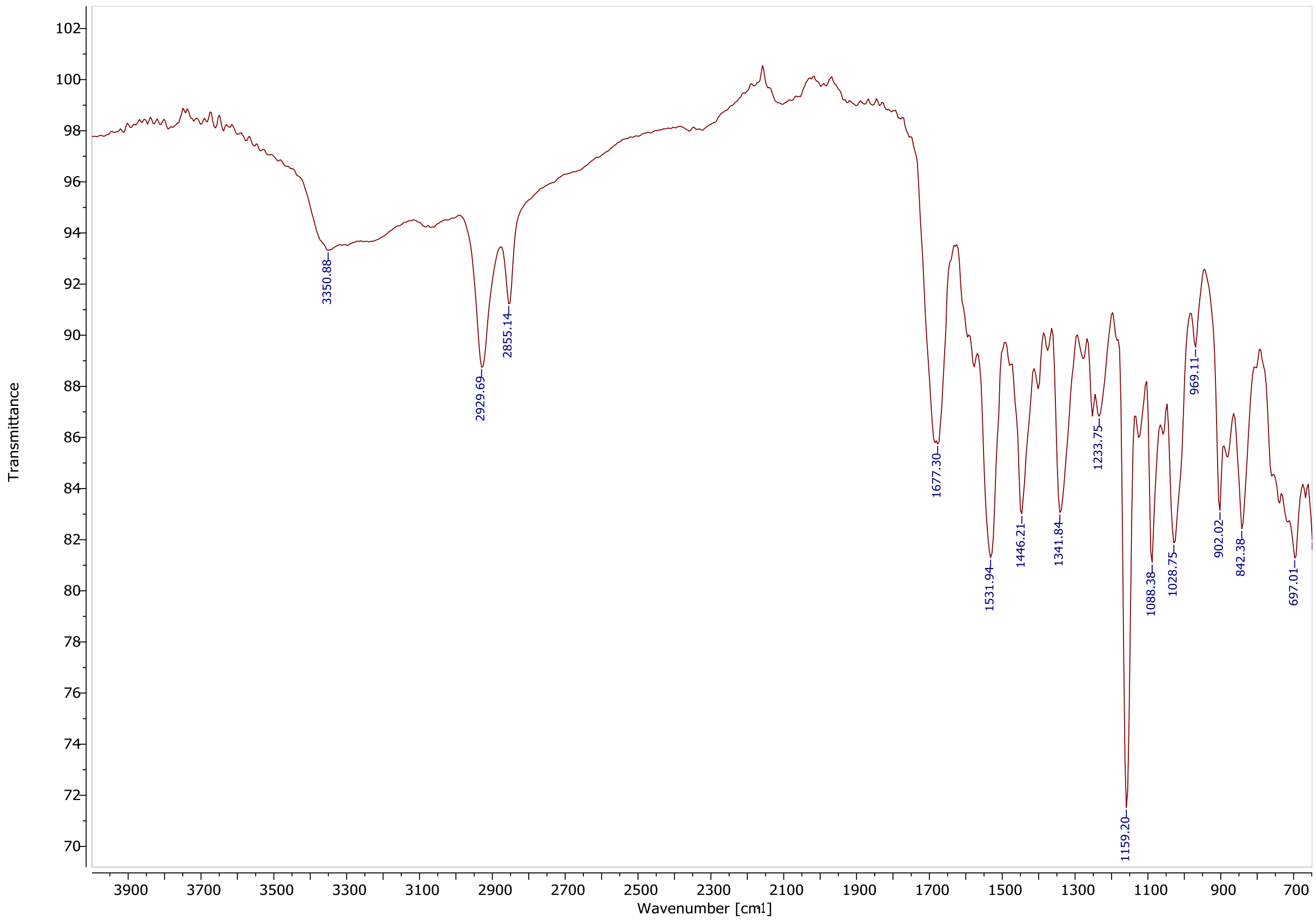

Figure S72. FTIR spectrum of compound **67**

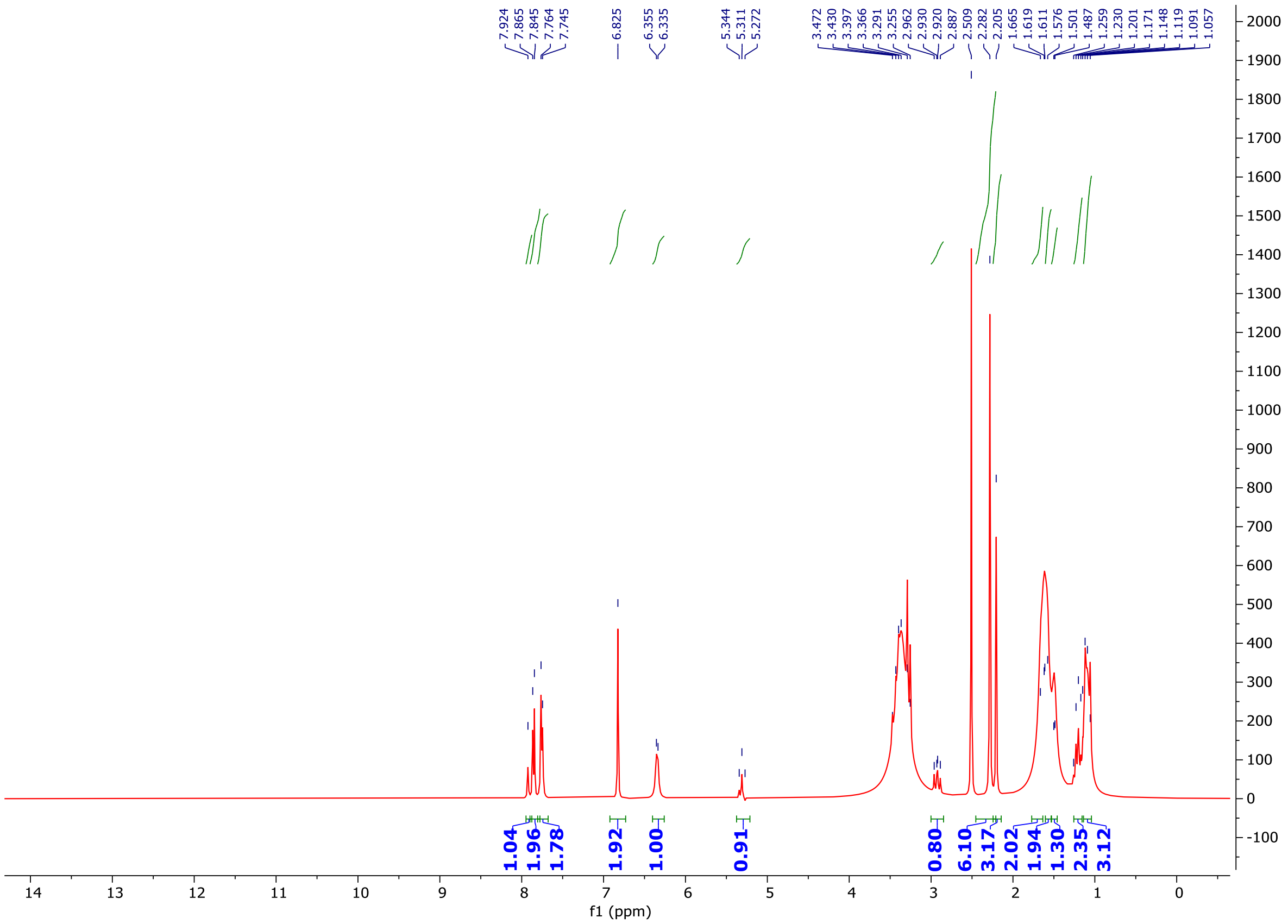

Figure S73. ^{1}H NMR spectrum of compound **67**

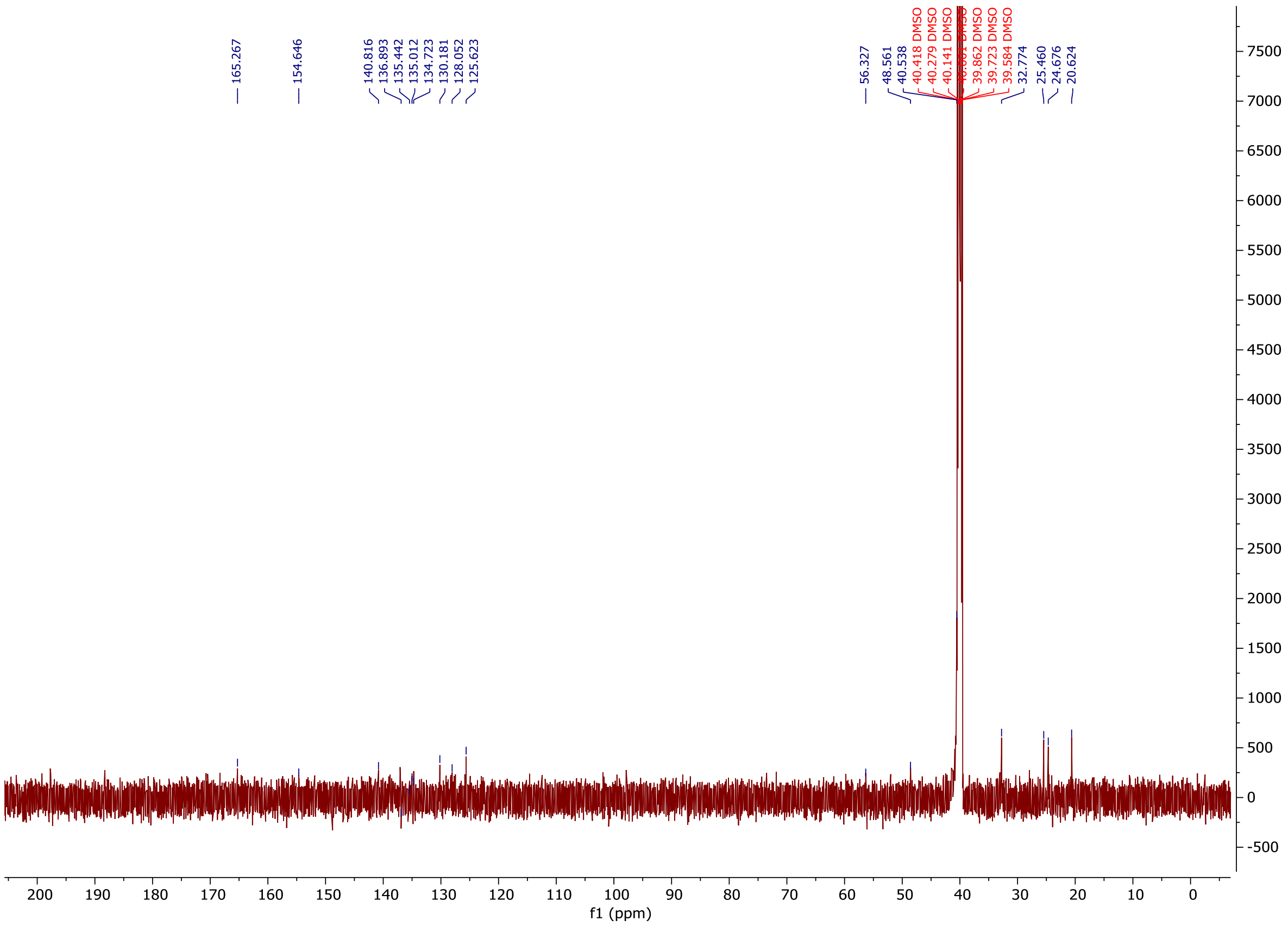

Figure S74 ^{13}C NMR spectrum of compound **67**

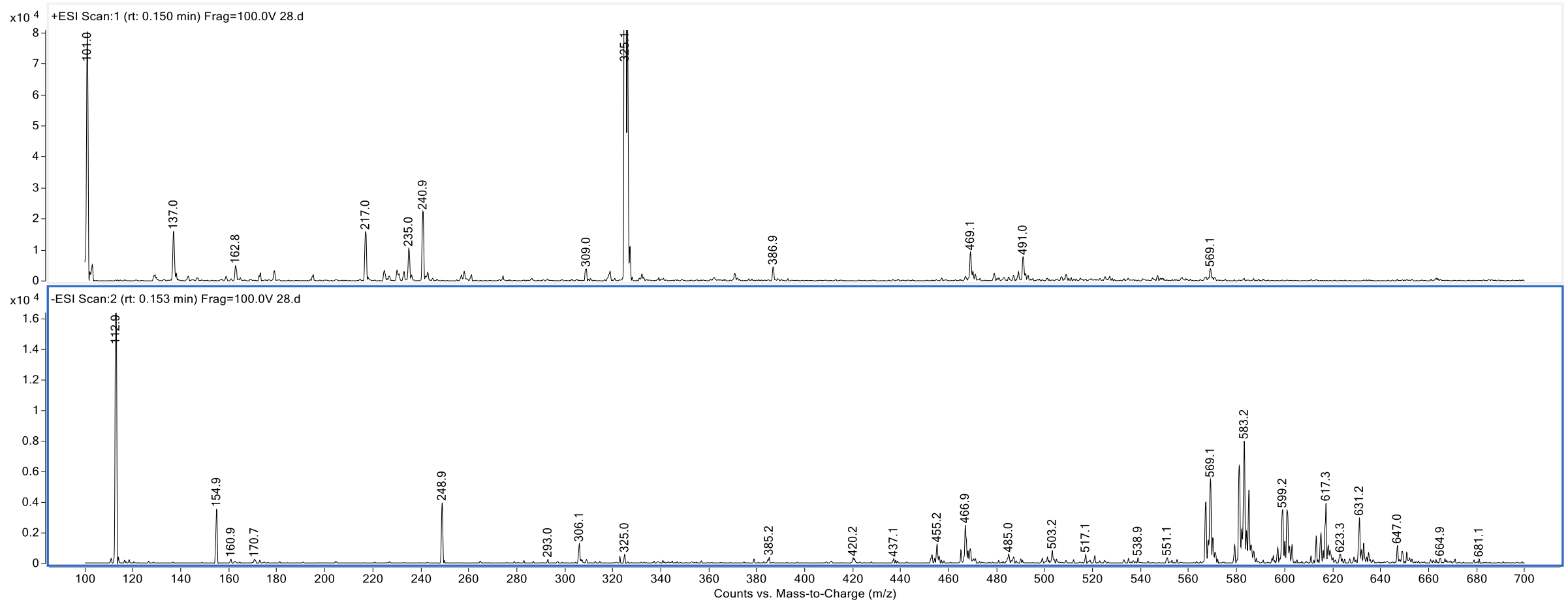

Figure S75. Mass spectrum of compound **67**

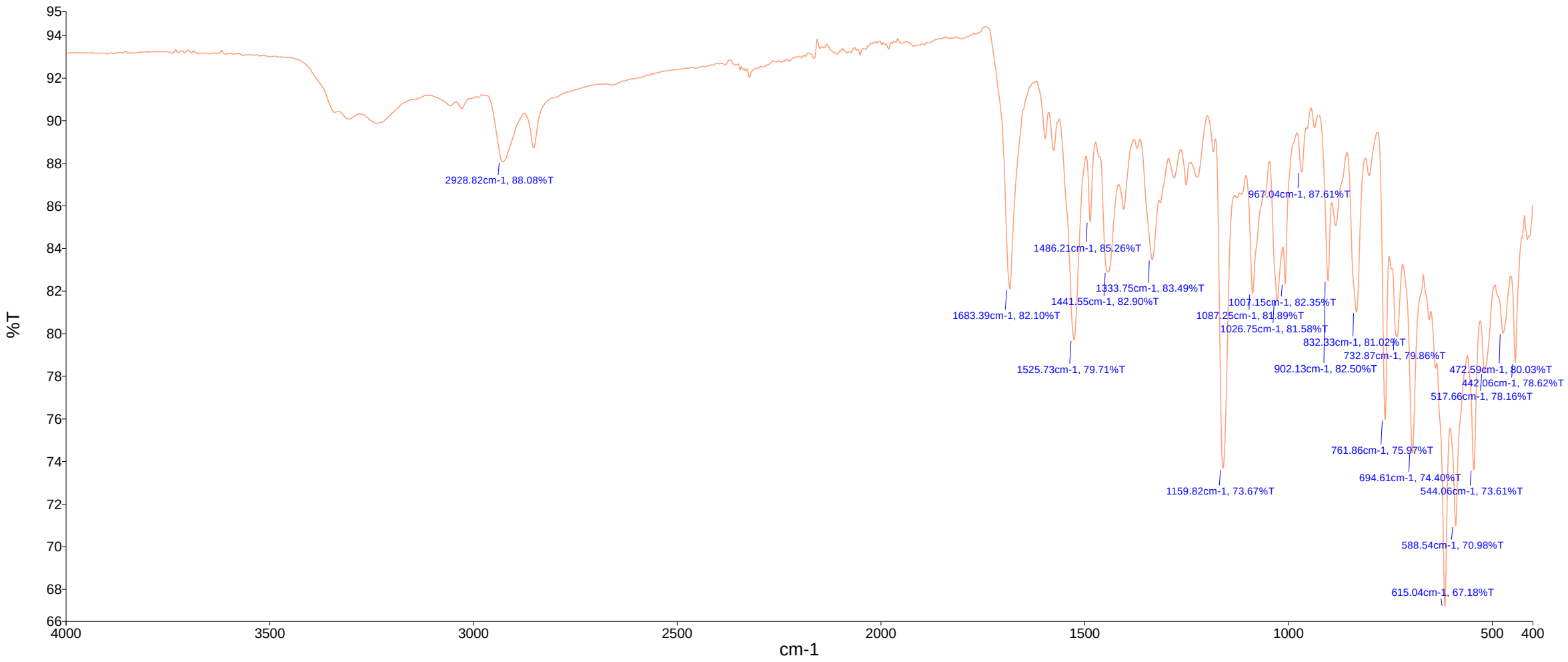

Figure S76. FTIR spectrum of compound **68**

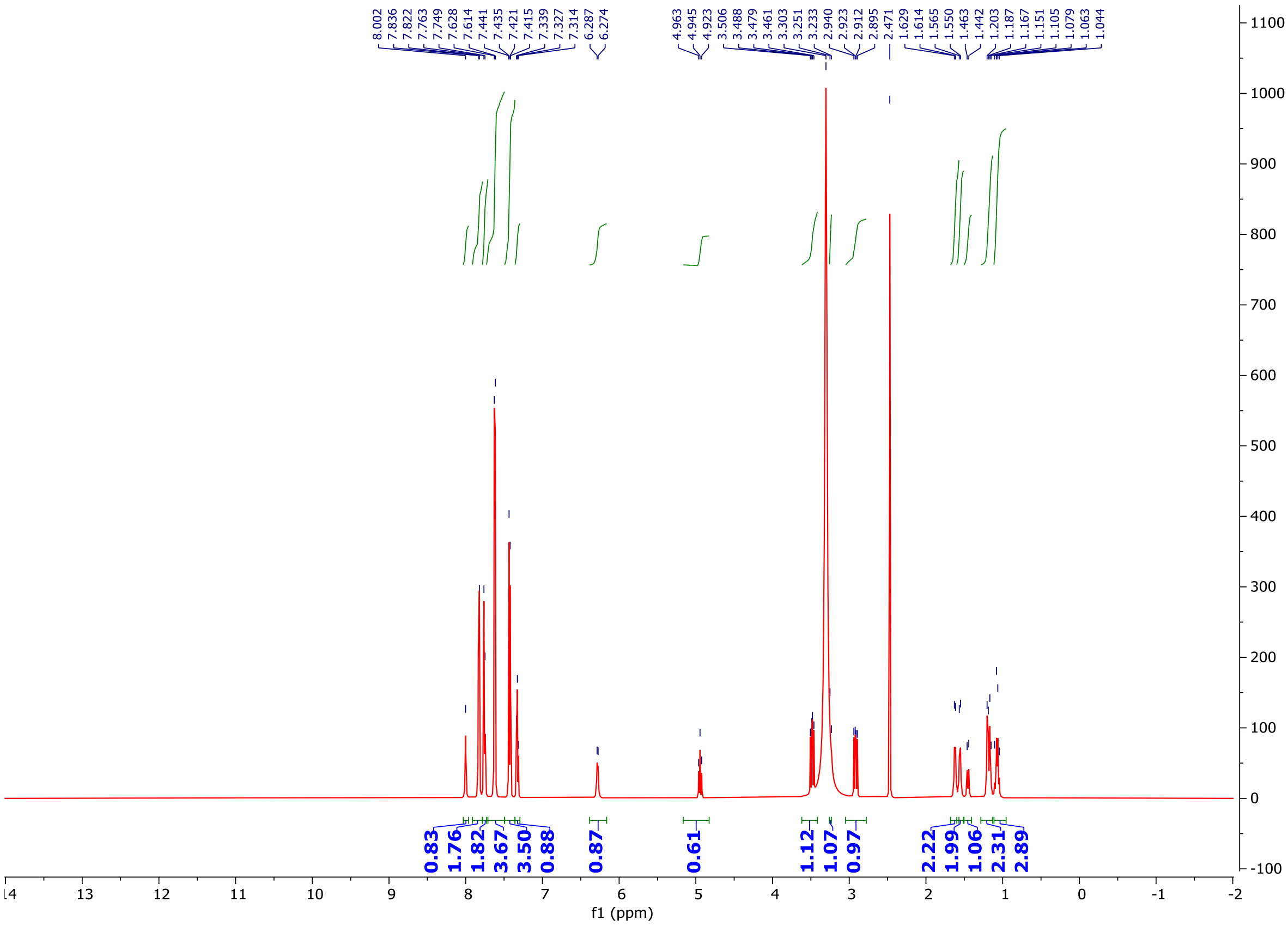

Figure S77. ^{1}H NMR spectrum of compound **68**

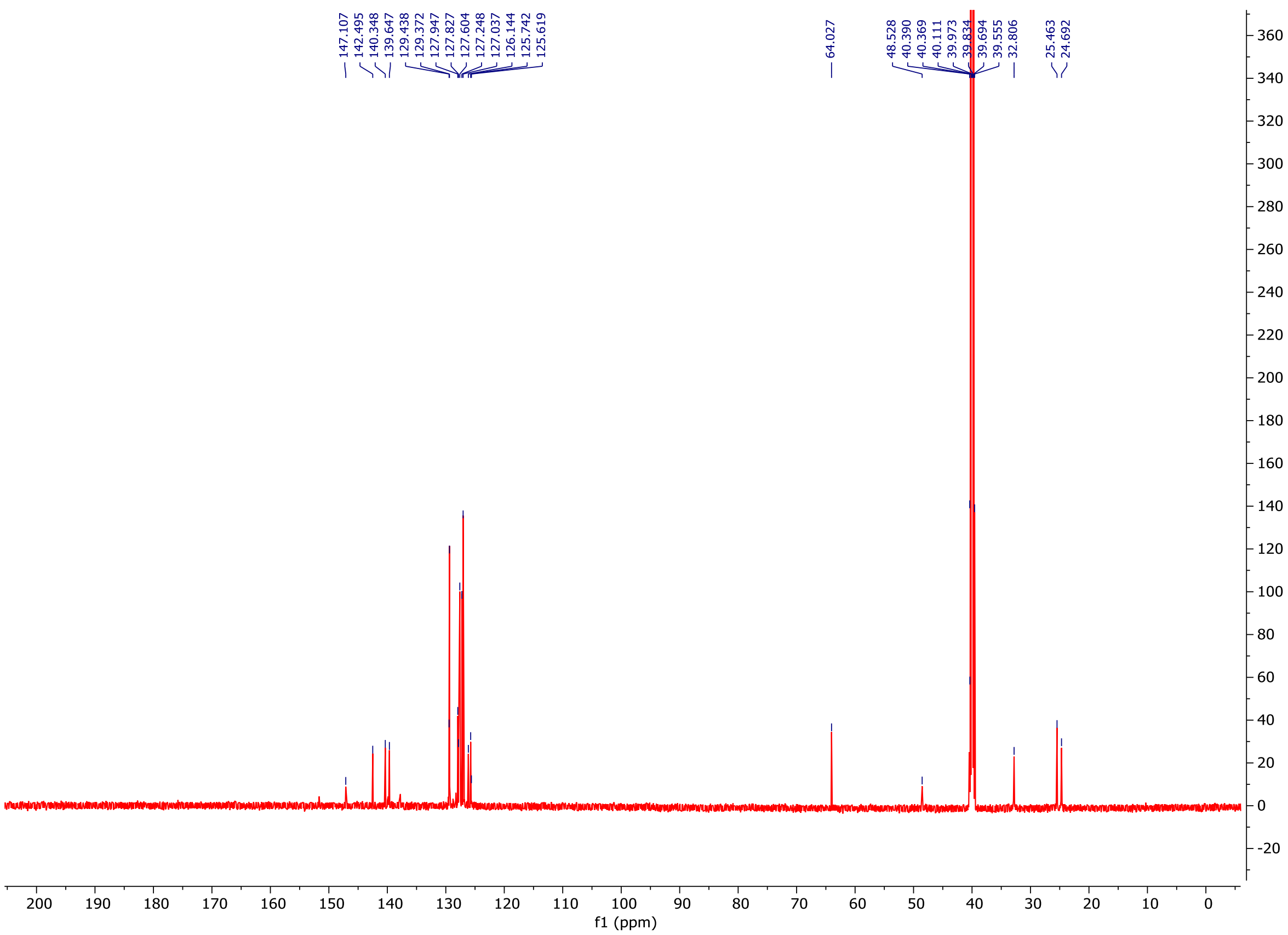

Figure S78. ^{13}C NMR spectrum of compound **68**

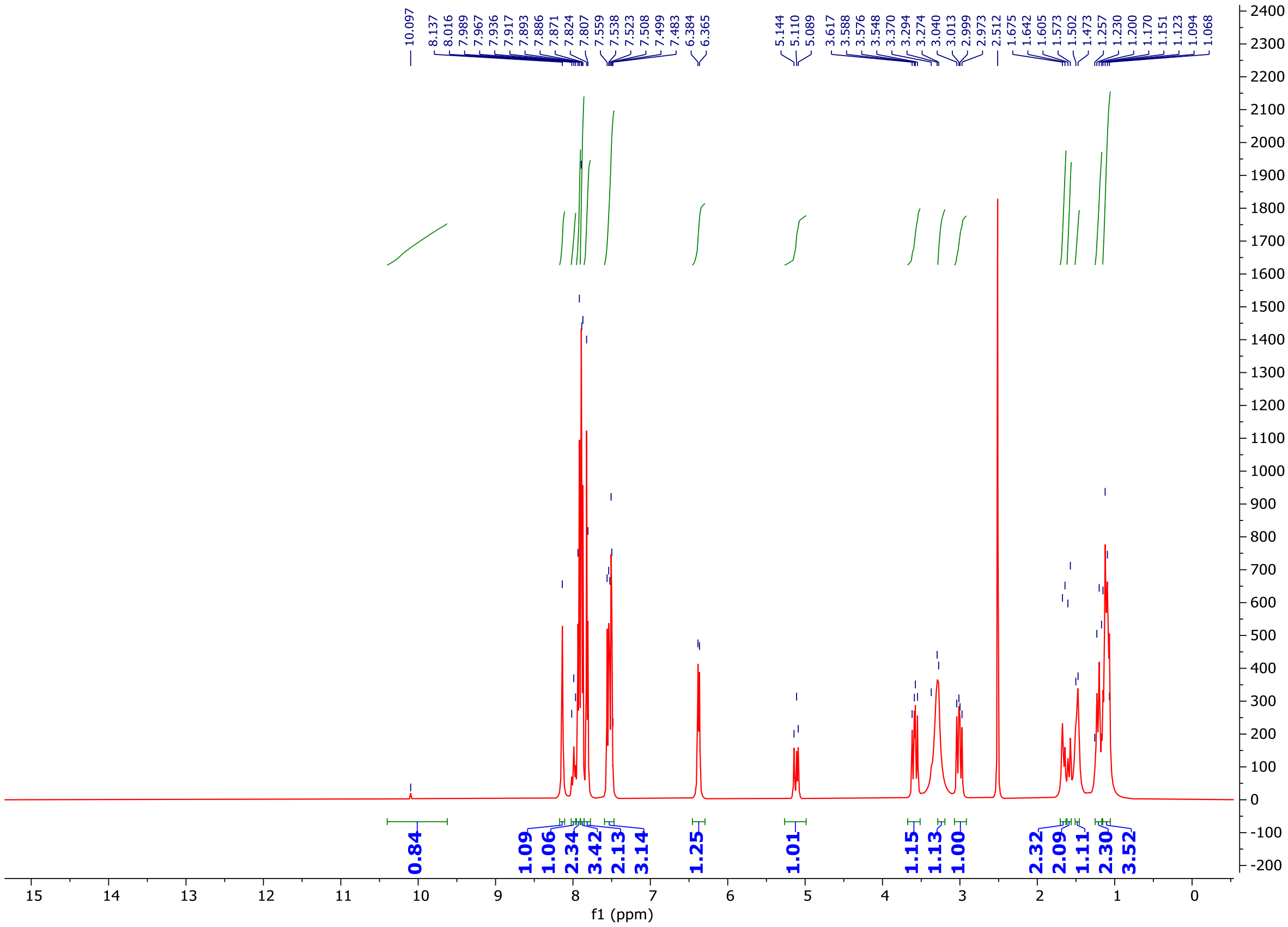

Figure S79. ^{1}H NMR spectrum of compound **70**

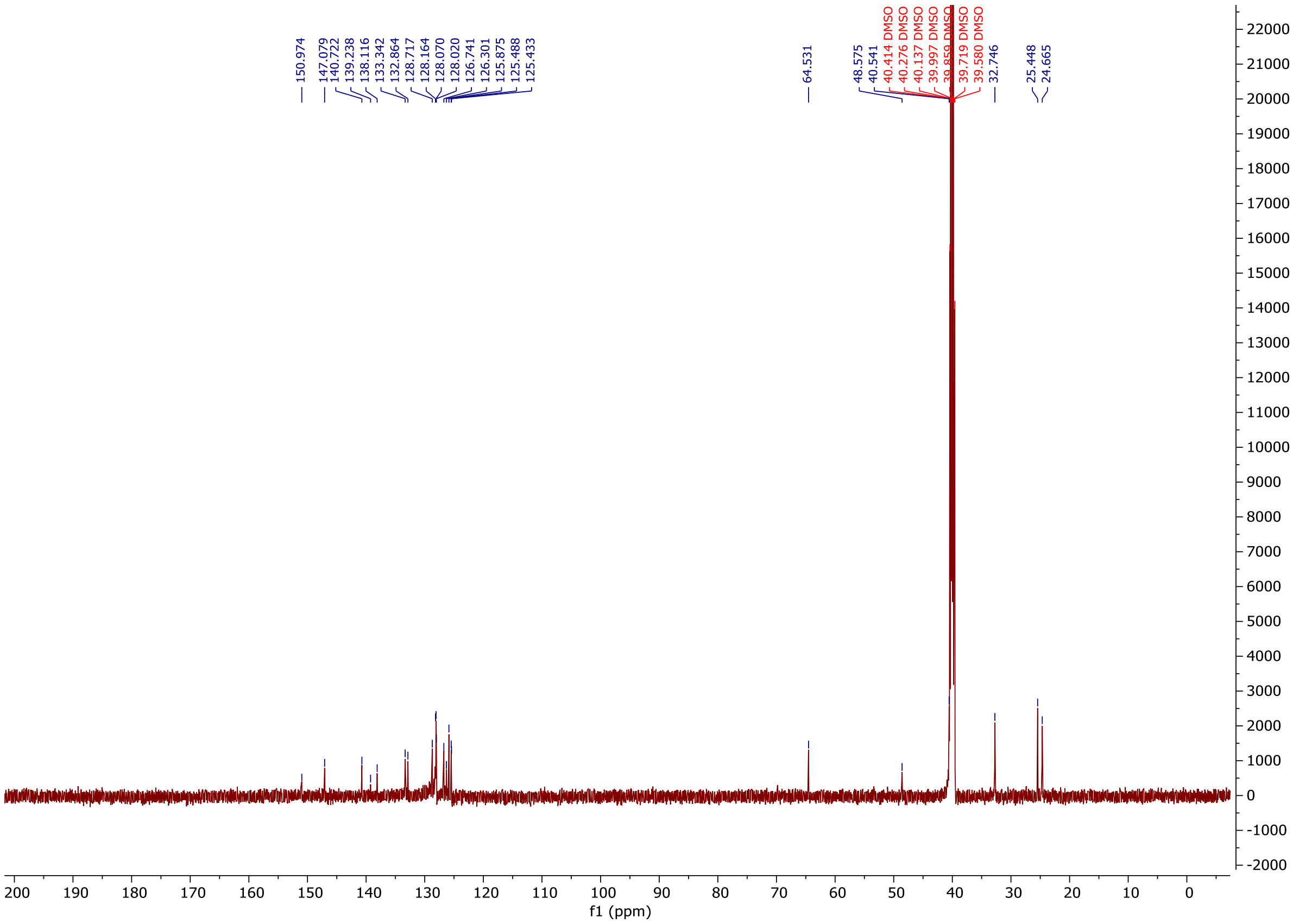

Figure S80. ^{13}C NMR spectrum of compound **70**

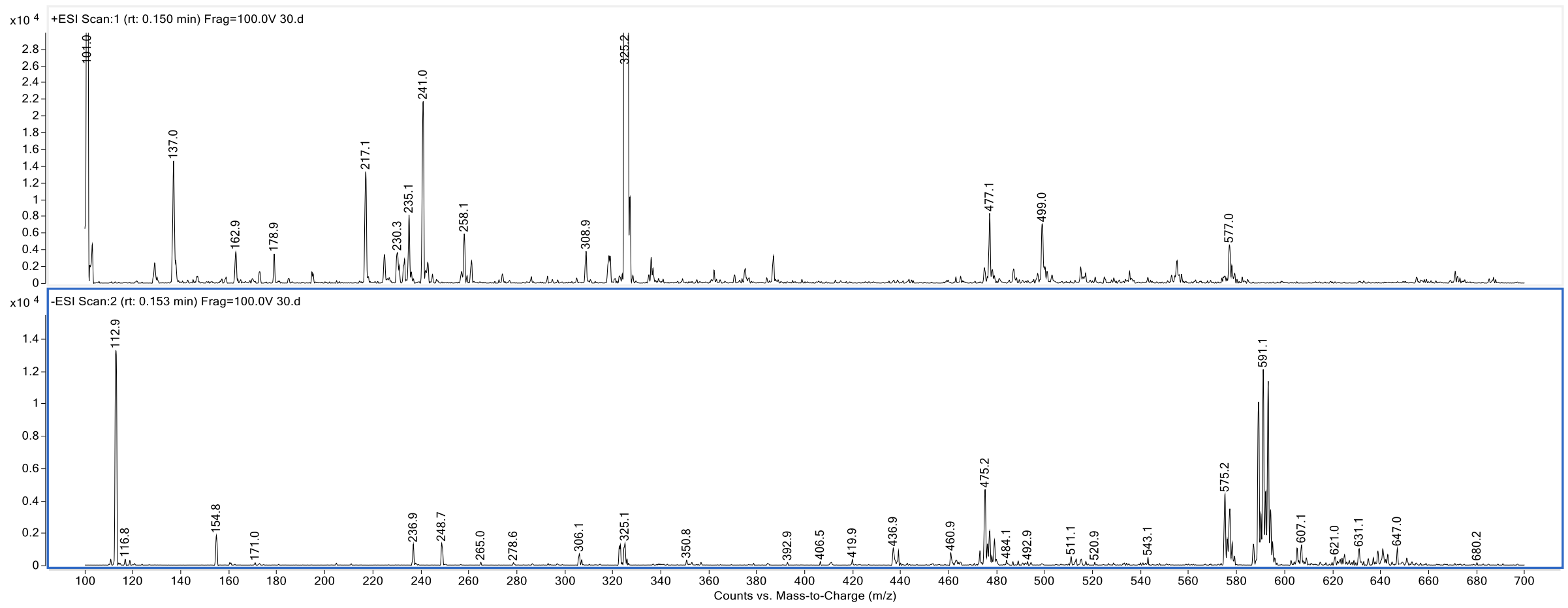

Figure S81. Mass spectrum of compound **70**

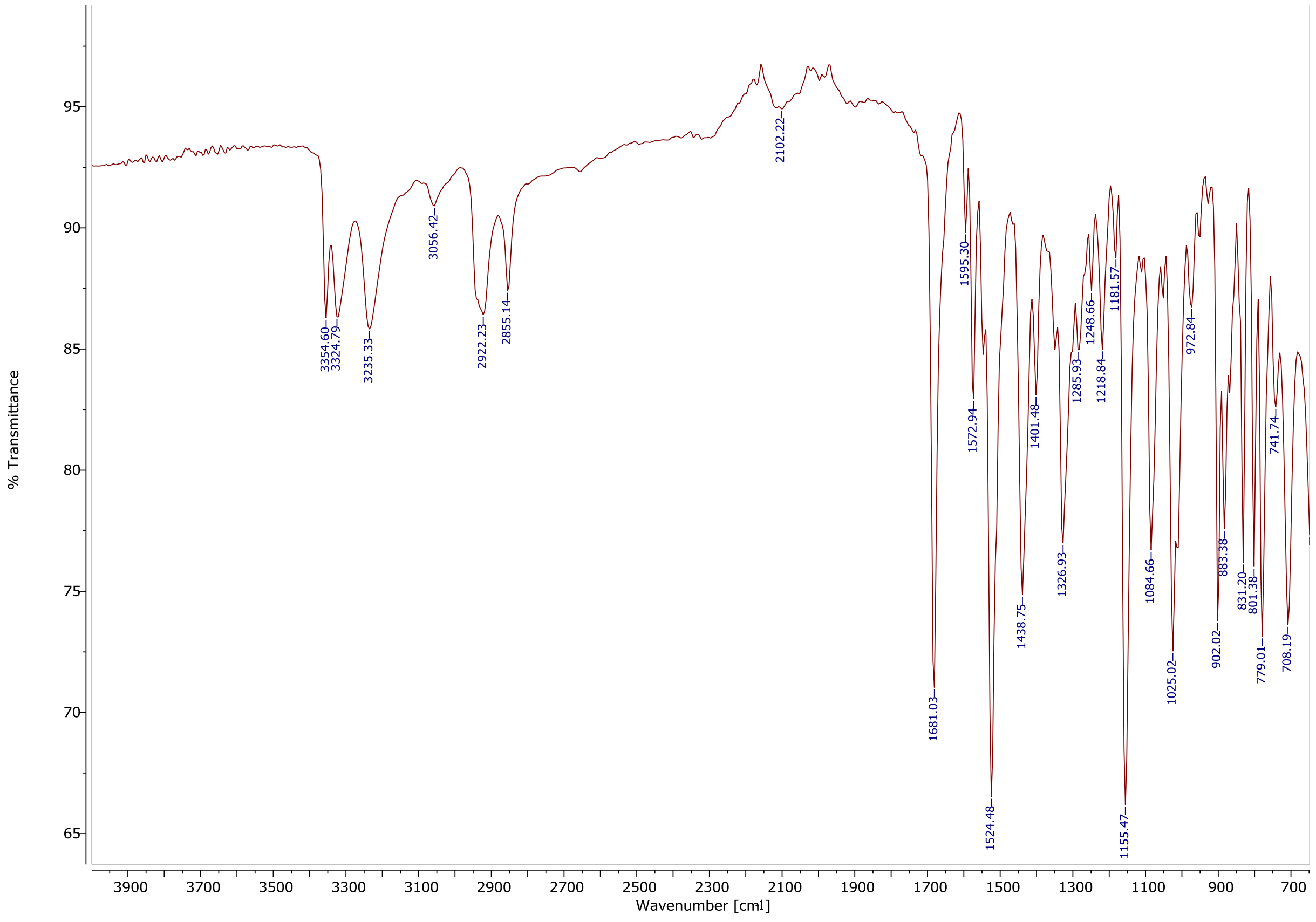

Figure S82. FTIR spectrum of compound **70A**

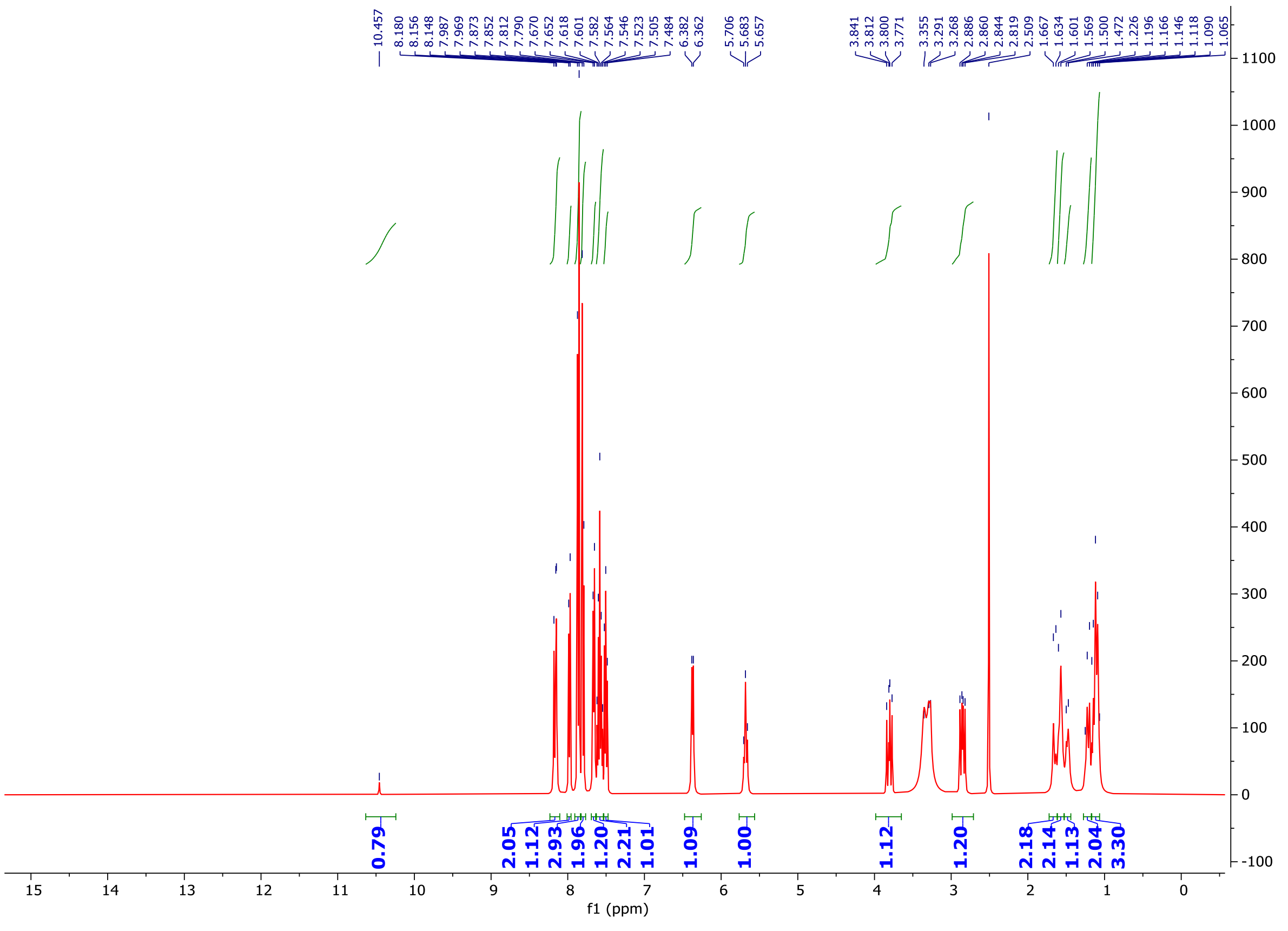

Figure S83. ^{1}H NMR spectrum of compound **70A**

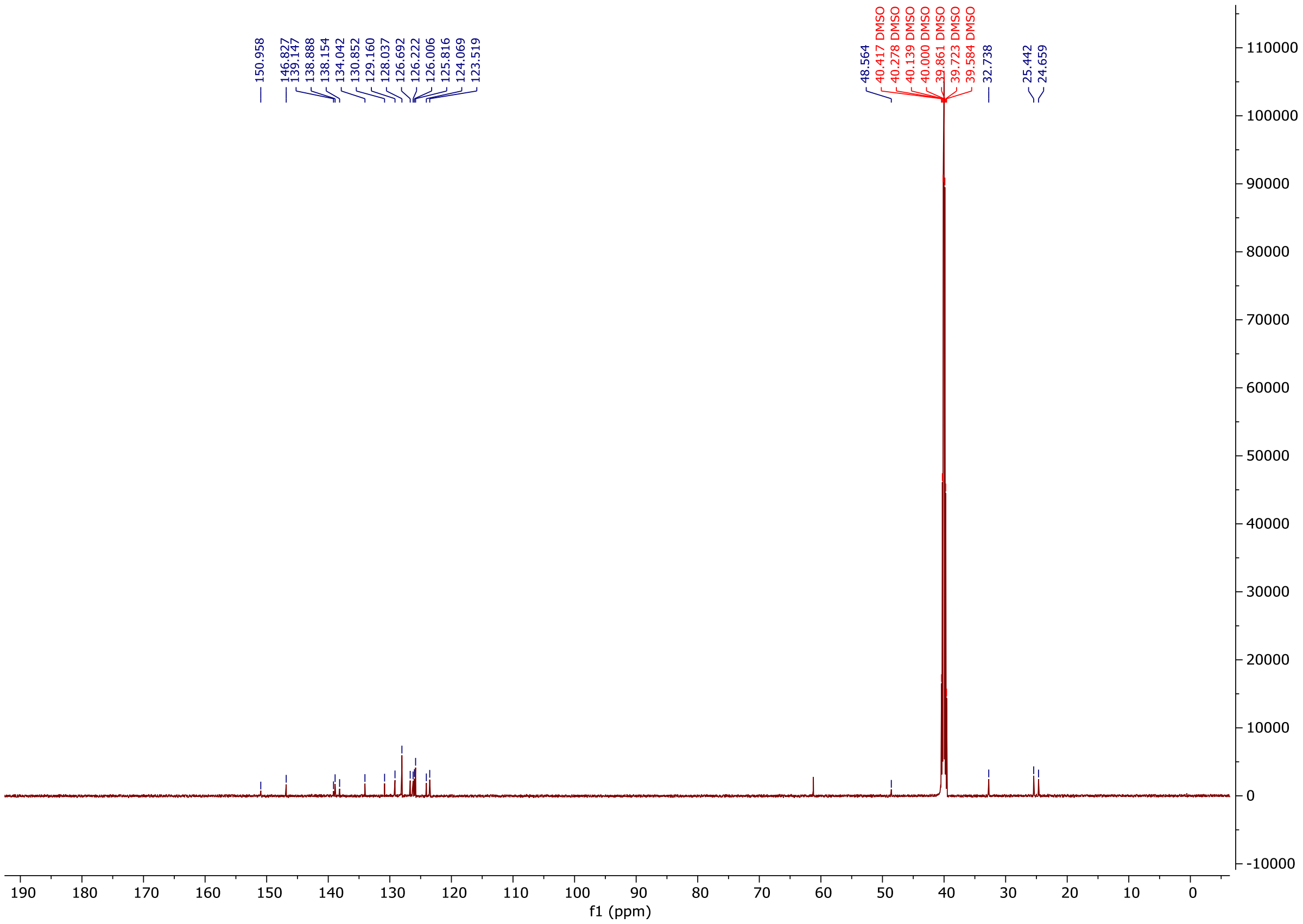

Figure S84 ^{13}C NMR spectrum of compound **70A**

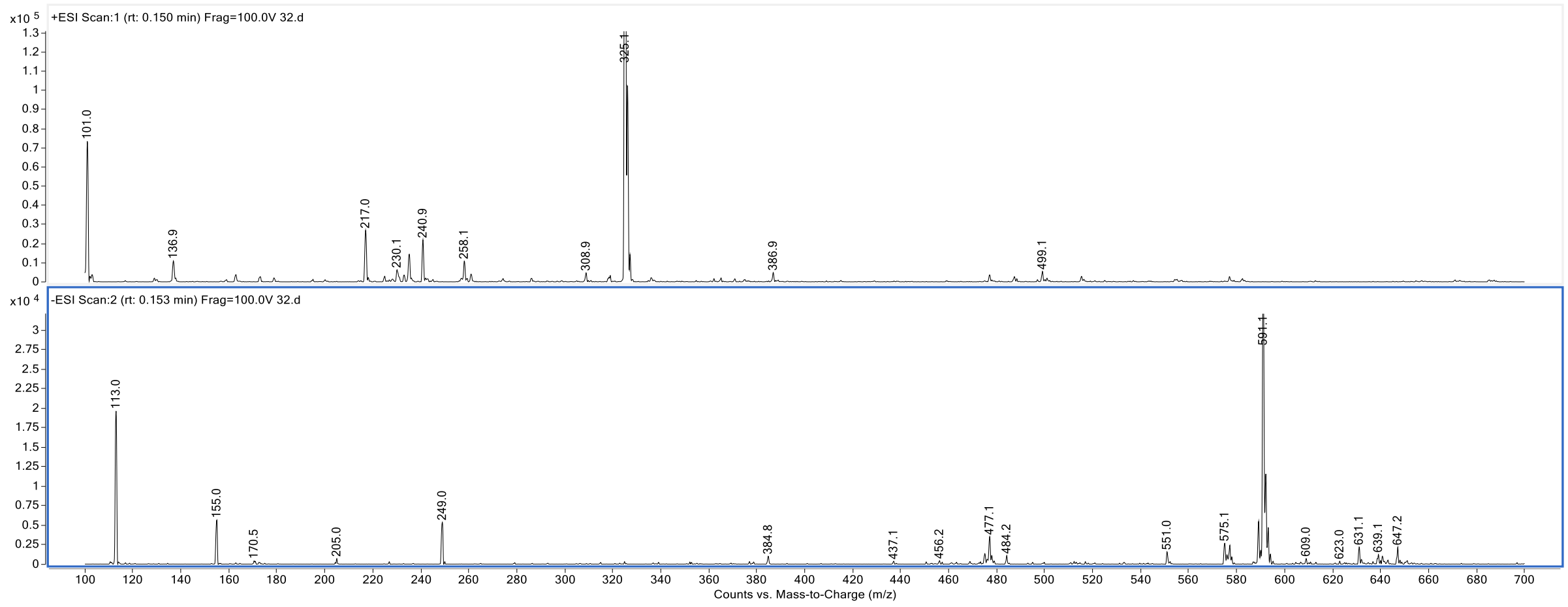

Figure S85. Mass spectrum of compound **70A**
